# Conformational control of Pd_2_L_4_ assemblies with unsymmetrical ligands†

**DOI:** 10.1039/c9sc05534g

**Published:** 2019-11-28

**Authors:** James E. M. Lewis, Andrew Tarzia, Andrew J. P. White, Kim E. Jelfs

**Affiliations:** Department of Chemistry, Imperial College London, Molecular Sciences Research Hub 80 Wood Lane London W12 0BZ UK james.lewis@imperial.ac.uk

## Abstract

With increasing interest in the potential utility of metallo-supramolecular architectures for applications as diverse as catalysis and drug delivery, the ability to develop more complex assemblies is keenly sought after. Despite this, symmetrical ligands have been utilised almost exclusively to simplify the self-assembly process as without a significant driving foa mixture of isomeric products will be obtained. Although a small number of unsymmetrical ligands have been shown to serendipitously form well-defined metallo-supramolecular assemblies, a more systematic study could provide generally applicable information to assist in the design of lower symmetry architectures. Pd_2_L_4_ cages are a popular class of metallo-supramolecular assembly; research seeking to introduce added complexity into their structure to further their functionality has resulted in a handful of examples of heteroleptic structures, whilst the use of unsymmetrical ligands remains underexplored. Herein we show that it is possible to design unsymmetrical ligands in which either steric or geometric constraints, or both, can be incorporated into ligand frameworks to ensure exclusive formation of single isomers of three-dimensional Pd_2_L_4_ metallo-supramolecular assemblies with high fidelity. In this manner it is possible to access Pd_2_L_4_ cage architectures of reduced symmetry, a concept that could allow for the controlled spatial segregation of different functionalities within these systems. The introduction of steric directing groups was also seen to have a profound effect on the cage structures, suggesting that simple ligand modifications could be used to engineer structural properties.

## Abstract 2

Steric and geometric constraints were used to design unsymmetrical ditopic ligands that form single Pd_2_L_4_ cage isomers with high fidelity.

## Graphical abstract

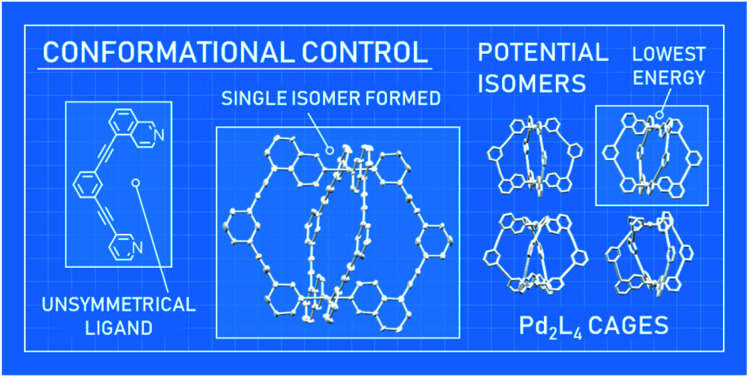

## Introduction

Non-covalent interactions are utilised by Nature to meticulously control self-assembly processes, ensuring that components combine in specific ratios and orientations to function effectively. For example, proteins can assemble with high fidelity into homo- or hetero-multimeric quaternary structures to carry out a range of biological functions.^1^ Over the last few decades chemists in various fields have attempted to emulate these principles, applying them to artificial systems.

Metallo-supramolecular chemistry entails the self-assembly of a combination of metal ions and ligands to form architectures of defined geometry.^2^ Following early seminal work by Lehn,^3^ Fujita^4^ and Stang^5^ amongst others, metallo-supramolecular architectures of myriad geometries have been prepared. To promote efficient self-assembly the majority of ligands used are symmetrical and give rise to highly symmetrical self-assembled structures.^6^

Since their first report by McMorran and Steel over two decades ago,^7^ Pd_2_L_4_ molecular cages^8^ – assembled from ditopic ligands and “naked” palladium(ii) ions – have been examined for a range of functions, including biomedical applications,^9^ catalysis^10^ and gas storage.^11^ Recently several examples have emerged in which two different ligands have been incorporated into cage structures in a controlled manner, resulting in heteroleptic complexes,^12^ either as a result of steric interactions,^13^ or through geometric design.^14^ This approach enables the preparation of cages with cavities that deviate from the standard approximately spherical shape generally associated with these complexes, and could potentially allow different segments of the cage to contain separate functionalities.^15^ In this manner cages could be prepared that, like enzymes, contain multiple binding sites.

**Fig. 1:**
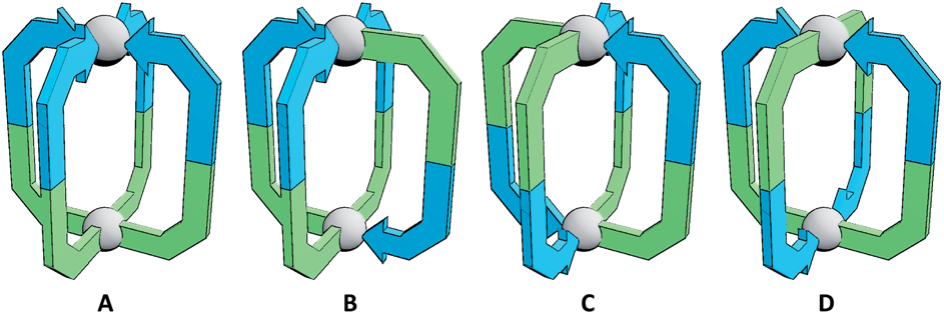
Cartoon representations of the four possible isomers of homoleptic Pd_2_L_4_ cages that can be formed with an unsymmetrical ditopic ligand: “all-up” (**A**), “three-up-one-down” (**B**), *cis* (**C**) and *trans* (**D**). Blue and green colours indicate non-equivalent ligand fragments.

However, functional group segregation could theoretically also be achieved with homoleptic assemblies through the controlled self-assembly of unsymmetrical ligands. Reports on the use of unsymmetrical ligands in metallo-supramolecular constructs,^16^ and three-dimensional polyhedra in particular,^17^ are rare. This is likely due to the fact that without a significant bias being built into the system, a statistical mixture of isomeric products will be obtained (Fig. 1). The selective formation of specific assemblies may be particularly challenging without resorting to ligand scaffolds incorporating coordinating moieties of different denticities, or the use of capping ligands to limit effective metal ion coordination numbers. However, it should be possible to design unsymmetrical ligands such that, upon self-assembly with metal ions, certain isomers of the resultant architectures are sufficiently energetically favourable to allow effective self-sorting into a single product. Indeed, whilst this manuscript was in preparation, Ogata and Yuasa reported an investigation into a stimuli-responsive [Pd_2_L_4_]^4+^ cage, capable of binding ReO_4_^−^ anions, that formed as a single isomer from an unsymmetrical carbazole-based ditopic ligand framework.^18^

Herein we report an initial exploration of unsymmetrical bis-monodentate ligands and their self-assembly with ‘naked’ Pd(ii) ions to form Pd_2_L_4_ cages. Despite the potential of forming a mixture of different isomers it is shown that, through careful design, *trans C*_2v_ and *cis C*_2h_-symmetry complexes can be formed preferentially due to steric or geometric control, or a combination of the two.

## Results and discussion

The simplest assemblies that can be formed from the coordination of unsymmetrical ditopic ligands, incapable of chelation, to square planar palladium(ii) ions are Pd_2_L_4_ architectures. Due to the lack of bilateral symmetry, the formation of four different isomers is possible (Fig. 1). We hypothesised that by using steric or geometric constraints, or a combination of the two, it should be possible to design systems in which one isomer in particular is sufficiently energetically favourable to be formed to the exclusion of the others, allowing predictable formation of specific cage isomers.

### Steric constraints

Initially we sought to direct the self-assembly of unsymmetrical ligands using steric control, inspired by recent work on heteroleptic metallo-assemblies. Crowley and co-workers have reported the displacement of unsubstituted di-pyridyl ligands from pre-formed homoleptic Pd_2_L_4_ cages with *ortho*-aminopyridyl ligands, resulting in Pd_2_L_2_L′_2_ architectures.^13*a*^ Similar Pd(ii) cages were prepared by Clever and co-workers using a combination of picolyl-derived ligands with exo- or endo-hedral methyl groups to sterically enforce the formation of heteroleptic structures.^13*b*^ Ligand **1** was designed around the well-known *m*-bis(pyridin-3-ylethynyl)aryl ligand motif, with a 6-methyl substituent appended to one of the pyridine rings, and prepared using successive Sonogashira reactions.^19^

**Fig. 2:**
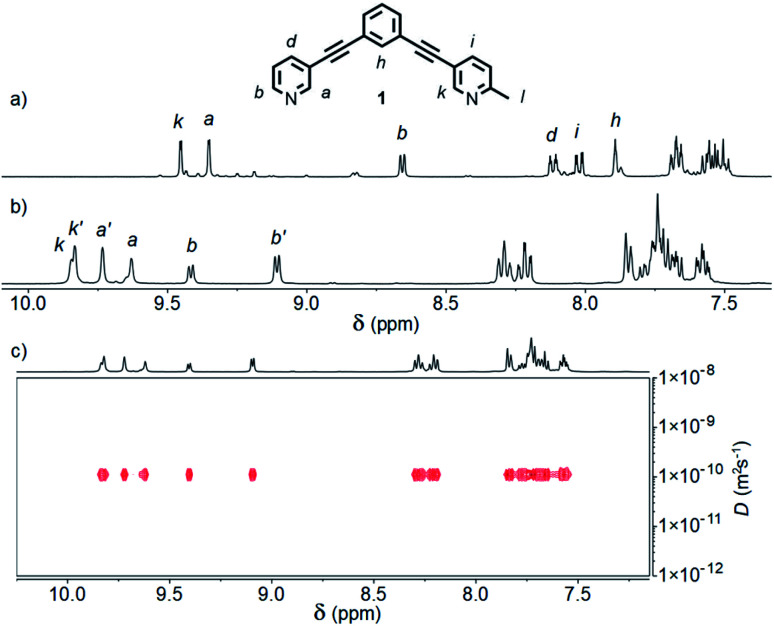
Partial ^1^H NMR spectra (400 MHz, 298 K) of cages [Pd_2_(**1**)_4_](BF_4_)_4_ formed in (a) CD_3_CN, and (b) *d*_6_-DMSO; (c) ^1^H DOSY spectrum (500 MHz, *d*_6_-DMSO) of *cis*- and *trans*-[Pd_2_(**1**)_4_](BF_4_)_4_ cage mixture.

Initially combining **1** with [Pd(CH_3_CN)_4_](BF_4_)_2_ in a 2 : 1 ratio in CD_3_CN at room temperature resulted in a mixture of products (Fig. S88†). Pleasingly, heating at 60 °C for 24 h resulted in coalescence to a single major set of signals (Fig. 2a).^20^ DOSY NMR (*D* = 8.10 × 10^−10^ m^2^ s^−1^; calculated hydrodynamic radius, *R*_H_, of 7.0 Å) and mass spectrometry (MS; *m*/*z* = 781 [Pd_2_(**1**)_4_(BF_4_)_2_]^2+^, Fig. S86†) indicated that a Pd_2_L_4_ assembly had been formed. To confirm that the observed simple ^1^H NMR spectrum was the result of a single cage isomer, rather than multiple rapidly interconverting species, a variable temperature NMR experiment was performed with ^1^H NMR spectra obtained between 20 and −30 °C (Fig. S89†). Even at −30 °C no significant broadening or splitting of the NMR signals could be observed, supporting the conclusion that a single thermodynamically stable cage had been formed.

**Fig. 3:**
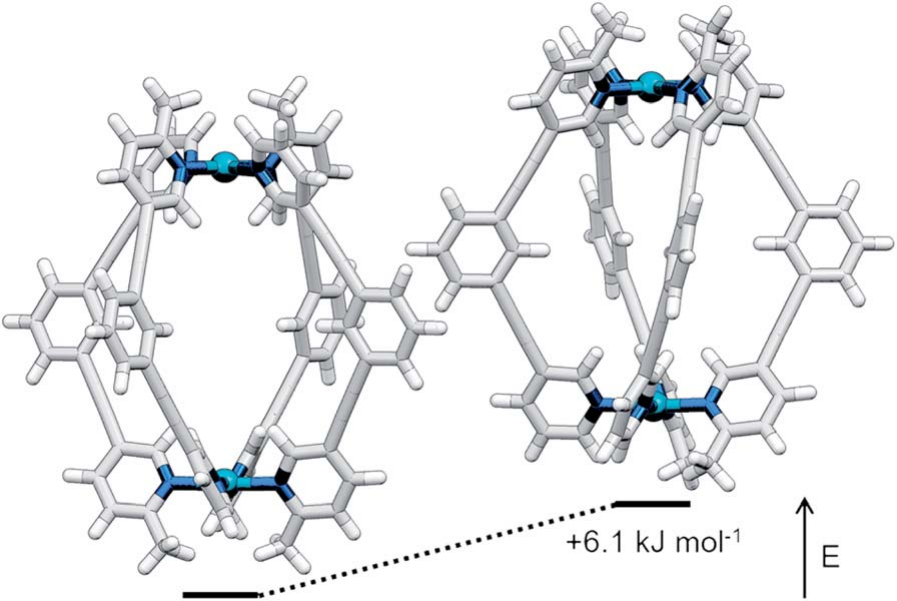
Calculated structures (B3LYP/6-31G(d)/SDD) and relative energies of *trans*-[Pd_2_(**1**)_4_]^4+^ (left) and *cis*-[Pd_2_(**1**)_4_]^4+^ (right).

The symmetry of the ^1^H NMR spectrum suggested that the product could not be isomer **B**, whilst cross-peaks observed in the ROESY NMR spectrum (Fig. S84†) between the methyl group (H_l_) of one pyridine ring and the exohedral proton *ortho* to the pyridyl nitrogen atom of the other (H_b_) ruled out isomer **A**. Unfortunately, despite multiple attempts, we were unable to generate X-ray quality crystals of the complex to confirm its structure in the solid state. We therefore turned to density functional theory (DFT) calculations (carried out at the B3LYP/6-31G(d) level of theory using the D3 empirical dispersion correction and the Stuttgart–Dresden (SDD) effective core potentials for Pd(ii); see ESI† for details) which were performed to rank the *cis* (**C**) and *trans* (**D**) isomers in terms of their relative energies (Table S1†). These calculations suggested that the *trans*-Pd_2_L_4_ cage was the thermodynamically favoured species by 6.1 kJ mol^−1^ (Fig. 3), and thus the likely product. The calculated structures revealed significant differences in the C⋯C distance between methyl groups in the two isomers (3.7 Å for the *cis*, 4.6 Å for the *trans*), corroborating the hypothesis that steric interactions between the methyl groups drive the observed isomeric bias.

Interestingly, when the self-assembly was repeated in *d*_6_-DMSO, two species were formed in an approximately 2 : 3 ratio (Fig. 2b). Continued heating at 60 °C for a prolonged period failed to further simplify the spectrum and ultimately led to degradation of the sample. Both species displayed the same diffusion coefficient (Fig. 2c) which, combined with MS data, indicated exclusive formation of Pd_2_L_4_ assemblies. In this instance, however, a mixture of two major isomers appeared to have formed. ROESY and TOCSY NMR (Fig. S74 and S75,† respectively) were used to assign signals as much as possible to the individual species; the former revealed cross-peaks between H_b_ and H_l_ for both, indicating that the mixture was composed of the *cis* (**C**) and *trans* (**D**) cages.

DFT calculations in implicit DMSO and MeCN (Table S1†) showed no difference in the relative energies of the *cis* and *trans* isomers in the two solvents, with the *trans* architecture remaining lower in energy. Unsurprisingly the calculations did show the higher energy *cis* structure to be the more polar of the two (based on a larger dipole moment from the DFT optimised structure; Table S1†), and it seems plausible that the difference in speciation observed between the two solvents could be related to this. In less polar acetonitrile, the more energetically favourable of the two cage isomers, *i.e. trans*-[Pd_2_(**1**)_4_](BF_4_)_4_, is formed exclusively; in DMSO the more polar *cis*-[Pd_2_(**1**)_4_](BF_4_)_4_ is stabilised by the increased polarity of the solvent.^21^ In the absence of more persuasive evidence, however, we remain cautious in our explanation of the observed effect on speciation in different solvents.

Thus we have shown that it is possible to use simple steric constraints, in this instance the inclusion of a single methyl group, to control conformational bias in the self-assembly of an unsymmetrical ligand. It was also discovered that this particular system is susceptible to the environment in which it is placed. In this instance, changing the solvent had a dramatic effect on the ratio of cage isomers observed at equilibrium. This sort of solvent-responsive ligand rearrangement in metallo-supramolecular systems is relatively uncommon^22^ and, once better understood, could potentially be exploited for adaptable constructs that change conformation upon exposure to different environments.^23^

### Geometric complementarity

**Fig. 4:**
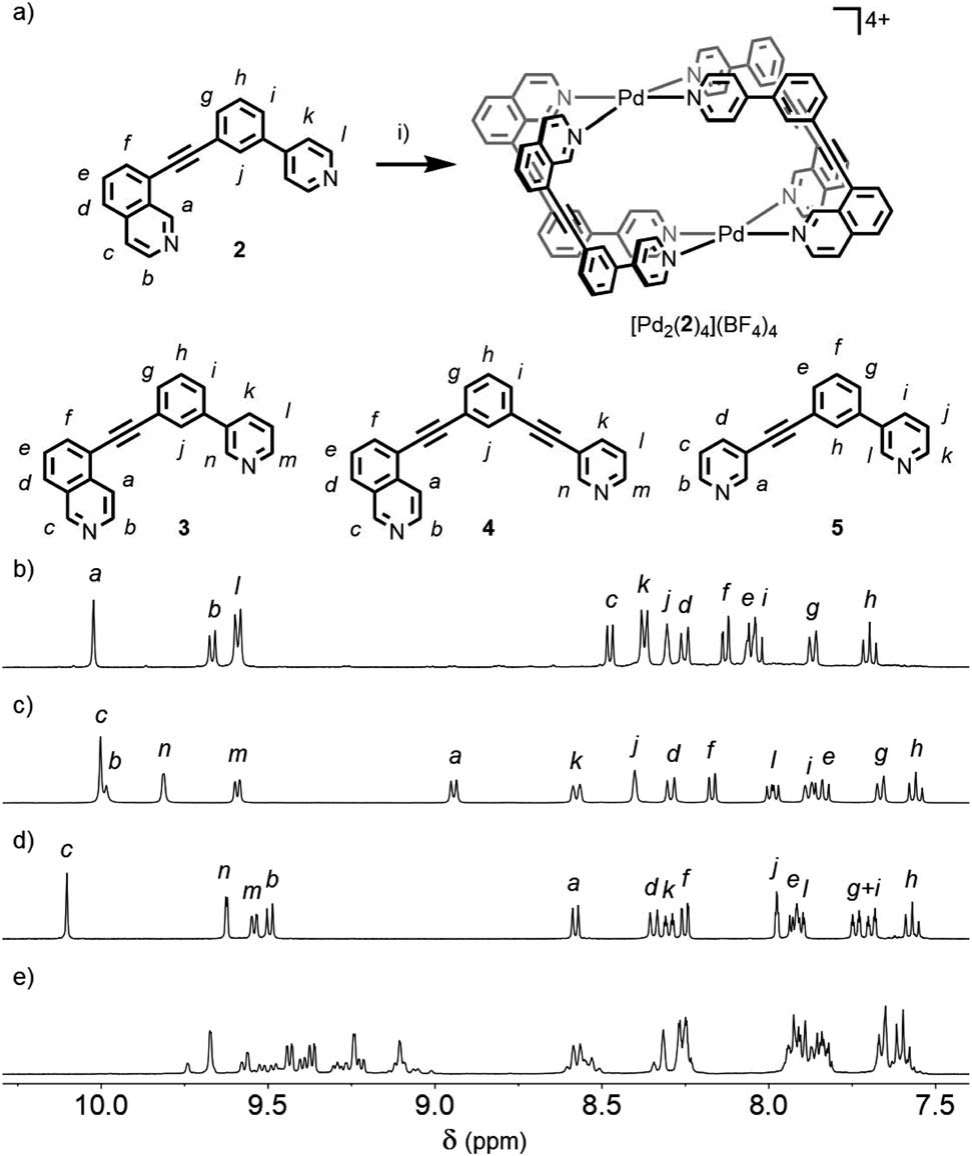
(a) Formation of the *cis*-Pd_2_L_4_ cage [Pd_2_(**2**)_4_](BF_4_)_4_ and structures of ligands **3**, **4** and **5**. Reagents and conditions: (i) [Pd(CH_3_CN)_4_](BF_4_)_2_, *d*_6_-DMSO, rt, 2 h. ^1^H NMR spectra (400 MHz, 298 K, *d*_6_-DMSO) of (b) [Pd_2_(**2**)_4_](BF_4_)_4_, (c) [Pd_2_(**3**)_4_](BF_4_)_4_, (d) [Pd_2_(**4**)_4_](BF_4_)_4_ and (e) equilibrated mixture of **5** and [Pd(CH_3_CN)_4_](BF_4_)_2_.

Subsequently we looked to assemble lower symmetry systems in which the design of the ligand geometry would enforce the assembly of a *cis*-Pd_2_L_4_ cage. To this end ligand **2** was prepared incorporating one isoquinoline and one pyridyl donor (Fig. 4a). This ligand was designed such that the planes orthogonal to the donor nitrogen atoms were no longer coincident with one another. Thus formation of a Pd_2_L_4_ species, entropically more favourable than assemblies of higher nuclearity, should occur with ligands arranged in a *cis* fashion (Fig. 4a).

**Fig. 5:**
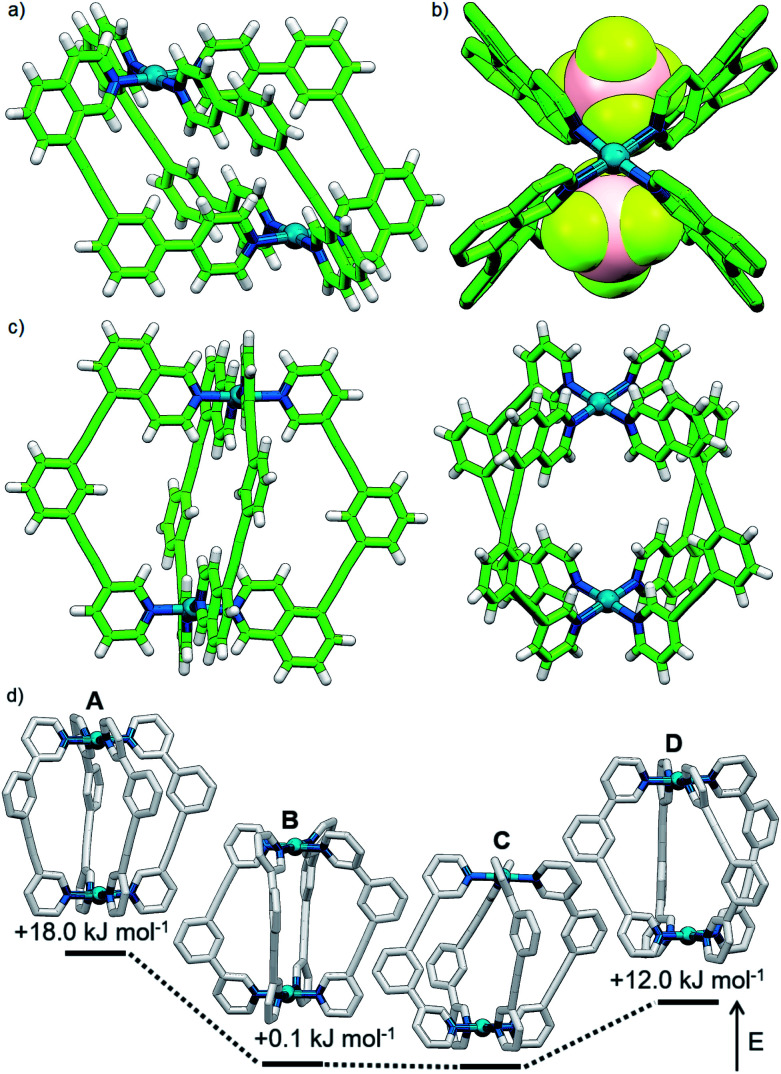
Solid state structure of *cis*-[Pd_2_(**2**)_4_](BF_4_)_4_ viewed (a) from the side and (b) down the Pd–Pd axis with encapsulated BF_4_^−^ anions shown (hydrogen atoms omitted for clarity). Bond lengths (Å): Pd–N_pyridine_ 1.990(17)/2.069(2); Pd–N_isoquinoline_ 2.029(3)/2.040(3). (c) Solid state structure of *cis*-[Pd_2_(**4**)_4_](BF_4_)_4_. Bond lengths (Å): Pd–N_pyridine_ 2.044(8)/2.056(8); Pd–N_isoquinoline_ 2.006(9)/2.007(8). (d) Calculated structures (B3LYP/6-31G(d)/SDD) of the **A**, **B**, **C** and **D** isomers of [Pd_2_(**5**)_4_]^4+^ with energies relative to **C**. Hydrogen atoms have been omitted for clarity.

Mixing **2** in *d*_6_-DMSO with [Pd(CH_3_CN)_4_](BF_4_)_2_ resulted in clean formation of a single cage, [Pd_2_(**2**)_4_](BF_4_)_4_, within 2 h at room temperature. The identity of the assembly was confirmed by DOSY NMR (*R*_H_ = 8.7 Å) and MS, and specific formation of the *cis* isomer (**C**) demonstrated in the solid state by single-crystal X-ray diffraction (SCXRD) (Fig. 5a). In the solid state the cage cavity was found to encapsulate two BF_4_^−^ anions (Fig. 5b), with the remaining counterions occupying exohedral sites adjacent to the Pd(ii) ions of the cage. To probe how significant the deviation from co-planarity the donor atoms needed to be to ensure quantitative assembly to a single *cis*-Pd_2_L_4_ species, the series of ligands **3**, **4** and **5** (Fig. 4a) was synthesised for comparison.

In the case of ligand **3**, an isomer of **2**, addition of 0.5 equivalents of [Pd(CH_3_CN)_4_](BF_4_)_2_ in *d*_6_-DMSO also resulted in clean formation of a single Pd_2_L_4_ species within 2 h at room temperature as observed by NMR (Fig. 4c) and MS. For ligand **4** equilibration was slower; however, within 24 h a single species was formed cleanly (Fig. 4d) and the expected Pd_2_L_4_ structure with *cis* ligand arrangement confirmed in the solid state by SCXRD (Fig. 5c). DFT calculations (Table S2†) supported the *cis*-[Pd_2_(**4**)_4_]^4+^ isomer as being the lowest energy species by 4.4–26.8 kJ mol^−1^ compared to the other three potential isomers. The “all-up” **A** configuration was determined to be the highest energy isomer, followed by the *trans***D** assembly, with isomer **B**, in which three of the ligands are orientated in the same direction, the second lowest energy structure.

For **5**, however, even after prolonged heating at 60 °C in *d*_6_-DMSO,^24^ the ^1^H NMR spectrum (Fig. 4e) failed to converge into a single set of signals. DOSY NMR (Fig. S117†) indicated that the assemblies formed were of a similar size, with the calculated *R*_H_ congruent with Pd_2_L_4_ architectures (8.6 Å), suggesting the formation of a mixture of dinuclear cage isomers.

The calculated structural isomers of [Pd_2_(**5**)_4_]^4+^ (Fig. 5d; Table S2†) exhibited the same trend in relative energies as with [Pd_2_(**4**)_4_]^4+^, *i.e.***C** < **B** < **D** < **A**. In contrast, however, the **D** isomer was higher in relative energy (12.0 kJ mol^−1^) whilst the difference in energies between the **B** and **C** isomers was not found to be significant (0.1 kJ mol^−1^), indicating that a mixture of at least the **B** and **C** isomers would potentially result from the equilibrium mixture of **5** and Pd(ii). Isomer **B** would also give the most complicated NMR spectrum due to possessing three different ligand environments. As such a mixture of isomers **B** and **C** would be expected to give a complex spectrum in accord with the observed NMR data; unfortunately severely overlapping signals prohibited detailed analysis of the equilibrated mixture.

### Combined steric and geometric control

**Fig. 6:**
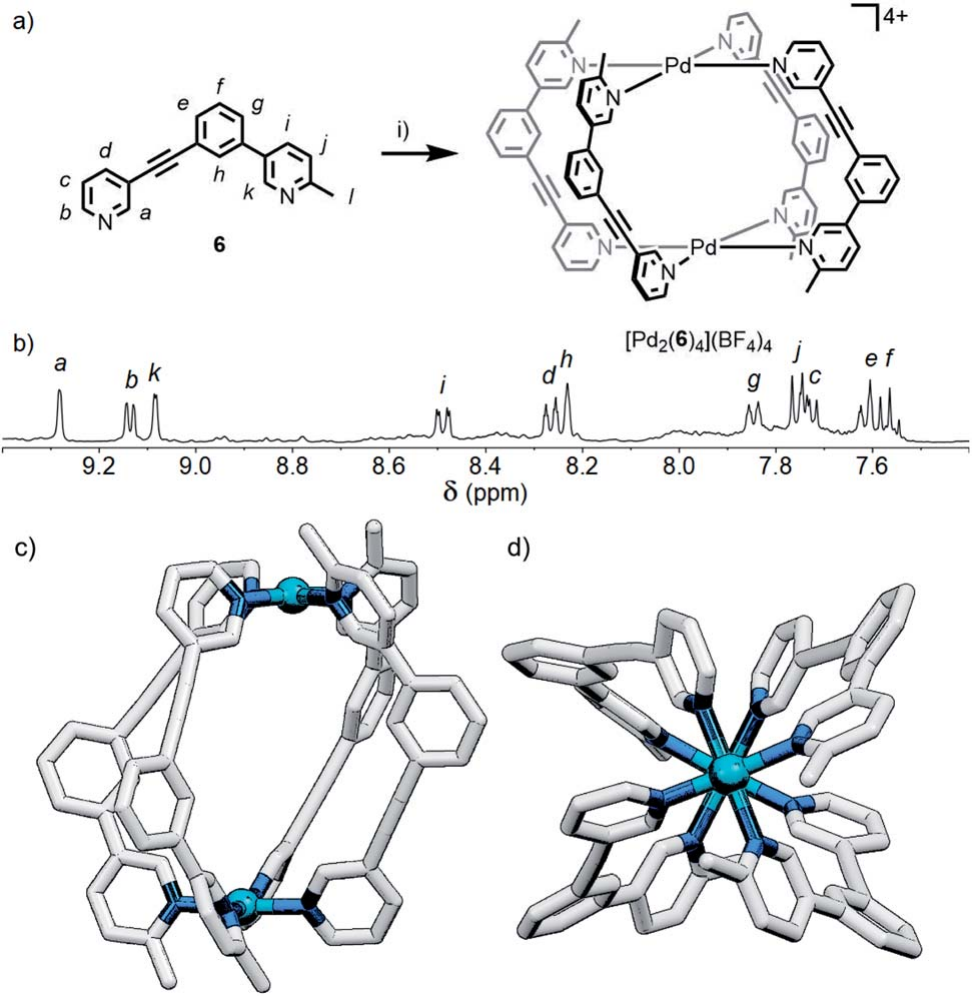
(a) Formation of the *cis*-Pd_2_L_4_ cage [Pd_2_(**6**)_4_](BF_4_)_4_. Reagents and conditions: (i) [Pd(CH_3_CN)_4_](BF_4_)_2_, *d*_6_-DMSO, rt, 24 h. (b) Partial ^1^H NMR spectrum (400 MHz, 298 K, *d*_6_-DMSO) of [Pd_2_(**6**)_4_](BF_4_)_4_. Calculated structure (B3LYP/6-31G(d)/SDD) of *cis*-[Pd_2_(**6**)_4_]^4+^ viewed from (c) the side, and (d) down the Pd–Pd axis; hydrogen atoms have been omitted for clarity.

Although the geometric constraint of ligand **5** was insufficient to give clean formation of a single cage isomer, it was hypothesised that in combination with a steric factor the **B** isomer could become sufficiently raised in energy to allow selective formation of the *cis* (**C**) assembly. To this end, ligand **6** (Fig. 6a) was synthesised in which one of the pyridyl moieties was replaced with a 2-picolyl unit; pleasingly, upon combination with Pd(ii) ions, a single species was observed to form by ^1^H NMR in *d*_6_-DMSO within 24 h (Fig. 6b). ^1^H, ROESY and DOSY (*R*_H_ = 8.7 Å) NMR (Fig. 6b, S128 and S129,† respectively) and MS data all indicated that either the *cis*- or *trans*-[Pd_2_(**6**)_4_](BF_4_)_4_ cage was formed as the sole product of self-assembly. As the *cis* (**C**) and *trans* (**D**) isomers could not be distinguished spectroscopically, their relative energies were calculated by DFT (Table S2†), with the *cis* assembly (Fig. 6c and d) determined to be the lower energy isomer by 12.2 kJ mol^−1^. We have therefore shown that it is possible to augment a particular geometric ligand design with a sterically influencing group to drive selective formation of a single species when one factor exerts insufficient bias by itself.

### Effect of steric directing groups on cage conformation

Ligands **5** and **6** differ only in the presence of a single methyl group, yet this small change meant the difference between formation of a single cage isomer or a mixture of isomers upon complexation with Pd(ii). A closer examination of the calculated structures of *cis*-[Pd_2_(**5**)_4_]^4+^ and *cis*-[Pd_2_(**6**)_4_]^4+^ revealed that the steric bulk of the methyl groups also affected the conformation of the ligands and of the cages as a whole (see ESI; Table S3†).^25^ The twist of the alkyne units (*ϕ*, the torsion angle across the alkyne bond; Fig. S137a and b†) was found to be increased between *cis*-[Pd_2_(**5**)_4_]^4+^ and *cis*-[Pd_2_(**6**)_4_]^4+^, and is at least partially responsible for the observed difference in the average ligand twist (*θ*; defined as the torsion angle between the two Pd–N bonds of an individual ligand, Fig. S137c†): ∼27° for *cis*-[Pd_2_(**5**)_4_]^4+^ and ∼41° for *cis*-[Pd_2_(**6**)_4_]^4+^, resulting in a larger helical twist. Surprisingly, the increased helicity of *cis*-[Pd_2_(**6**)_4_]^4+^ compared to *cis*-[Pd_2_(**5**)_4_]^4+^ does not significantly alter the diameter of the largest theoretical sphere able to fit in the cavity of the cage^26^ (Table S3;† this result may be a limitation of using a spherical probe to analyse intrinsic porosity as pore shapes become more anisotropic).

A similar effect on cage conformation could be seen by comparing the calculated structure of *trans*-[Pd_2_(**1**)_4_]^4+^ with previously reported^27^ SCXRD structures of the Pd_2_L_4_ complex of the unsubstituted ligand (1,3-bis(pyridin-3-ylethynyl)benzene, **L**). The latter [Pd_2_(**L**)_4_]^4+^ species adopt a pseudo-*D*_4h_ symmetry with minimal twisting of the alkyne units (*ϕ*_average_ ≈ 9°) and of the ligands (*θ* ≤ 1°) which contrasts with the greater distortion seen in the calculated structure of *trans*-[Pd_2_(**1**)_4_]^4+^ (*ϕ* ≈ 17–30°; *θ* ≈ 42°) (Table S3†). Again, no significant reduction in the calculated cavity size of *trans*-[Pd_2_(**1**)_4_]^4+^ was observed.

These structural distortions induced by the steric encumbrance of the methyl groups suggests that relatively minor modifications to the ligand framework could be used to dramatically alter the assembly conformation, allowing precision engineering of the internal cavity space without any significant loss in intrinsic porosity, a concept we are currently exploring.

## Conclusions

Metallo-supramolecular systems have evolved in complexity since their initial realisation; heteroleptic, functionalised and even interlocked assemblies^28^ have been reported. Despite the significant advances made in the chemists' toolbox of techniques for designing these systems, symmetrical ligands still tend to be employed to simplify the self-assembly process. In this work we have shown that it is possible, through careful ligand design, to exploit steric or geometric factors, or a combination of the two, to assemble unsymmetrical ditopic ligands with “naked” palladium(ii) ions, forming single Pd_2_L_4_ cage isomers with high fidelity. This approach does not require the incorporation of ligand motifs of different denticity, nor the use of substantially bulky moieties, both of which can introduce exorbitant complexity into the ligand framework and make them synthetically very taxing, inhibiting general utility.

Structure calculations have been invaluable in helping to explain the outcomes of the self-assembly processes examined. Following on from this work we expect to be able to exploit these as a predictive tool to forecast successful outcomes, defined as the self-assembly of a single cage isomer that is sufficiently lower in energy than other possible structural isomers as to be the only spectroscopically detectable product.

Ongoing work in our lab will also look to utilise the self-assembly principles delineated through this work to prepare multi-functional systems in which moieties are held in specific arrangements relative to each other. It is hoped that in this manner functionalities within the cage cavities may be spatially segregated, a concept we envisage exploiting for a variety of applications.

## Conflicts of interest

There are no conflicts to declare.

## Supplementary Material

SC-011-C9SC05534G-s001

SC-011-C9SC05534G-s002
