# Supplementary material for: Conformational control of Pd2L4 assemblies with unsymmetrical ligands
Source: Chem Sci. 2019 Nov 28;11(3):677–83. doi: 10.1039/c9sc05534g (PMC8146399; doi:10.1039/c9sc05534g)
Supplement: SC-011-C9SC05534G-s001 [file SC-011-C9SC05534G-s001.pdf]

Electronic Supplementary Material (ESI) for Chemical Science.
This journal is © The Royal Society of Chemistry 2019

Electronic Supporting Information

Conformational Control of Pd_2L_4 Assemblies with Unsymmetrical Ligands

James E. M. Lewis,* Andrew Tarzia, Andrew J. P. White and Kim E. Jelfs

Department of Chemistry
Imperial College London
Molecular Sciences Research Hub
80 Wood Lane
London W12 0BZ, UK

*james.lewis@imperial.ac.uk

Contents

General Experimental 3
Synthetic Procedures 4
S1 4
S2 8
S3 10
S4 13
S5 16
S6 19
S7 22
S8 25
S9 28
1 31
2 34
3 37
4 40
5 43
6 46
$[Pd_2(\mathbf{1})_4](BF_4)_4$ 49
$[Pd_2(\mathbf{2})_4](BF_4)_4$ 59
$[Pd_2(\mathbf{3})_4](BF_4)_4$ 63
$[Pd_2(\mathbf{4})_4](BF_4)_4$ 68
$[Pd_2(\mathbf{5})_4](BF_4)_4$ 73
$[Pd_2(\mathbf{6})_4](BF_4)_4$ 77
X-ray Crystallographic Data 83
X-ray crystal structure of *cis*-$[Pd_2(\mathbf{2})_4](BF_4)_4$ 83
X-ray crystal structure of *cis*-$[Pd_2(\mathbf{4})_4](BF_4)_4$ 84
Density Functional Theory Calculations 85
Ligand Distortion Measurements 87
References 88

General Experimental

Synthesis: Unless otherwise stated, all reagents, including anhydrous solvents, were purchased from commercial sources and used without further purification. $CDCl_3$ was stored over 4 Å molecular sieves prior to use. All reactions were carried out under an atmosphere of N_2 using degassed, anhydrous solvents unless otherwise stated. Petrol refers to the fraction of petroleum ether boiling in the range 40-60 °C. Analytical TLC was performed on pre-coated silica gel plates (0.25 mm thick, 60F254, Merck, Germany) and observed under UV light. EDTA solution refers to a 0.1 M solution of EDTA-Na_2 in 3% $NH_{3(aq)}$.

Analysis: NMR spectra were recorded on Bruker AV400 or AV500 instrument, at a constant temperature of 298 K. Chemical shifts are reported in parts per million from low to high field and referenced to residual solvent. Standard abbreviations indicating multiplicity were used as follows: m = multiplet, quint = quintet, q = quartet, t = triplet, d = doublet, s = singlet, app. = apparent, br. = broad. Signal assignment was carried out using 2D NMR methods (HSQC, HMBC, COSY, NOESY) where necessary. In the case of some signals absolute assignment was not possible. Here indicative either/or assignments (e.g. H_A/H_B for H_A or H_B) are provided. All melting points were determined using a hot stage apparatus and are uncorrected. Mass spectrometry was carried out by the Imperial College London, Department of Chemistry Mass Spectroscopy Service using Waters LCT Premier for HR-ESI-MS and Thermo Scientific Q-Exactive for tandem MS.

The following compounds were synthesised according to literature procedures: 5-Iodoisoquinoline.[1]

Synthetic Procedures

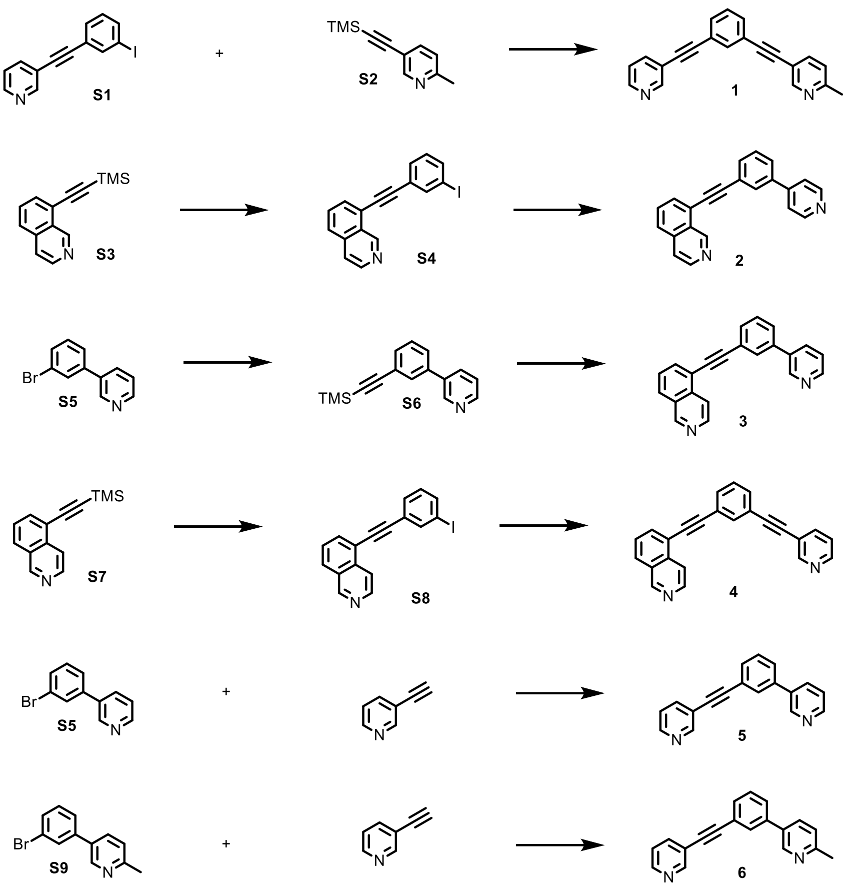

S1

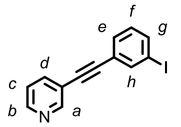

3-Ethynylpyridine (0.209 g, 3.00 mmol, 1 eq.), 1,3-diiodobenzene (1.48 g, 4.50 mmol, 1.5 eq.), $Pd(PPh_3)_2Cl_2$ (0.042 g, 0.060 mmol, 2 mol%) and CuI (0.023 g, 0.12 mmol, 4 mol%) were stirred at rt in $^{i}Pr_2NH$ (15 mL) for 16 h. EDTA solution (25 mL) was added and the aqueous phase extracted with CH_2Cl_2 (3 × 20mL). The combined organic phases were dried ($MgSO_4$) and the solvent removed *in vacuo*. After purification by column chromatography on silica (1:4 EtOAc/petrol) the product was obtained as a yellow oil (0.657 g, 72%) that solidified on standing. M.p. 75-77 °C. ^{1}H NMR (400 MHz, $CDCl_3$) δ: 8.75 (dd, *J* = 2.1, 0.8 Hz, 1H, H_a), 8.56 (dd, *J* = 4.9, 1.7 Hz, 1H, H_b), 7.91 (app. t, *J* = 1.6 Hz, 1H, H_h), 7.79 (app. dt, *J* = 7.9, 1.9 Hz, 1H, H_d), 7.70 (ddd, *J* = 8.0, 1.8, 1.1 Hz, 1H, H_g), 7.50 (ddd, *J* = 7.7, 1.5, 1.0 Hz, 1H, H_e), 7.29 (ddd, *J* = 7.9, 4.9, 0.9 Hz, 1H, H_c), 7.10 (app. t, *J* = 7.9 Hz, 1H, H_f). ^{13}C NMR (101 MHz, $CDCl_3$) δ: 152.4, 149.0, 140.4, 138.6, 137.9, 130.9, 130.1, 124.7, 123.2, 120.1, 93.9, 91.0, 87.3. HR-ESI-MS *m/z* = 305.9777 $[M+H]^+$ calc. 305.9780.

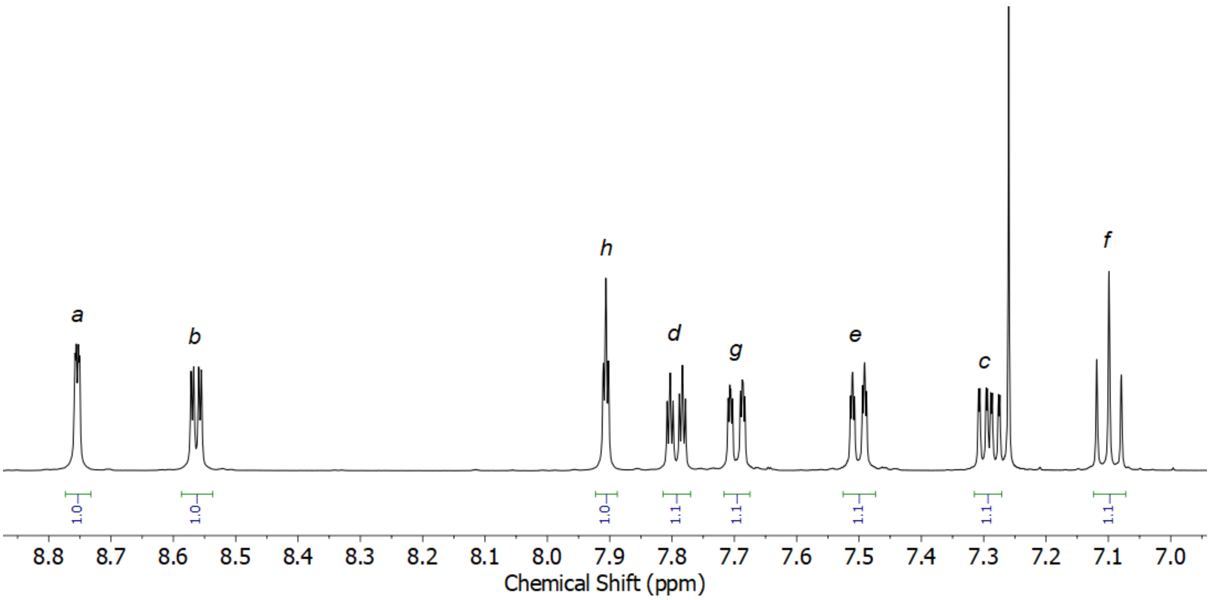

Figure S1 ^{1}H NMR ($CDCl_3$, 400 MHz) of **S1**.

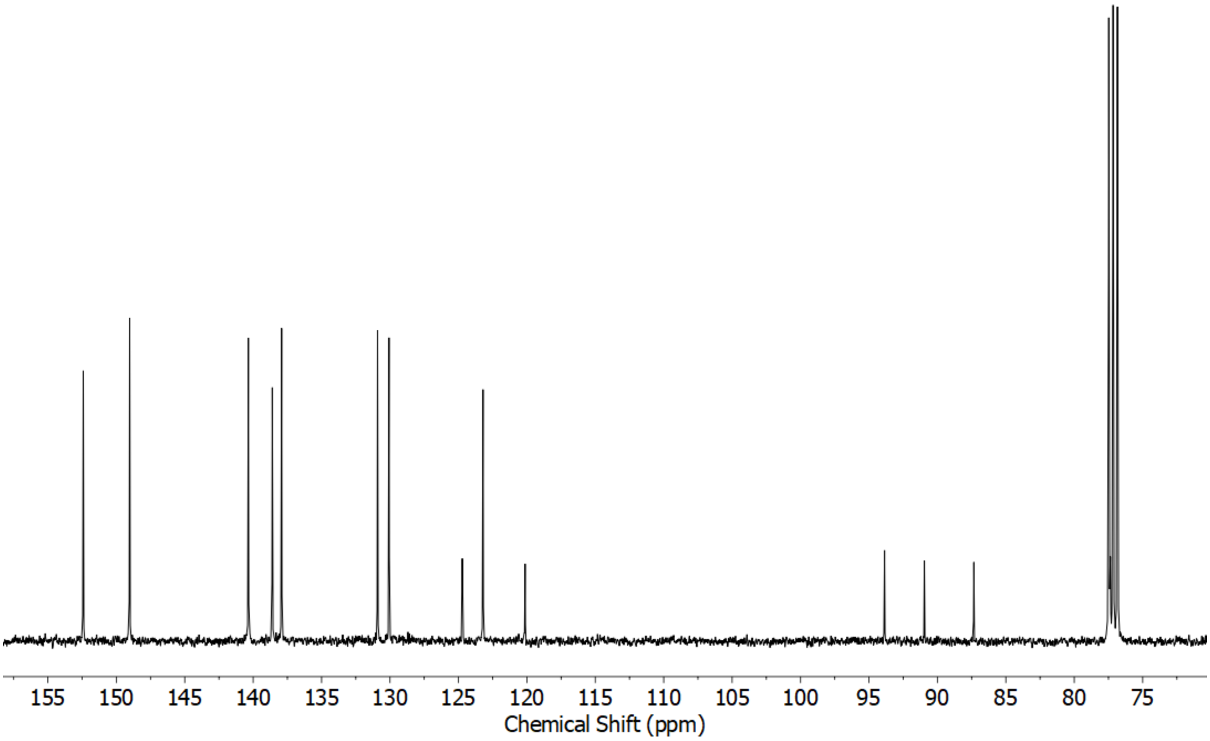

Figure S2 ^{13}C NMR ($CDCl_3$, 101 MHz) of **S1**.

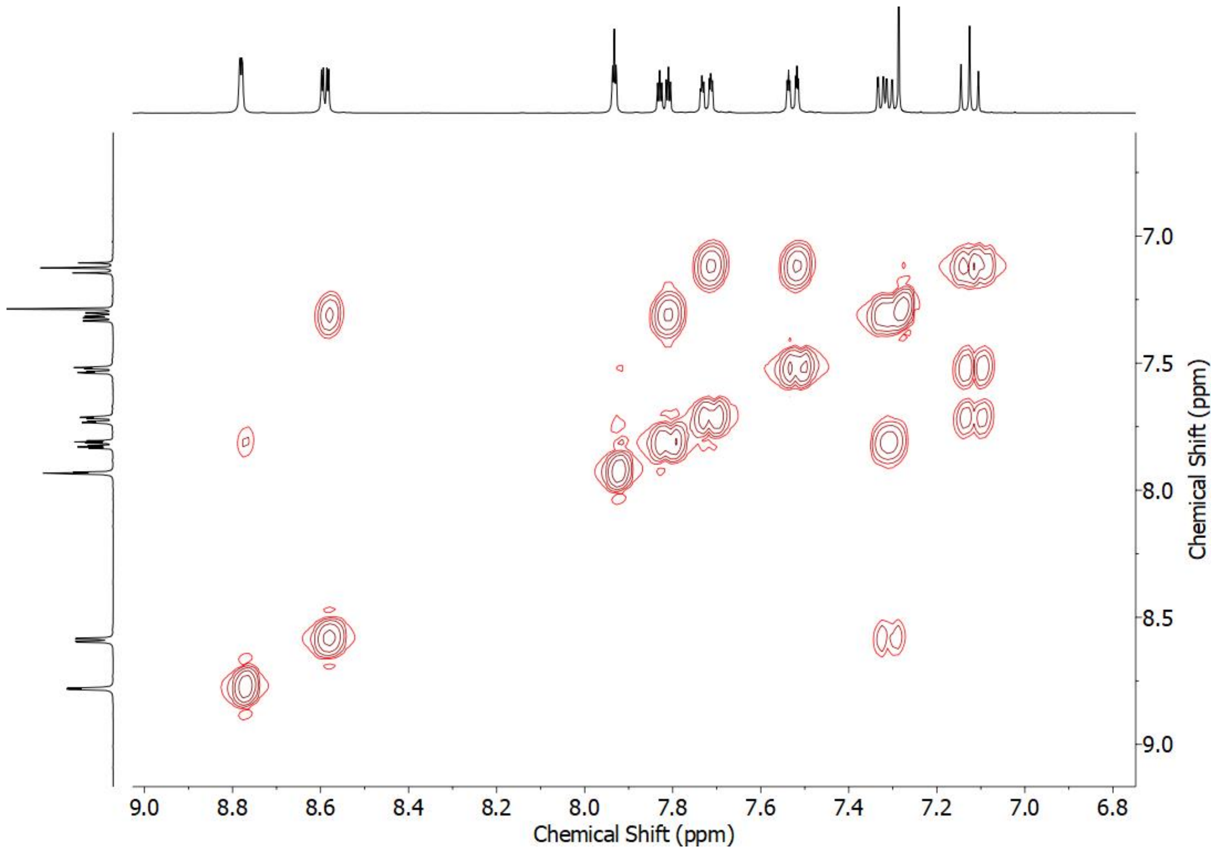

Figure S3 COSY NMR ($CDCl_3$) of **S1**.

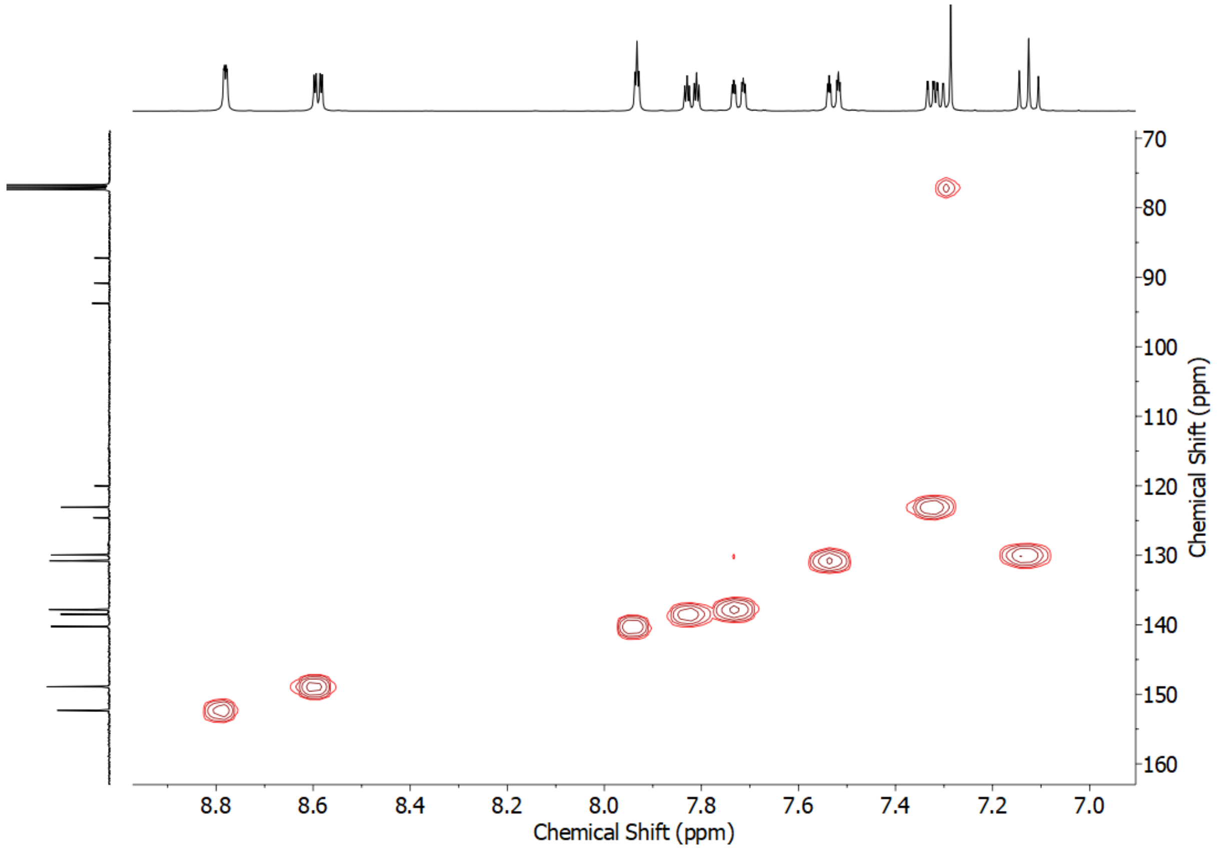

Figure S4 HSQC NMR ($CDCl_3$) of **S1**.

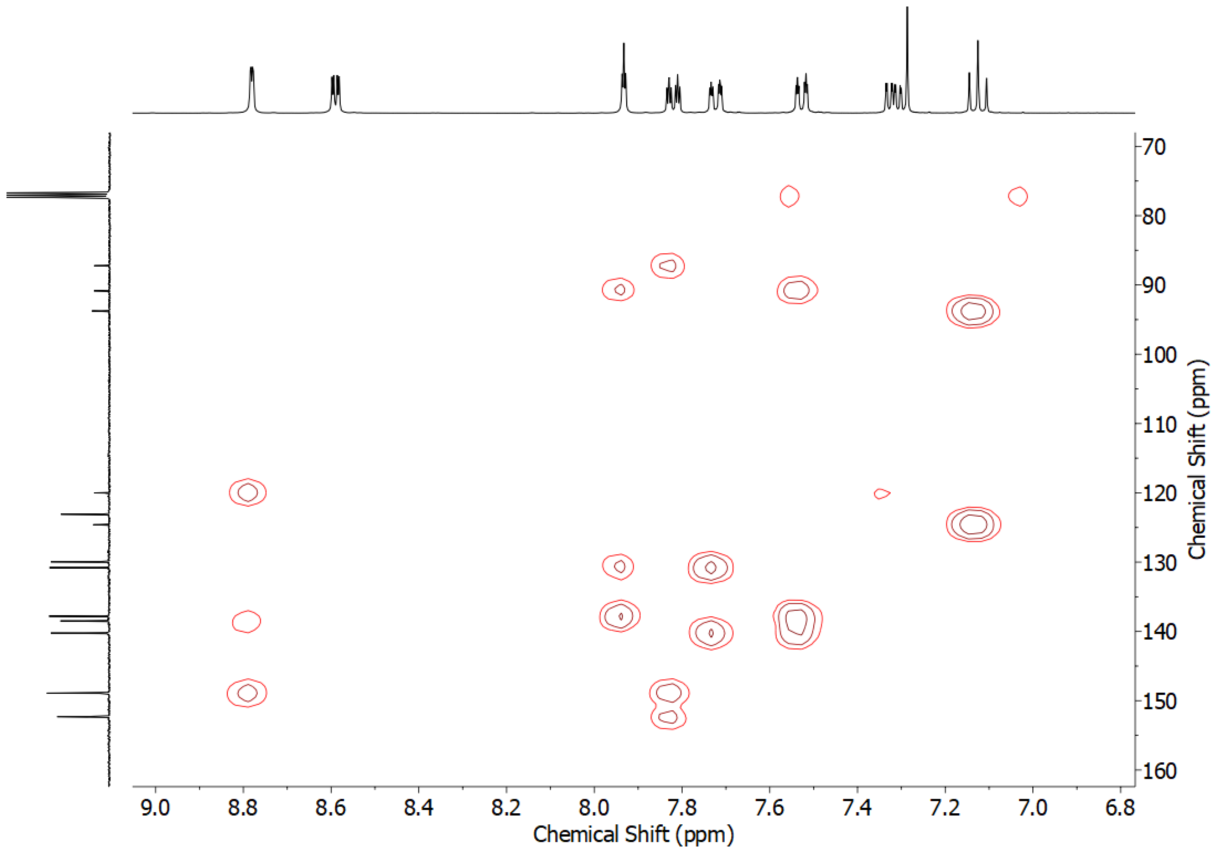

Figure S5 HMBC NMR ($CDCl_3$) of **S1**.

S2

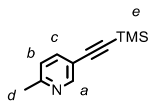

5-Bromo-2-methylpyridine (0.344 g, 2.0 mmol, 1 eq.), trimethylsilylacetylene (0.42 mL, 3.0 mmol, 1.5 eq.), $Pd(PPh_3)_2Cl_2$ (0.070 g, 010 mmol, 5 mol%) and CuI ((0.038 g, 0.20 mmol, 10 mol%) were stirred at 80 °C in ^{i}Pr$_2$NH (10 mL) in a sealed vial for 21 h. EDTA solution (20 mL) was added and the aqueous phase extracted with CH_2Cl_2 (3 × 20 mL). The combined organic phases were dried ($MgSO_4$) and the solvent removed *in vacuo*. After purification by column chromatography on silica (1:19 EtOAc/petrol) the product was obtained as a brown oil (0.350 g, 92%). ^{1}H NMR (400 MHz, $CDCl_3$) δ: 8.57 (dd, *J* = 2.2, 0.9 Hz, 1H, H$_a$), 7.62 (dd, *J* = 8.1, 2.2 Hz, 1H, H$_c$), 7.09 (d, *J* = 8.0 Hz, 1H, H$_b$), 2.55 (s, 3H, H$_d$), 0.25 (s, 9H, H$_e$). ^{13}C NMR (101 MHz, $CDCl_3$) δ: 158.1, 152.1, 139.2, 122.7, 117.3, 101.9, 97.3, 24.7, 0.0. HR-ESI-MS *m/z* = 190.1063 [M+H]$^+$ calc. 190.1052.

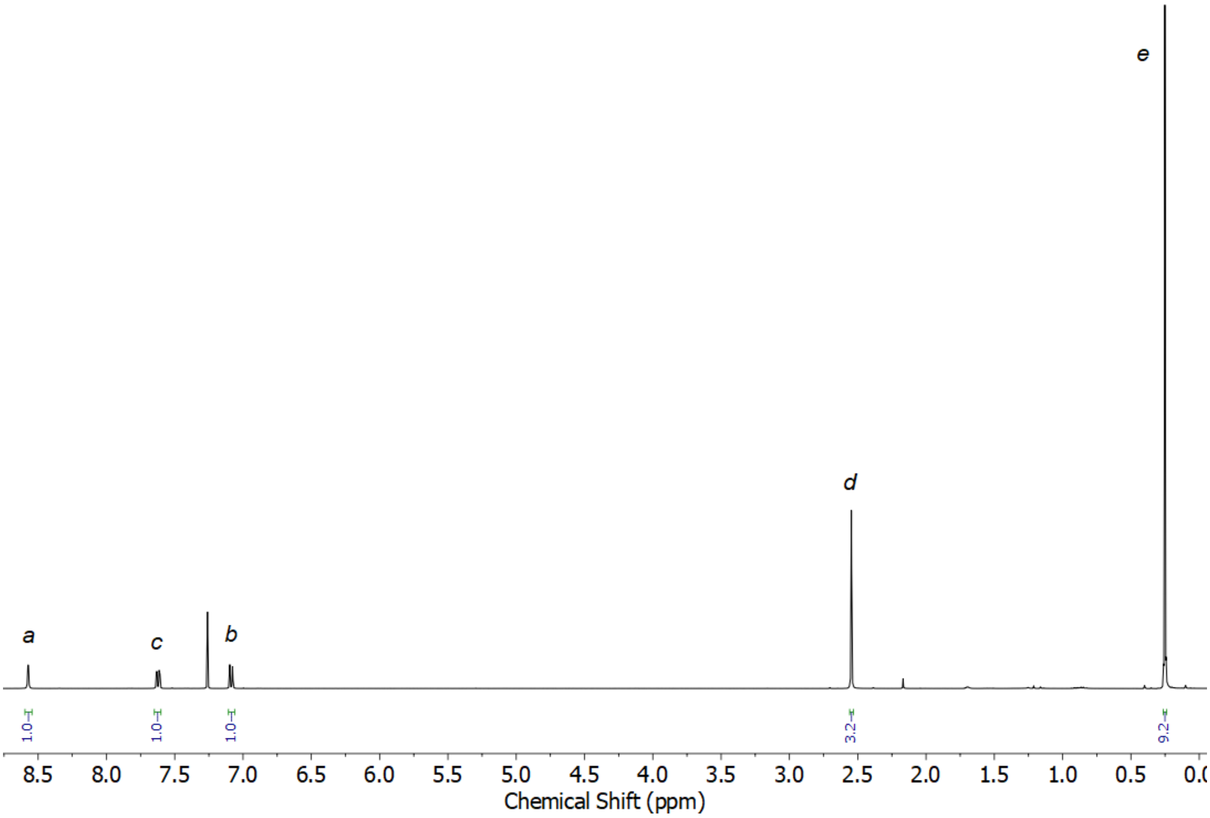

Figure S6 ^{1}H NMR ($CDCl_3$, 400 MHz) of **S2**.

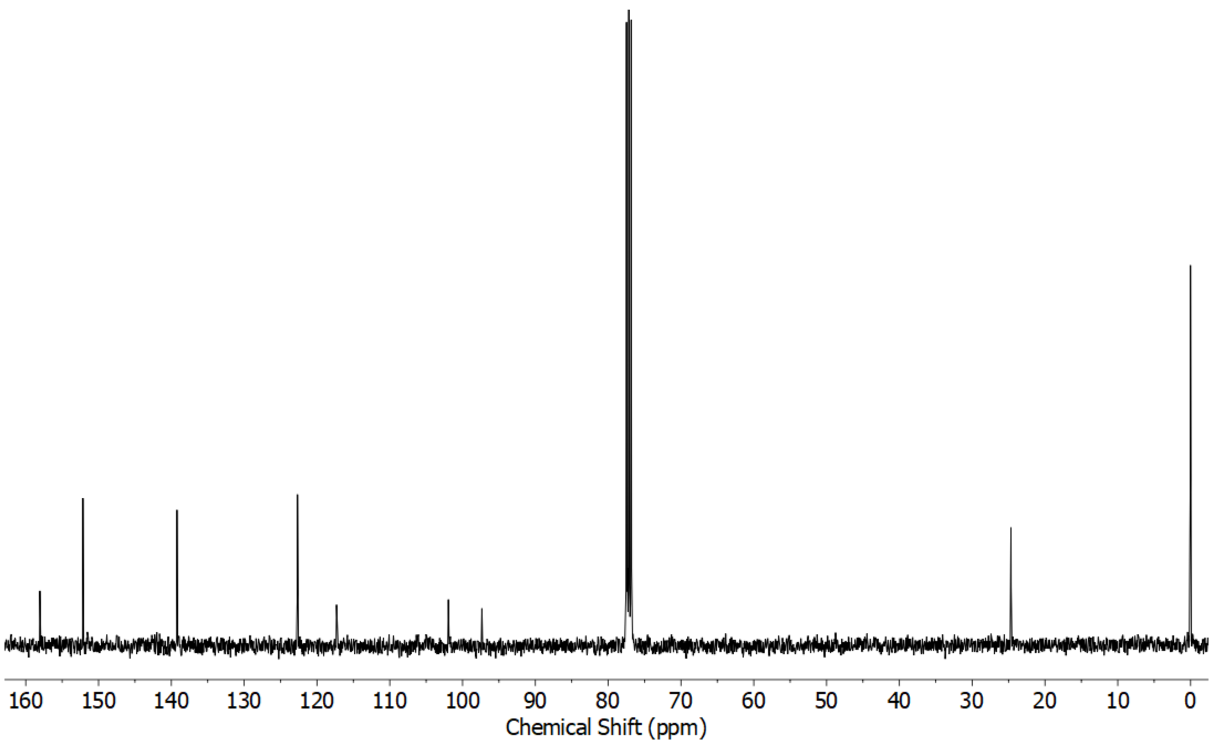

Figure S7 ^{13}C NMR ($CDCl_3$, 101 MHz) of **S2**.

S3

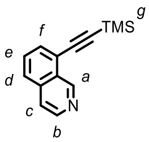

8-Bromoisoquinoline (0.624 g, 3.00 mmol, 1 eq.), trimethylsilylacetylene (0.50 mL, 3.6 mmol, 1.2 eq.), $Pd(PPh_3)_2Cl_2$ (0.053 g, 0.075 mmol, 2.5 mol%) and CuI (0.029 g, 0.15 mmol, 5 mol%) were stirred at 75 °C in $^{i}Pr_2NH$ (15 mL) in a sealed vial for 18 h. EDTA solution (25 mL) was added and the aqueous phase extracted with CH_2Cl_2 (3 × 25 mL). The combined organic phases were dried ($MgSO_4$) and the solvent removed *in vacuo*. After purification by column chromatography on silica (1:9 EtOAc/pentane) the product was obtained as a yellow oil (0.601 g, 89%). ^{1}H NMR (400 MHz, $CDCl_3$) δ: 9.72 (s, 1H, H_a), 8.58 (d, *J* = 5.7 Hz, 1H, H_b), 7.79-7.74 (m, 2H, H_d, H_f), 7.64-7.59 (m, 2H, H_c, H_e), 0.34 (s, 9H, H_g). ^{13}C NMR (101 MHz, $CDCl_3$) δ: 151.5, 143.9, 135.8, 131.9, 129.8, 128.3, 127.3, 121.6, 120.5, 101.7, 101.0, 0.1. HR-ESI-MS *m/z* = 226.1047 $[M+H]^+$ calc. 226.1052.

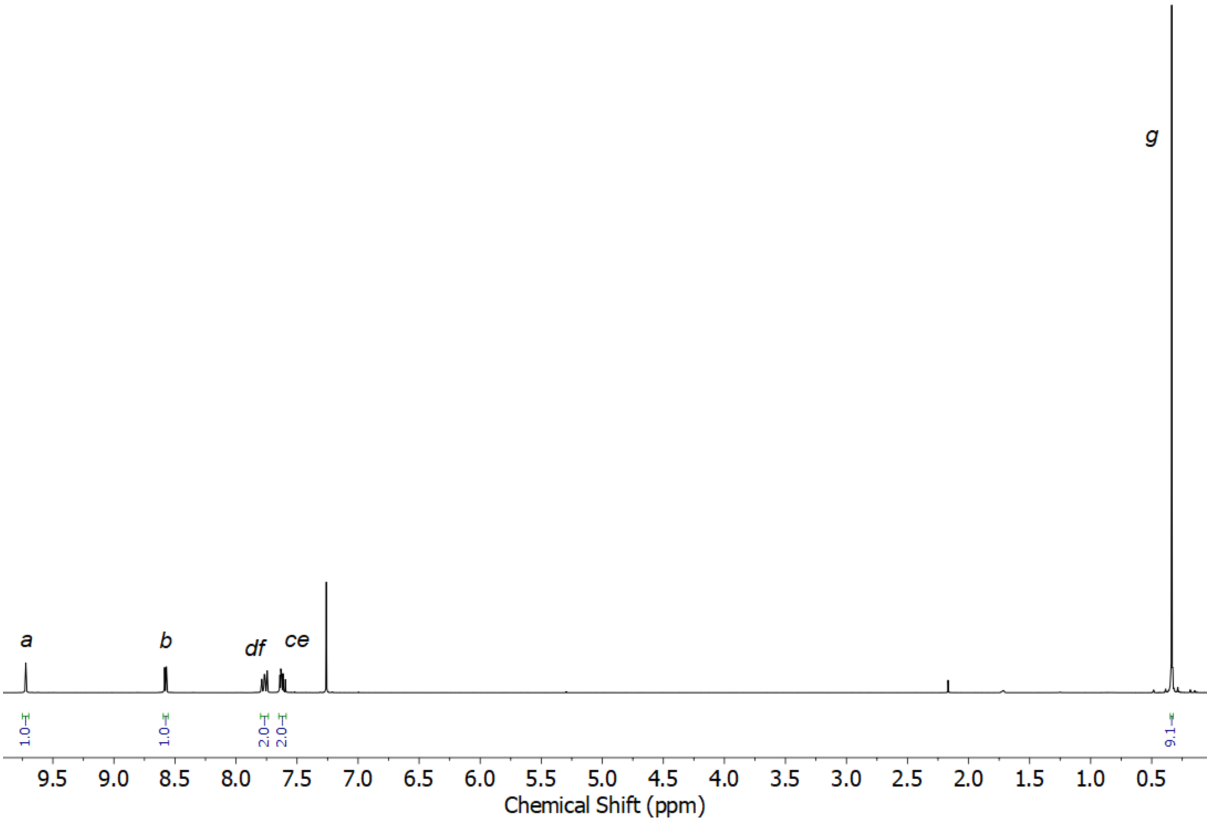

Figure S8 ^{1}H NMR ($CDCl_3$, 400 MHz) of **S3**.

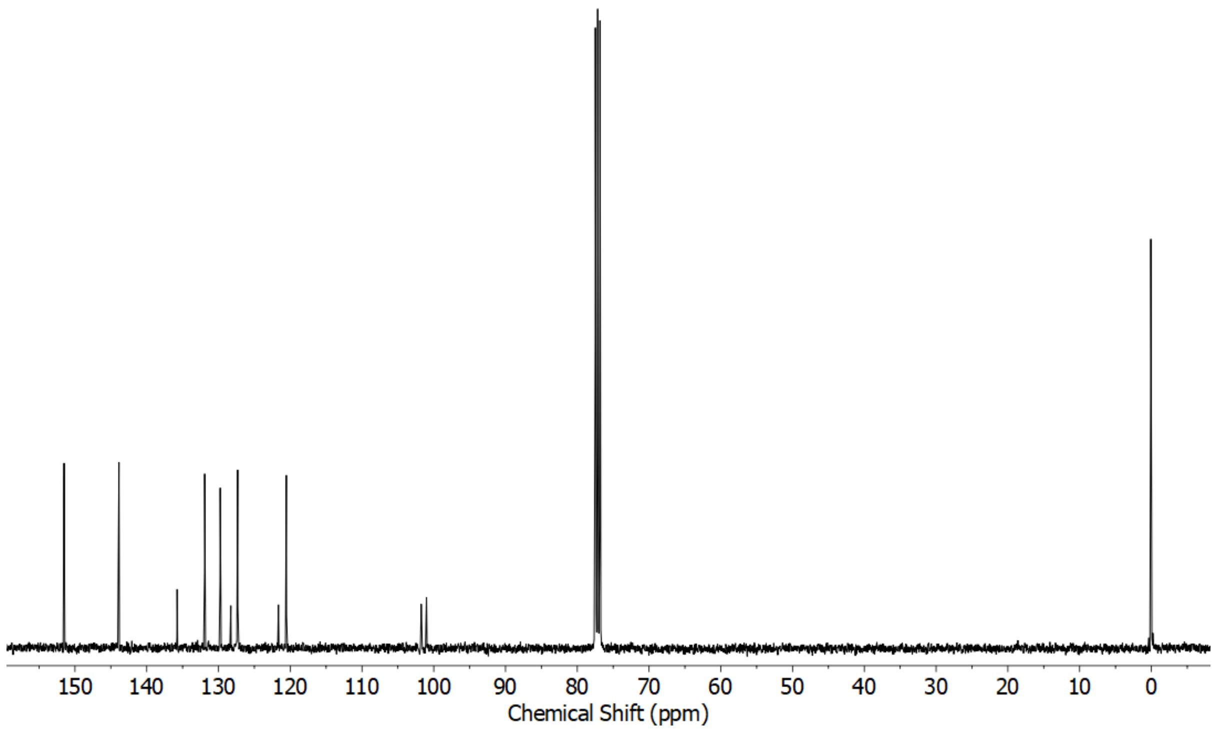

Figure S9 ^{13}C NMR ($CDCl_3$, 101 MHz) of **S3**.

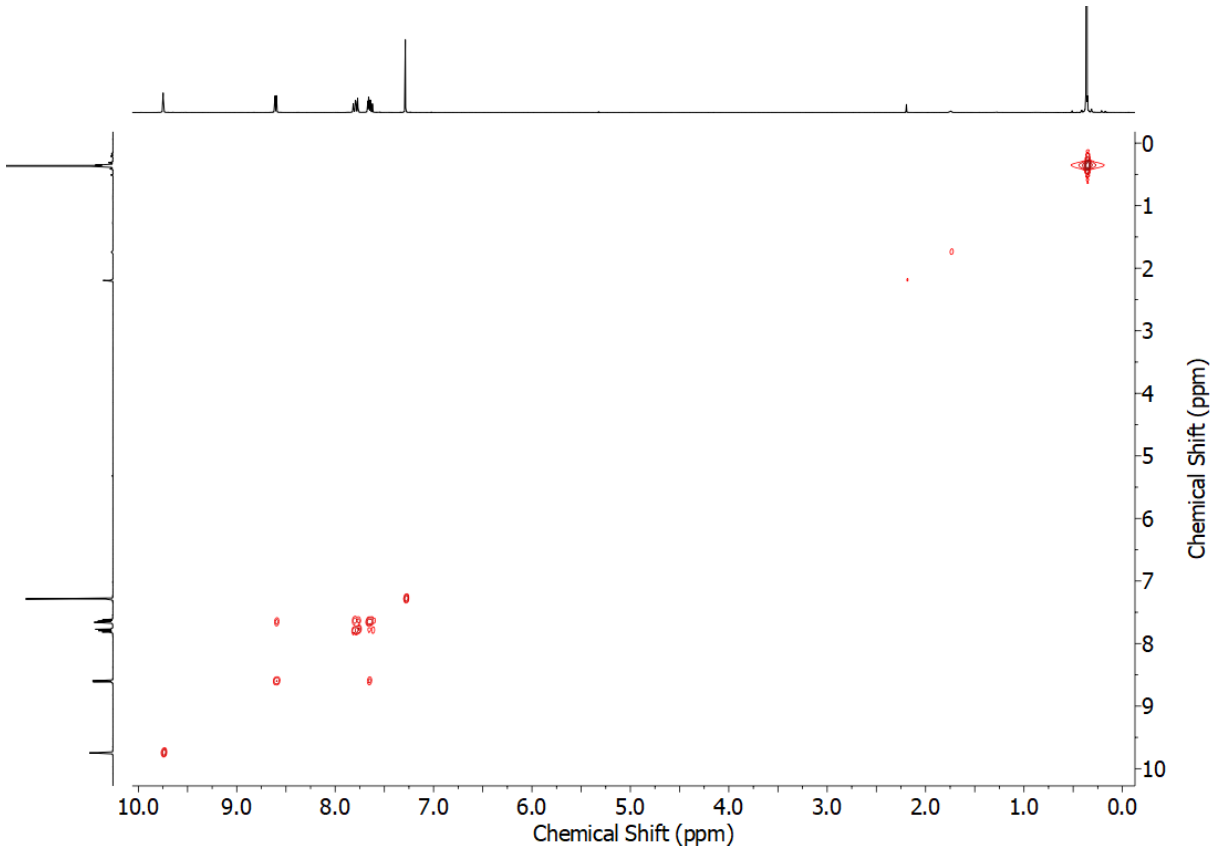

Figure S10 COSY NMR ($CDCl_3$) of **S3**.

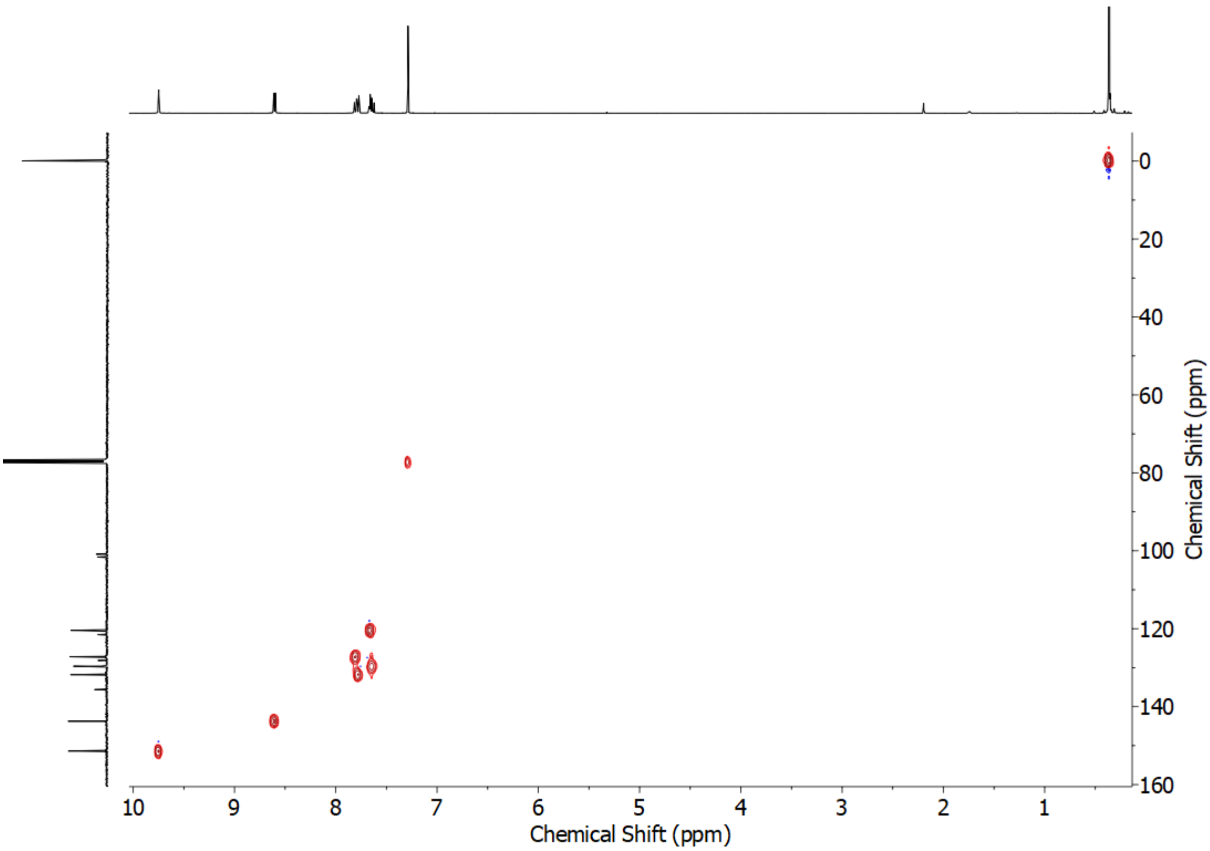

Figure S11 HSQC NMR ($CDCl_3$) of **S3**.

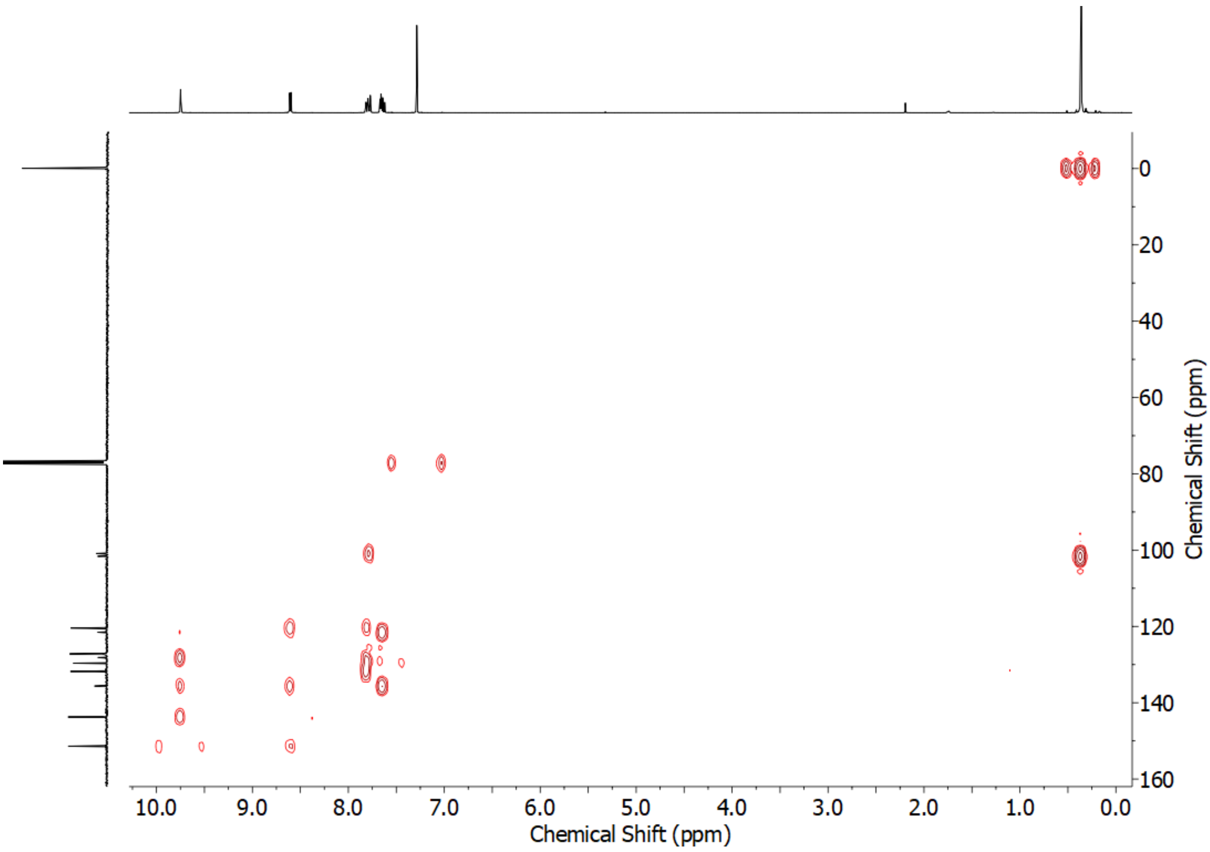

Figure S12 HMBC NMR ($CDCl_3$) of **S3**.

S4

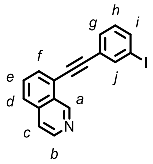

To **S3** (0.225 g, 1.0 mmol, 1 eq.), 1,3-diiodobenzene (0.495 g, 1.5 mmol, 1.5 eq.), $Pd(PPh_3)_2Cl_2$ (0.018 g, 0.025 mmol, 2.5 mol%) and CuI (0.010 g, 0.050 mmol, 5 mol%) in CH_3CN (5 mL) was added DBU (2.7 mL, 18 mmol, 18 eq.) and the reaction stirred at rt for 18 h. EDTA solution (25 mL) was added, and the aqueous extracted with CH_2Cl_2 (3 × 20 mL). The combined organic phases were dried ($MgSO_4$) and the solvent removed *in vacuo*. After purification by column chromatography on silica (1:9 EtOAc/petrol followed by 1:3 EtOAc/petrol) the product was obtained as a yellow oil that solidified on standing (0.243 g, 68%). M.p. 91-93 °C. ^{1}H NMR (400 MHz, $CDCl_3$) δ: 9.78 (s, 1H, H_a), 8.61 (d, *J* = 5.7 Hz, 1H, H_b), 8.01 (app. t, *J* = 1.6 Hz, 1H, H_j), 7.83-7.80 (m, 2H, 2 of H_d, H_e, H_f), 7.73 (ddd, *J* = 8.0, 1.8, 1.1 Hz, 1H, H_g/H_i), 7.69-7.65 (m, 2H, H_c, 1 of H_d, H_e, H_f), 7.61 (app. dt, *J* = 7.7, 1.3 Hz, 1H, H_g/H_i), 7.14 (app. t, *J* = 7.8 Hz, 1H, H_h). ^{13}C NMR (101 MHz, $CDCl_3$) δ: 151.3, 144.0, 140.4, 138.0, 135.9, 131.7, 131.0, 130.2, 129.9, 128.0, 127.5, 124.9, 121.3, 120.6, 94.1, 94.0, 87.0. HR-ESI-MS *m/z* = 355.9941 $[M+H]^+$ calc. 355.9936.

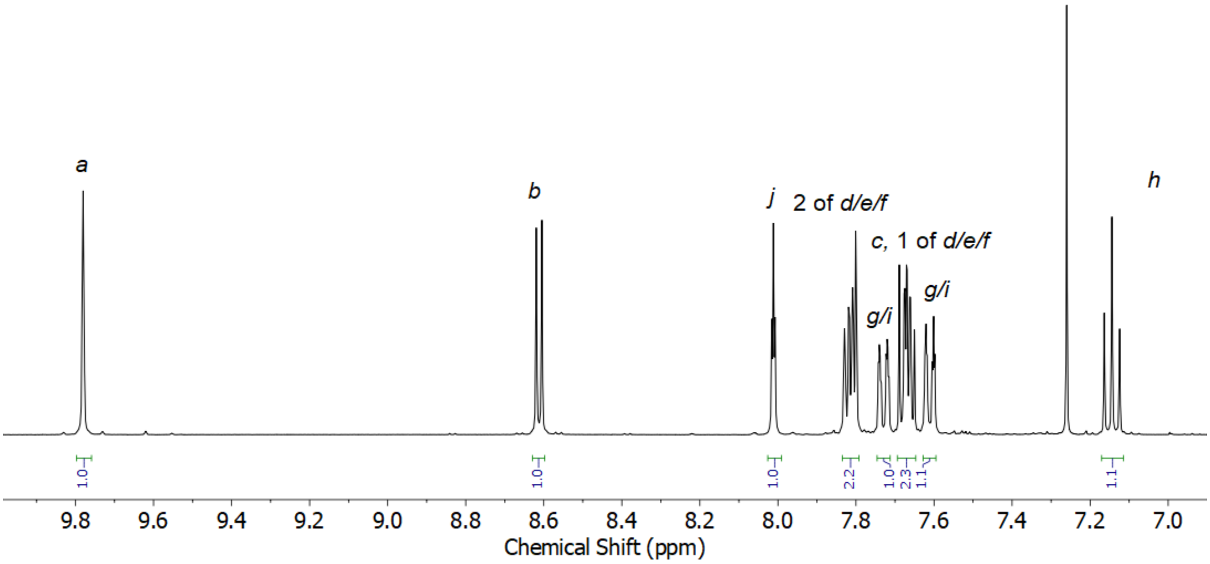

Figure S13 ^{1}H NMR ($CDCl_3$, 400 MHz) of **S4**.

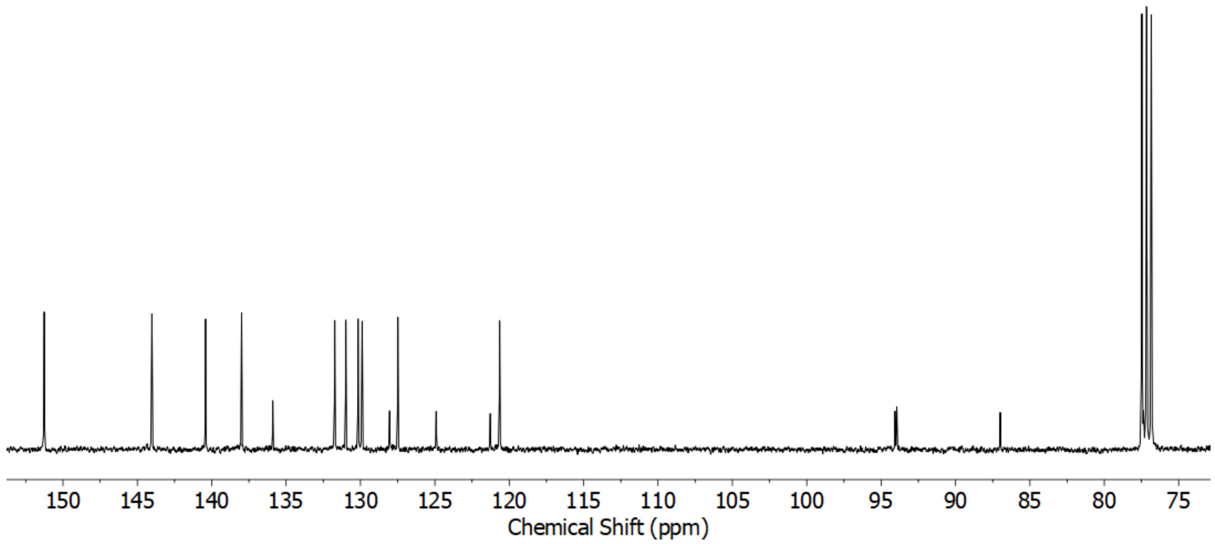

Figure S14 ^{13}C NMR ($CDCl_3$, 101 MHz) of **S4**.

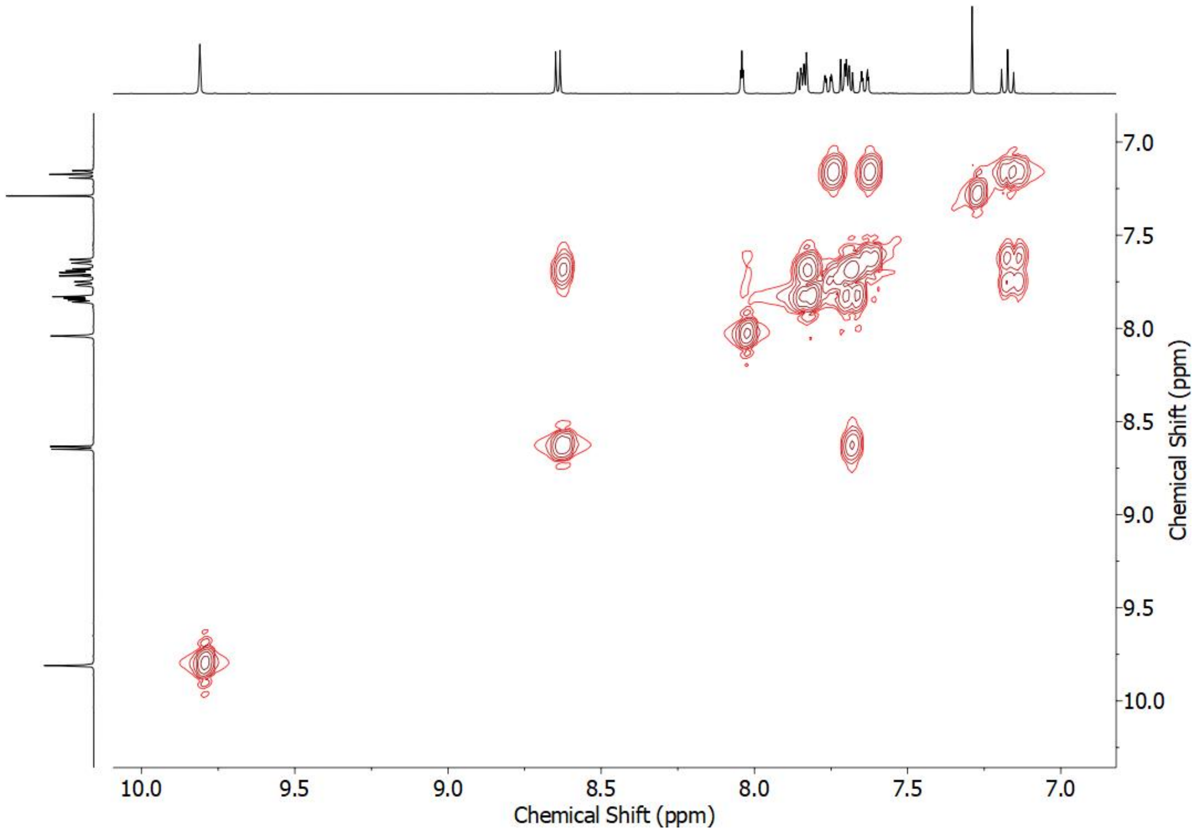

Figure S15 COSY NMR ($CDCl_3$) of **S4**.

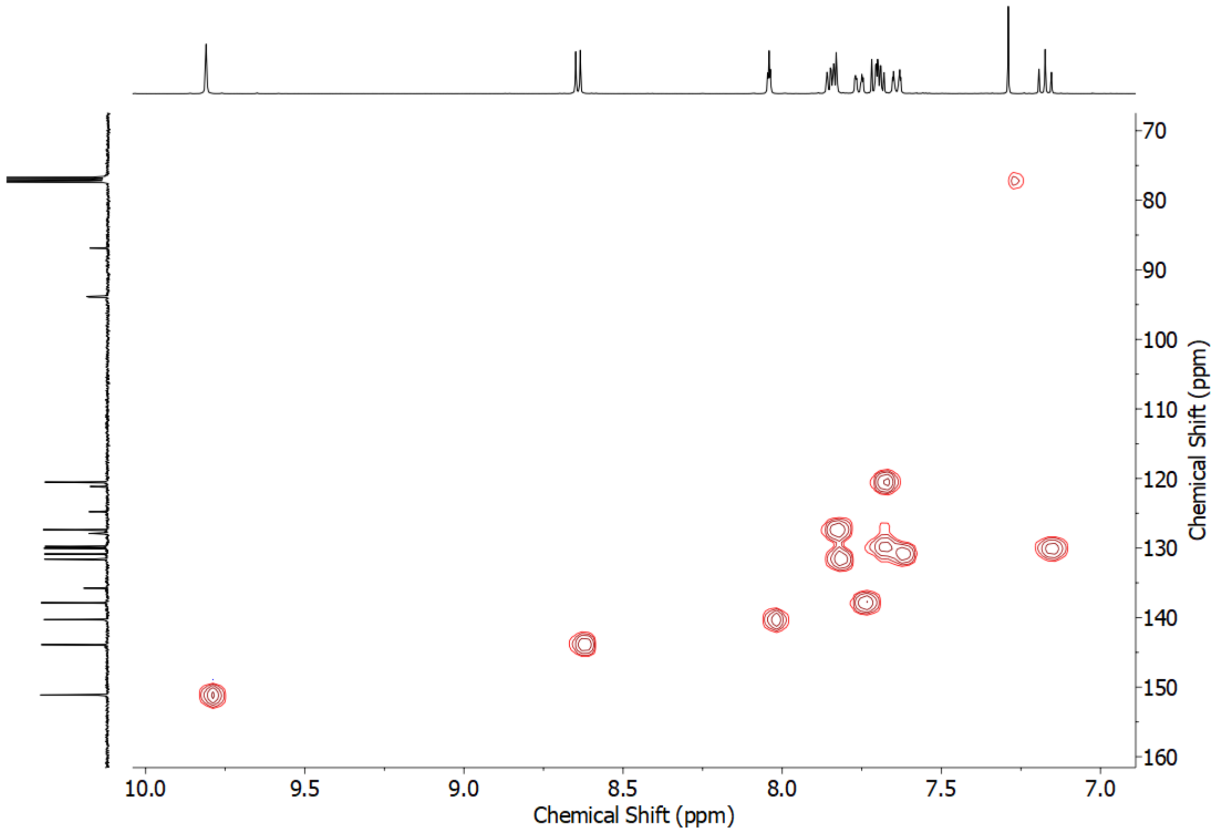

Figure S16 HSQC NMR ($CDCl_3$) of **S4**.

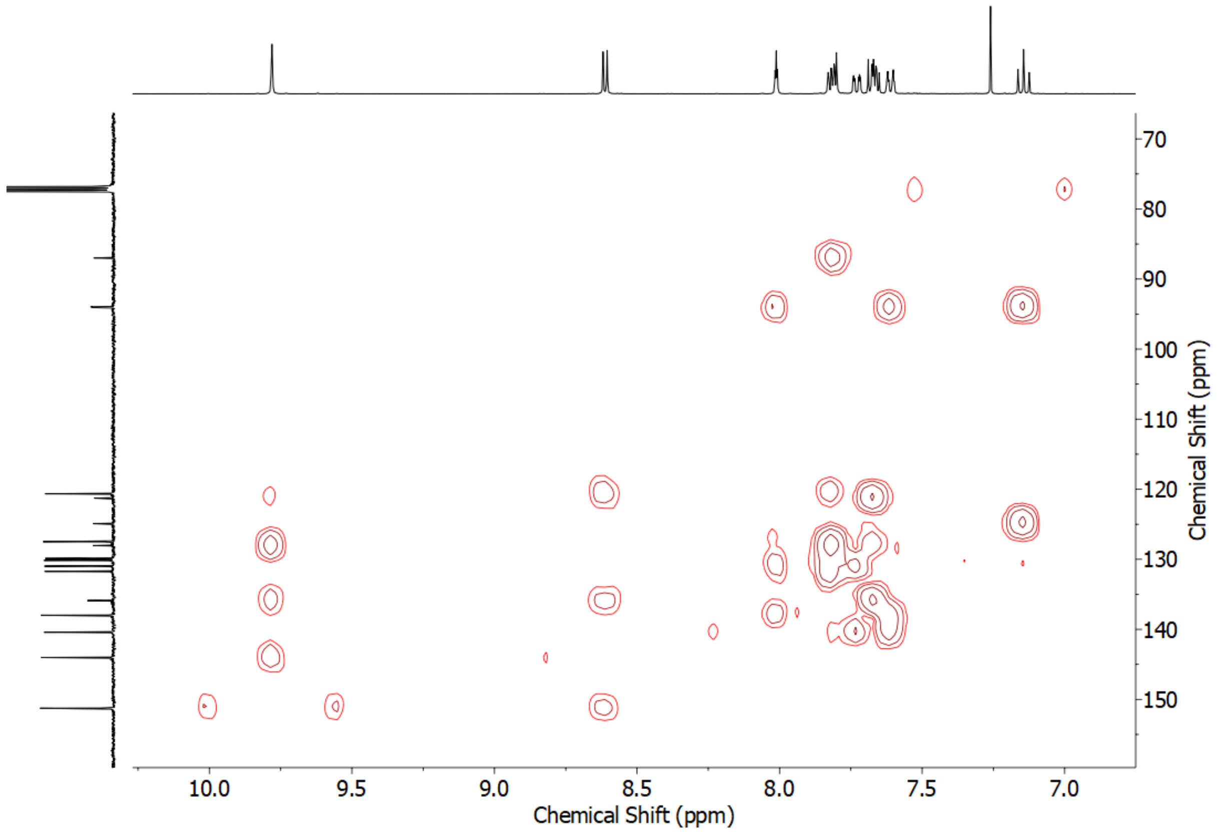

Figure S17 HMBC NMR ($CDCl_3$) of **S4**.

S5

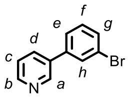

To 3-pyridinylboronic acid (0.369 g, 3.0 mmol, 1 eq.), 1,3-dibromobenzene (0.708 g, 3.0 mmol, 1 eq.) and $Pd(PPh_3)_2Cl_2$ (0.105 g, 0.15 mmol, 5 mol%) in dioxane (10 mL) was added a solution of K_2CO_3 (1.04 g, 7.5 mmol, 2.5 eq.) in H_2O (5 mL) whilst stirring vigorously. The reaction mixture was stirred at 100 °C for 15 h before EDTA solution (25 mL) was added. The aqueous phase was extracted with CH_2Cl_2 (3 × 25 mL), the combined organic phases dried ($MgSO_4$) and the solvent removed *in vacuo*. After purification by column chromatography on silica (1:9 acetone/CH_2Cl_2) the product was obtained as a yellow oil (0.277 g, 39%). ^{1}H NMR (400 MHz, $CDCl_3$) δ: 8.82 (d, *J* = 2.3 Hz, 1H, H_a), 8.62 (dd, *J* = 4.8, 1.5 Hz, 1H, H_b), 7.85 (app. dt, *J* = 7.9, 1.7 Hz, 1H, H_d), 7.73 (app. t, *J* = 1.7 Hz, 1H, H_h), 7.55-7.50 (m, 2H, 2 of H_e, H_f, H_g), 7.39-7.34 (m, 2H, H_c, H_e/H_f/H_g). ^{13}C NMR (101 MHz, $CDCl_3$) δ: δ 149.2, 148.4, 140.1, 135.4, 134.5, 131.2, 130.7, 130.3, 125.9, 123.7, 123.3. HR-ESI-MS *m/z* = 233.9926 $[M+H]^+$ calc. 233.9918.

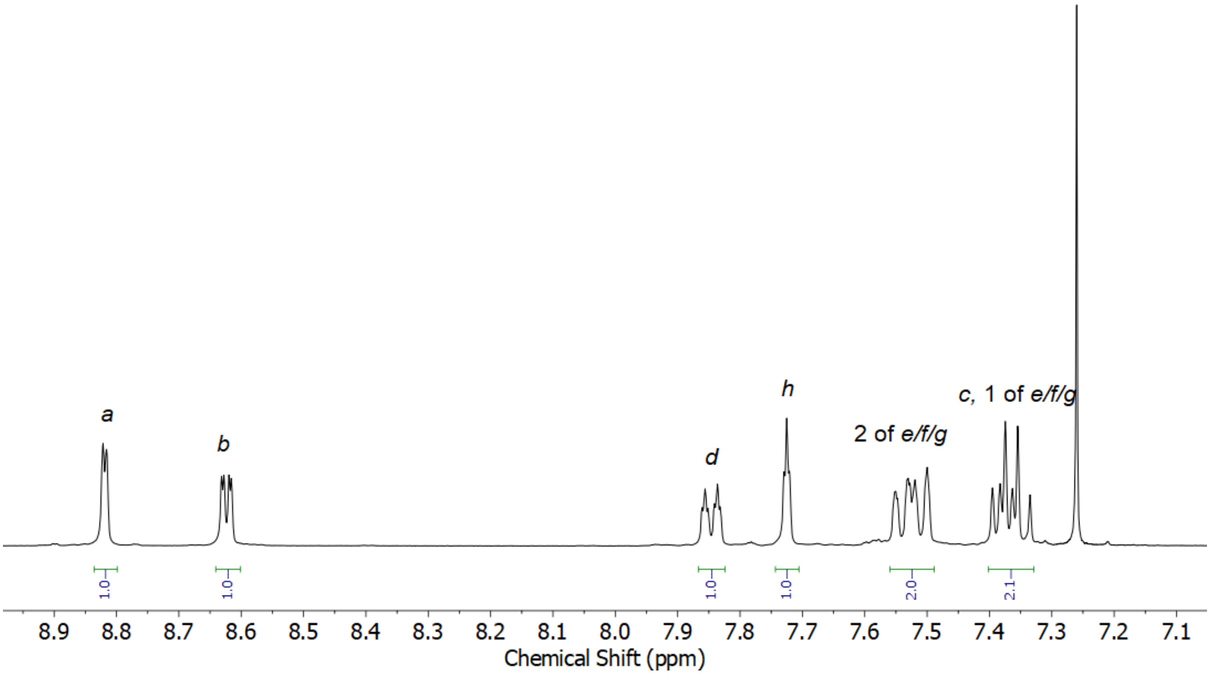

Figure S18 ^{1}H NMR ($CDCl_3$, 400 MHz) of **S5**.

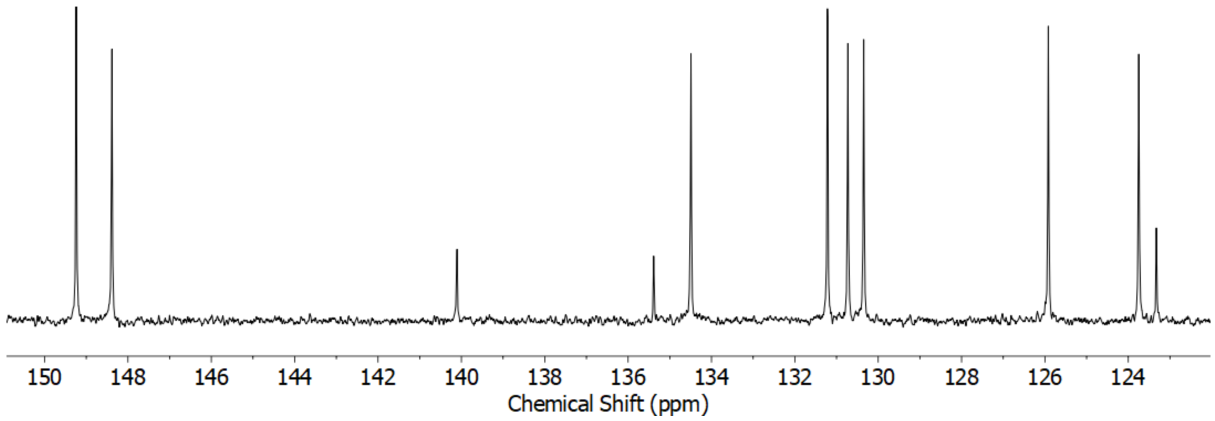

Figure S19 ^{13}C NMR ($CDCl_3$, 101 MHz) of **S5**.

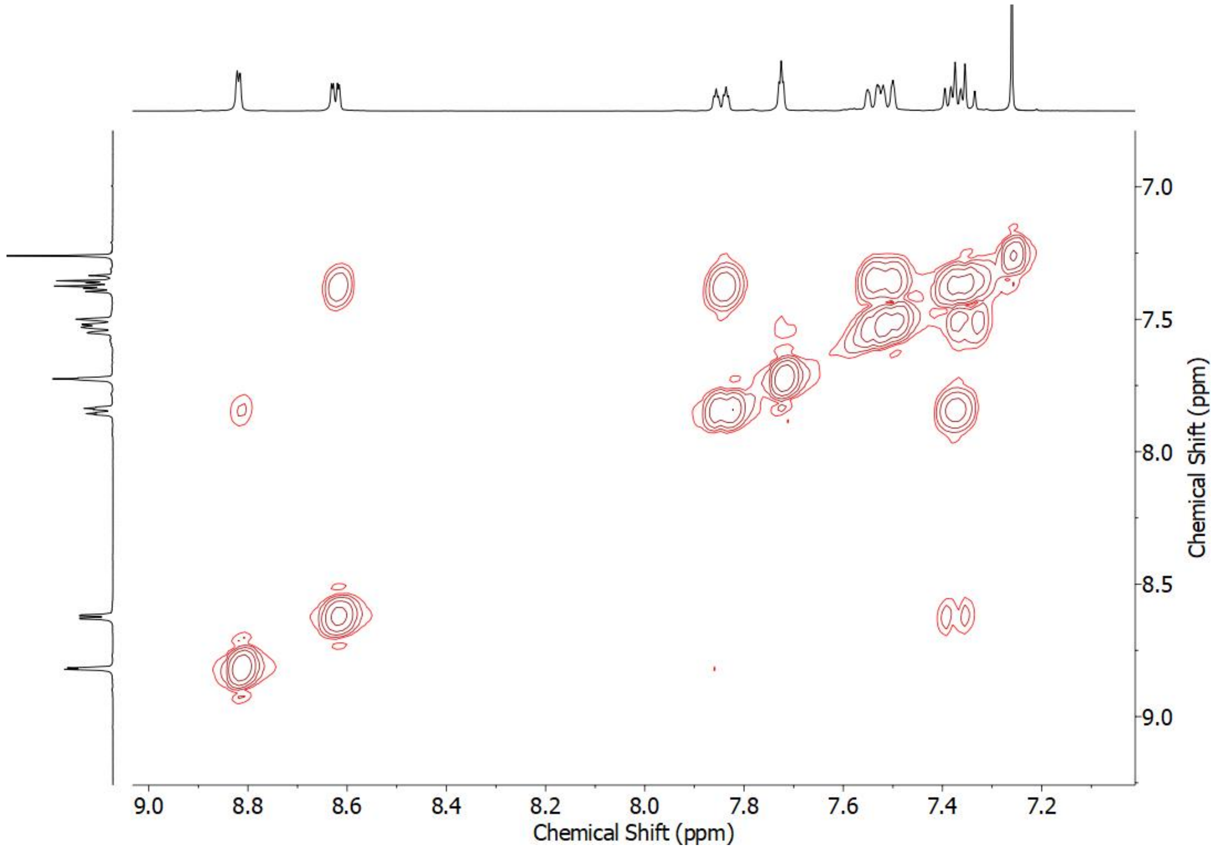

Figure S20 COSY NMR ($CDCl_3$) of **S5**.

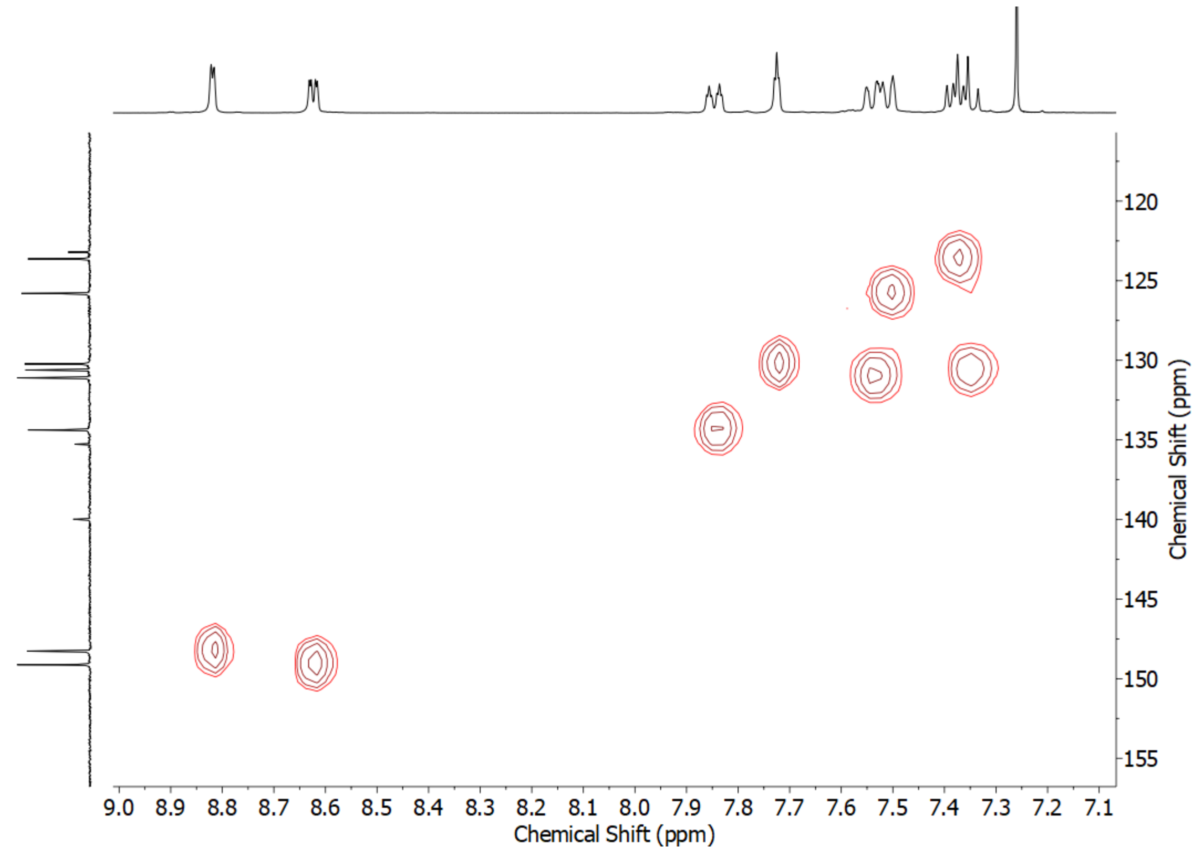

Figure S21 HSQC NMR ($CDCl_3$) of **S5**.

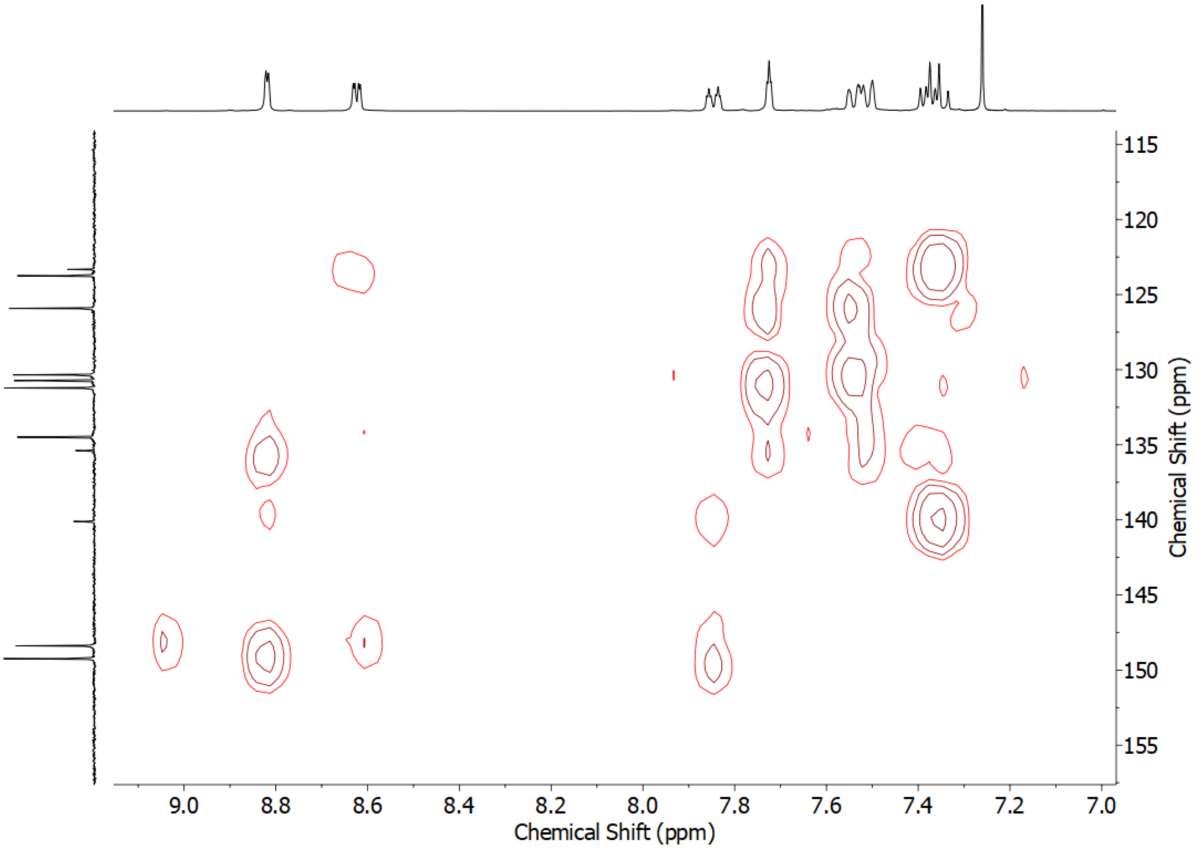

Figure S22 HMBC NMR ($CDCl_3$) of **S5**.

S6

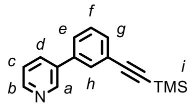

3-(Pyridin-3-yl)bromobenzene (0.277 g, 1.2 mmol, 1 eq.), trimethylsilylacetylene (0.25 mL, 1.8 mmol, 1.5 eq.), $Pd(PPh_3)_2Cl_2$ (0.021 g, 0.030 mmol, 2.5 mol%) and CuI (0.011 g, 0.059 mmol, 5 mol%) in $^{i}Pr_2NH$ (10 mL) were stirred at 80 °C in a sealed vial for 18 h. EDTA solution (25 mL) was added and the aqueous phase extracted with CH_2Cl_2 (3 × 25 mL). The combined organic phases were dried ($MgSO_4$) and the solvent removed *in vacuo*. After purification by column chromatography on silica (1:9 acetone/CH_2Cl_2) the product was obtained as a brown oil (0.281 g, 95%). ^{1}H NMR (400 MHz, $CDCl_3$) δ: 8.83 (d, J = 1.9 Hz, 1H, H_a), 8.61 (dd, J = 4.8, 1.4 Hz, 1H, H_b), 7.86 (ddd, J = 7.9, 2.4, 1.6 Hz, 1H, H_d), 7.69 (app. t, J = 1.5 Hz, 1H, H_h), 7.54-7.49 (2H, H_e, H_g), 7.41 (app. t, J = 7.7 Hz, 1H, H_f), 7.36 (ddd, J = 7.9, 4.8, 0.7 Hz, 1H, H_c), 0.27 (s, 9H, H_i). ^{13}C NMR (101 MHz, $CDCl_3$) δ: 149.0, 148.4, 138.1, 136.0, 134.5, 131.6, 130.8, 129.1, 127.4, 124.2, 123.7, 104.7, 95.1, 0.1. HR-ESI-MS m/z = 252.1214 $[M+H]^+$ calc. 252.1209.

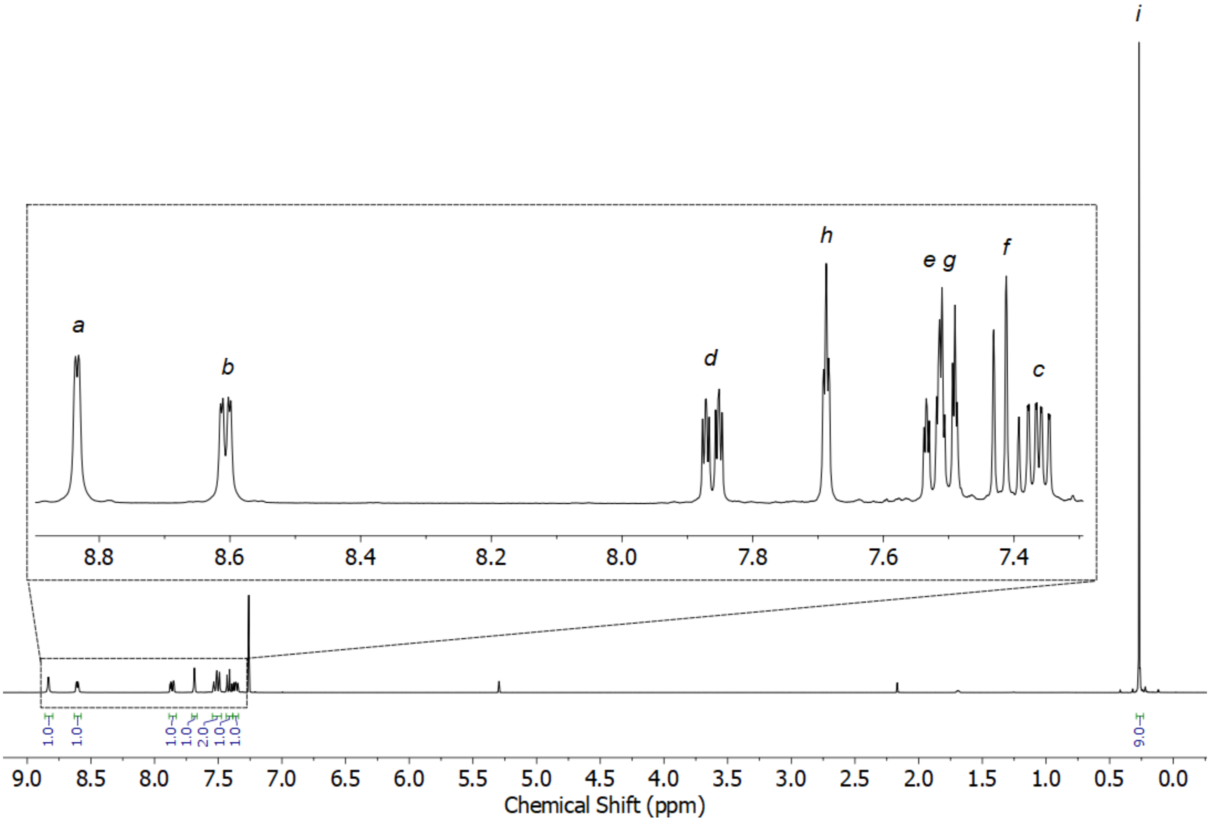

Figure S23 ^{1}H NMR ($CDCl_3$, 400 MHz) of **S6**.

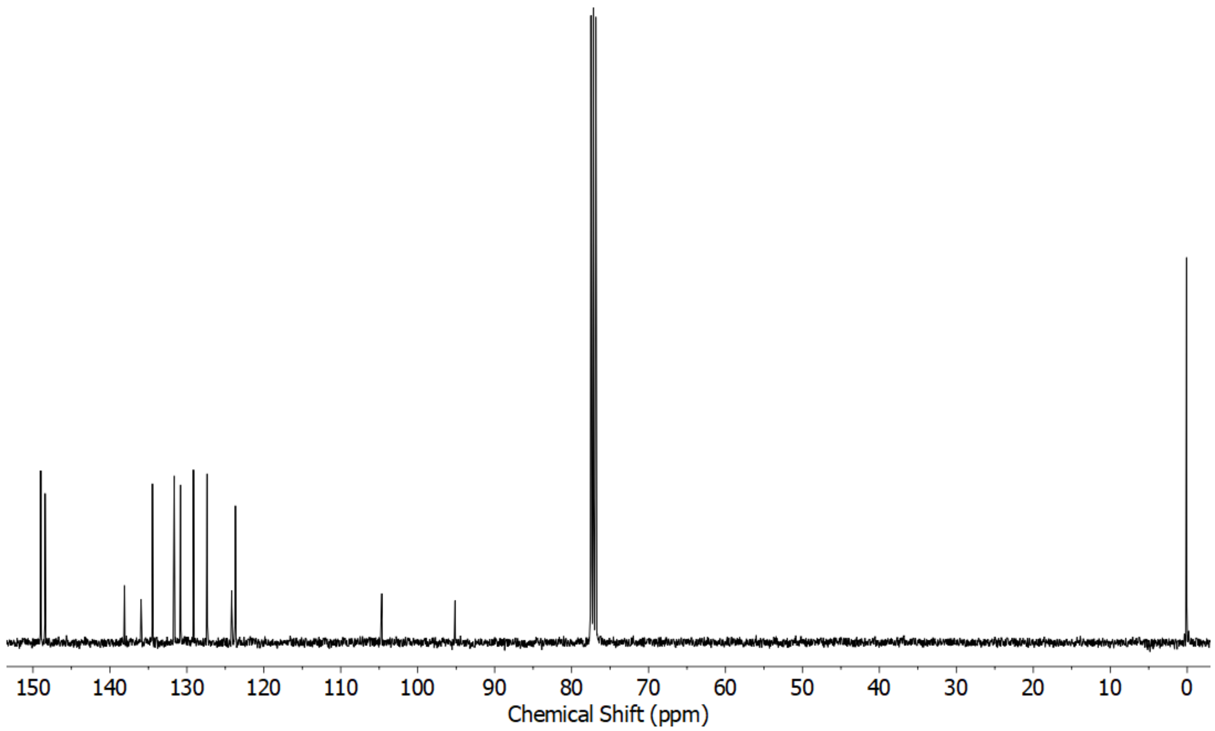

Figure S24 ^{13}C NMR ($CDCl_3$, 101 MHz) of **S6**.

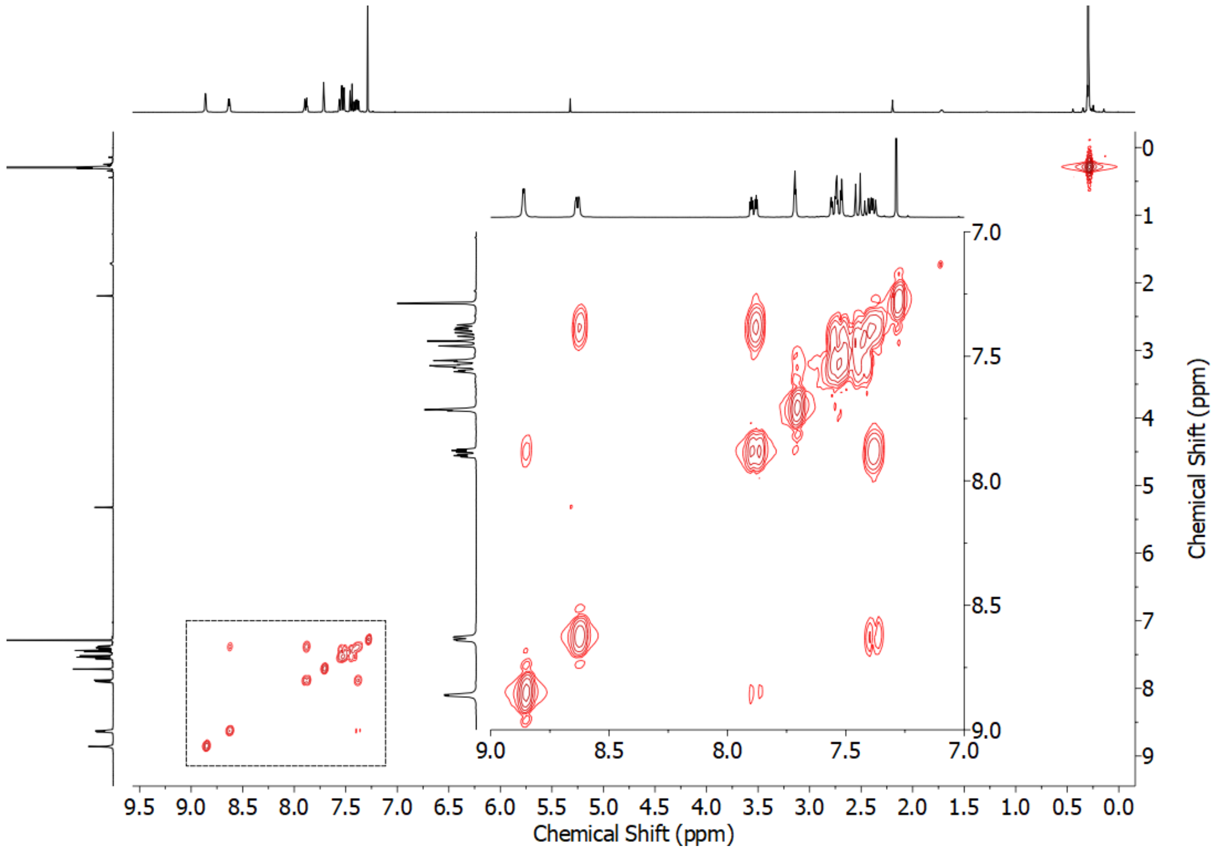

Figure S25 COSY NMR ($CDCl_3$) of **S6**.

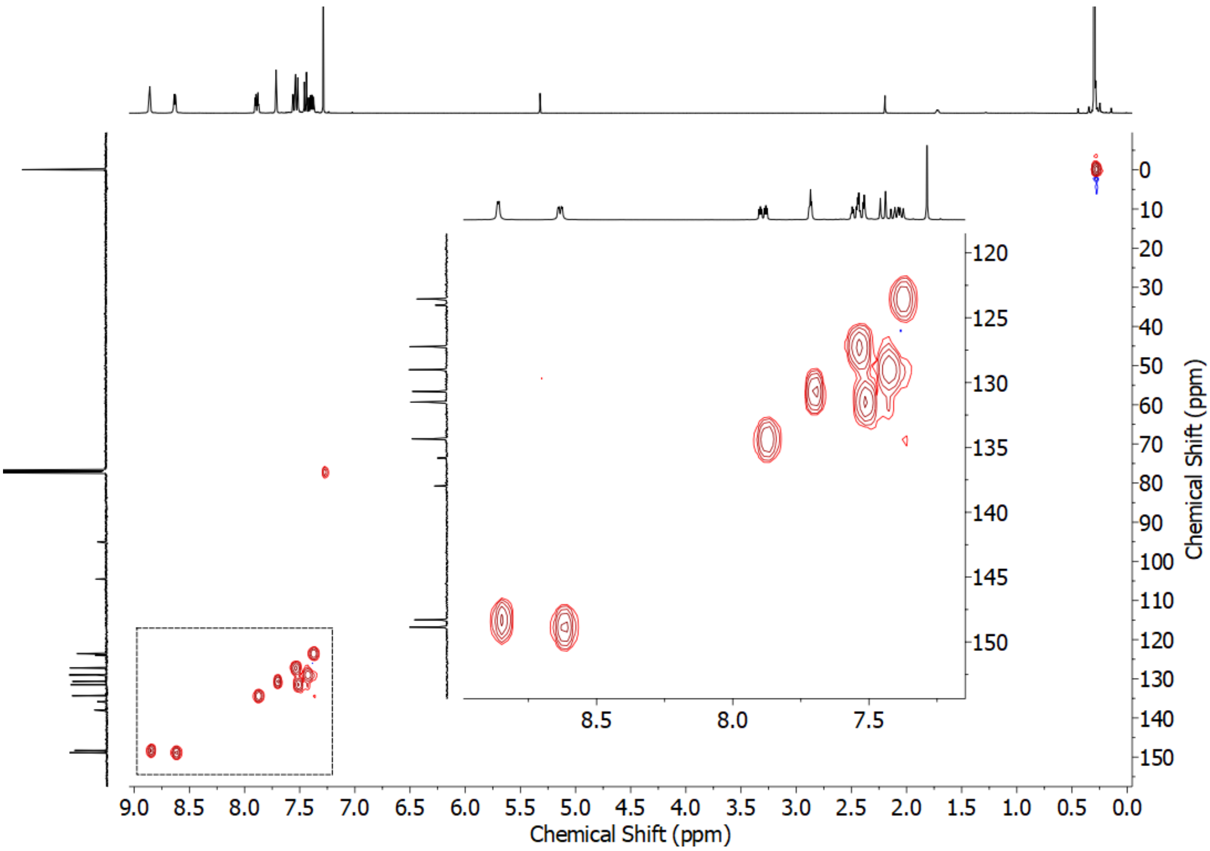

Figure S26 HSQC NMR ($CDCl_3$) of **S6**.

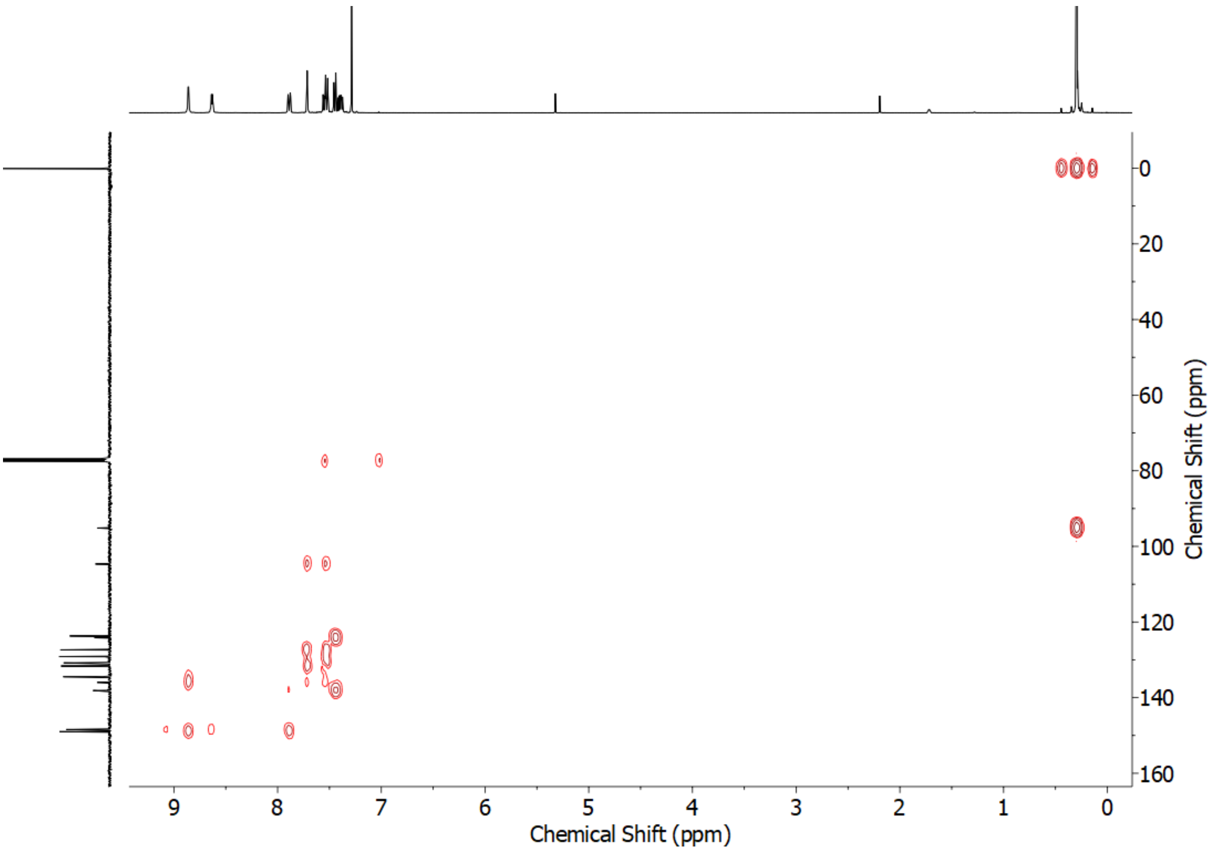

Figure S27 HMBC NMR ($CDCl_3$) of **S6**.

S7

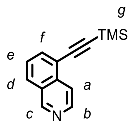

5-Iodoisoquinoline (0.510 g, 2.0 mmol, 1 eq.), trimethylsilylacetylene (0.33 mL, 2.4 mmol, 1.2 eq.), $Pd(PPh_3)_2Cl_2$ (0.028 g, 0.040 mmol, 2 mol%) and CuI (0.015 g, 0.080 mmol, 4 mol%) were stirred at rt in $^{i}Pr_2NH$ (15 mL) for 36 h. EDTA solution (25 mL) was added and the aqueous phase extracted with CH_2Cl_2 (3 × 20 mL). The combined organic phases were dried ($MgSO_4$) and the solvent removed *in vacuo*. After purification by column chromatography on silica (1:4 EtOAc/petrol) the product was obtained as a brown oil (0.445 g, 99%). ^{1}H NMR (400 MHz, $CDCl_3$) δ: 9.25 (d, *J* = 1.0 Hz, 1H, H_c), 8.62 (d, *J* = 5.8 Hz, 1H, H_b), 8.07 (d, *J* = 5.9 Hz, 1H, H_a), 7.94 (d, *J* = 8.3 Hz, 1H, H_d), 7.88 (dd, *J* = 7.2, 1.1, 1H, H_f), 7.55 (dd, *J* = 8.2, 7.2 Hz, 1H, H_e), 0.34 (s, 9H, H_g). ^{13}C NMR (101 MHz, $CDCl_3$) δ: 152.9, 144.2, 136.3, 134.6, 128.4, 128.4, 126.7, 120.3, 119.0, 101.4, 101.2, 0.1. HR-ESI-MS *m/z* = 226.1055 $[M+H]^+$ calc. 226.1052.

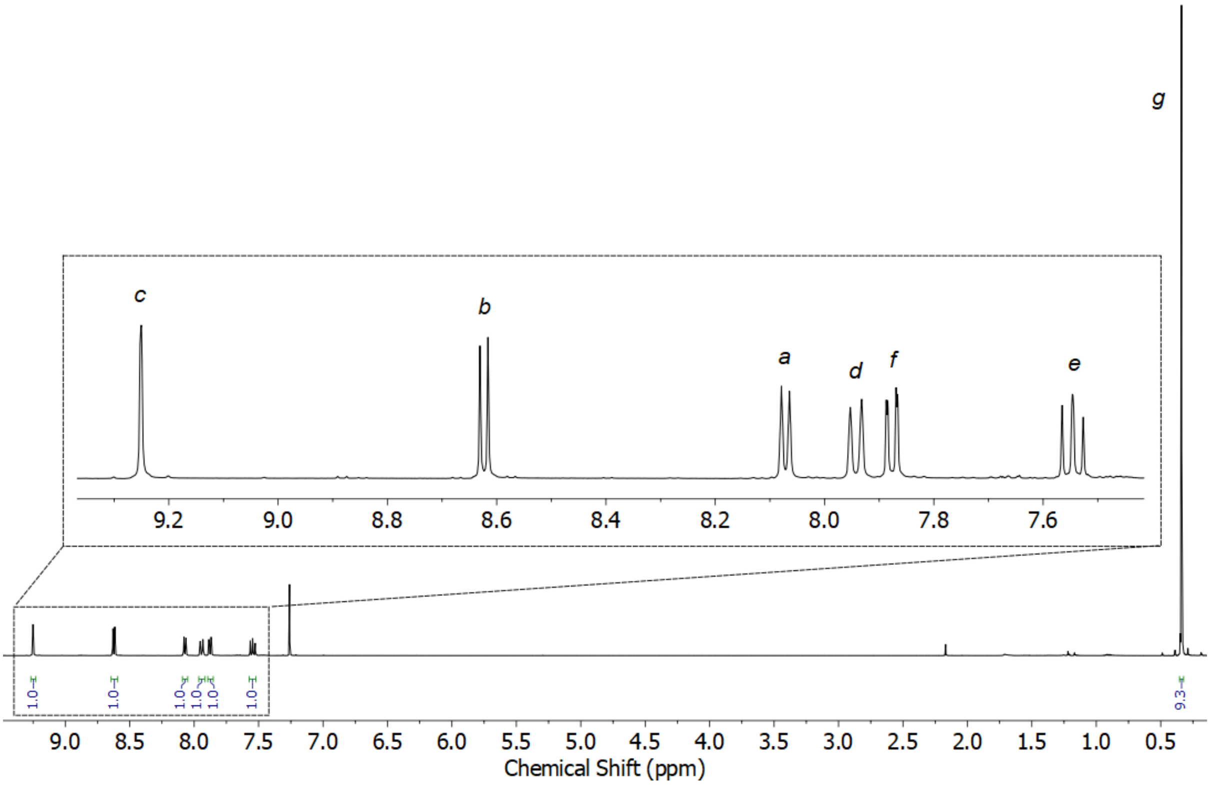

Figure S28 ^{1}H NMR ($CDCl_3$, 400 MHz) of **S7**.

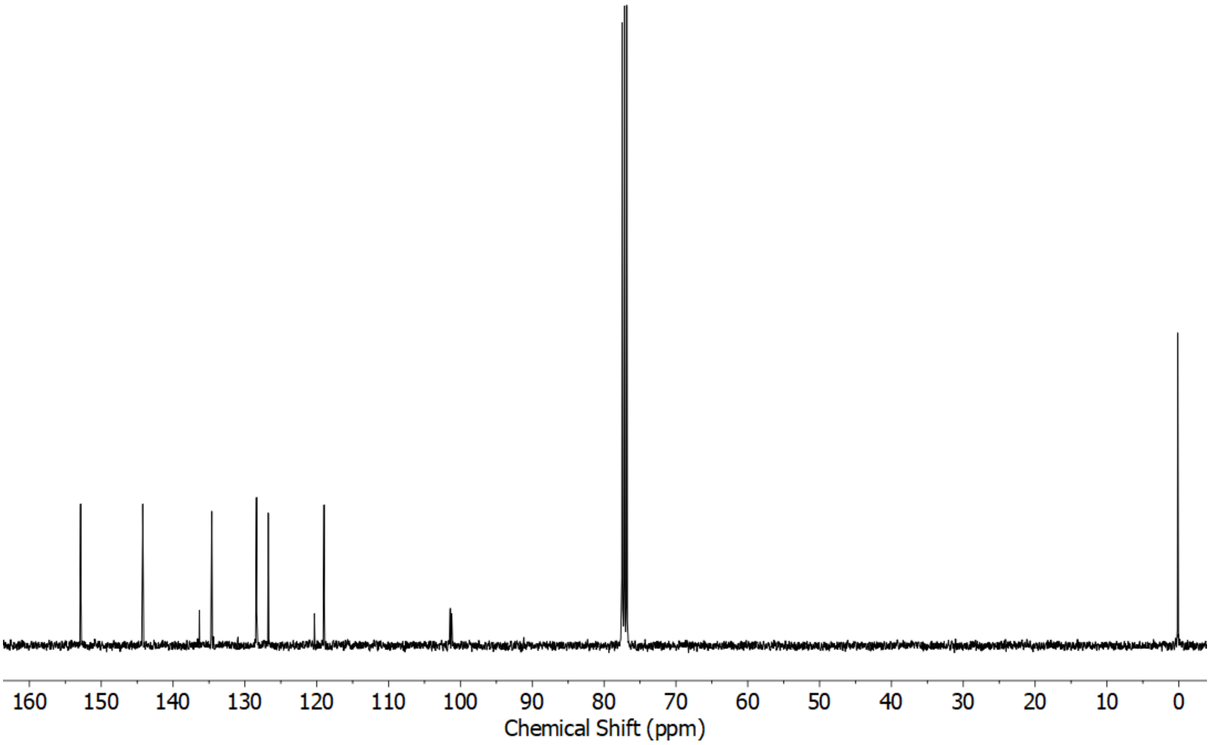

Figure S29 ^{13}C NMR ($CDCl_3$, 101 MHz) of **S7**.

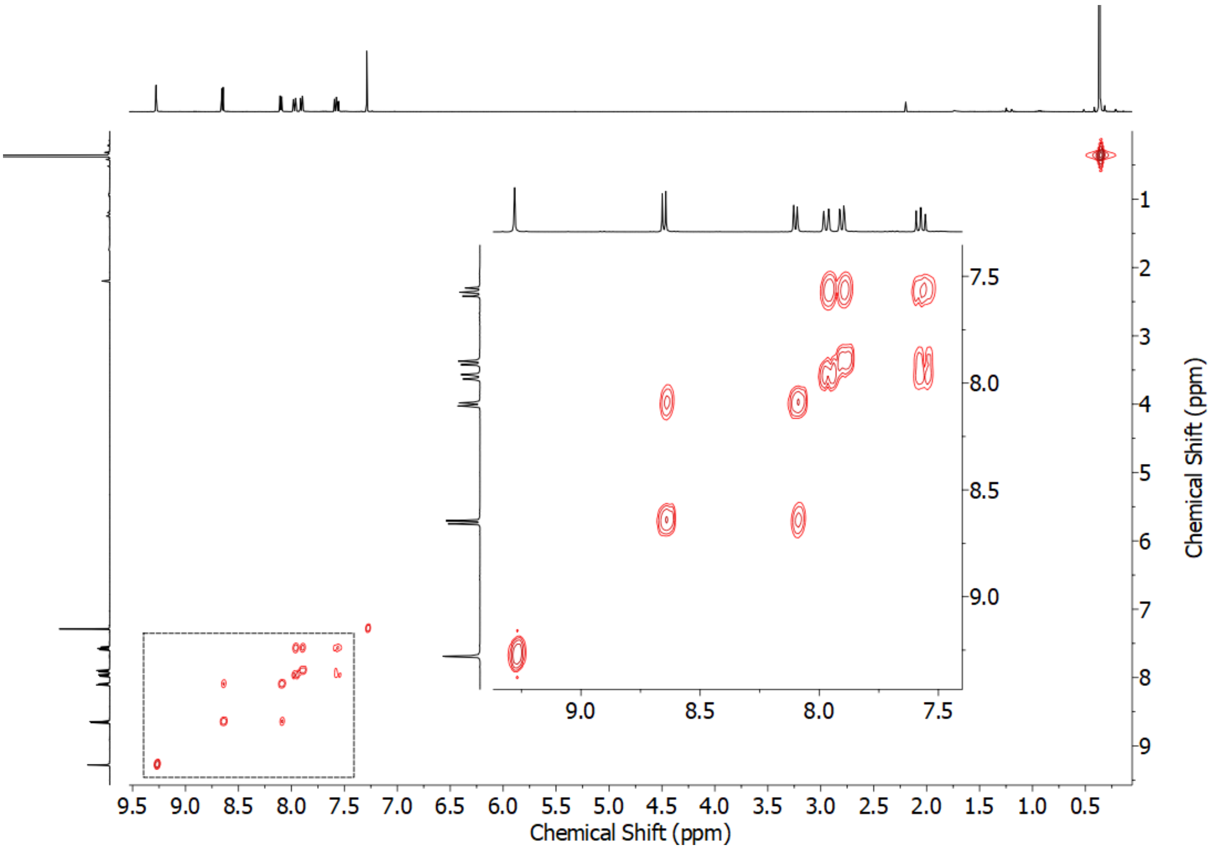

Figure S30 COSY NMR ($CDCl_3$) of **S7**.

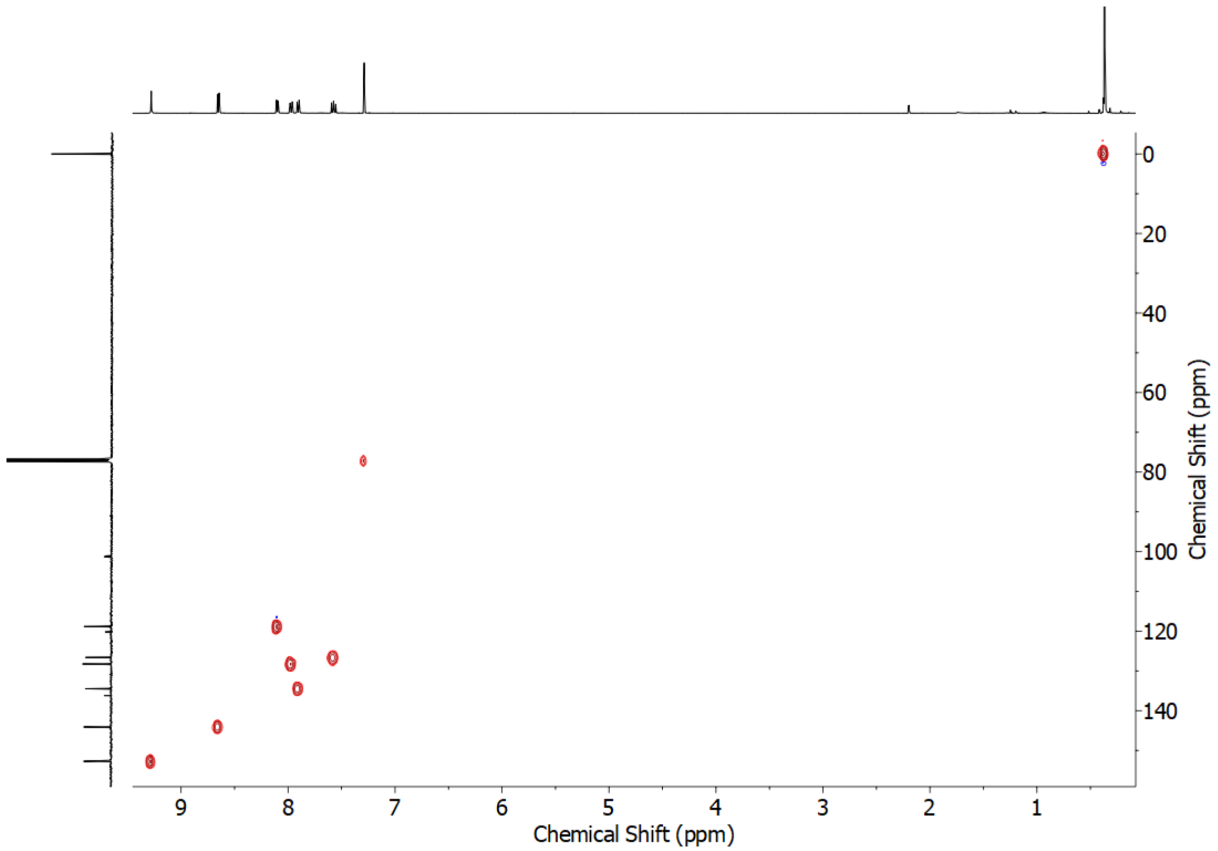

Figure S31 HSQC NMR ($CDCl_3$) of **S7**.

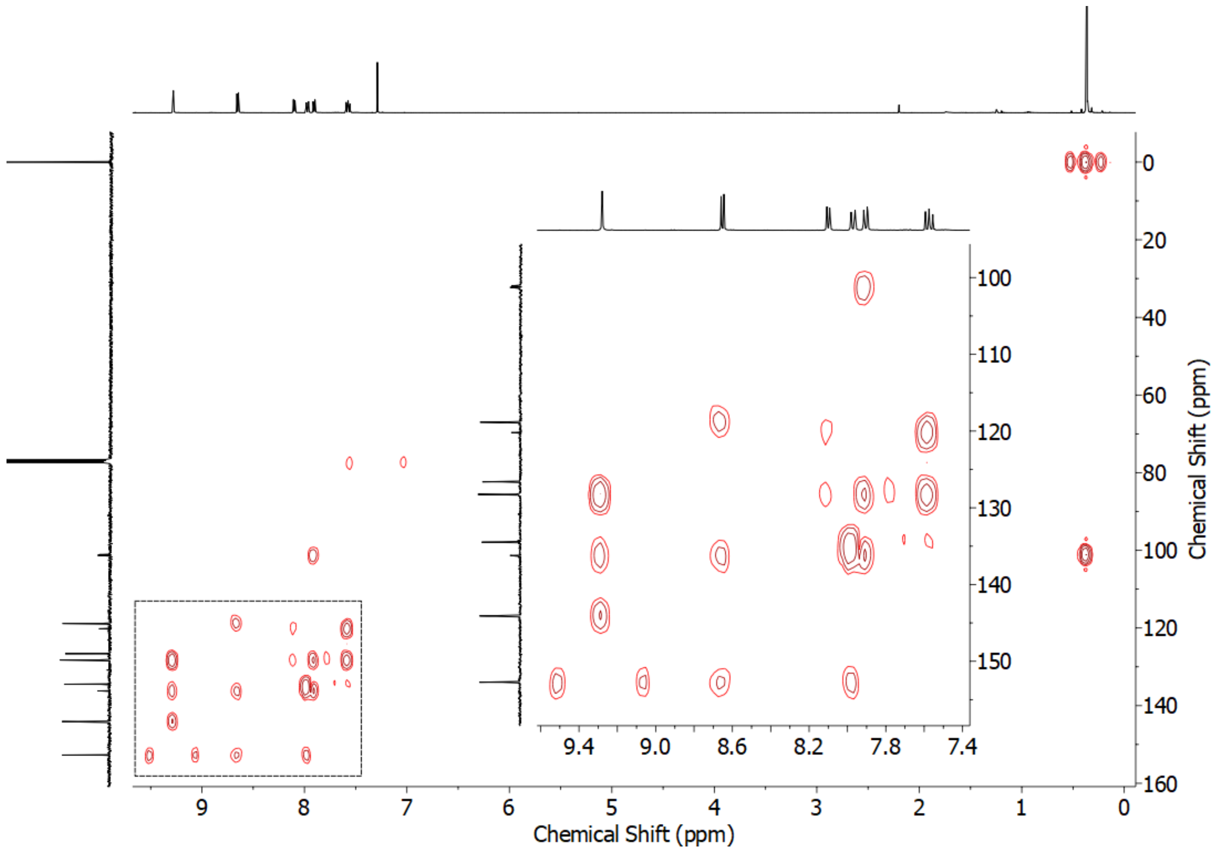

Figure S32 HMBC NMR ($CDCl_3$) of **S7**.

S8

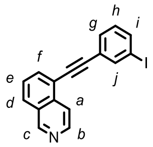

To **S7** (0.225 g, 1.0 mmol, 1 eq.), 1,3-diiodobenzene (0.660 g, 2.0 mmol, 2 eq.), $Pd(PPh_3)_2Cl_2$ (0.014 g, 0.020 mmol, 2 mol%) and CuI (0.008 g, 0.04 mmol, 4 mol%) in CH_3CN (5 mL) was added DBU (1.8 mL, 12 mmol, 12 eq.) via syringe and the reaction mixture stirred at rt for 22 h. EDTA solution (20 mL) was added and the aqueous phase extracted with CH_2Cl_2 (3 × 20 mL). The combined organic phases were dried ($MgSO_4$) and the solvent removed *in vacuo*. After purification by column chromatography on silica (pentane followed by 1:1 EtOAc/pentane) the product was obtained as a yellow oil that solidified on standing (0.226 g, 64%). ^{1}H NMR (400 MHz, $CDCl_3$) δ: 9.28 (d, *J* = 1.0 Hz, 1H, H_c), 8.65 (d, *J* = 5.8 Hz, 1H, H_b), 8.13 (app. dt, *J* = 5.7, 1.0 Hz, 1H, H_a), 8.00-7.97 (m, 2H, H_d/H_f, H_j), 7.93 (dd, *J* = 7.2, 1.2 Hz, 1H, H_d/H_f), 7.73 (ddd, *J* = 8.0, 1.7, 1.0 Hz, 1H, H_g/H_i), 7.62-7.58 (m, 2H, H_e, H_g/H_i), 7.14 (app. t, *J* = 7.9 Hz, 1H, H_h). ^{13}C NMR (101 MHz, $CDCl_3$) δ: 153.0, 144.3, 140.3, 137.9, 136.1, 134.4, 131.0, 130.1, 128.5, 128.5, 126.9, 125.0, 120.0, 118.9, 94.0, 93.7, 87.3. HR-ESI-MS *m/z* = 355.9942 $[M+H]^+$ calc. 355.9936.

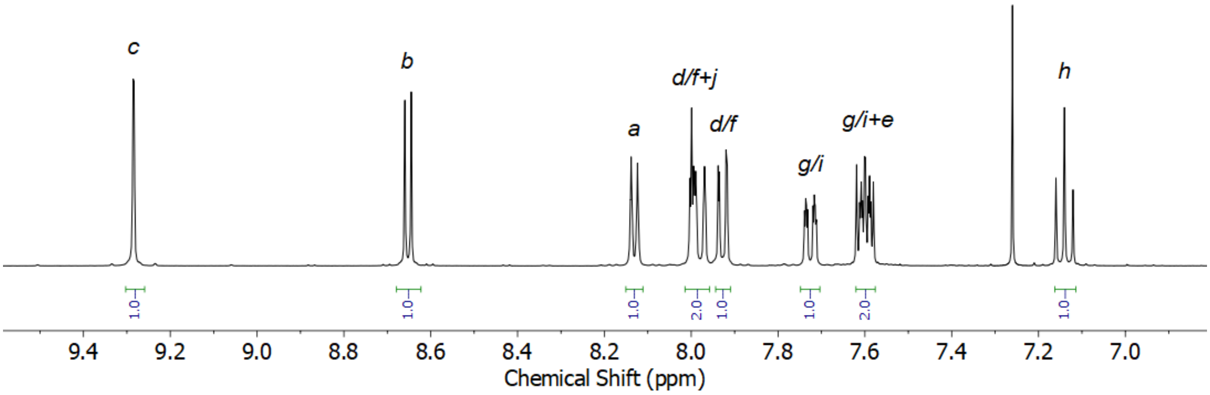

Figure S33 ^{1}H NMR ($CDCl_3$, 400 MHz) of **S8**.

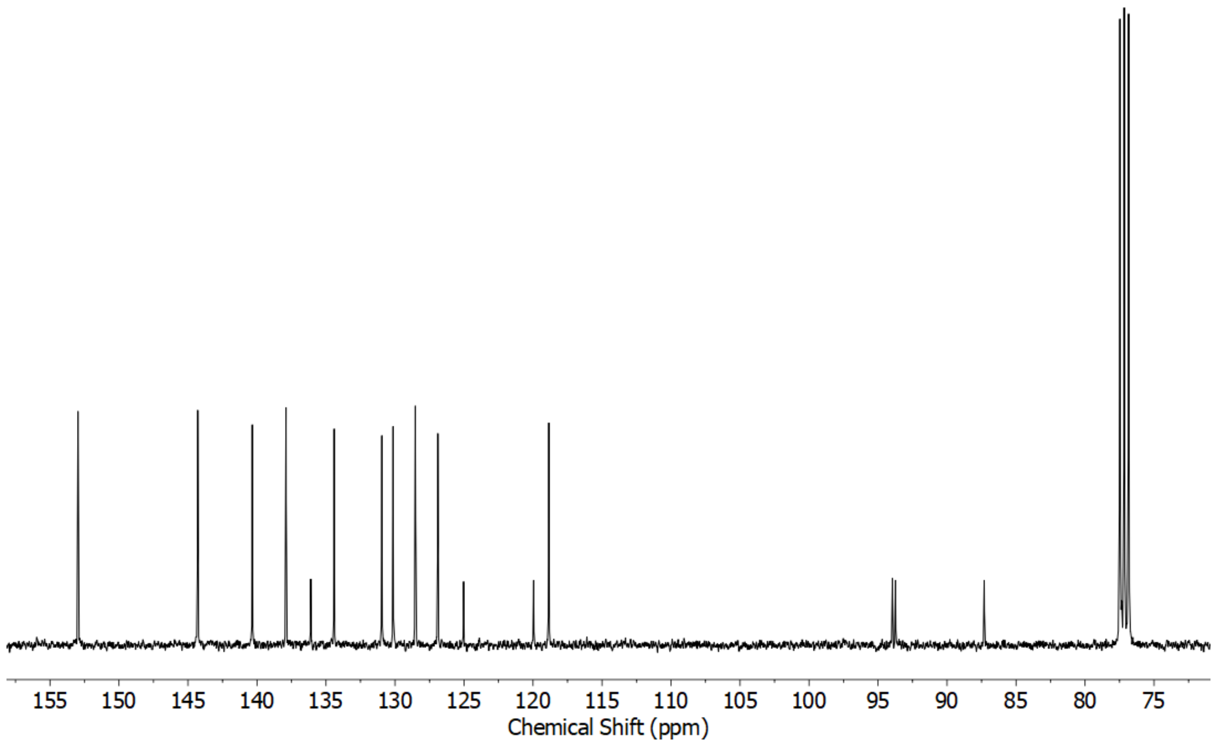

Figure S34 ^{13}C NMR ($CDCl_3$, 101 MHz) of **S8**.

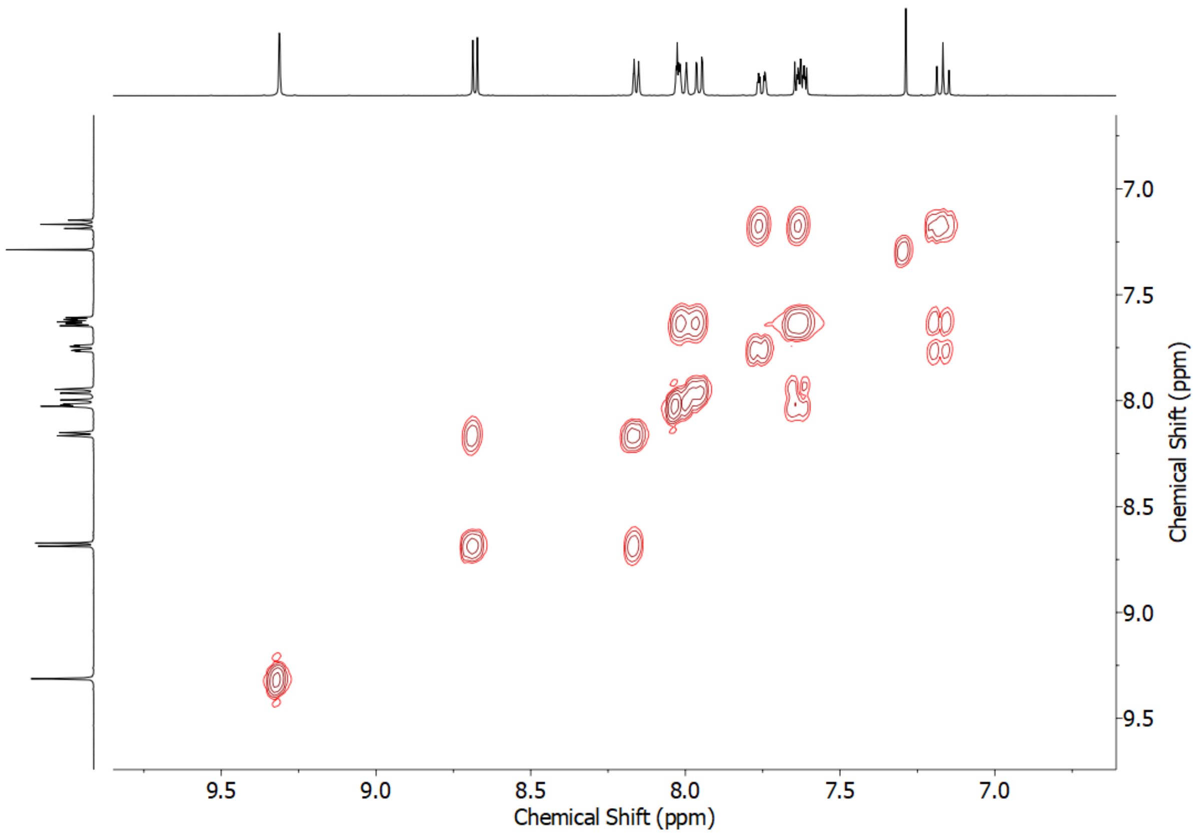

Figure S35 COSY NMR ($CDCl_3$) of **S8**.

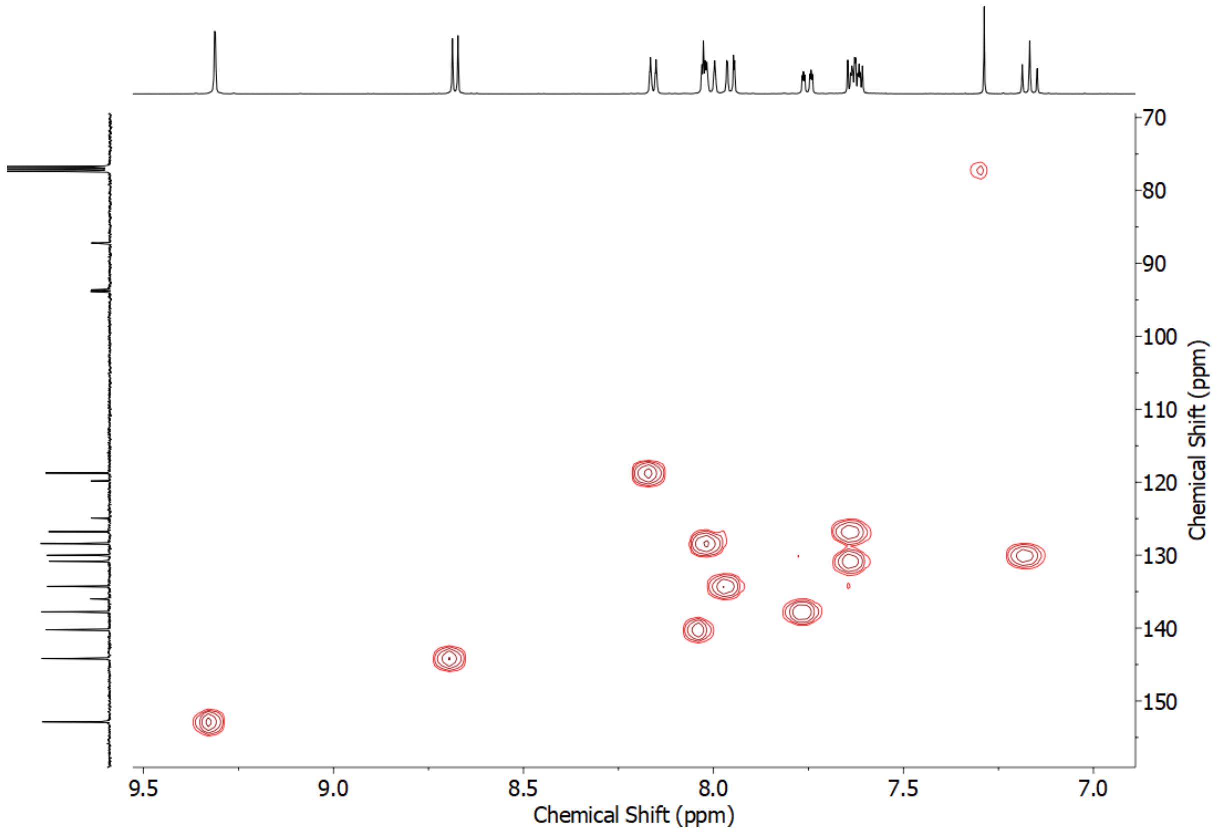

Figure S36 HSQC NMR ($CDCl_3$) of **S8**.

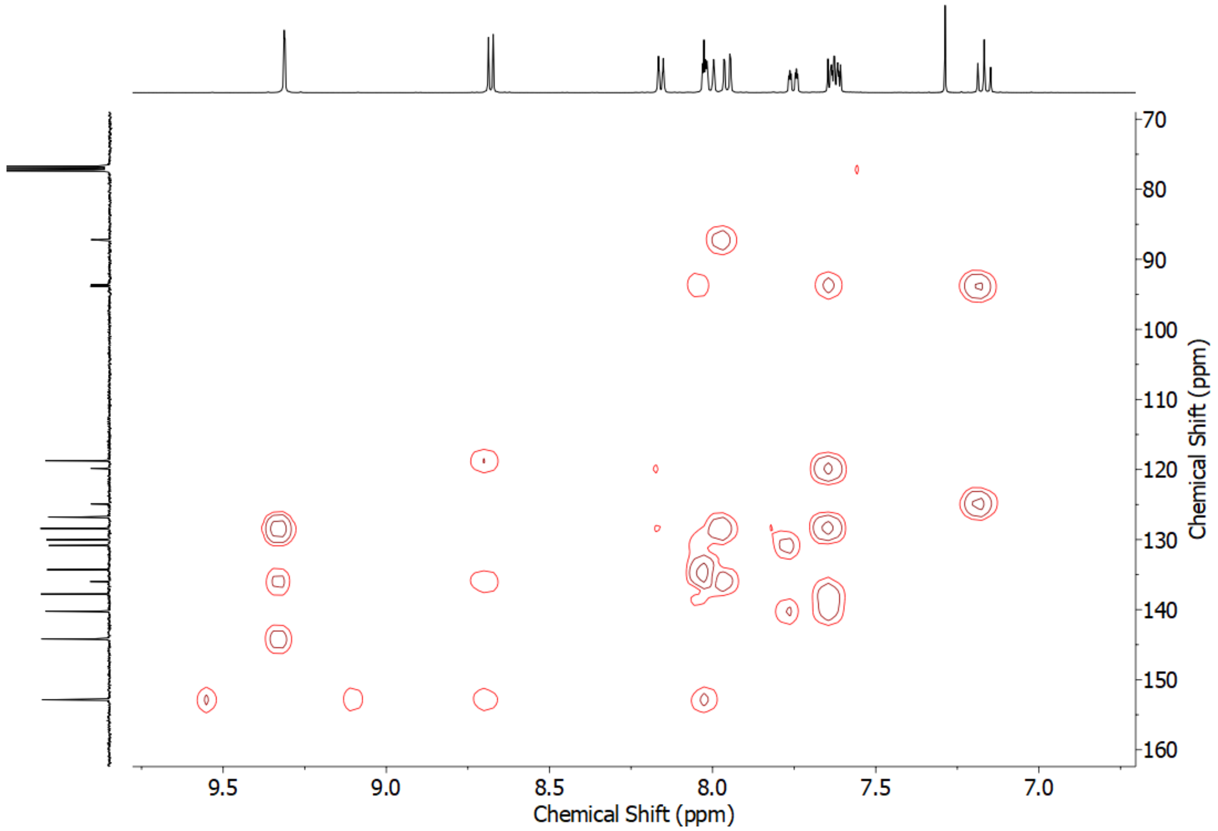

Figure S37 HMBC NMR ($CDCl_3$) of **S8**.

S9

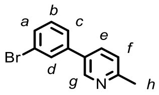

To a stirring solution of 2-methylpyridine-5-boronic acid pinacol ester (0.657 g, 3.0 mmol, 1 eq.), 1,3-dibromobenzene (0.708 g, 3.0 mmol, 1 eq.) and $Pd(PPh_3)_2Cl_2$ (0.105 g, 0.15 mmol, 5 mol%) in dioxane (10 mL) was added a solution of K_2CO_3 (1.04 g, 7.5 mmol, 2.5 eq.) in H_2O (5 mL). The reaction mixture was stirred at 100 °C for 19 h. EDTA solution (20 mL) was added and the aqueous phase extracted with CH_2Cl_2 (3 × 20 mL). The combined organic phases were dried ($MgSO_4$) and the solvent removed *in vacuo*. After purification by column chromatography on silica (CH_2Cl_2 followed by 1:9 acetone/CH_2Cl_2), the product was obtained as a colourless oil that solidified on standing (0.400 g, 54%). M.p. 54-56 °C. ^{1}H NMR (400 MHz, $CDCl_3$) δ: 8.69 (d, *J* = 2.1 Hz, 1H, H_g), 7.74 (dd, *J* = 8.0, 2.4 Hz, 1H, H_e), 7.70 (app. t, *J* = 1.8 Hz, 1H, H_d), 7.52-7.47 (m, 2H, H_a, H_c), 7.33 (app. t, *J* = 7.9 Hz, 1H, H_b), 7.23 (d, *J* = 8.0 Hz, 1H, H_f). ^{13}C NMR (101 MHz, $CDCl_3$) δ: 158.1, 147.6, 140.2, 134.8, 132.5, 130.9, 130.7, 130.1, 125.7, 123.4, 123.3, 24.3. HR-ESI-MS *m/z* = 248.0067 $[M+H]^+$ calc. 248.0075.

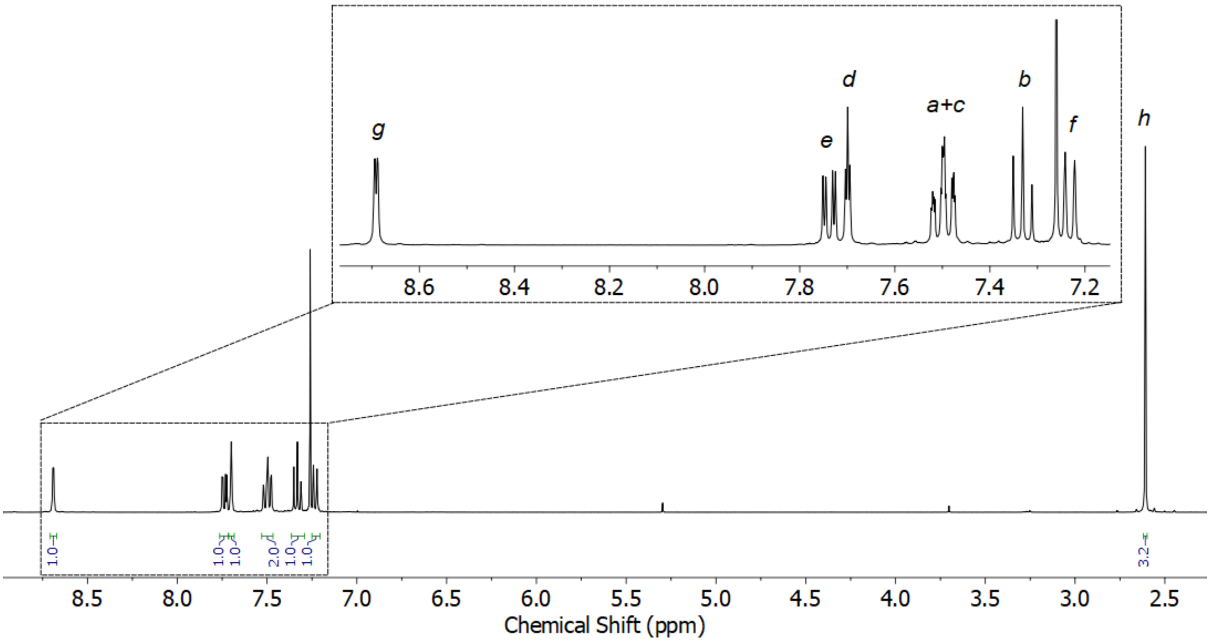

Figure S38 ^{1}H NMR ($CDCl_3$, 400 MHz) of **S9**.

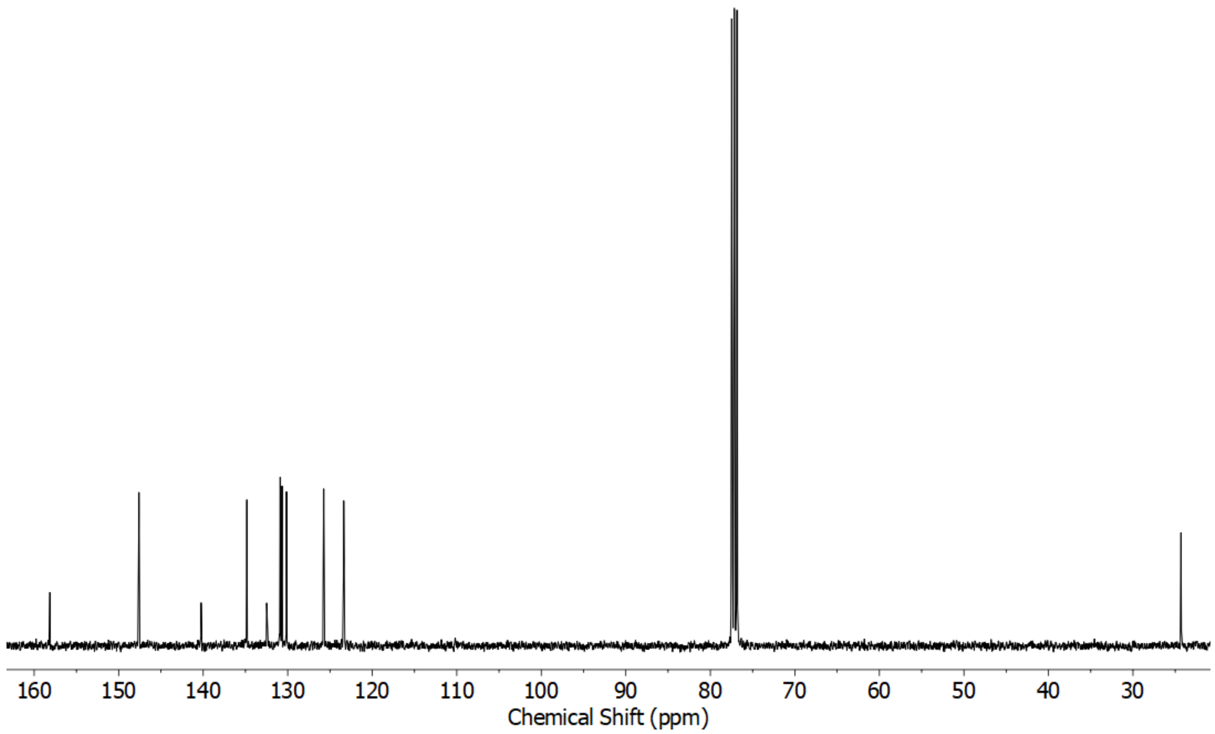

Figure S39 ^{13}C NMR ($CDCl_3$, 101 MHz) of **S9**.

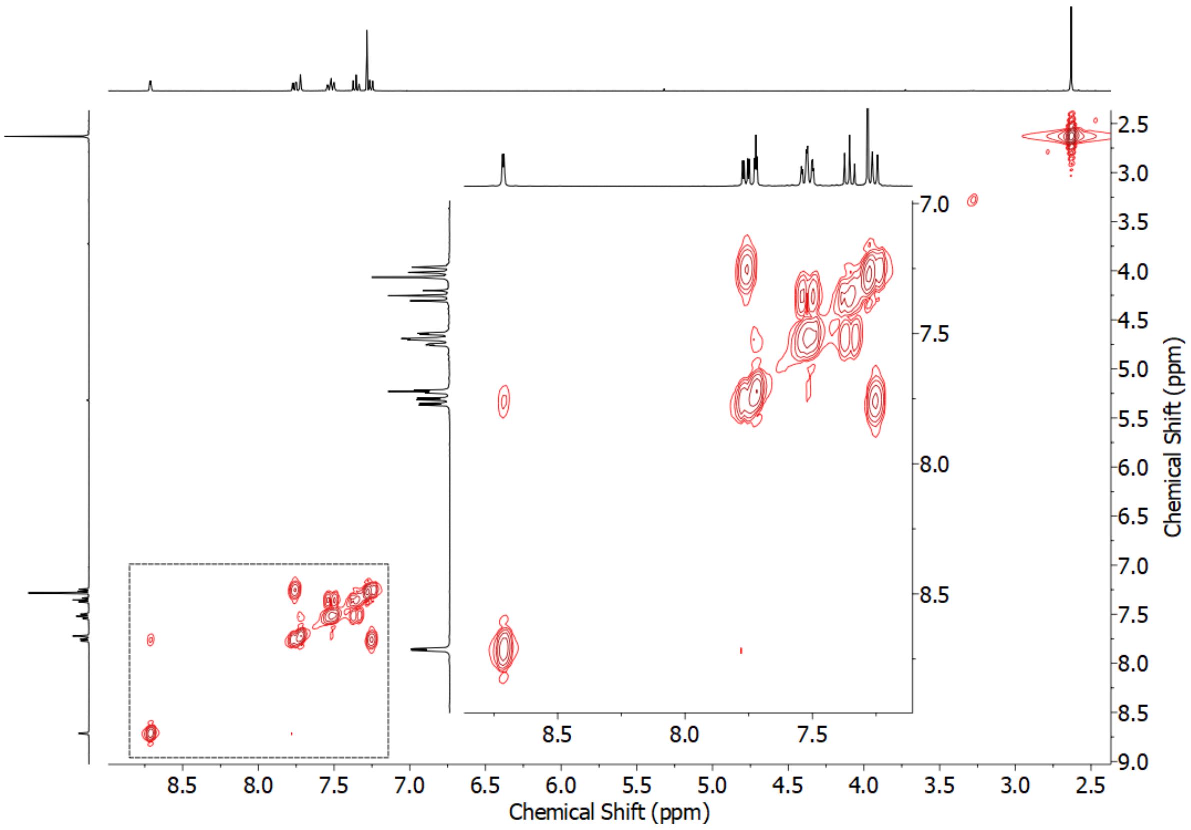

Figure S40 COSY NMR ($CDCl_3$) of **S9**.

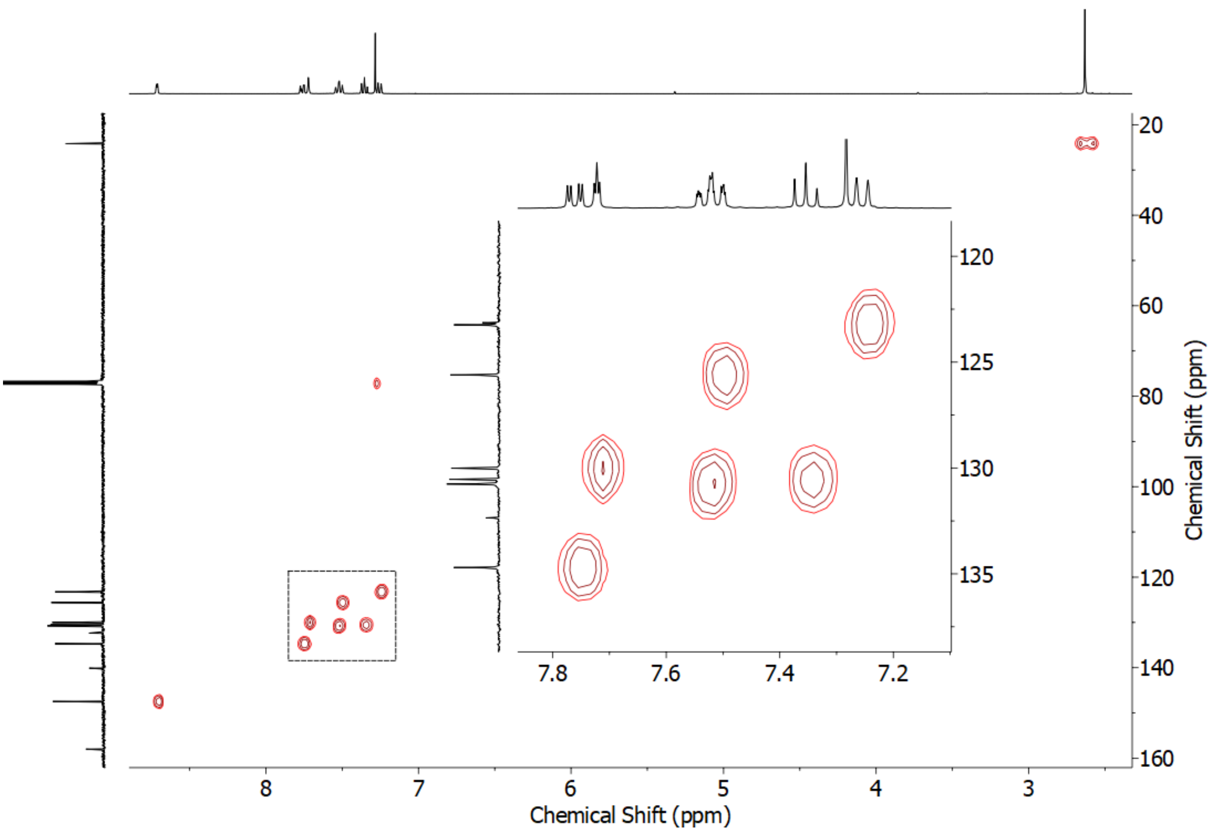

Figure S41 HSQC NMR ($CDCl_3$) of **S9**.

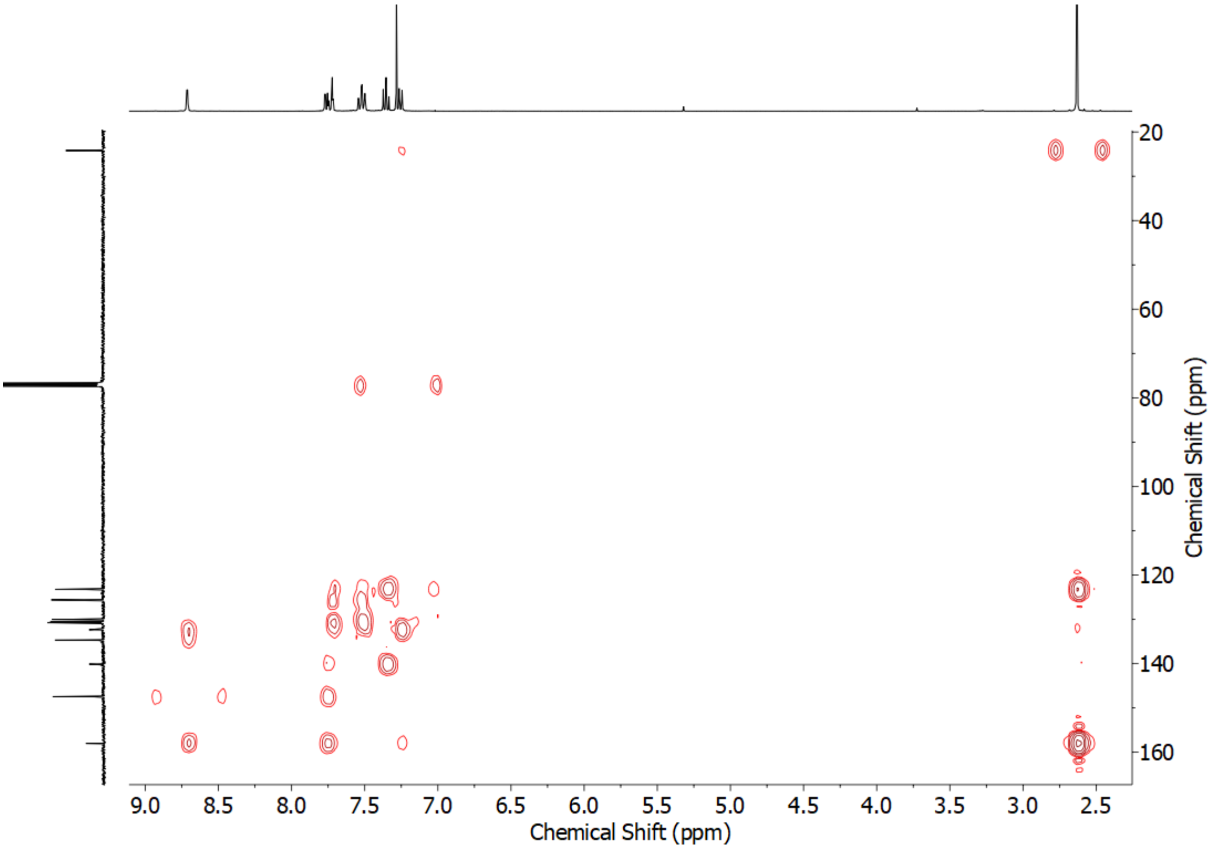

Figure S42 HMBC NMR ($CDCl_3$) of **S9**.

1

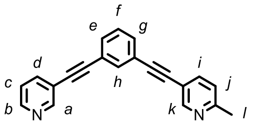

To **S1** (0.153 mg, 0.50 mmol, 1 eq.), **S2** (0.142 g, 0.75 mmol, 1.5 eq.), $Pd(PPh_3)_2Cl_2$ (0.018 g, 0.025 mmol, 5 mol%) and CuI (0.010 g, 0.050 mmol, 10 mol%) in CH_3CN (2.5 mL) was added DBU (0.90 mL, 6.0 mmol, 12 eq.) via syringe and the reaction stirred at rt for 18 h. EDTA solution (25 mL) was added and the aqueous phase was extracted with CH_2Cl_2 (3 × 20 mL). The combined organic phases were dried ($MgSO_4$) and the solvent removed *in vacuo*. After purification by column chromatography on silica (1:1 pentane/Et_2O followed by neat Et_2O) the product was obtained as a yellow solid (0.132 g, 90%). M.p. 92-94 °C. ^1H NMR (400 MHz, $CDCl_3$) δ: 8.77 (dd, *J* = 2.1, 0.9 Hz, 1H, H_a), 8.66 (d, *J* = 1.8 Hz, 1H, H_k), 8.57 (dd, *J* = 4.9, 1.7 Hz, 1H, H_b), 7.81 (app. dt, *J* = 7.9, 1.9 Hz, 1H, H_d), 7.74 (t, *J* = 1.4 Hz, 1H, H_h), 7.70 (dd, *J* = 8.0, 2.2 Hz, 1H, H_i/H_j), 7.54-7.51 (m, 2H, H_e, H_g), 7.37 (app. t, *J* = 7.8 Hz, 1H, H_f), 7.30 (ddd, *J* = 7.9, 4.9, 0.9 Hz, 1H, H_c), 7.16 (d, *J* = 8.0 Hz, 1H, H_i/H_j), 2.59 (s, 3H, H_l). ^{13}C NMR (101 MHz, $CDCl_3$) δ: 158.2, 152.5, 151.8, 149.0, 138.8, 138.6, 134.8, 131.9, 131.8, 128.8, 123.4, 123.2, 123.1, 122.9, 120.3, 117.1, 91.8, 90.9, 87.2, 86.8, 24.7. HR-ESI-MS *m/z* = 295.1244 $[M+H]^+$ calc. 295.1235.

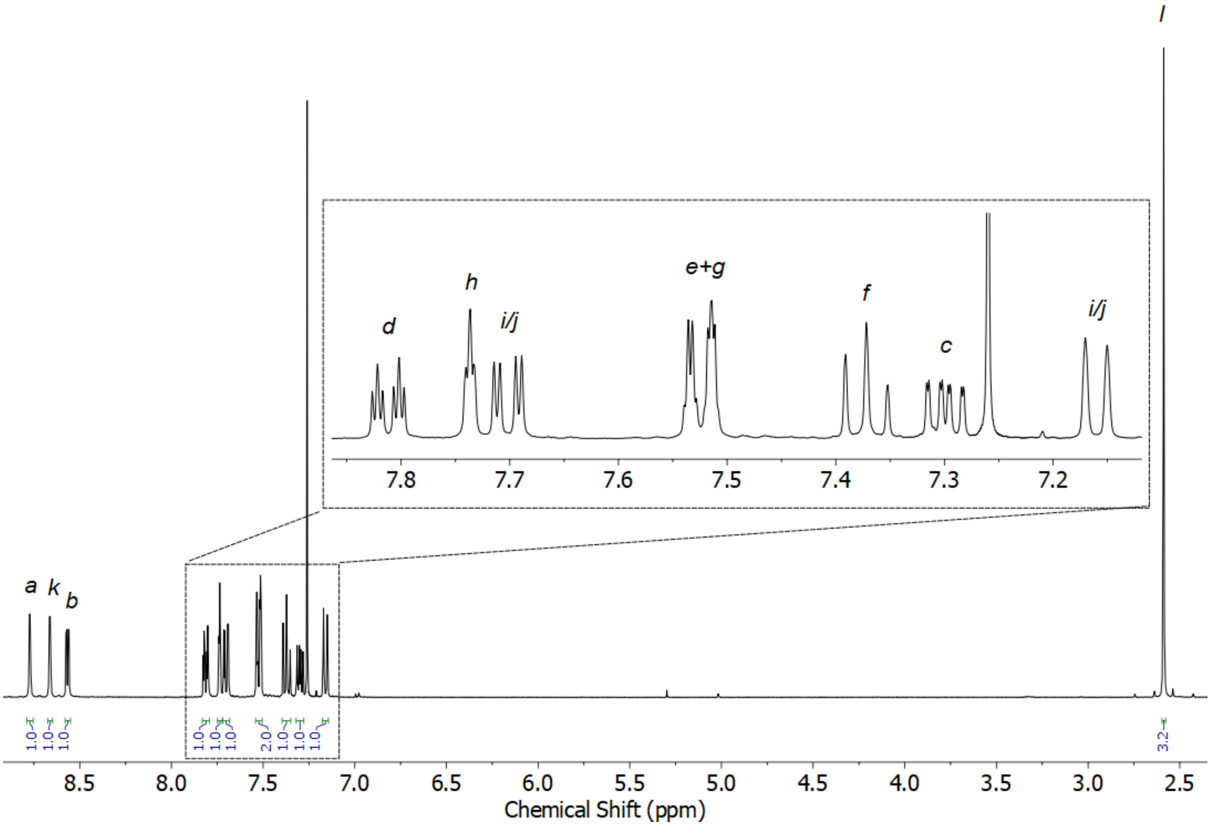

Figure S43 ^1H NMR ($CDCl_3$, 400 MHz) of **1**.

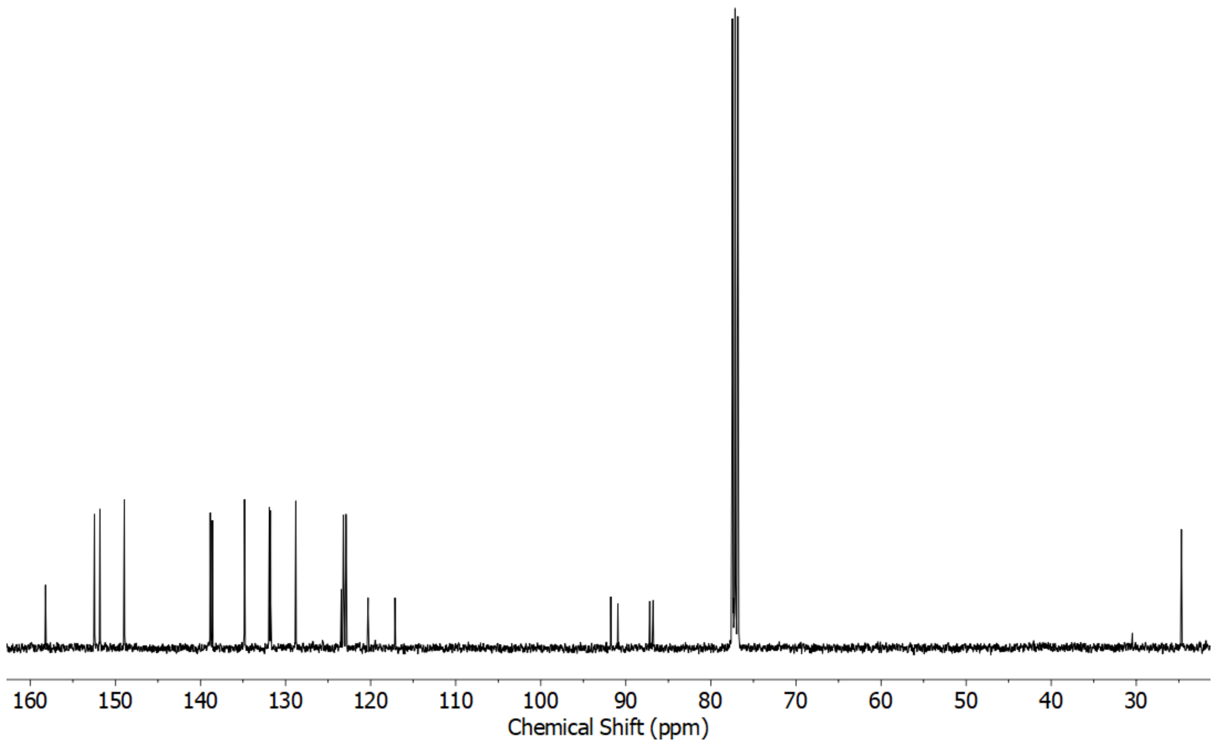

Figure S44 ^{13}C NMR ($CDCl_3$, 101 MHz) of **1**.

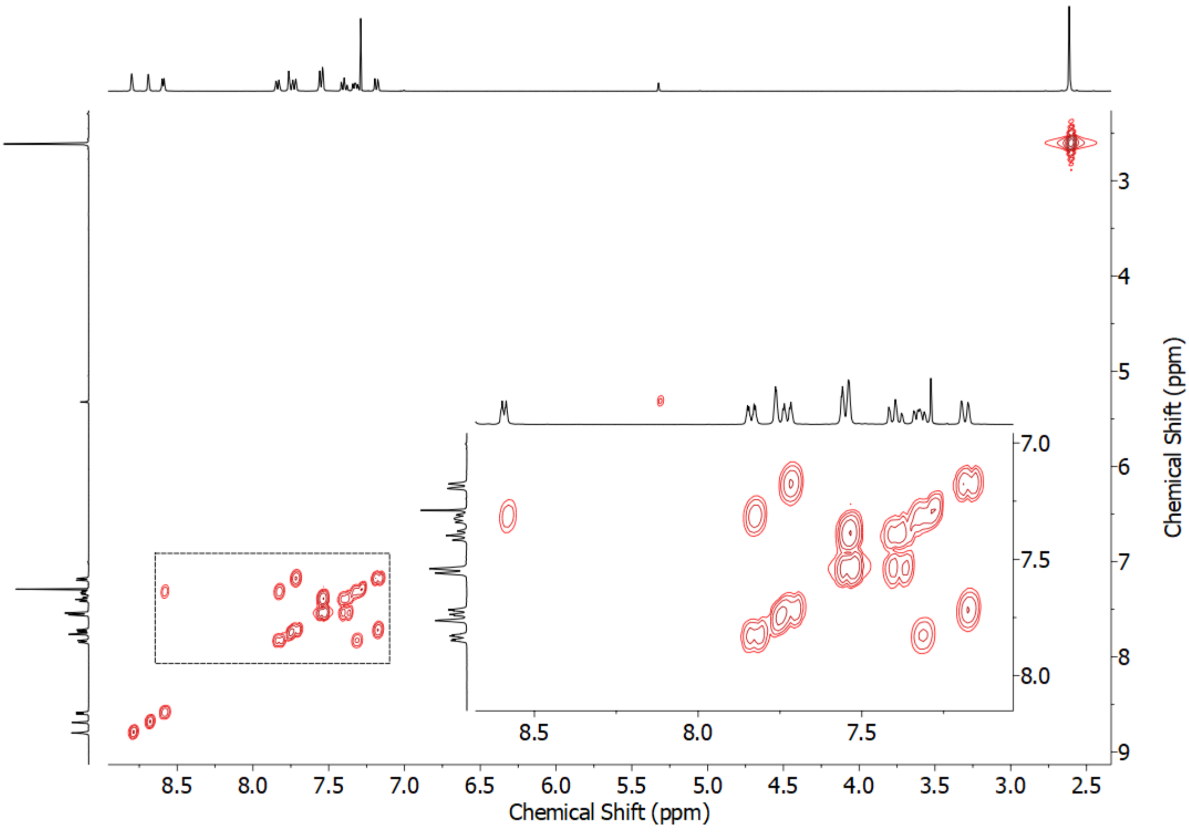

Figure S45 COSY NMR ($CDCl_3$) of **1**.

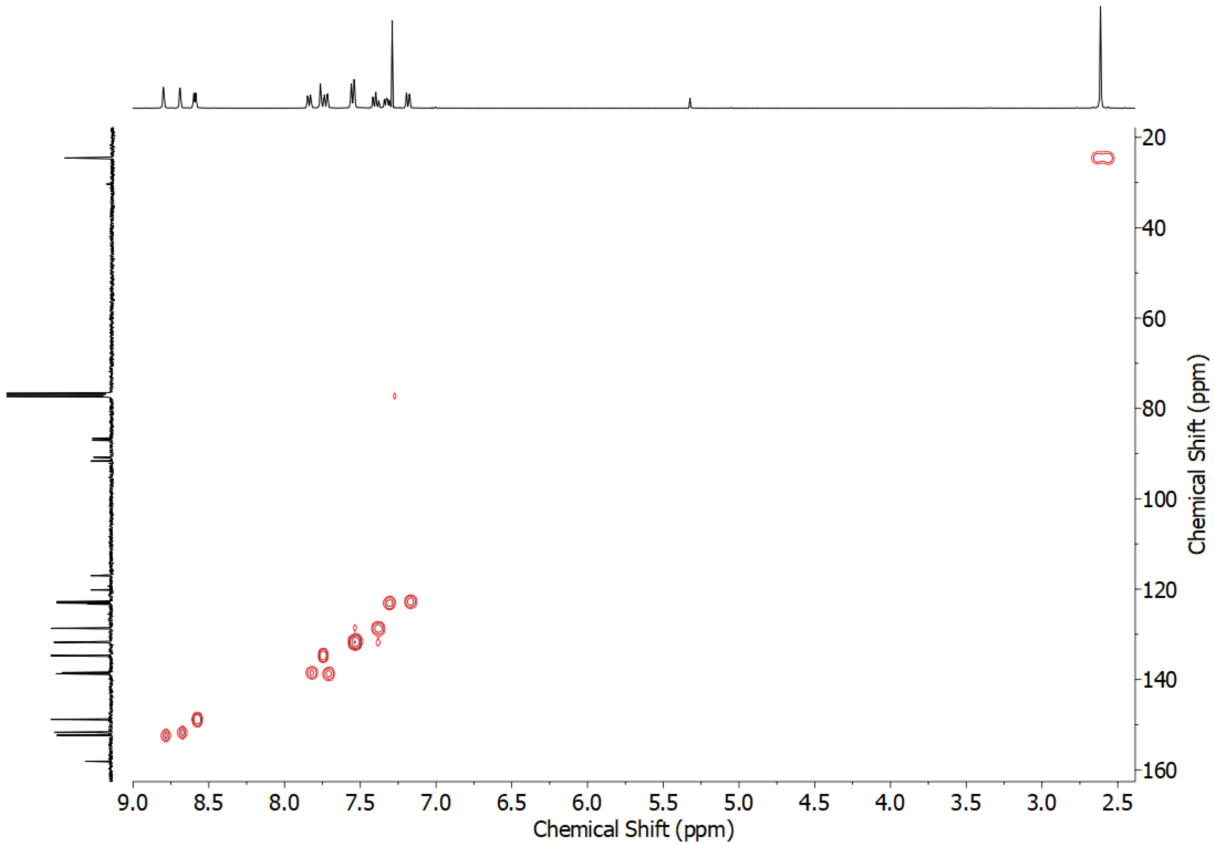

Figure S46 HSQC NMR ($CDCl_3$) of **1**.

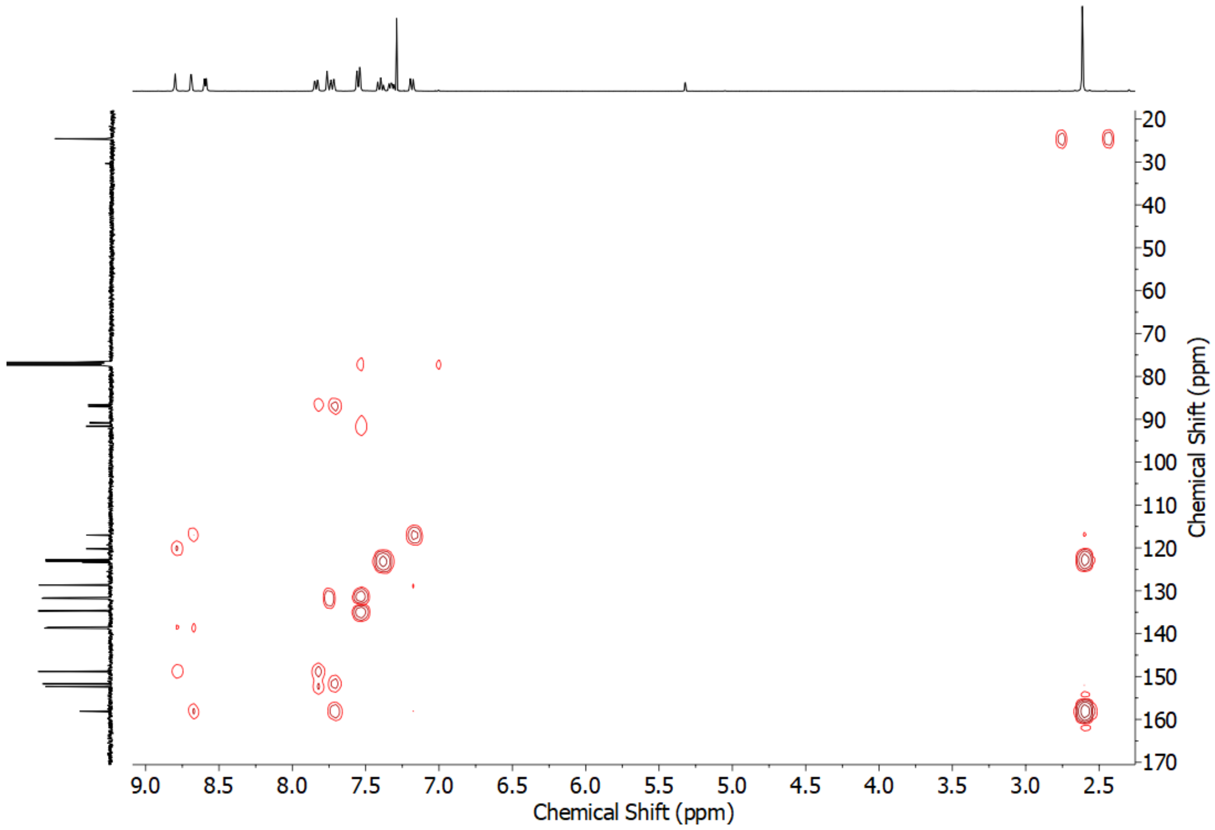

Figure S47 HMBC NMR ($CDCl_3$) of **1**.

2

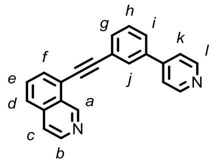

To a vigorously stirring solution of **S4** (0.178 g, 0.50 mmol, 1 eq.), 4-pyridinylboronic acid (0.074 g, 0.60 mmol, 1.2 eq.) and $Pd(PPh_3)_2Cl_2$ (0.018 g, 0.025 mmol, 5 mol%) in dioxane (2.0 mL) was added K_2CO_3 (0.173 g, 1.25 mmol, 2.5 eq.) in H_2O (1.0 mL) via syringe. The reaction mixture was stirred at 80 °C for 16 h. EDTA solution (20 mL) was added, and the aqueous phase extracted with CH_2Cl_2 (3 × 20 mL). The combined organic phases were dried ($MgSO_4$) and the solvent removed *in vacuo*. After purification by column chromatography on silica (2:3 MeCN/CH_2Cl_2 followed by 2:3 acetone/CH_2Cl_2) the product was obtained as a colourless oil that solidified on standing (0.031 g, 20%). M.p. 112-114 °C. ^{1}H NMR (400 MHz, $CDCl_3$) δ: 9.85 (s, 1H, H_a), 8.71 (m, 2H, H_l), 8.62 (d, J = 5.7 Hz, 1H, H_b), 7.93 (t, J = 1.8 Hz, 1H, H_j), 7.86-7.82 (m, 2H, 2 of H_d, H_e, H_f, H_g, H_h), 7.74-7.65 (4H, H_c, H_i, 2 of H_d, H_e, H_f, H_g, H_h), 7.56-7.52 (m, 3H, H_k, 1 of H_d, H_e, H_f, H_g, H_h). ^{13}C NMR (101 MHz, $CDCl_3$) δ: 151.3, 150.6, 147.5, 144.0, 138.8, 135.9, 132.3, 131.7, 130.4, 129.9, 129.5, 128.1, 127.6, 127.4, 123.9, 121.8, 121.5, 120.7, 95.2, 86.5. HR-ESI-MS *m/z* = 307.1240 $[M+H]^+$ calc. 307.1235.

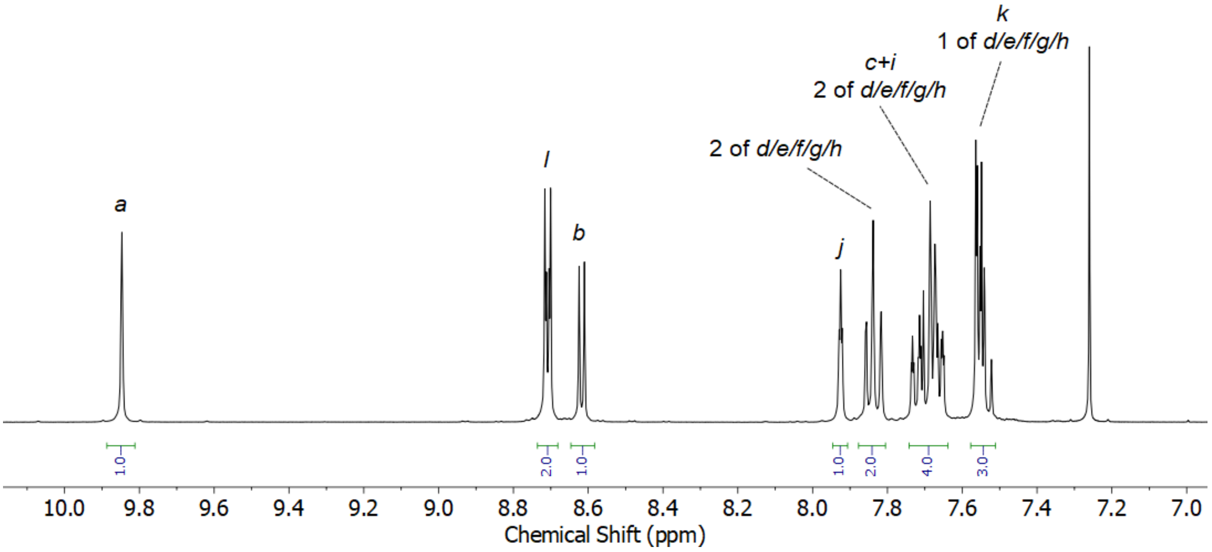

Figure S48 ^{1}H NMR ($CDCl_3$, 400 MHz) of **2**.

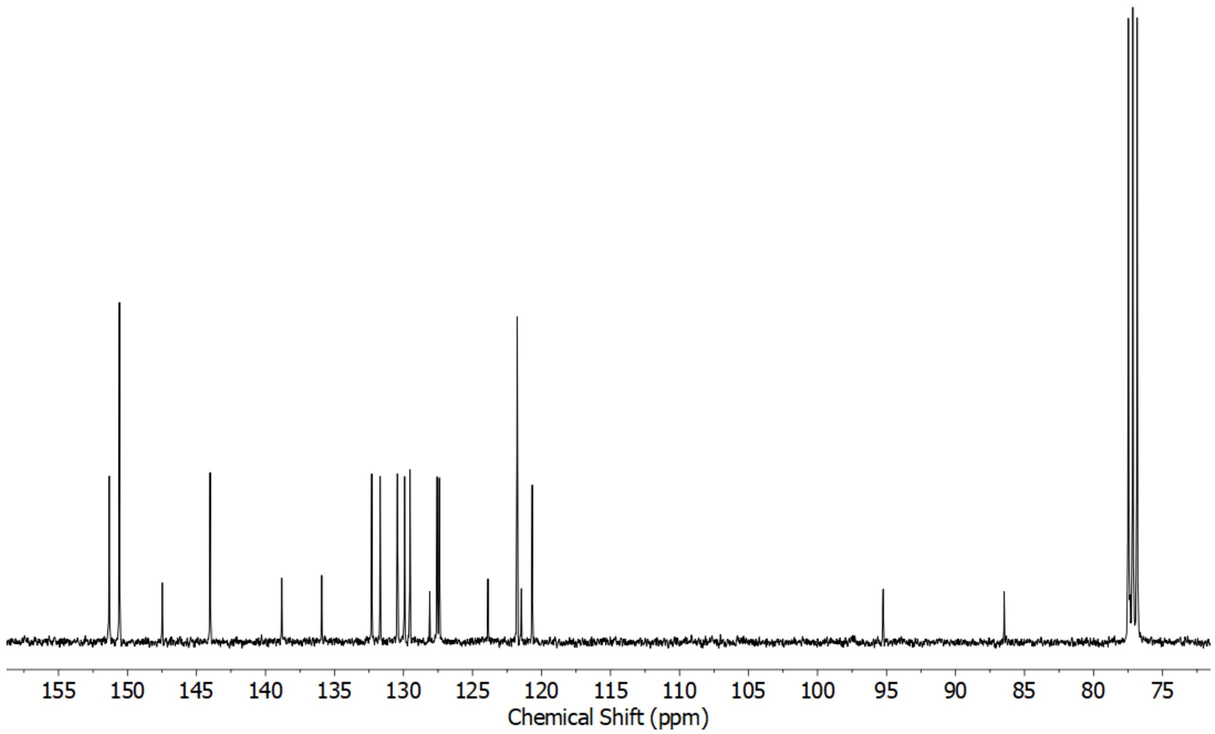

Figure S49 ^{13}C NMR ($CDCl_3$, 101 MHz) of **2**.

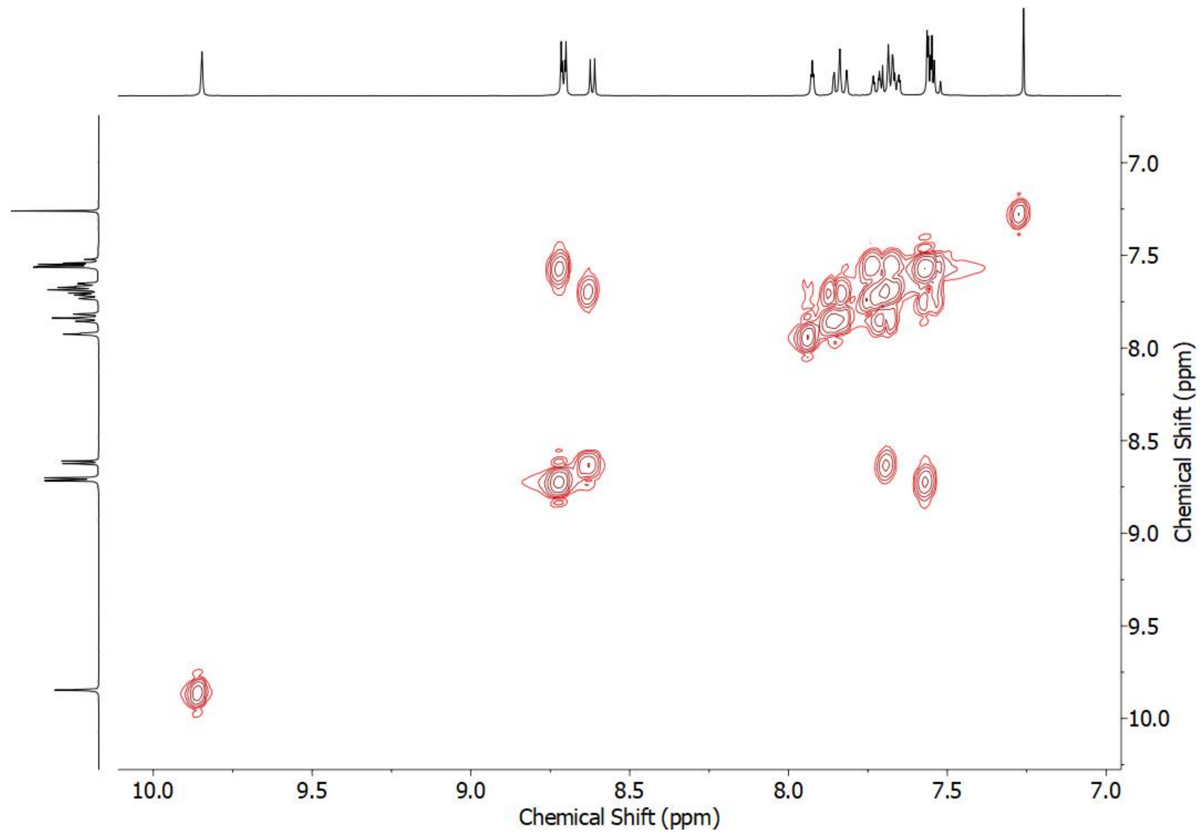

Figure S50 COSY NMR ($CDCl_3$) of **2**.

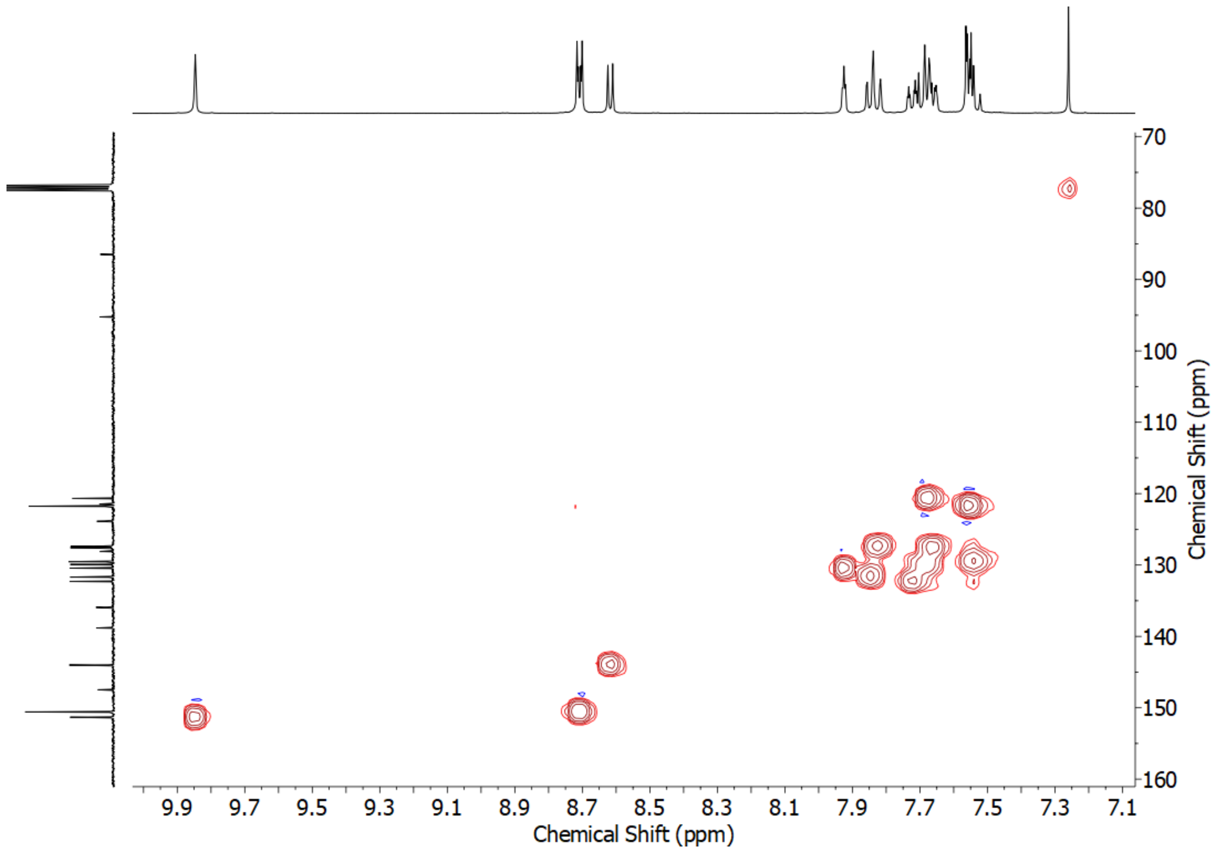

Figure S51 HSQC NMR ($CDCl_3$) of **2**.

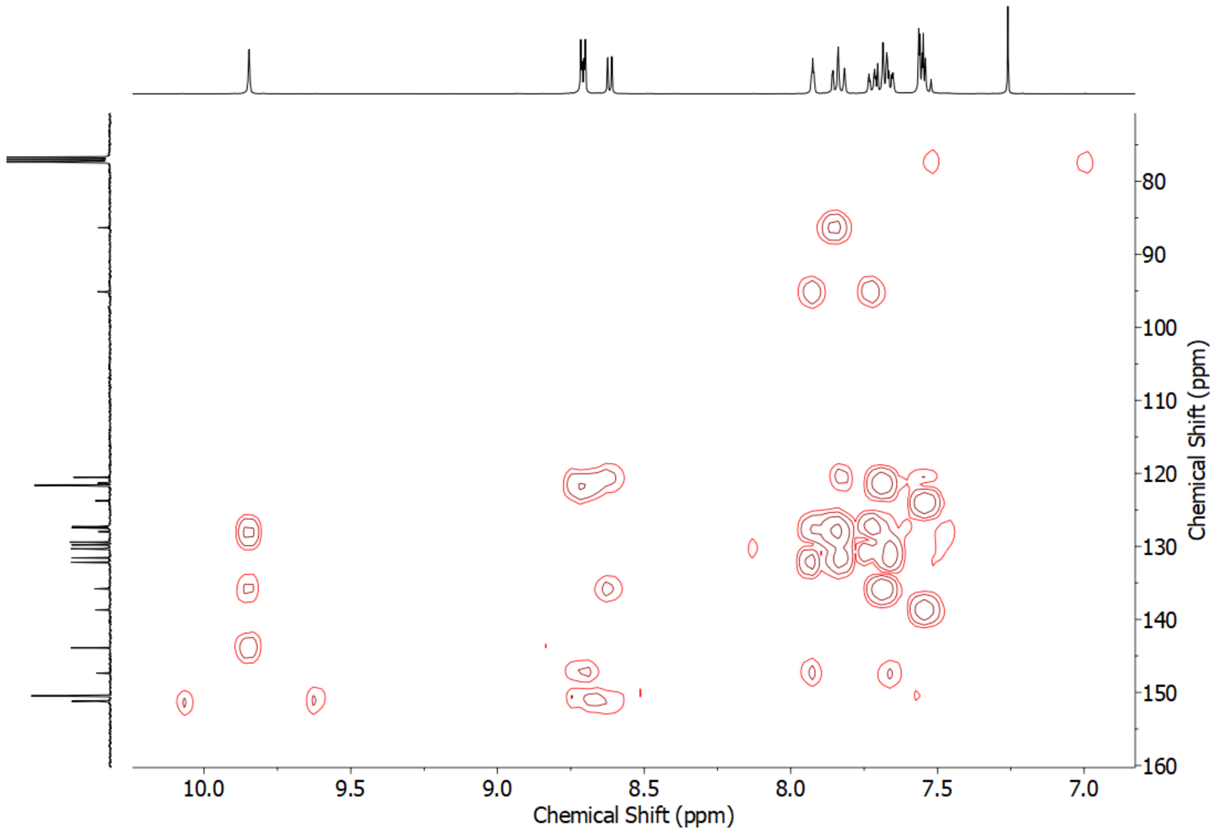

Figure S52 HMBC NMR ($CDCl_3$) of **2**.

3

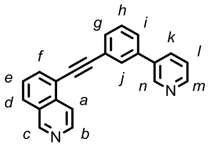

To **S6** (0.126 g, 0.50 mmol, 1 eq.), 5-iodoisoquinoline (0.128 g, 0.50 mmol, 1 eq.), $Pd(PPh_3)_2Cl_2$ (0.0088 g, 0.013 mmol, 2.5 mol%) and CuI (0.0048 g, 0.025 mmol, 5 mol%) in MeCN (5 mL) was added via syringe DBU (0.90 mL, 6.0 mmol, 12 eq.). N_2 was bubbled through the reaction solution for 5 minutes before stirring at rt for 21 h. The solvent was removed *in vacuo* and the residue was dissolved in CH_2Cl_2 (50 mL) before washing with EDTA solution (20 mL), H_2O (2 × 20 mL) and brine (20 mL), dried ($MgSO_4$) and the solvent removed *in vacuo*. After purification by column chromatography on silica (CH_2Cl_2 with a step gradient of 5% increments up to 30% acetone) the product was obtained as a light yellow solid (0.098 g, 64%). M.p. 116-118 °C. ^{1}H NMR (400 MHz, $CDCl_3$) δ: 9.28 (s, 1H, H_c), 8.89 (d, *J* = 1.9 Hz, 1H, H_n), 8.66-8.62 (m, 2H, H_b, H_m), 8.18 (dt, *J* = 5.8, 1.0 Hz, 1H, H_a), 7.98-7.94 (m, 2H, H_f, H_d/H_e), 7.91 (ddd, *J* = 7.9, 2.4, 1.6 Hz, 1H, H_k), 7.85 (t, *J* = 1.5 Hz, 1H, H_j), 7.67 (dt, *J* = 7.6, 1.4 Hz, 1H, H_g), 7.61-7.58 (m, 2H, H_i, H_d/H_e), 7.52 (app. td, *J* = 7.7, 0.6 Hz, 1H, H_h), 7.39 (ddd, *J* = 7.9, 4.8, 0.9 Hz, 1H, H_l). ^{13}C NMR (101 MHz, $CDCl_3$) δ: 152.9, 149.1, 148.4, 144.2, 138.5, 136.1, 135.8, 134.5, 134.3, 131.4, 130.5, 129.4, 128.5, 128.3, 127.6, 126.9, 123.9, 123.7, 120.2, 118.9, 95.0, 86.6. HR-ESI-MS *m/z* = 307.1241 $[M+H]^+$ calc. 307.1235.

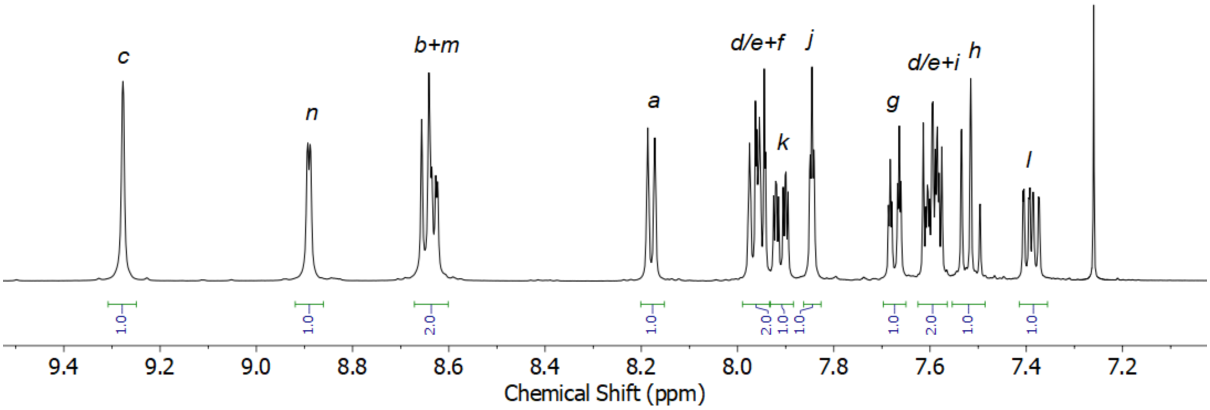

Figure S53 ^{1}H NMR ($CDCl_3$, 400 MHz) of **3**.

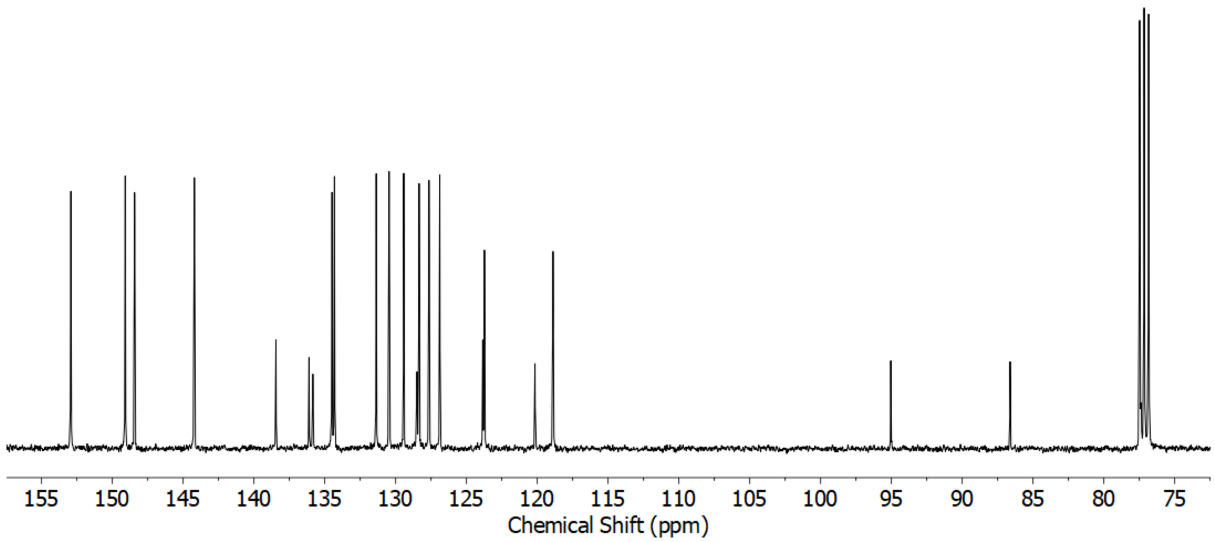

Figure S54 ^{13}C NMR ($CDCl_3$, 101 MHz) of **3**.

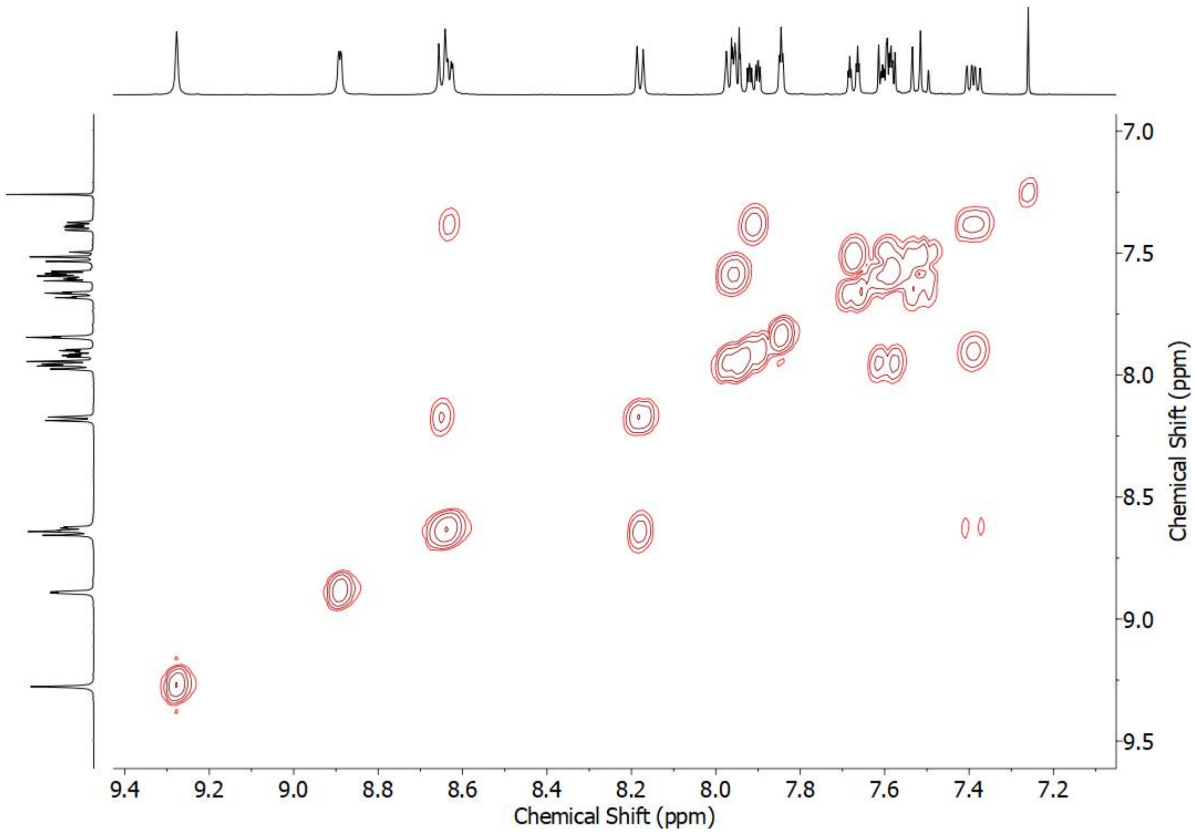

Figure S55 COSY NMR ($CDCl_3$) of **3**.

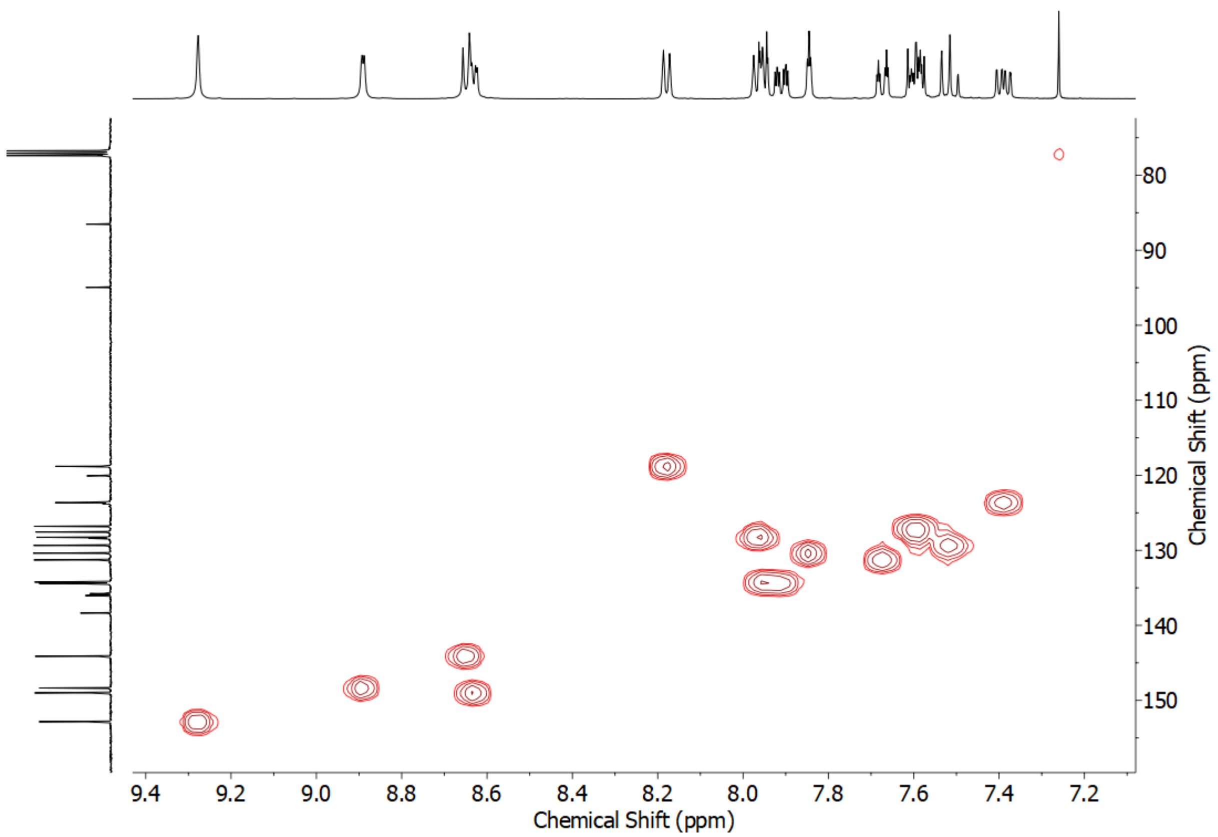

Figure S56 HSQC NMR ($CDCl_3$) of **3**.

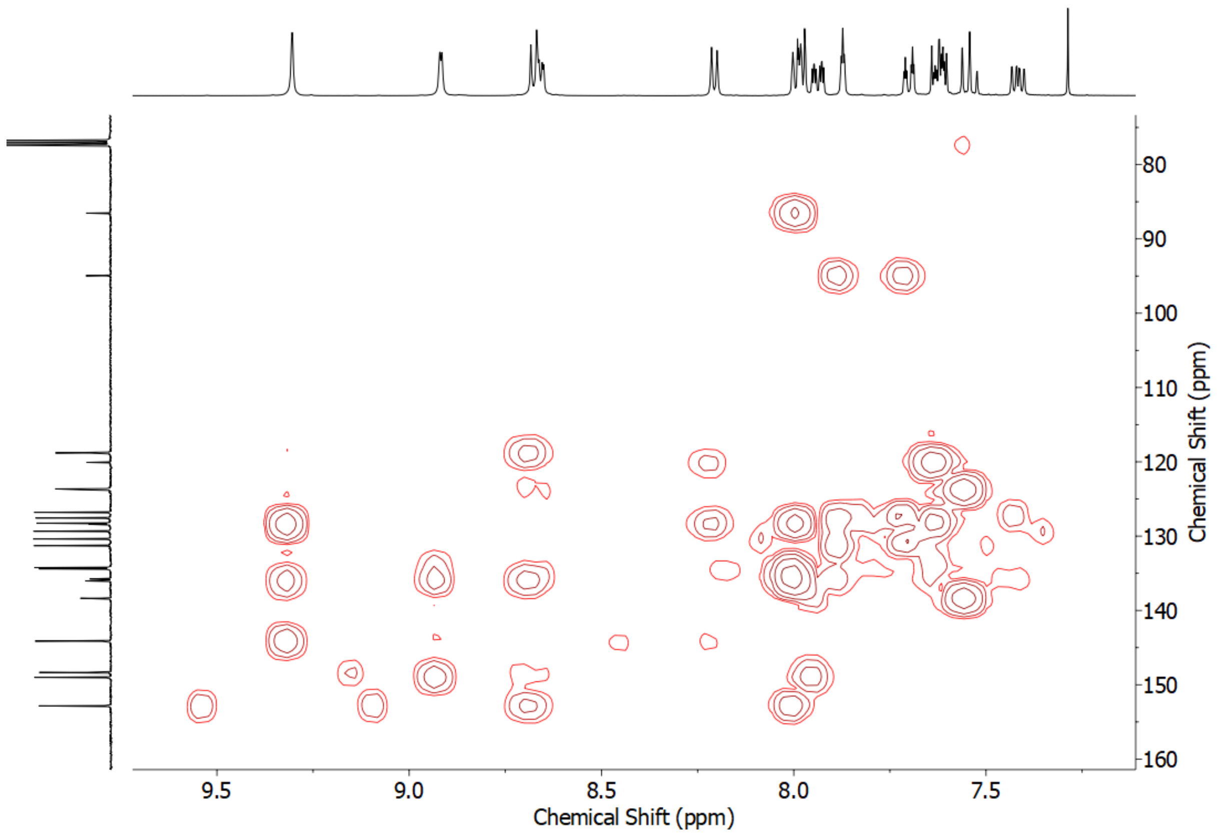

Figure S57 HMBC NMR ($CDCl_3$) of **3**.

4

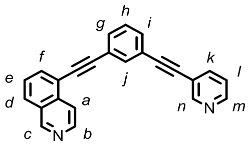

S8 (0.080 g, 0.23 mmol, 1 eq.), 3-ethynylpyridine (0.028 g, 0.27 mmol, 1.2 eq.), $Pd(PPh_3)_2Cl_2$ (0.0079 g, 0.011 mmol, 5 mol%) and CuI (0.0043 g, 0.023 mmol, 10 mol%) were stirred at rt in $^iPr_2NH$ (5 mL) for 22 h. EDTA solution (10 mL) was added and the aqueous phase extracted with CH_2Cl_2 (3 × 10 mL). The combined organic phases were dried ($MgSO_4$) and the solvent removed *in vacuo*. After purification by column chromatography on silica (gradient of 1:9 to 1:3 acetone/CH_2Cl_2) the product was obtained as a yellow solid (0.069 g, quant.). M.p. 136-138 °C. ^1H NMR (400 MHz, $CDCl_3$) δ:9.29 (d, *J* = 1.0 Hz, 1H, H_c), 8.79 (dd, *J* = 2.1, 0.9 Hz, 1H, H_n), 8.66 (d, *J* = 5.8 Hz, 1H, H_b), 8.57 (dd, *J* = 4.9, 1.7 Hz, 1H, H_m), 8.17 (d, *J* = 5.9 Hz, 1H, H_a), 7.98 (d, *J* = 8.2 Hz, 1H, H_d), 7.95 (dd, *J* = 7.2, 1.1 Hz, 1H, H_f), 7.84-7.82 (m, 2H, H_j, H_k), 7.65-7.55 (m, 3H, H_e, H_g, H_i), 7.42 (app. td, *J* = 7.8, 0.6 Hz, 1H, H_h), 7.31 (ddd, *J* = 7.9, 4.9, 0.9 Hz, 1H, H_l). ^{13}C NMR (101 MHz, $CDCl_3$) δ: 153.0, 152.5, 149.0, 144.3, 138.6, 136.2, 134.9, 134.4, 132.0, 132.0, 128.9, 128.5, 128.4, 126.9, 123.5, 123.3, 123.2, 120.3, 120.1, 118.9, 94.5, 91.7, 86.9, 86.8. HR-ESI-MS *m/z* = 331.1241 $[M+H]^+$ calc. 331.1235.

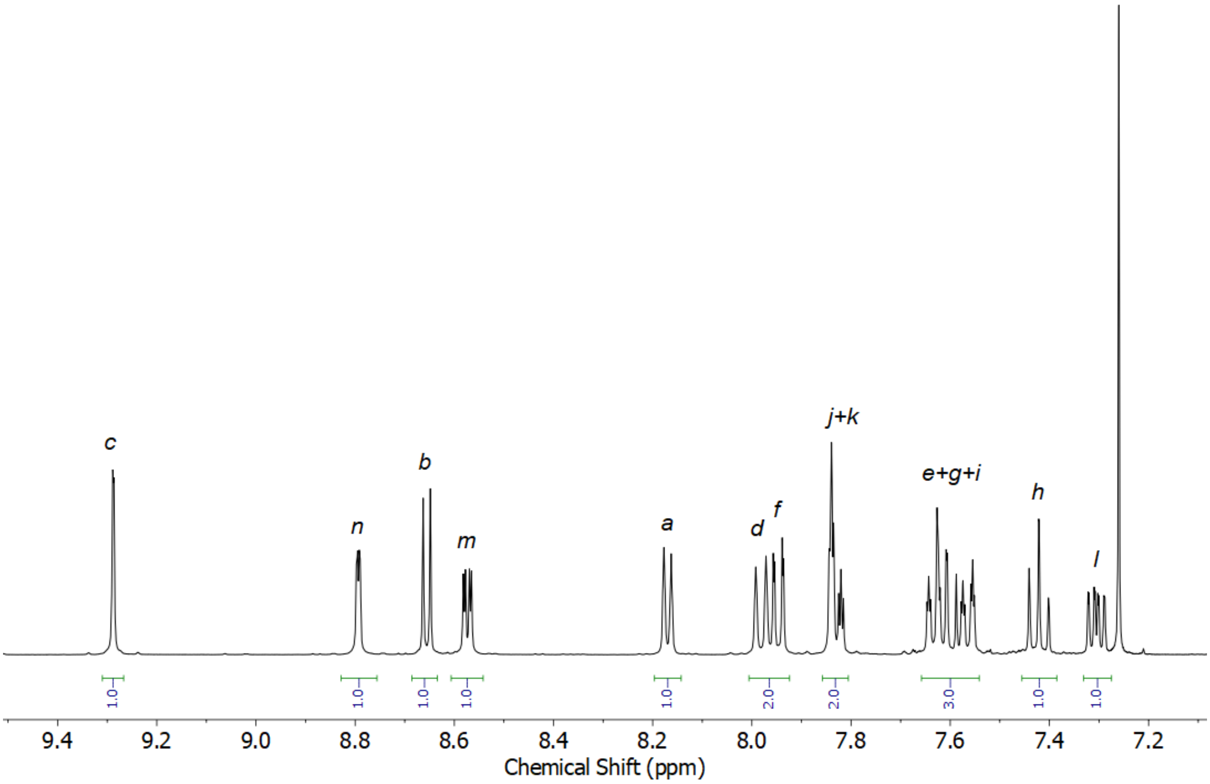

Figure S58 ^1H NMR ($CDCl_3$, 400 MHz) of **4**.

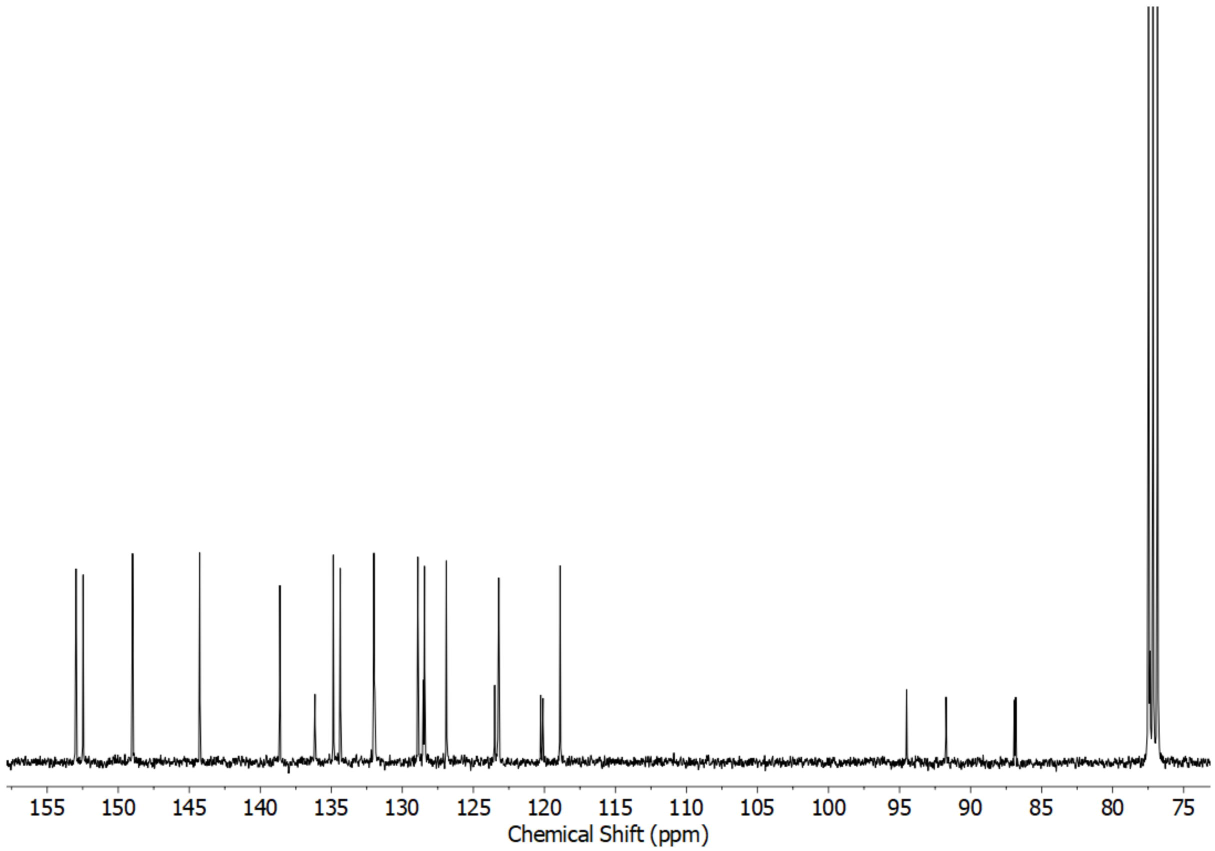

Figure S59 ^{13}C NMR ($CDCl_3$, 101 MHz) of **4**.

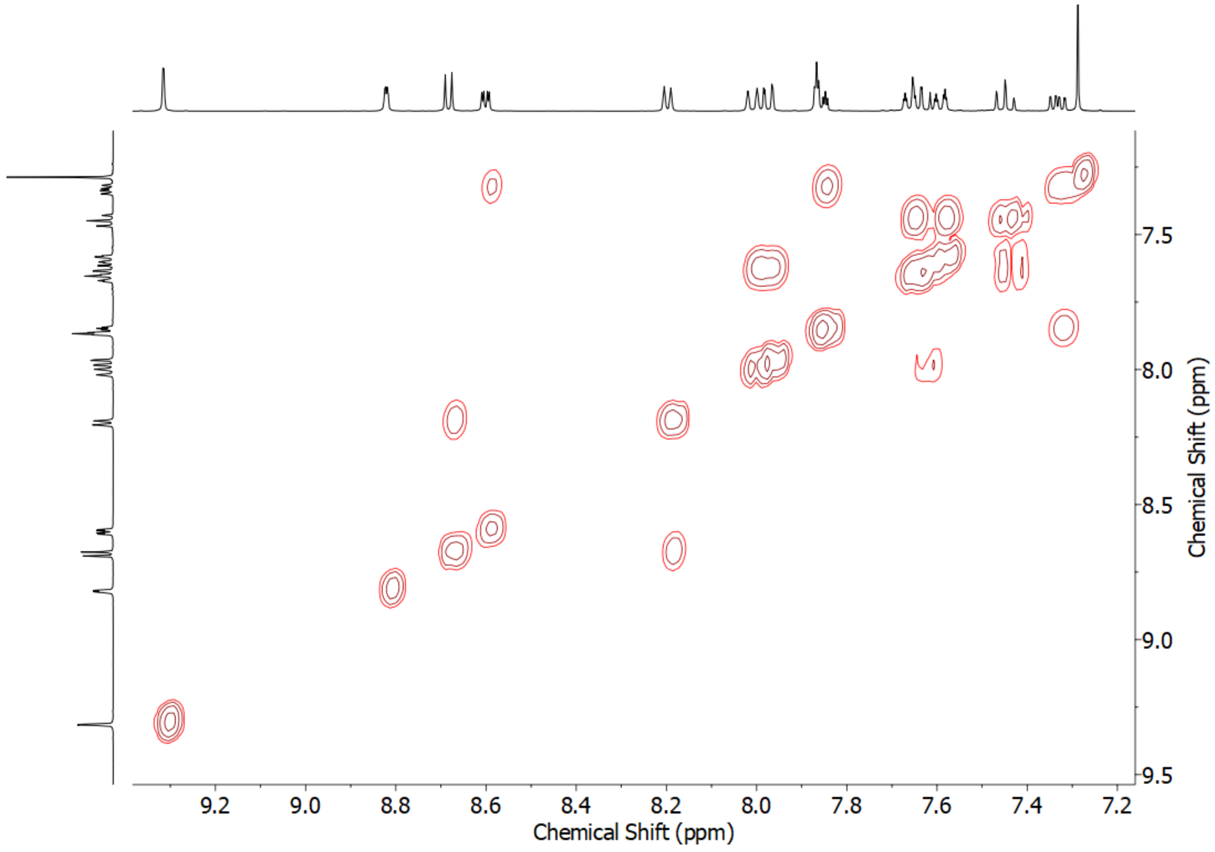

Figure S60 COSY NMR ($CDCl_3$) of **4**.

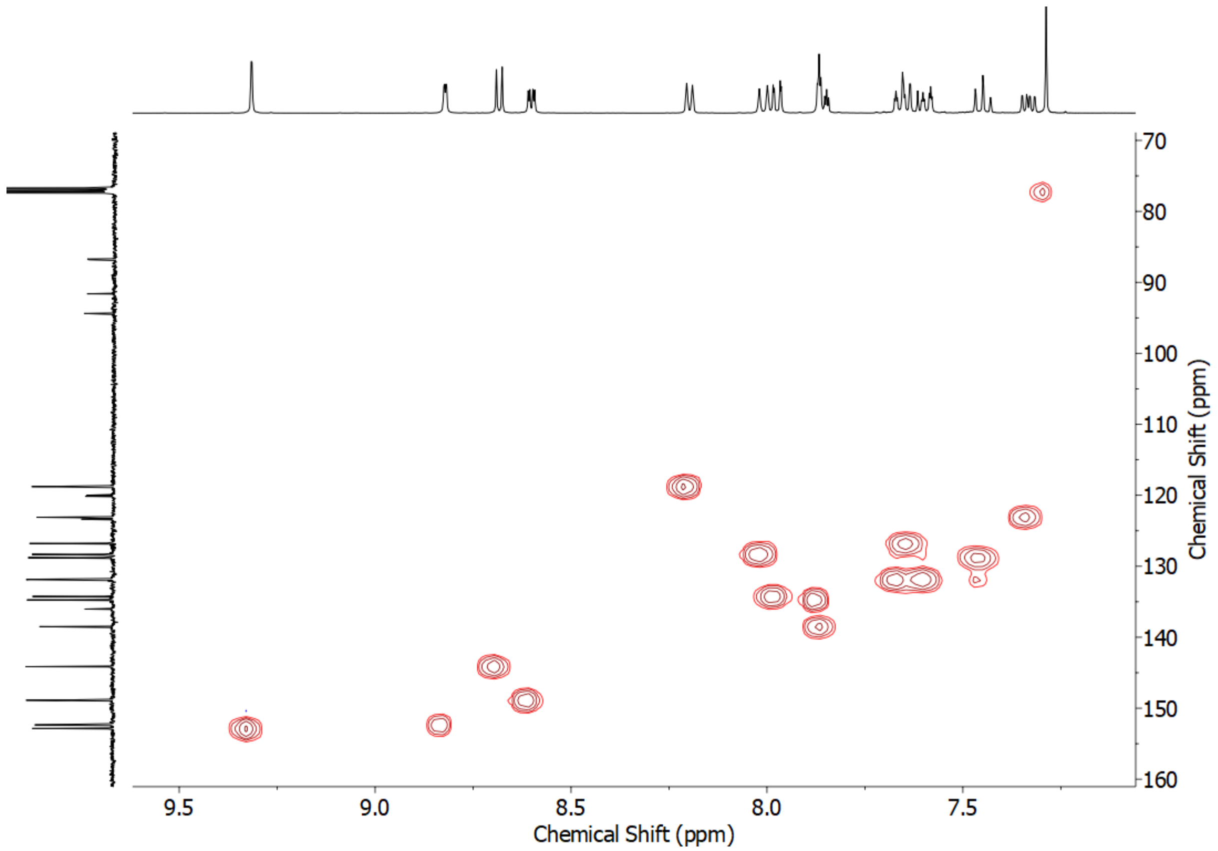

Figure S61 HSQC NMR ($CDCl_3$) of **4**.

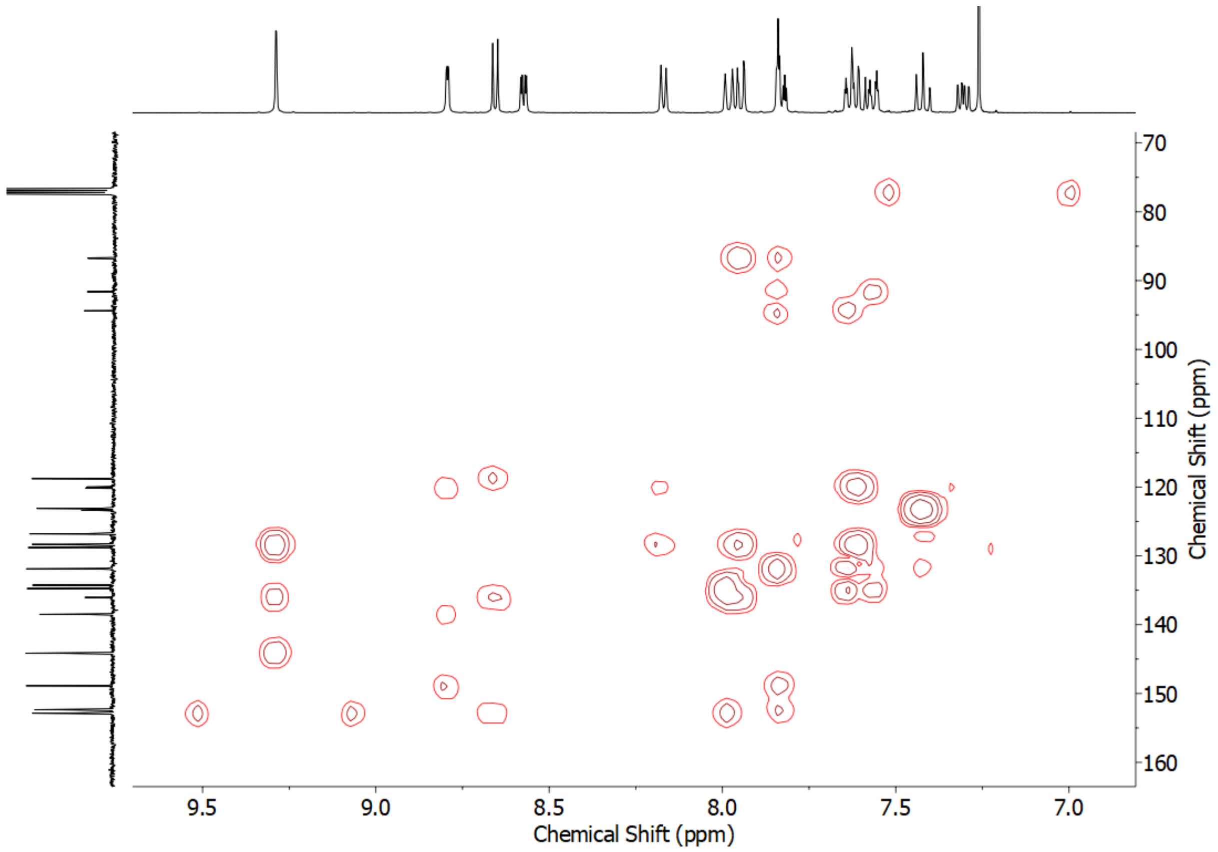

Figure S62 HMBC NMR ($CDCl_3$) of **4**.

5

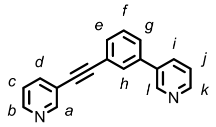

S5 (0.117 g, 0.50 mmol, 1 eq.), 3-ethynylpyridine (0.062 g, 0.60 mmol, 1.2 eq), $Pd(PPh_3)_2Cl_2$ (0.0088 g, 0.013 mmol, 2.5 mol%) and CuI (0.0048 g, 0.025 mmol, 5 mol%) were stirred at 80 °C in ${}^{i}Pr_2NH$ (5 mL) in a sealed vial for 17 h. EDTA solution (20 mL) was added and the aqueous phase extracted with CH_2Cl_2 (3 × 20 mL). The combined organic phases were dried ($MgSO_4$) and the solvent removed *in vacuo*. After purification by column chromatography on silica (CH_2Cl_2 with a step gradient of 5% increments up to 25% acetone) the product was obtained as a light yellow oil (0.083 g, 65%). ^1H NMR (400 MHz, $CDCl_3$) δ: 8.86 (dd, *J* = 2.4, 0.9 Hz, 1H, H_l), 8.79 (dd, *J* = 2.1, 0.9 Hz, 1H, H_a), 8.62 (dd, *J* = 4.8, 1.6 Hz, 1H, H_k), 8.56 (dd, *J* = 4.9, 1.7 Hz, 1H, H_b), 7.89 (ddd, *J* = 7.9, 2.4, 1.7 Hz, 1H, H_i), 7.83 (app. dt, *J* = 7.9, 1.9 Hz, 1H, H_d), 7.77 (t, *J* = 1.5 Hz, 1H, H_h), 7.59-7.57 (m, 2H, H_e, H_g), 7.51-7.47 (m, 1H, H_f), 7.38 (ddd, *J* = 7.9, 4.8, 0.9 Hz, 1H, H_j), 7.30 (ddd, *J* = 7.9, 4.9, 0.9 Hz, 1H, H_c). ^{13}C NMR (101 MHz, $CDCl_3$) δ: 152.4, 149.1, 148.9, 148.4, 138.6, 138.4, 135.8, 134.5, 131.3, 130.5, 129.4, 127.7, 123.7, 123.6, 123.2, 120.3, 92.3, 86.7. HR-ESI-MS *m/z* = 257.1070 $[M+H]^+$ calc. 257.1079.

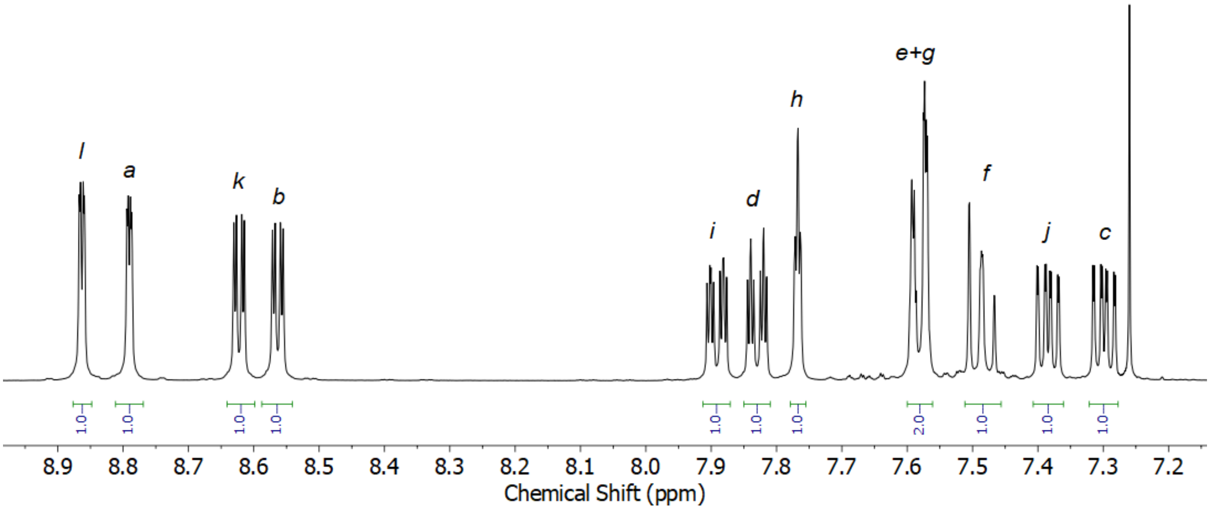

Figure S63 ^1H NMR ($CDCl_3$, 400 MHz) of **5**.

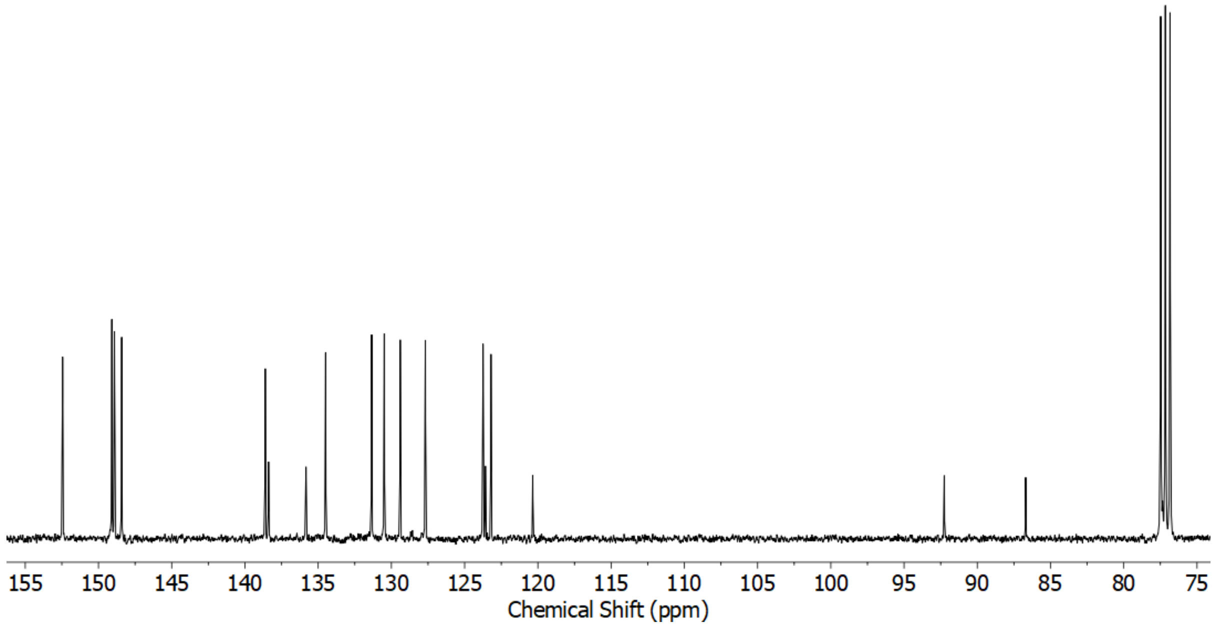

Figure S64 ^{13}C NMR ($CDCl_3$, 101 MHz) of **5**.

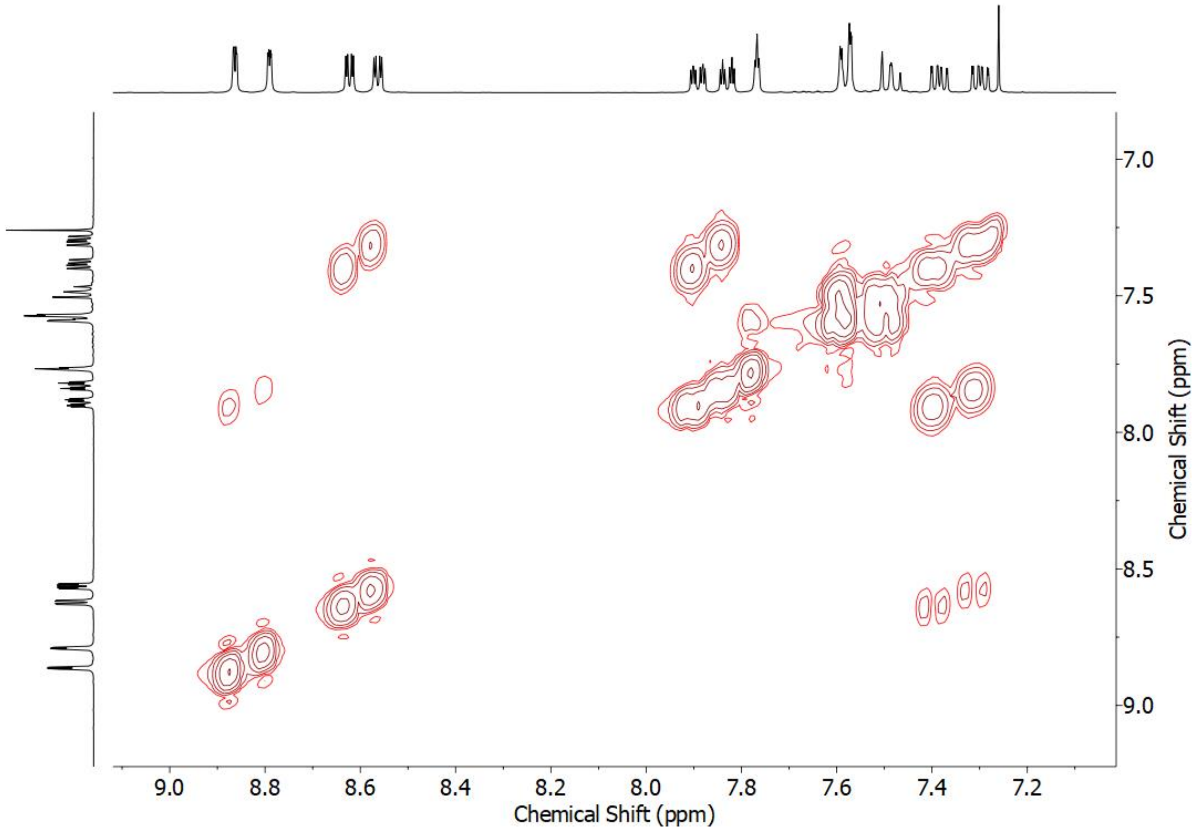

Figure S65 COSY NMR ($CDCl_3$) of **5**.

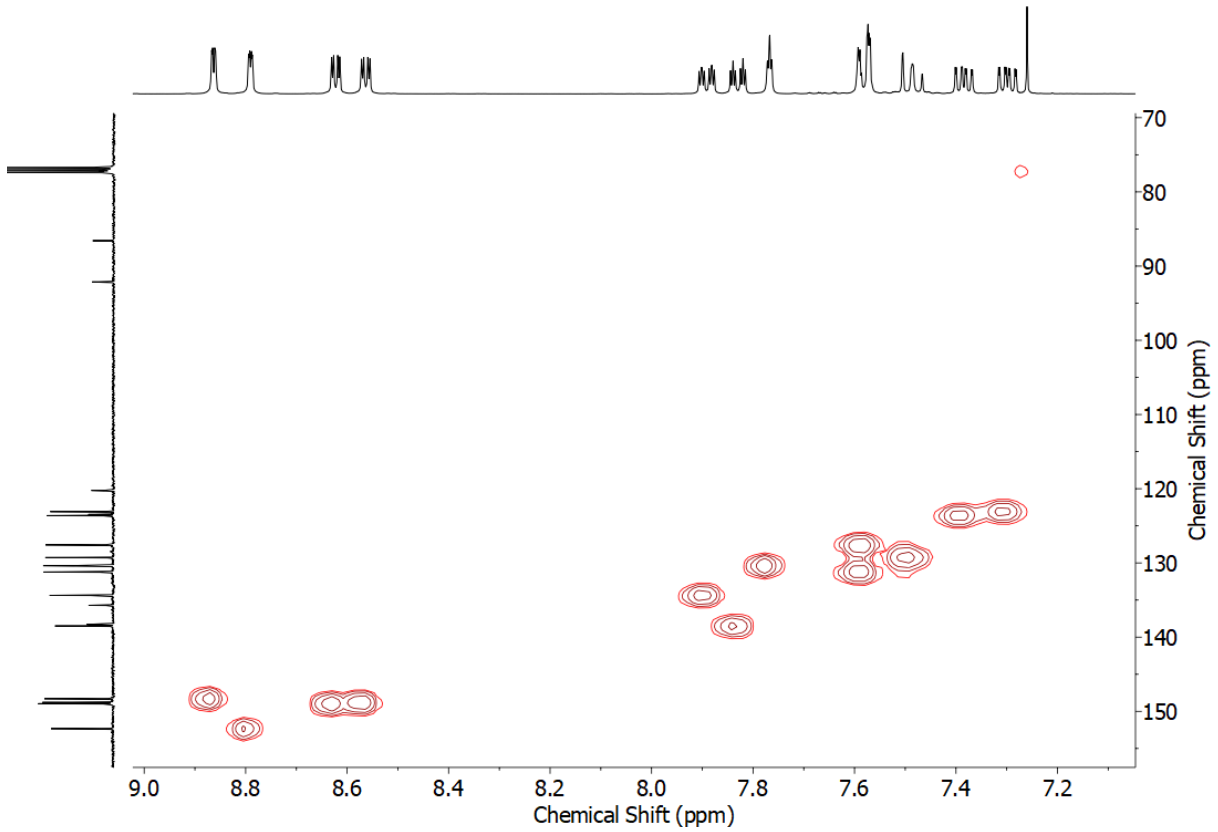

Figure S66 HSQC NMR ($CDCl_3$) of **5**.

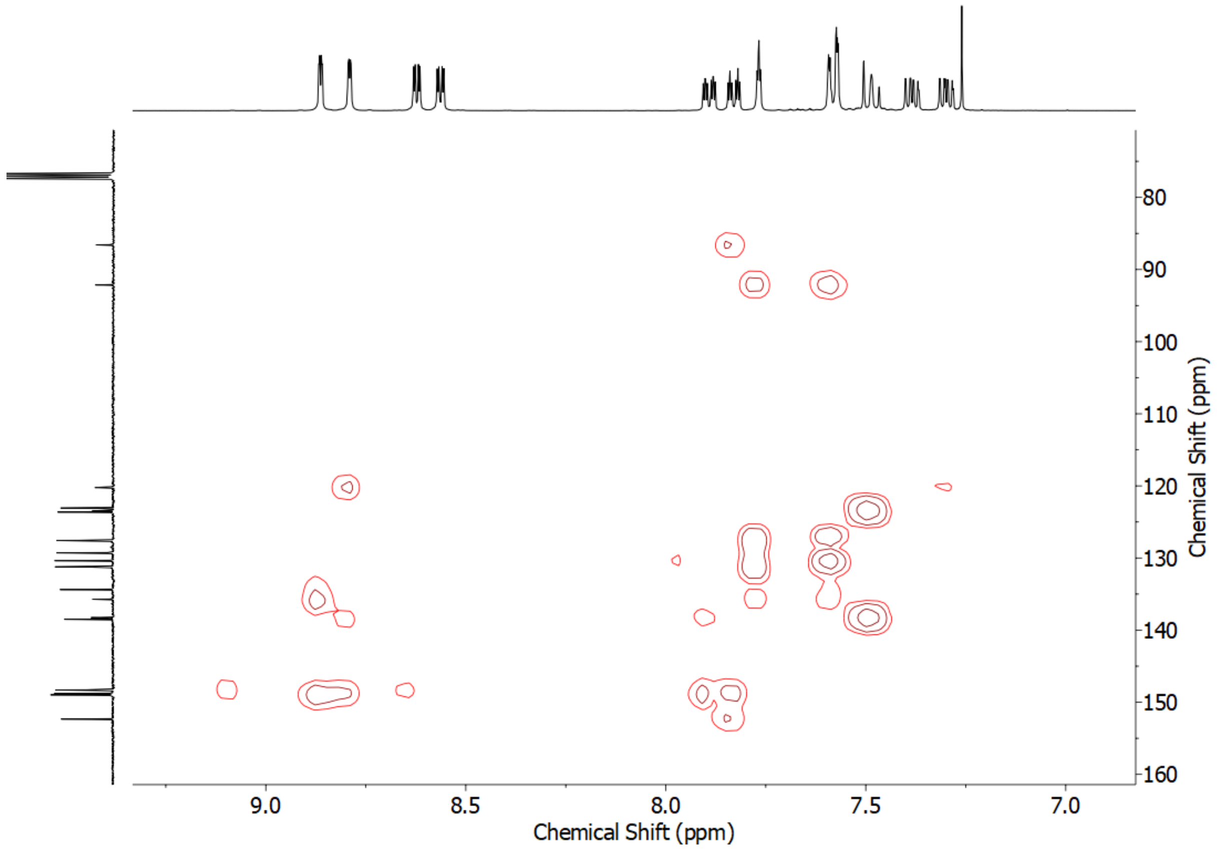

Figure S67 HMBC NMR ($CDCl_3$) of **5**.

6

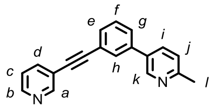

S9 (0.248 g, 1.0 mmol, 1 eq.), 3-ethynylpyridine (0.124 g, 1.2 mmol, 1.2 eq.), $Pd(PPh_3)_2Cl_2$ (0.018 g, 0.025 mmol, 2.5 mol%) and CuI (0.010 g, 0.050 mmol, 5 mol%) were stirred at 80 °C in $^iPr_2NH$ (10 mL) in a sealed vial for 24 h. EDTA solution (20 mL) was added and the aqueous phase extracted with CH_2Cl_2 (3 × 20mL). The combined organic phases were dried ($MgSO_4$) and the solvent removed *in vacuo*. After purification by column chromatography on silica (CH_2Cl_2 with a step gradient of 5% increments up to 25% acetone) the product was obtained as a light brown solid (0.170 g, 63%). M.p. 90-92 °C. ^{1}H NMR (400 MHz, $CDCl_3$) δ: 8.79 (dd, *J* = 2.1, 0.8 Hz, 1H, H_a), 8.74 (dd, *J* = 2.4, 0.9 Hz, 1H, H_k), 8.56 (dd, *J* = 4.9, 1.7 Hz, 1H, H_b), 7.83 (dt, *J* = 7.9, 1.9 Hz, 1H, H_d), 7.79 (dd, *J* = 8.0, 2.4 Hz, 1H, H_i), 7.74 (td, *J* = 1.8, 0.6 Hz, 1H, H_h), 7.58-7.55 (m, 2H, H_e, H_g), 7.47 (ddd, *J* = 8.0, 7.3, 0.6 Hz, 1H, H_f), 7.30 (ddd, *J* = 7.9, 4.9, 0.9 Hz, 1H, H_c), 7.24 (d, *J* = 8.0 Hz, 1H, H_j), 2.61 (s, 3H, H_l). ^{13}C NMR (101 MHz, $CDCl_3$) δ: 157.9, 152.5, 148.9, 147.6, 138.6, 138.5, 134.8, 132.9, 131.1, 130.3, 129.3, 127.5, 123.5, 123.4, 123.2, 120.4, 92.4, 86.6, 24.3. HR-ESI-MS *m/z* = 271.1230 [M+H]$^+$ calc. 271.1235.

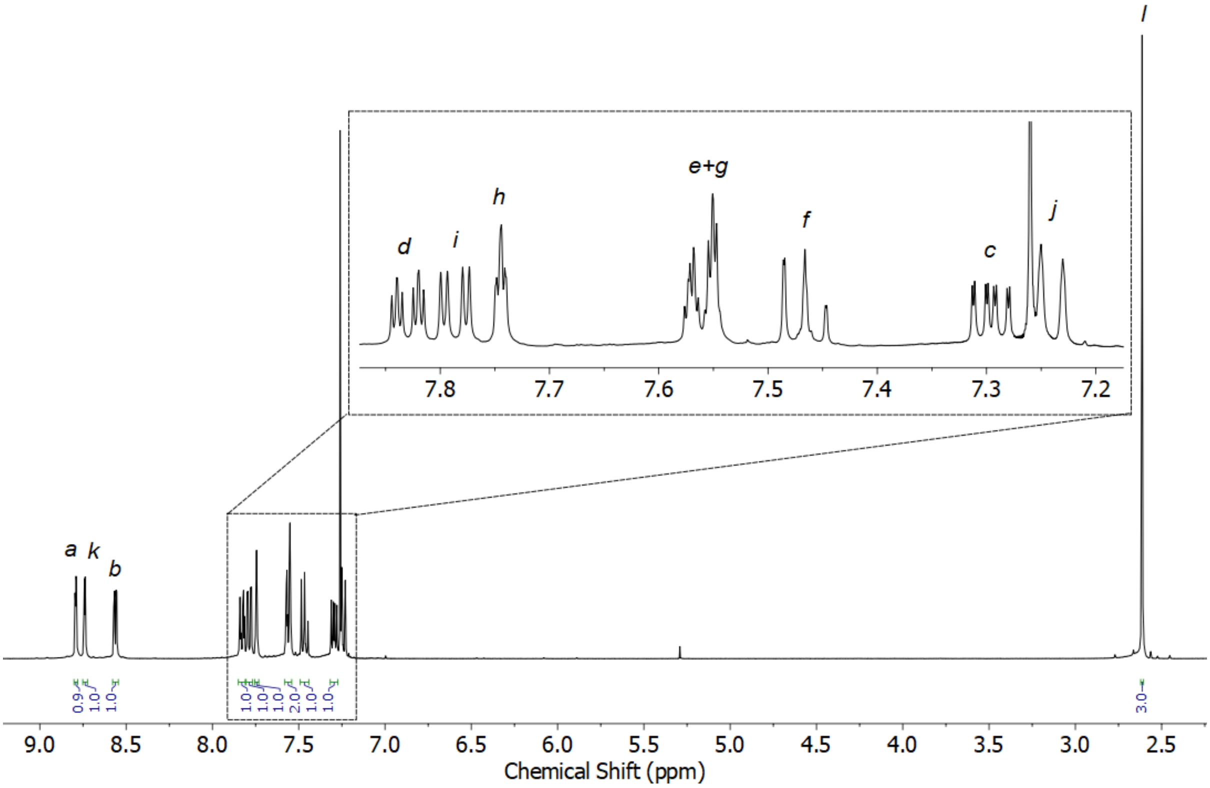

Figure S68 ^{1}H NMR ($CDCl_3$, 400 MHz) of **6**.

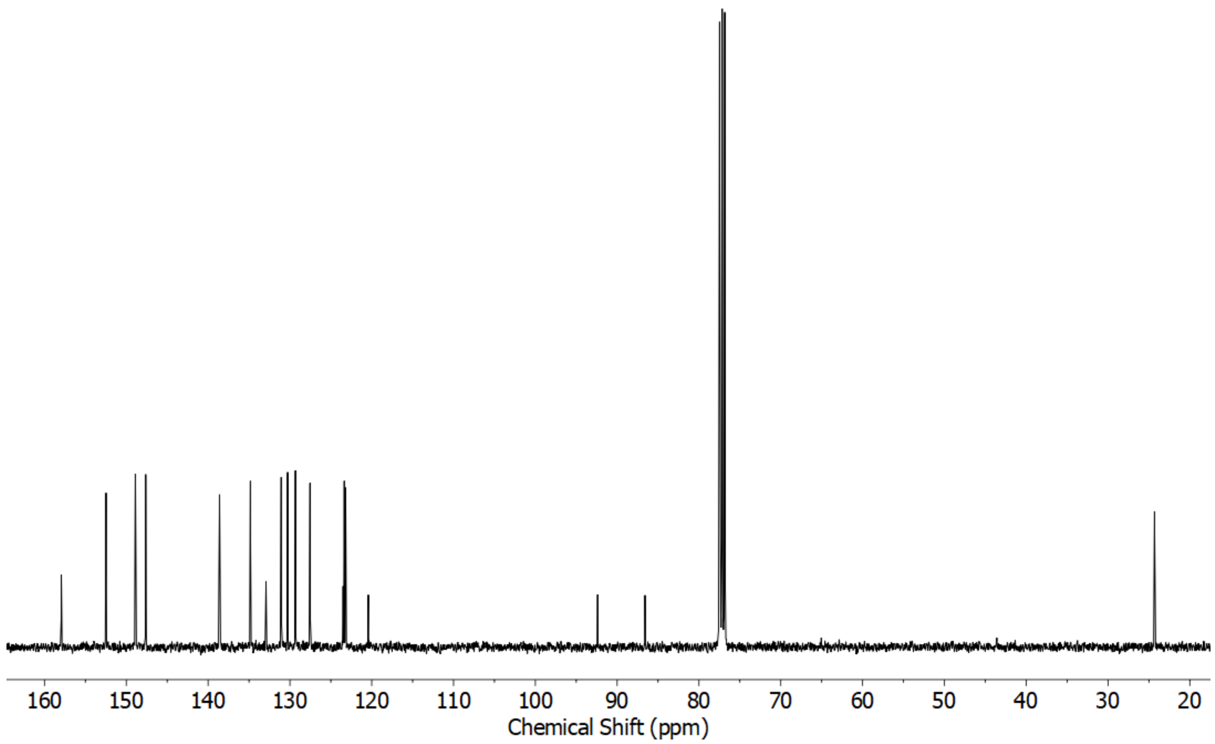

Figure S69 ^{13}C NMR ($CDCl_3$, 101 MHz) of **6**.

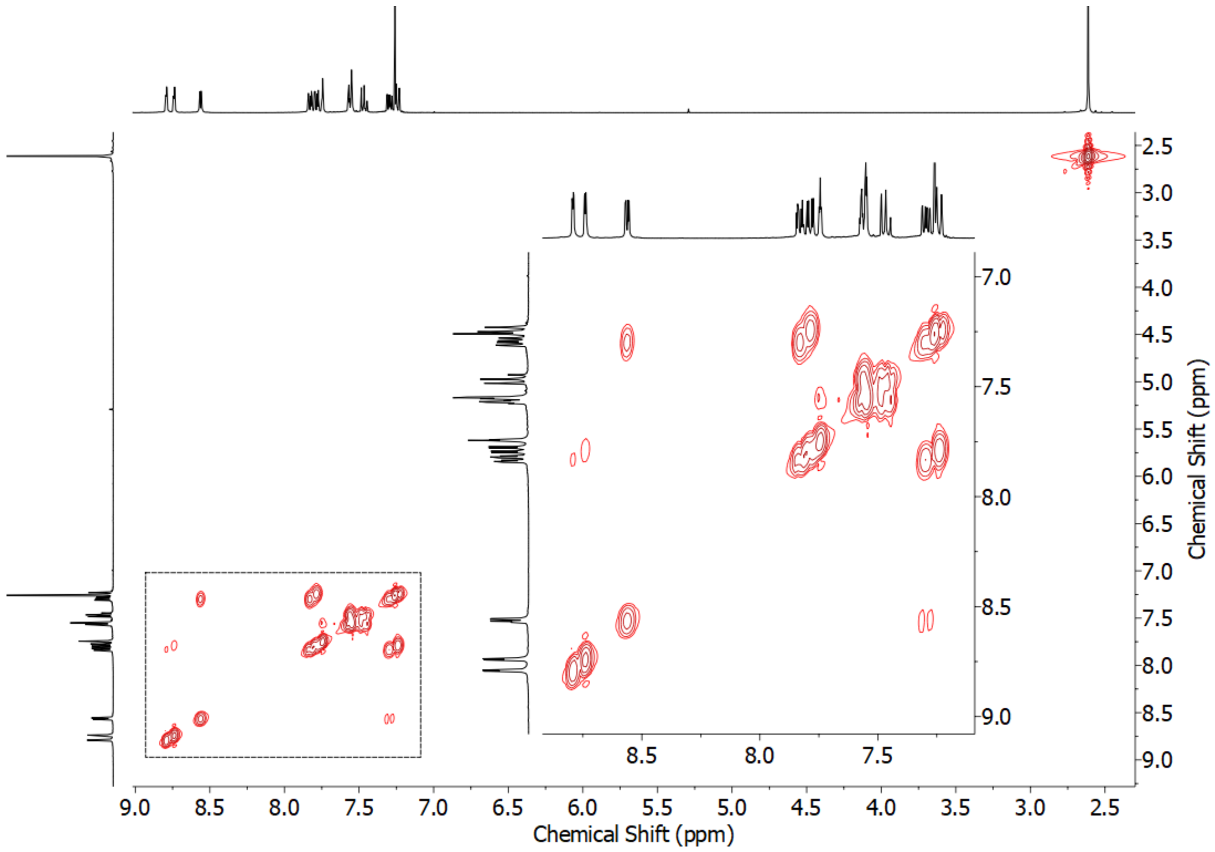

Figure S70 COSY NMR ($CDCl_3$) of **6**.

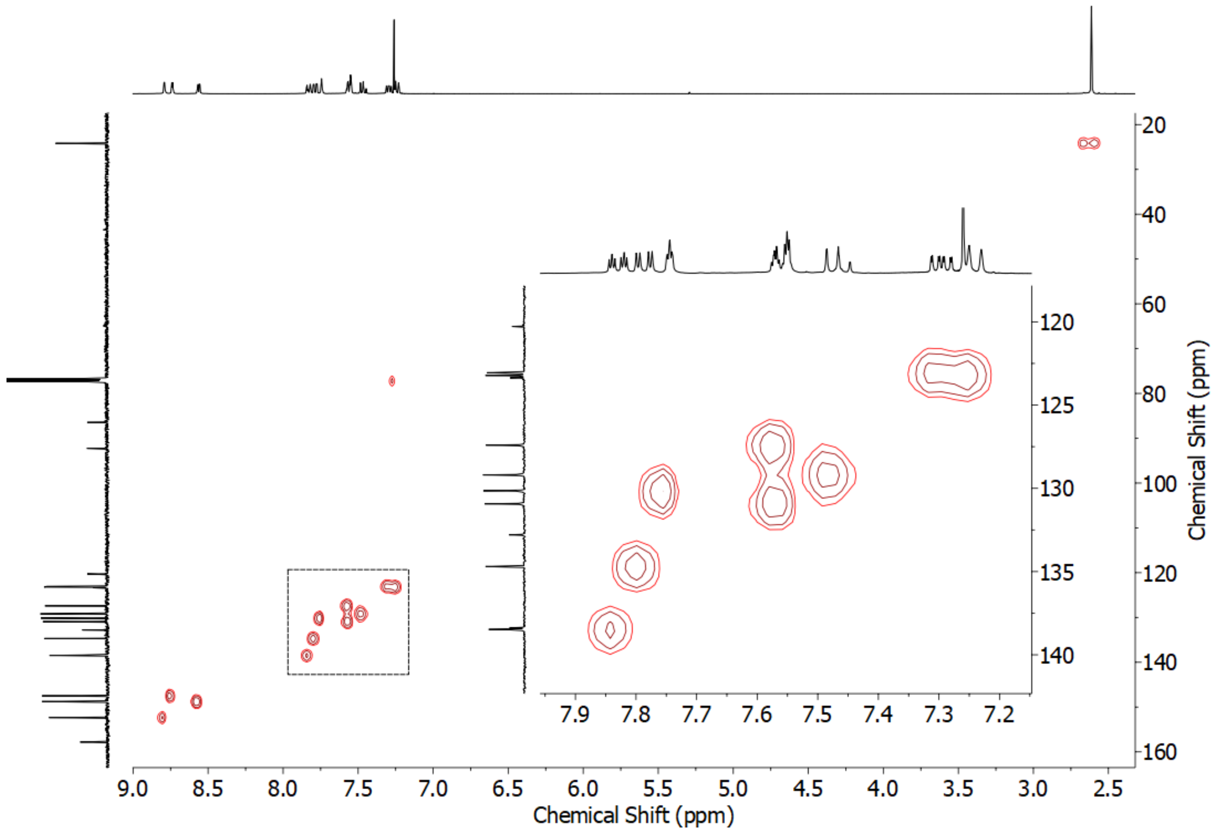

Figure S71 HSQC NMR ($CDCl_3$) of **6**.

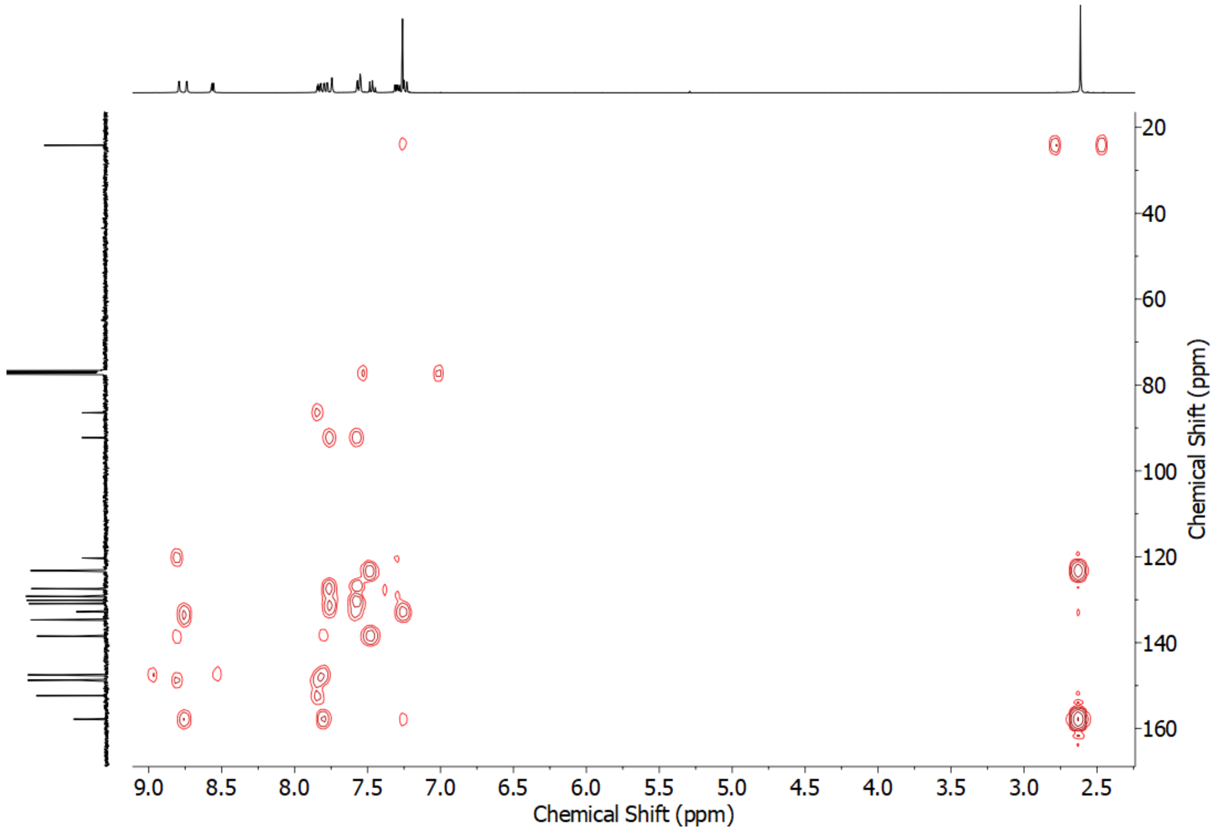

Figure S72 HMBC NMR ($CDCl_3$) of **6**.

[Pd$_2$(1)$_4$](BF$_4$)$_4$

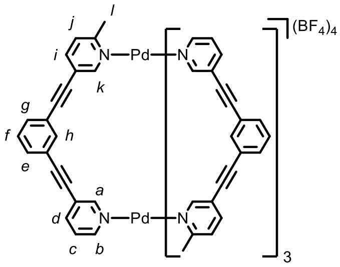

1 (8.8 mg, 0.030 mmol, 2 eq.) and [Pd(CH$_3$CN)$_4$](BF$_4$)$_2$ (6.7 mg, 0.015 mmol, 1 eq.) were sonicated in d_6-DMSO (0.75 mL) until all solids were dissolved. After standing at rt for 24 h, the formation of two major species could be observed by ^{1}H NMR. Diffusion coefficient (500 MHz, d_6-DMSO) *D*: 1.12 × 10^{-10} m^2s^{-1}. ESI-MS *m/z* = 781.13 {[Pd$_2$(C$_{21}$H$_{14}$N$_2$)$_4$](BF$_4$)$_2$}$^{2+}$ calc. 781.14.

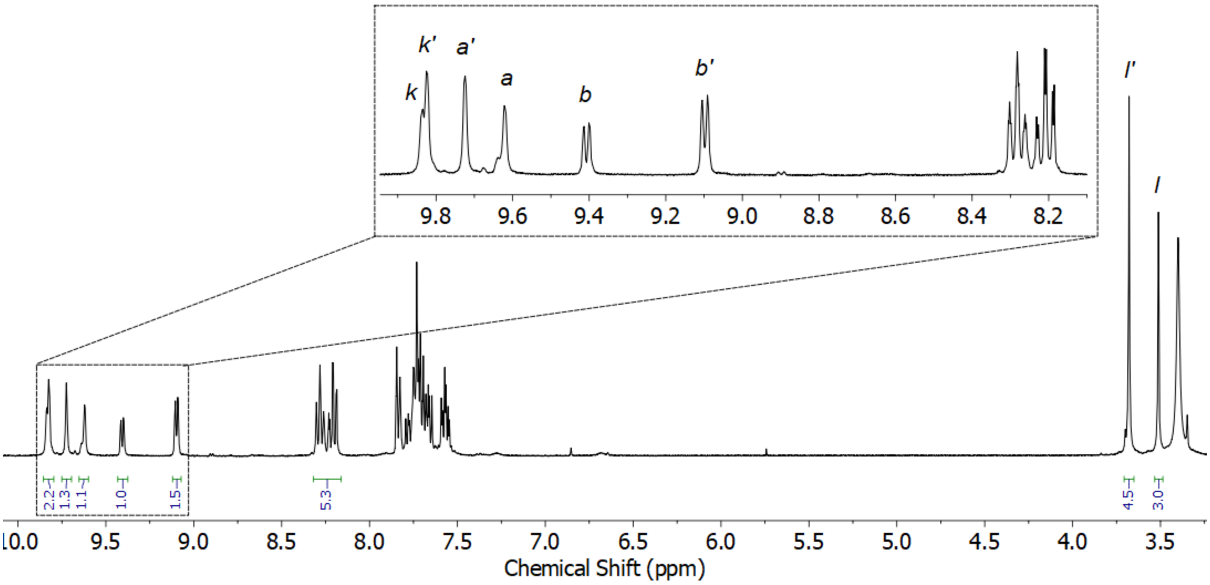

Figure S73 ^{1}H NMR (d_6-DMSO, 500 MHz) of [Pd$_2$(**1**)$_4$](BF$_4$)$_4$.

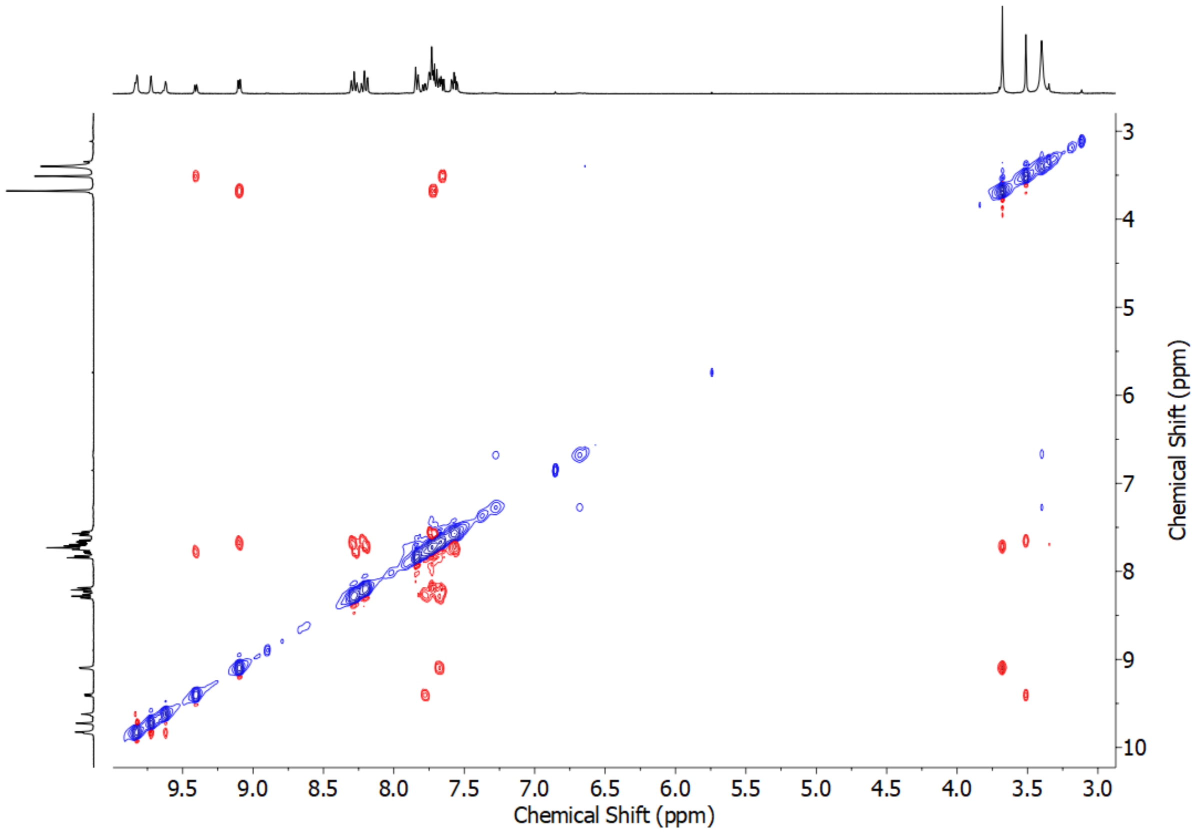

Figure S74 ROESY NMR (d_6-DMSO, 500 MHz) of $[Pd_2(\mathbf{1})_4](BF_4)_4$.

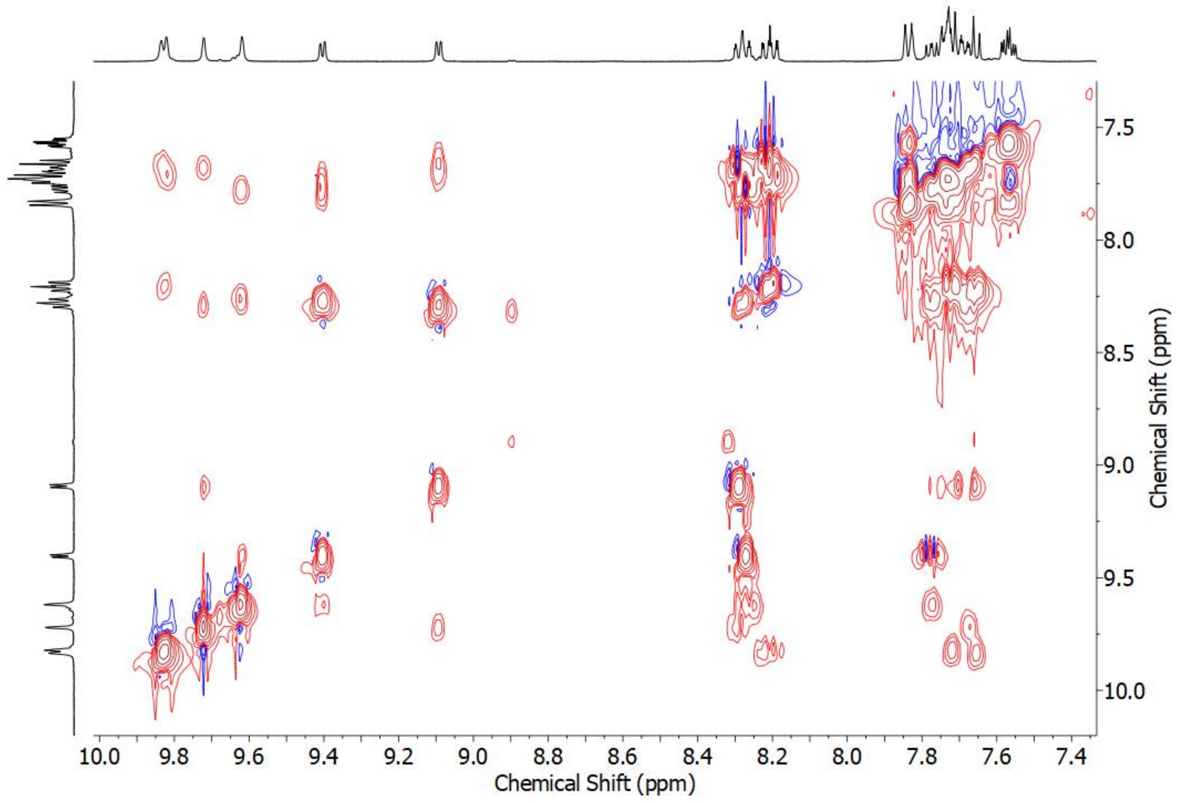

Figure S75 Partial 2D TOCSY NMR (d_6-DMSO, 500 MHz) of $[Pd_2(\mathbf{1})_4](BF_4)_4$.

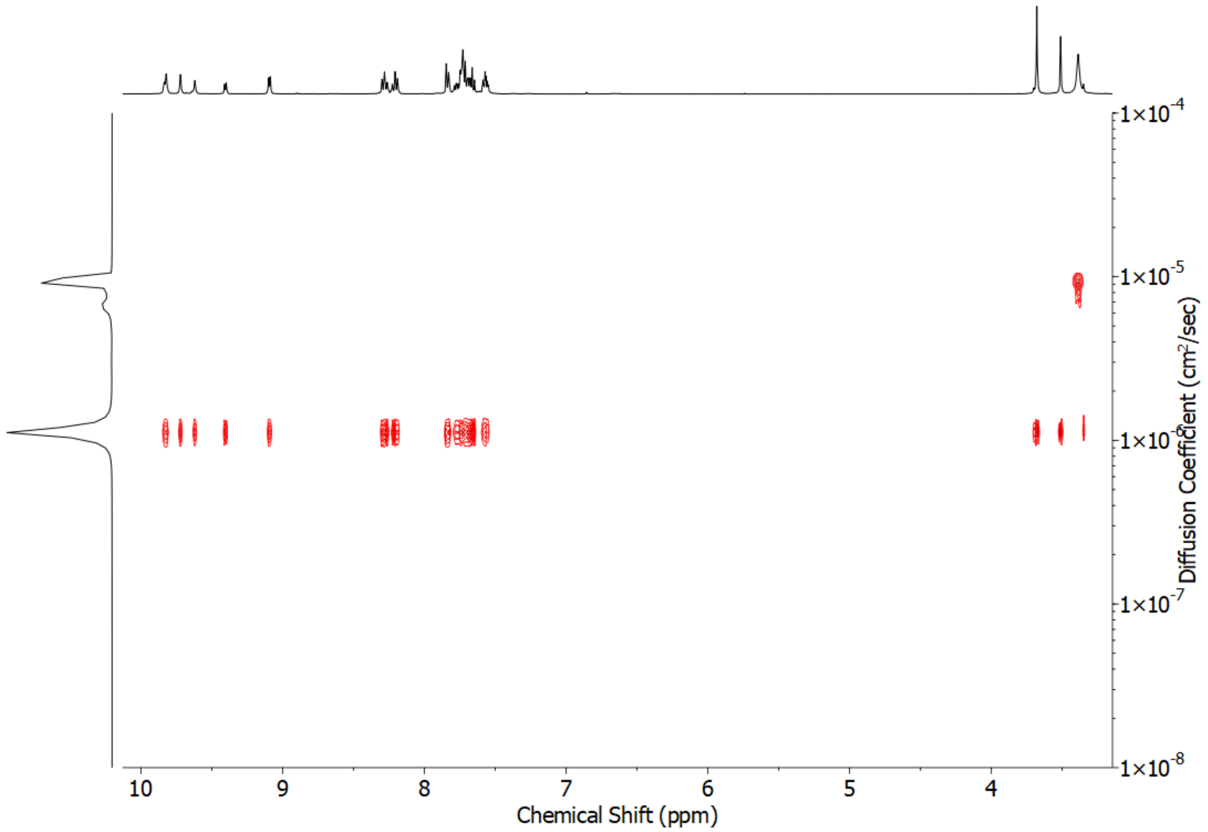

Figure S76 DOSY NMR (d_6-DMSO, 500 MHz) of $[Pd_2(\mathbf{1})_4](BF_4)_4$.

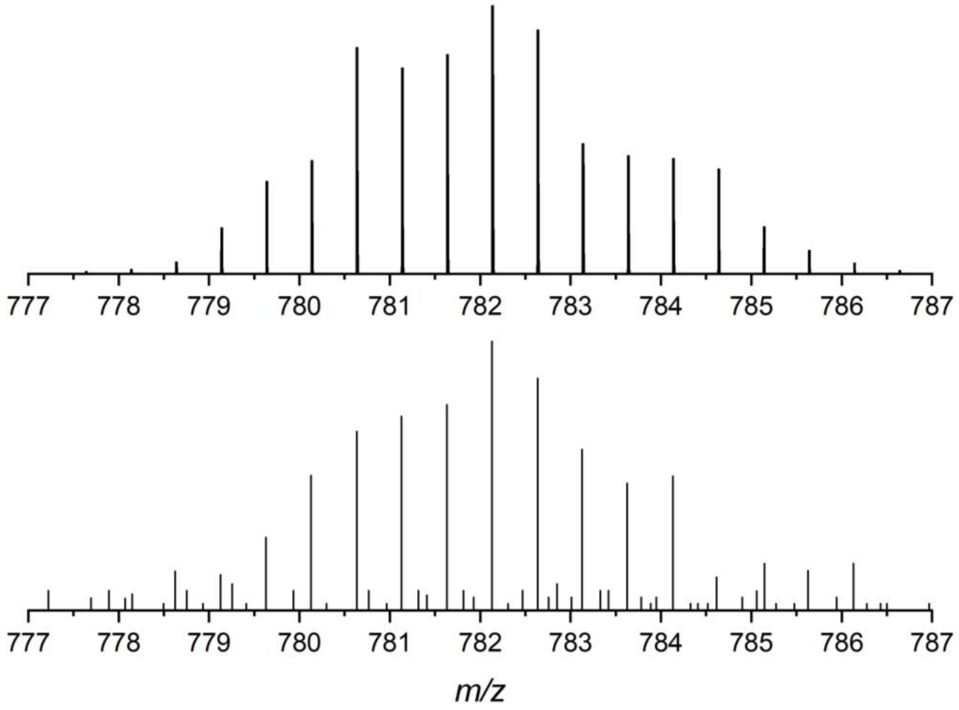

Figure S77 Calculated (top) and experimental (bottom) isotopic patterns for $\{[Pd_2(\mathbf{1})_4](BF_4)_2\}^{2+}$.

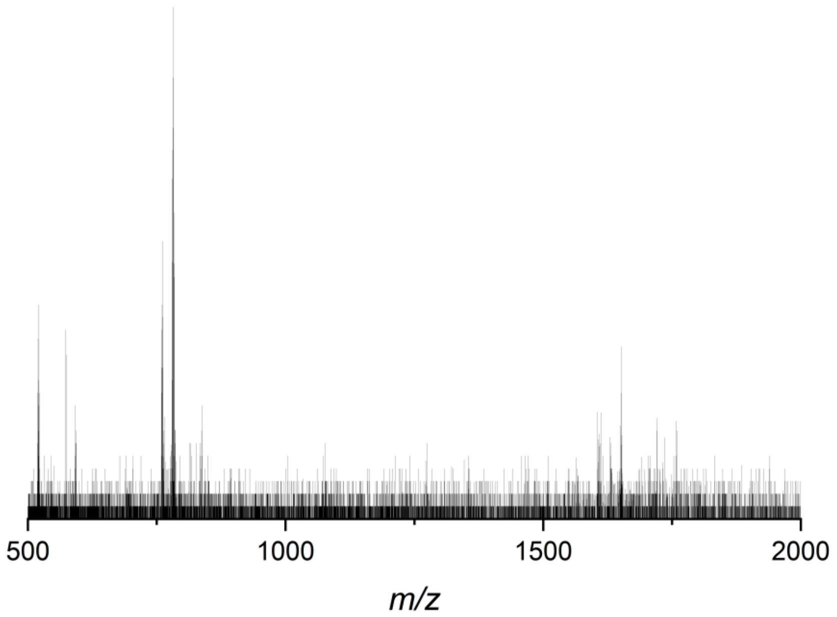

Figure S78 ESI-MS of $[Pd_2(\mathbf{1})_4](BF_4)_4$ formed in d_6-DMSO.

1 (8.8 mg, 0.030 mmol, 2 eq.) and $[Pd(CH_3CN)_4](BF_4)_2$ (6.7 mg, 0.015 mmol, 1 eq.) were sonicated in CD_3CN (0.75 mL) until all solids were dissolved. After standing at 60 °C for 24 h, the formation of a single major species could be observed by ^1H NMR. ^1H NMR (400 MHz, CD_3CN) δ: 9.45 (d, *J* = 1.6 Hz, 4H, H_k), 9.35 (d, *J* = 1.7 Hz, 4H, H_a), 8.66 (m, 4H, H_b), 8.12 (ddd, *J* = 8.0, 1.8, 1.3 Hz, 4H, H_d), 8.02 (dd, *J* = 8.2, 1.9 Hz, 4H, H_i), 7.89 (app. t, *J* = 1.3 Hz, 4H, H_h), 7.70-7.65 (m, 8H, H_e, H_g), 7.58-7.49 (m, 12H, H_c, H_j, H_f), 3.60 (s, 12H, H_l). Diffusion coefficient (500 MHz, CD_3CN) *D*: 8.10×10^{-10} m^2s^{-1}. ^{13}C NMR (101 MHz, CD_3CN) δ: 161.2, 153.7, 153.1, 152.0, 144.3, 144.0, 134.6, 134.5, 134.1, 130.7, 129.1, 128.4, 125.1, 123.3, 123.0, 122.2, 95.2, 94.4, 85.4, 85.1, 26.7. ESI-MS *m/z* = 781.06 $\{[Pd_2(C_{21}H_{14}N_2)_4](BF_4)_2\}^{2+}$ calc. 781.14.

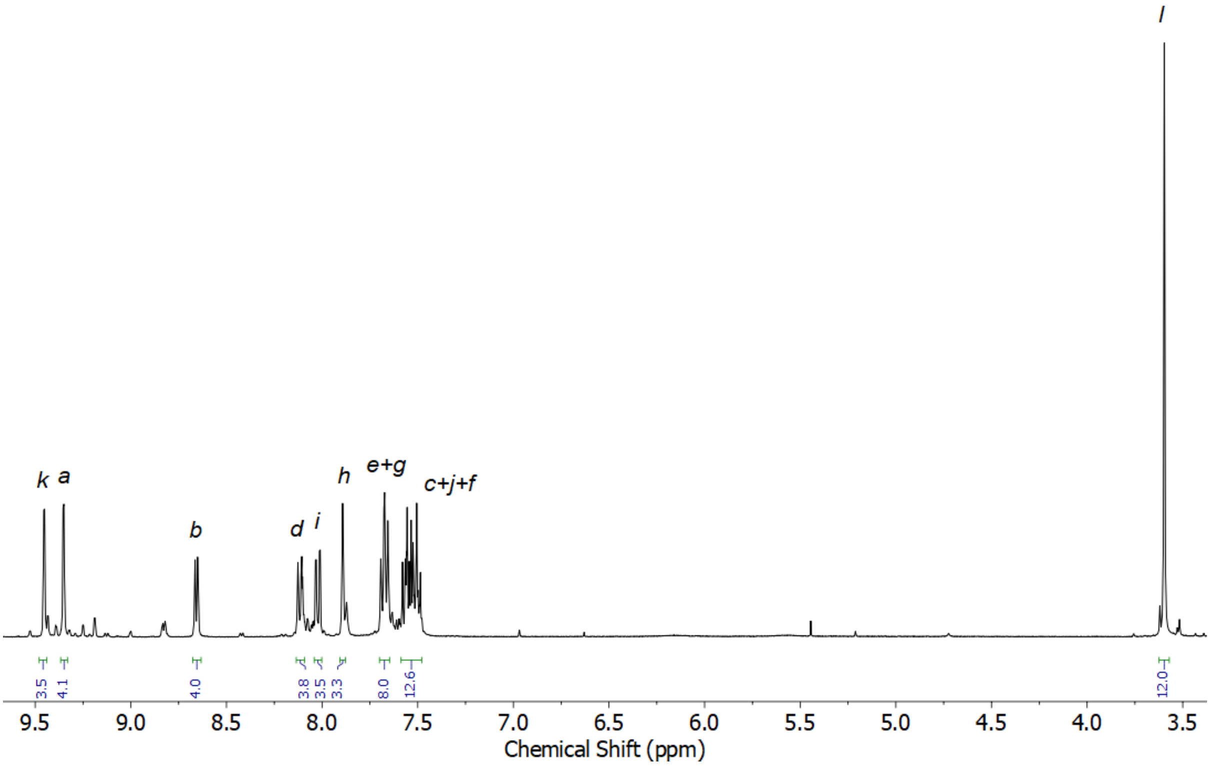

Figure S79 ^1H NMR (CD_3CN, 400 MHz) of $[Pd_2(\mathbf{1})_4](BF_4)_4$.

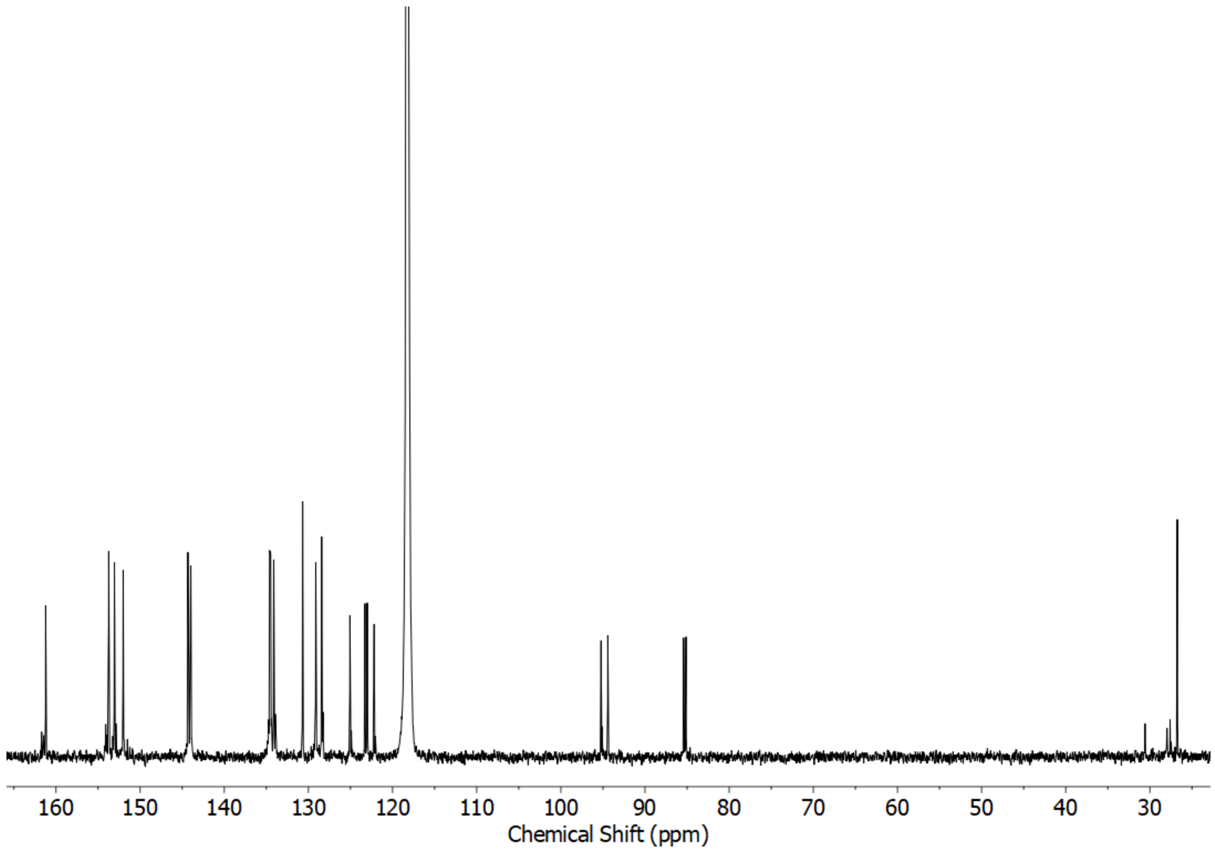

Figure S80 ^{13}C NMR (CD_3CN, 101 MHz) of $[Pd_2(\mathbf{1})_4](BF_4)_4$.

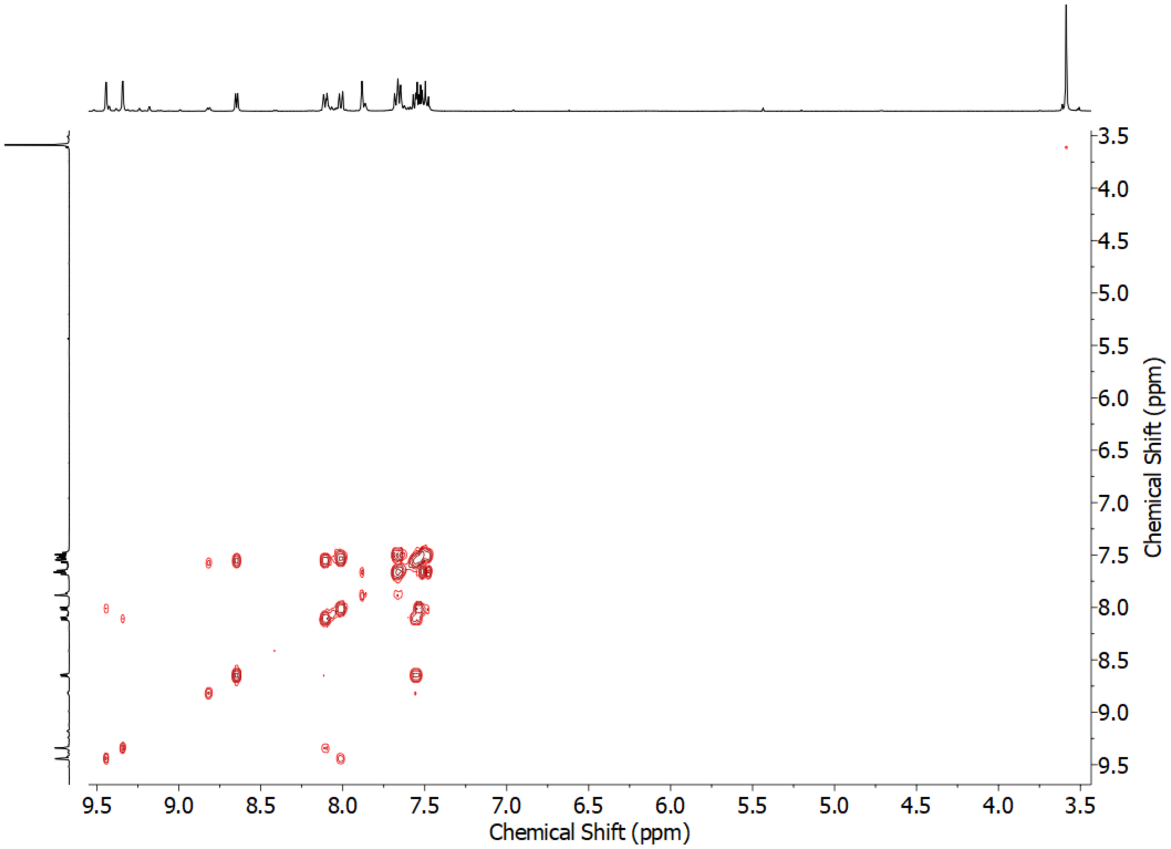

Figure S81 COSY NMR (CD_3CN) of $[Pd_2(\mathbf{1})_4](BF_4)_4$.

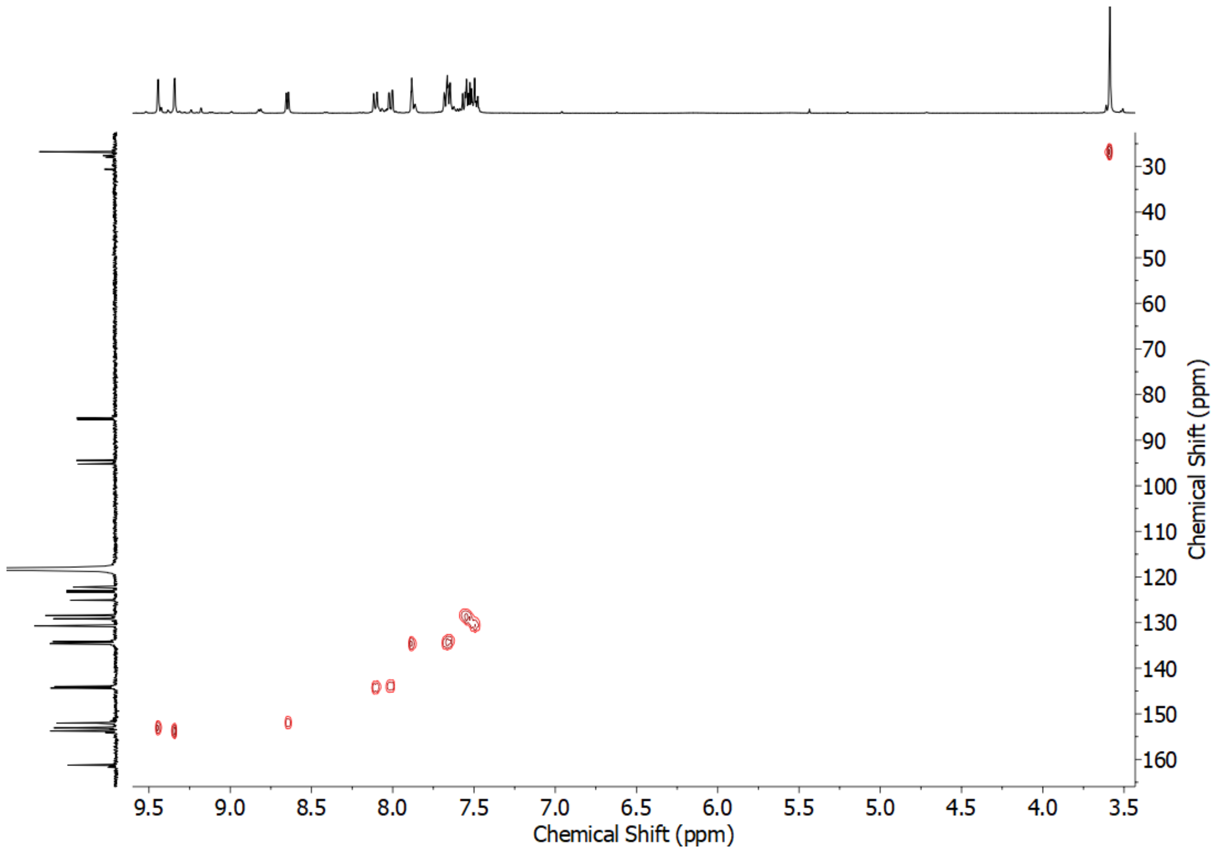

Figure S82 HSQC NMR (CD_3CN) of $[Pd_2(\mathbf{1})_4](BF_4)_4$.

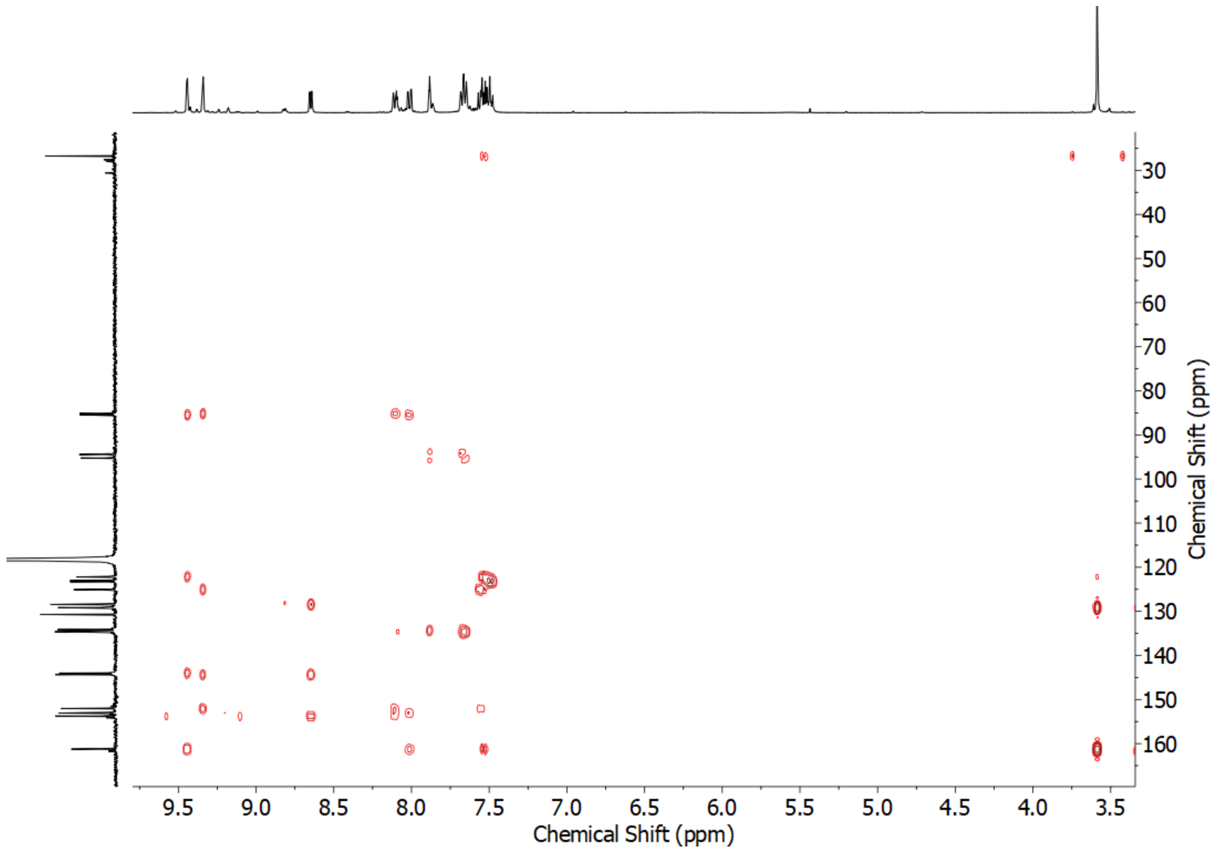

Figure S83 HMBC NMR (CD_3CN) of $[Pd_2(\mathbf{1})_4](BF_4)_4$.

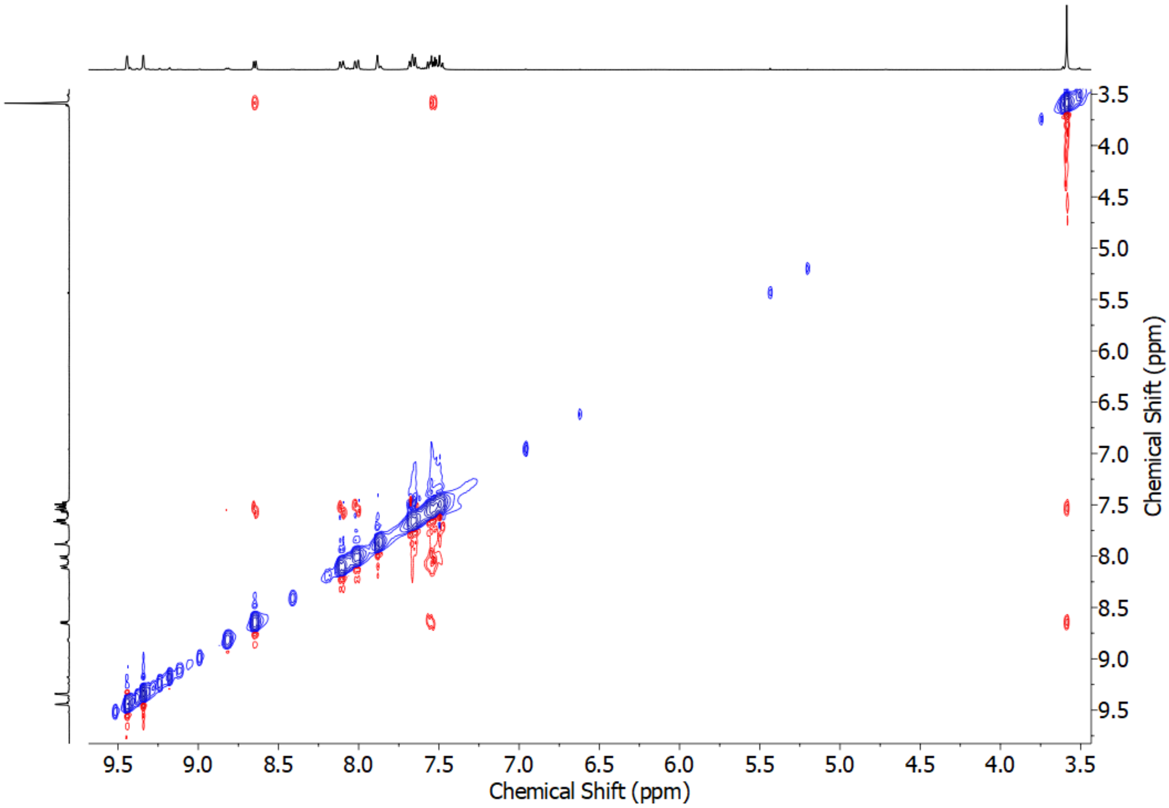

Figure S84 ROESY NMR (CD_3CN) of $[Pd_2(\mathbf{1})_4](BF_4)_4$.

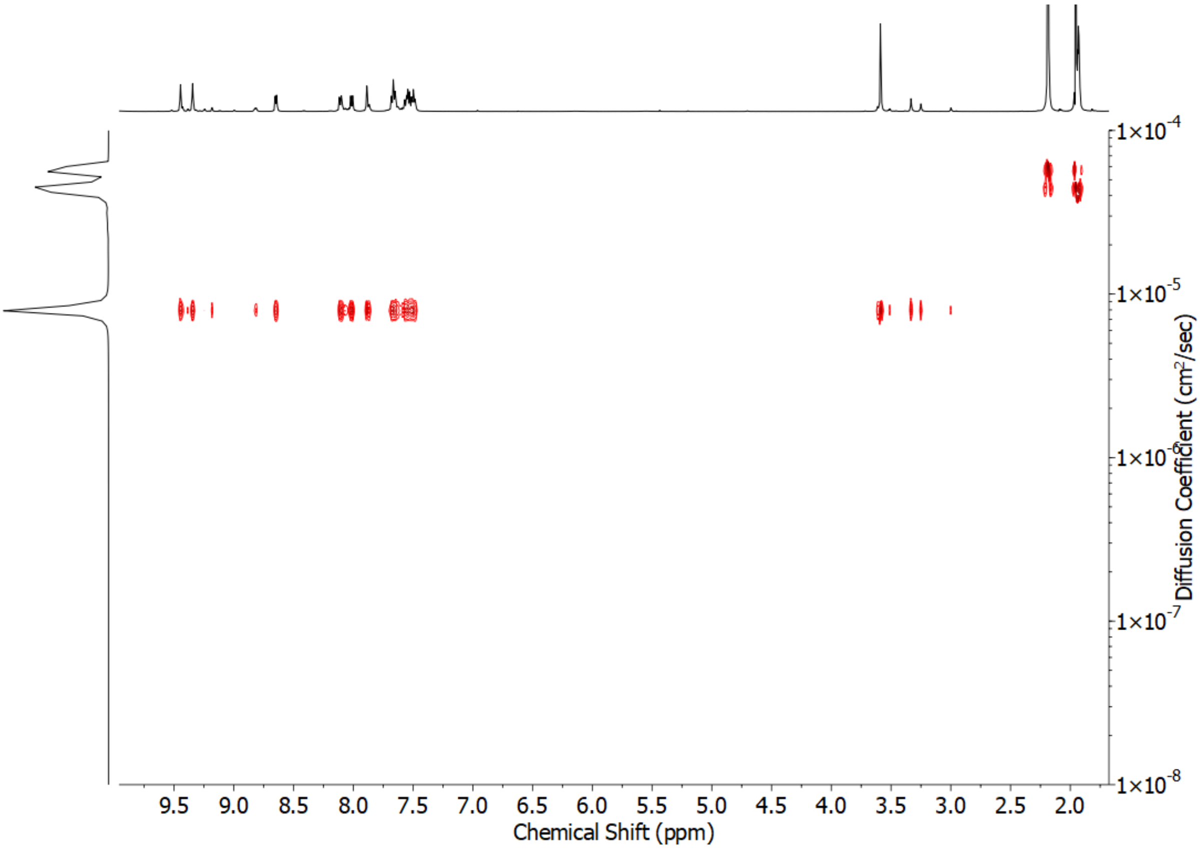

Figure S85 DOSY NMR (CD_3CN, 500 MHz) of $[Pd_2(\mathbf{1})_4](BF_4)_4$.

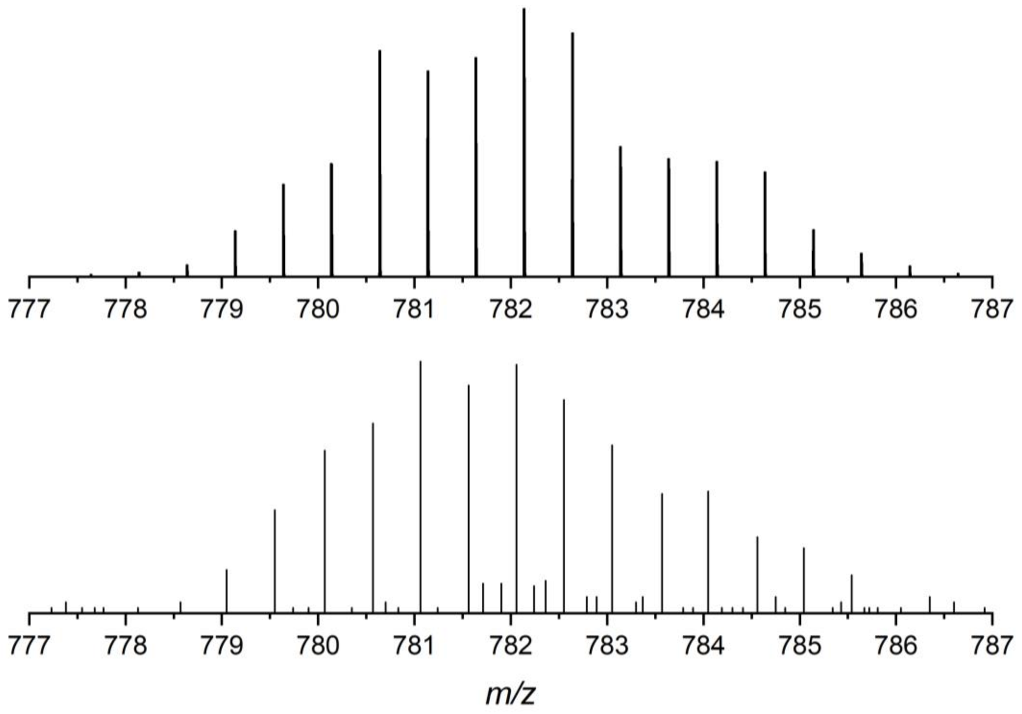

Figure S86 Calculated (top) and experimental (bottom) isotopic patterns for $\{[Pd_2(\mathbf{1})_4](BF_4)_2\}^{2+}$.

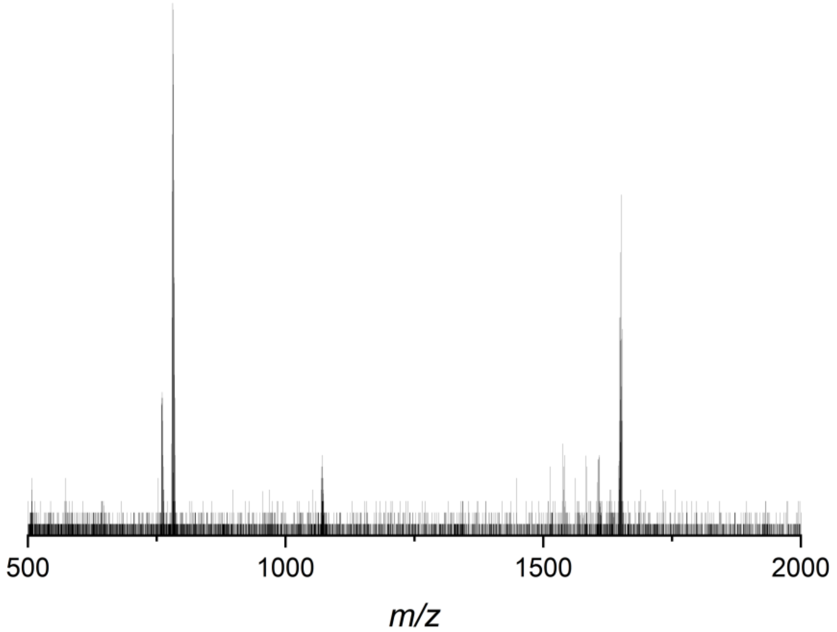

Figure 87 ESI-MS of $[Pd_2(\mathbf{1})_4](BF_4)_4$ formed in CD_3CN.

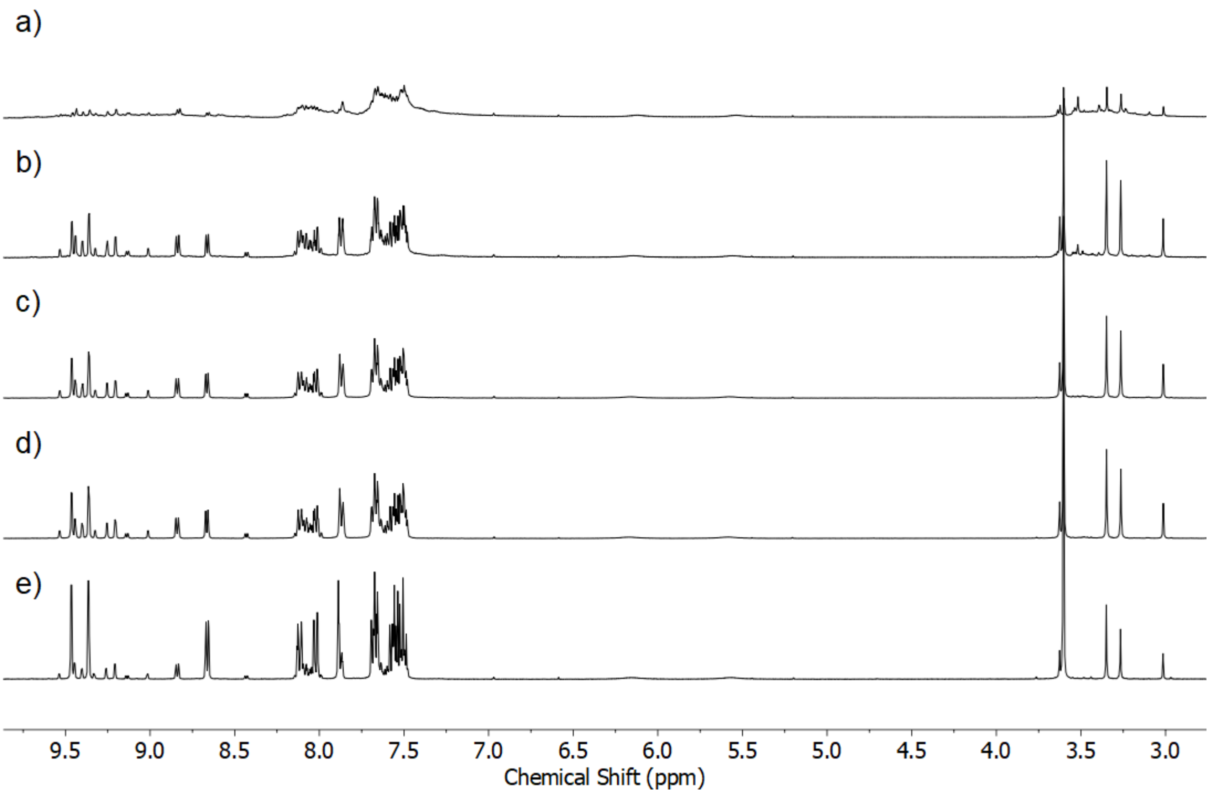

Figure S88 ^{1}H NMR (CD_3CN, 400 MHz) of 2:1 mixture of **1** and $[Pd(CH_3CN)_4](BF_4)_2$ after a) 0 h, b) 2 h, c) 6 h and d) 22 h at room temperature, and e) after heating at 60 °C.

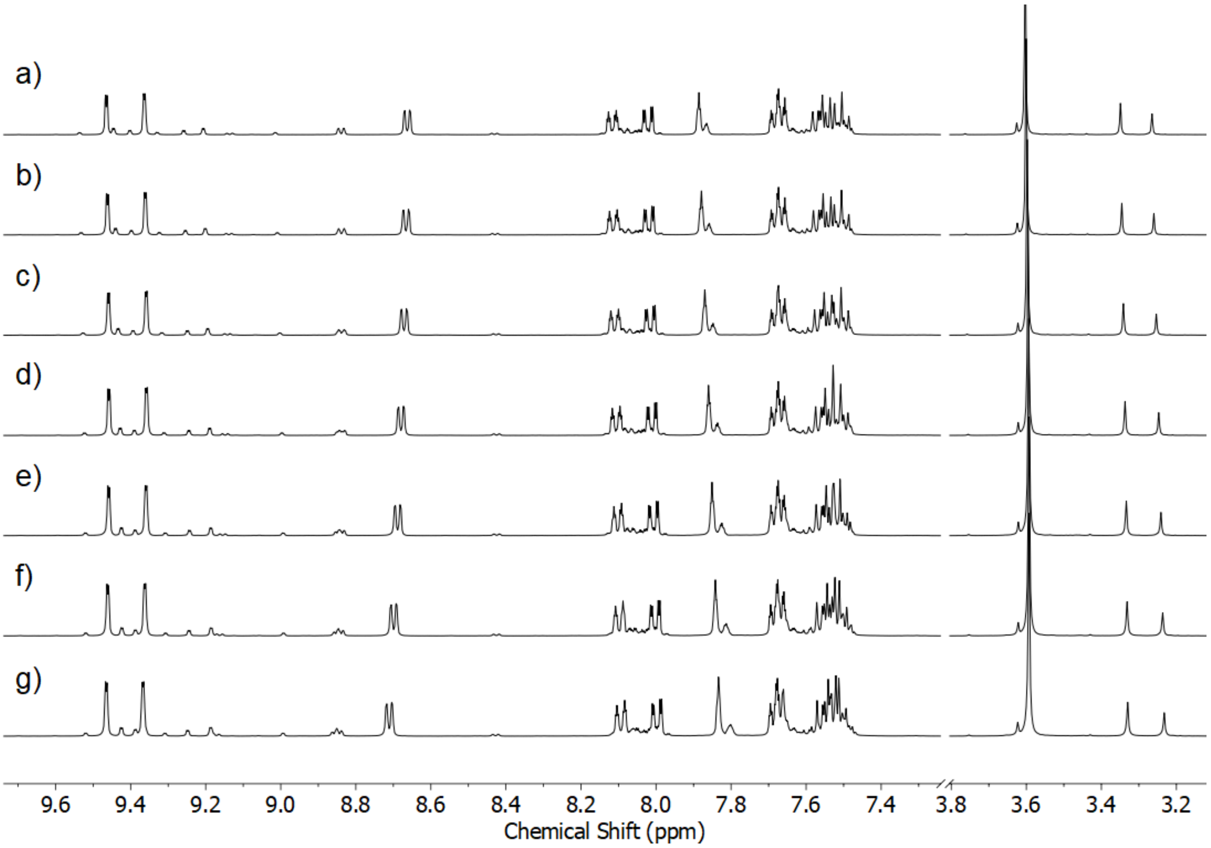

Figure S89 Partial ^{1}H NMR (CD_3CN, 400 MHz) of $[Pd_2(\mathbf{1})_4](BF_4)_4$ at a) 298 K, b) 293 K, c) 283 K, d) 273 K, e) 263 K, f) 253 K and g) 243 K.

[Pd$_2$(2)$_4$](BF$_4$)$_4$

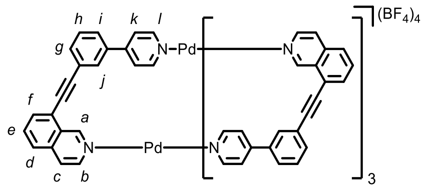

2 (9.2 mg, 0.030 mmol, 2 eq.) and [Pd(CH$_3$CN)$_4$](BF$_4$)$_2$ (6.7 mg, 0.015 mmol, 1 eq.) were sonicated in *d*$_6$-DMSO (0.75 mL) until all solids were dissolved. After standing at rt for 2 h, quantitative conversion to [Pd$_2$(**2**)$_4$](BF$_4$)$_4$ was observed by ^{1}H NMR. ^{1}H NMR (400 MHz, *d*$_6$-DMSO) δ: 10.02 (s, 4H, H$_a$), 9.67 (d, *J* = 6.6 Hz, 4H, H$_b$), 9.59 (d, *J* = 6.6 Hz, 8H, H$_l$), 8.48 (d, *J* = 6.5 Hz, 4H, H$_c$), 8.37 (d, *J* = 6.9 Hz, 8H, H$_k$), 8.30 (s, 4H, H$_j$), 8.25 (d, *J* = 8.3 Hz, 4H, H$_d$), 8.13 (dd, *J* = 7.2, 1.0 Hz, 4H, H$_f$), 8.07-8.02 (m, 8H, H$_e$, H$_i$), 7.87 (dt, *J* = 7.6, 1.2 Hz, 4H, H$_g$), 7.70 (app. t, *J* = 7.8 Hz, 4H, H$_h$). Diffusion coefficient (500 MHz, *d*$_6$-DMSO) *D*: 1.15 × 10^{-10} m^{2}s^{-1}. ^{13}C NMR (101 MHz, *d*$_6$-DMSO) δ: 153.0, 151.0, 149.8, 142.9, 136.4, 134.7, 134.3, 134.1, 133.8, 131.8, 130.4, 128.2, 128.0, 127.7, 125.1, 124.3, 122.3, 120.4, 96.2, 86.0. ESI-MS *m/z* = 805.15 {[Pd$_2$(C$_{22}$H$_{14}$N$_2$)$_4$](BF$_4$)$_2$}$^{2+}$ calc. 805.14.

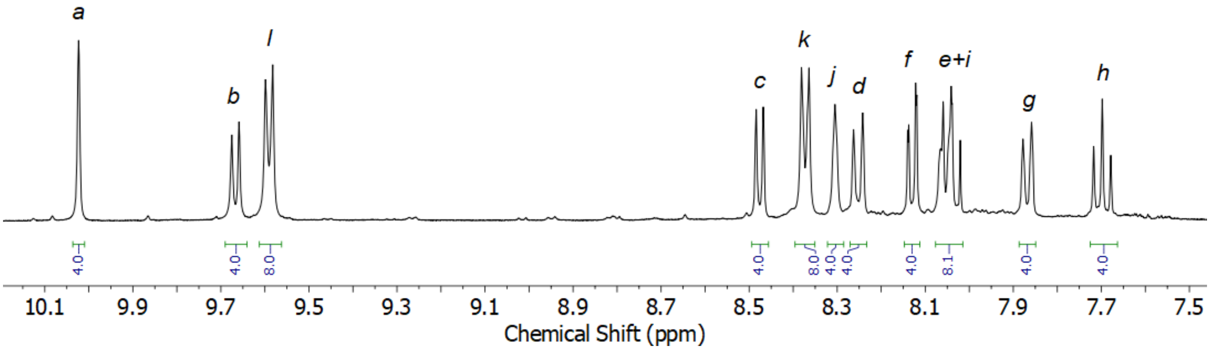

Figure S90 ^{1}H NMR (*d*$_6$-DMSO, 400 MHz) of [Pd$_2$(**2**)$_4$](BF$_4$)$_4$.

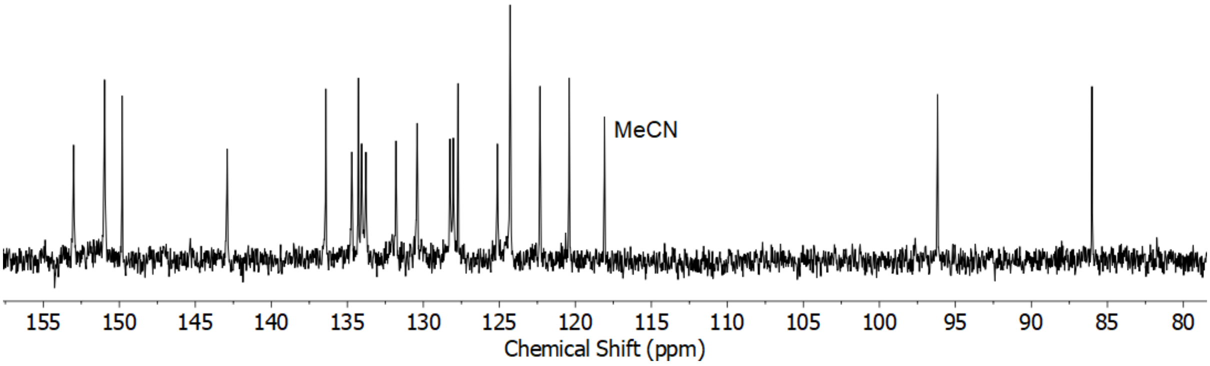

Figure S91 ^{13}C NMR (*d*$_6$-DMSO, 101 MHz) of [Pd$_2$(**2**)$_4$](BF$_4$)$_4$.

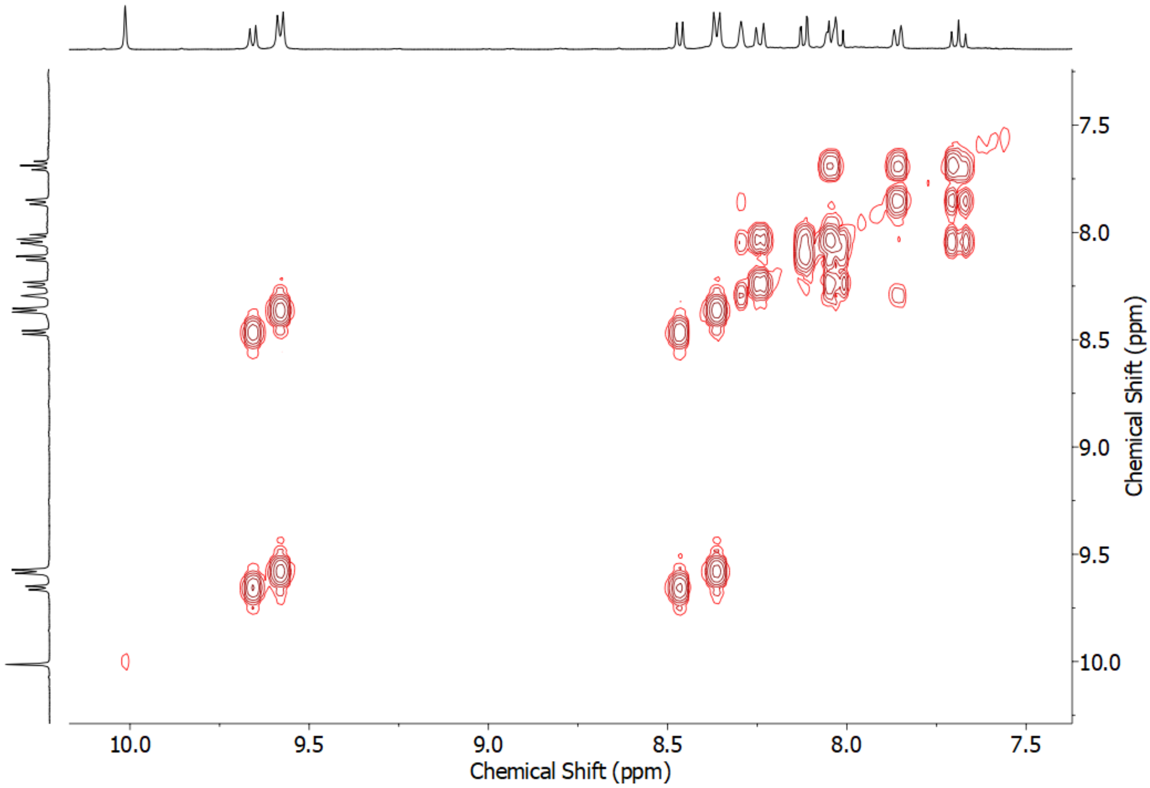

Figure S92 COSY NMR (d_6-DMSO) of $[Pd_2(\mathbf{2})_4](BF_4)_4$.

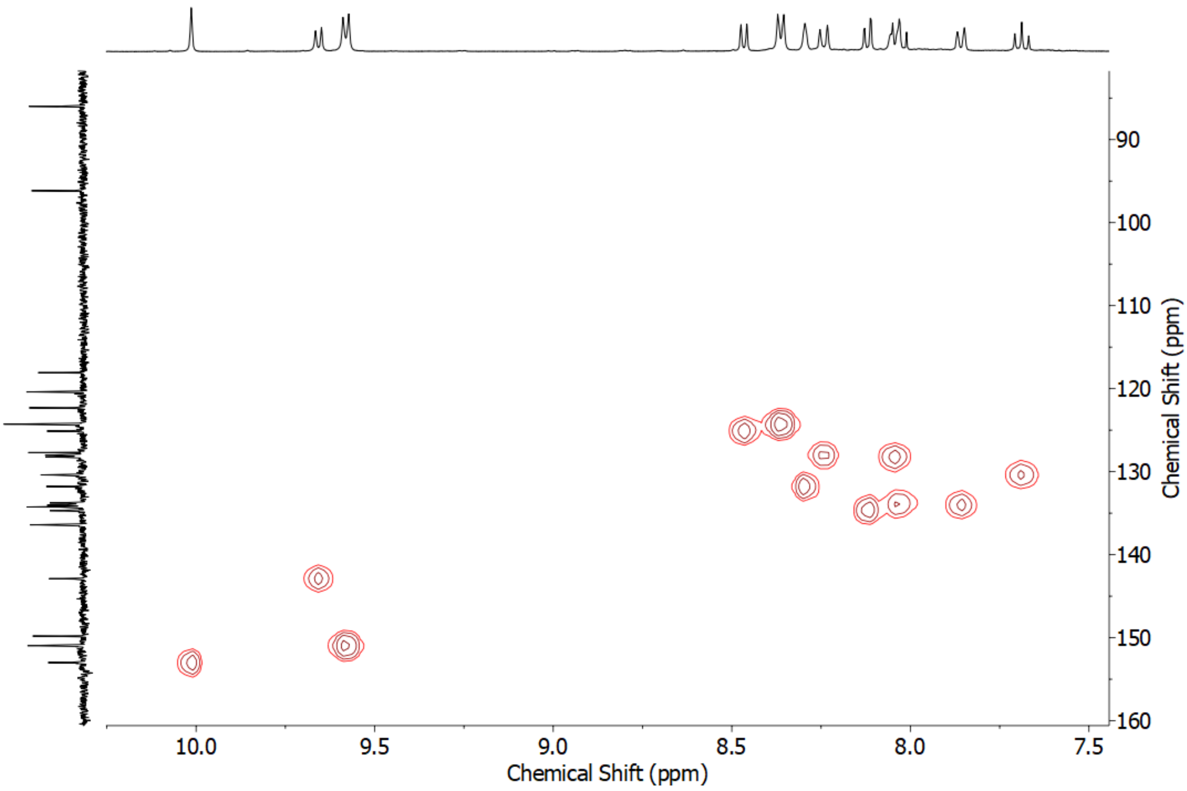

Figure S93 HSQC NMR (d_6-DMSO) of $[Pd_2(\mathbf{2})_4](BF_4)_4$.

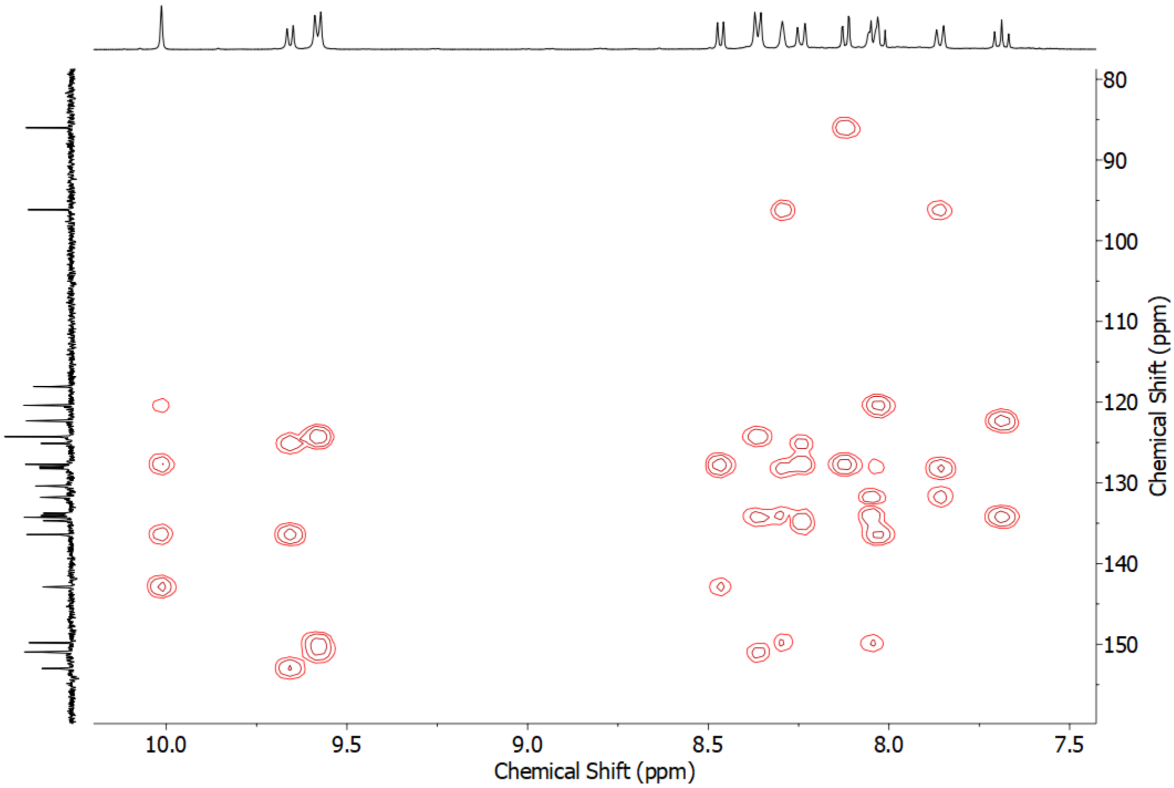

Figure S94 HMBC NMR (d_6-DMSO) of $[Pd_2(\mathbf{2})_4](BF_4)_4$.

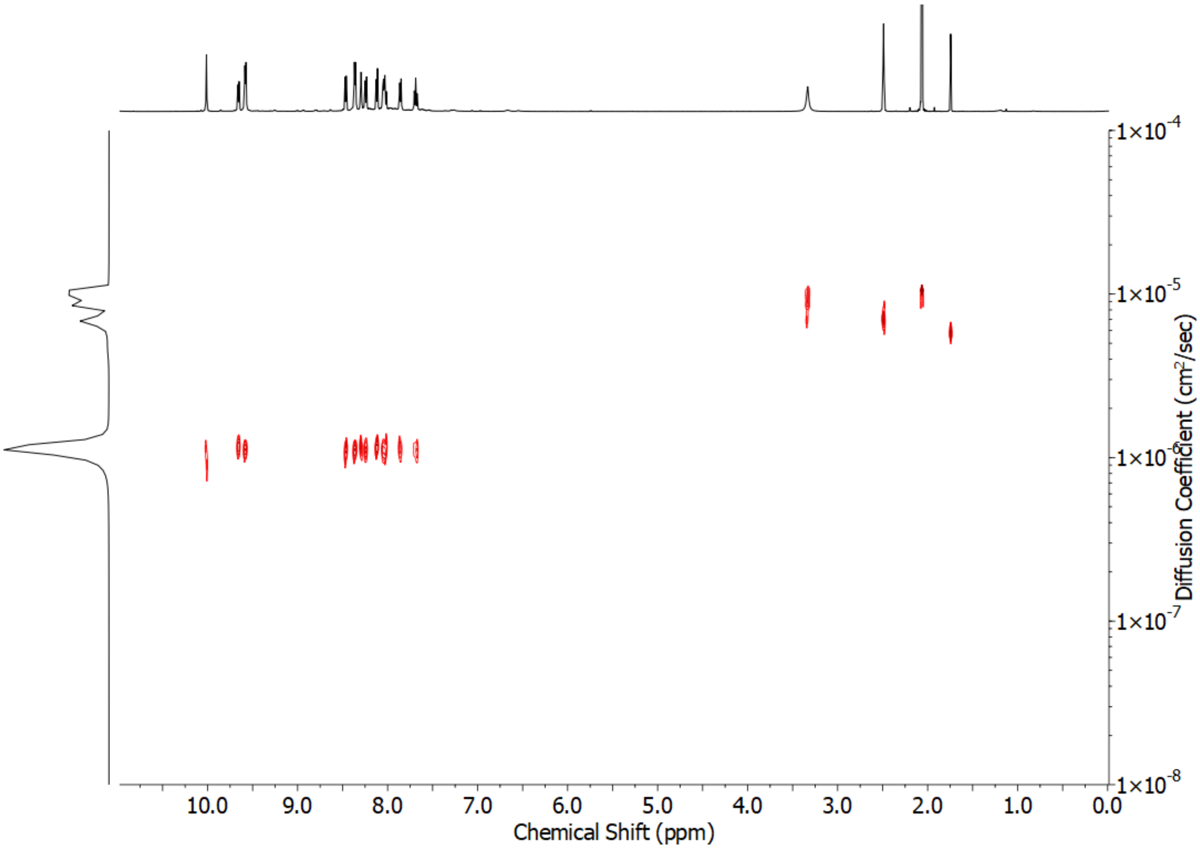

Figure S95 DOSY NMR (d_6-DMSO, 500 MHz) of $[Pd_2(\mathbf{2})_4](BF_4)_4$.

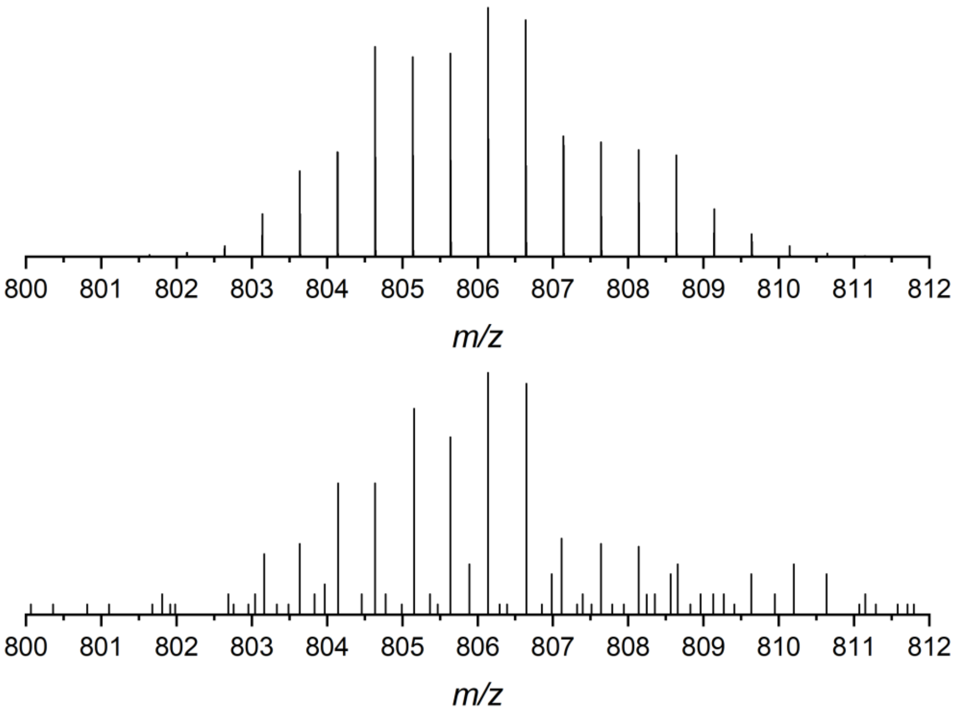

Figure S96 Experimental (top) and calculated (bottom) isotopic patterns for $\{[Pd_2(\mathbf{2})_4](BF_4)_2\}^{2+}$.

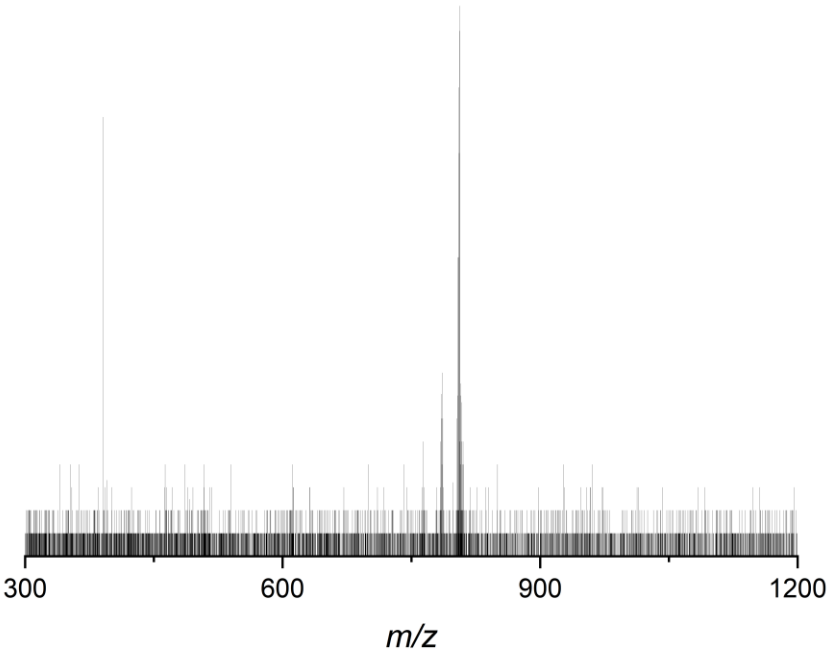

Figure S97 ESI-MS of $[Pd_2(\mathbf{2})_4](BF_4)_4$.

[Pd$_2$(3)$_4$](BF$_4$)$_4$

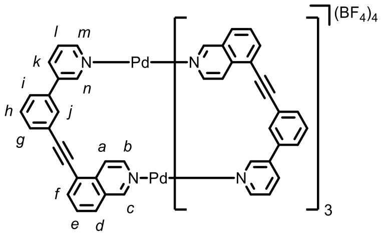

3 (9.2 mg, 0.030 mmol, 2 eq.) and [Pd(CH$_3$CN)$_4$](BF$_4$)$_2$ (6.7 mg, 0.015 mmol, 1 eq.) were sonicated in d_6-DMSO (0.75 mL) until all solids were dissolved. After standing at rt for 2 h, quantitative conversion to [Pd$_2$(**3**)$_4$](BF$_4$)$_4$ was observed by ^{1}H NMR. ^{1}H NMR (400 MHz, d_6-DMSO) δ: 10.00-9.98 (m, 8H, H$_b$, H$_c$), 9.81 (d, J = 1.6 Hz, 4H, H$_n$), 9.59 (dd, J = 5.7, 1.3 Hz, 4H, H$_m$), 8.94 (d, J = 6.6 Hz, 4H, H$_a$), 8.58 (d, J = 7.8 Hz, 4H, H$_k$), 8.40 (s, 4H, H$_j$), 8.29 (d, J = 8.5 Hz, 4H, H$_d$), 8.17 (dd, J = 7.2, 1.1 Hz, 4H, H$_f$), 7.99 (dd, J = 8.1, 5.7 Hz, 4H, H$_l$), 7.88 (m, 4H, H$_i$), 7.84 (dd, J = 8.3, 7.3 Hz, 4H, H$_e$), 7.67 (app. dt, J = 7.7, 1.3 Hz, 4H, H$_g$), 7.56 (app. t, J = 7.8 Hz, 4H, H$_h$). Diffusion coefficient (500 MHz, d_6-DMSO) D: 1.16 × 10^{-10} m^{2}s^{-1}. ^{13}C NMR (101 MHz, d_6-DMSO) δ: 155.7, 150.2, 149.2, 143.2, 138.3, 136.8, 136.6, 135.8, 134.4, 132.2, 130.1, 129.6, 129.4, 129.3, 128.2, 128.0, 127.8, 124.0, 122.7, 119.4, 95.7, 85.4. ESI-MS m/z = 805.1 {[Pd$_2$(C$_{22}$H$_{14}$N$_2$)$_4$](BF$_4$)$_2$}$^{2+}$ calc. 805.1; 1697.3 {[Pd$_2$(C$_{22}$H$_{14}$N$_2$)$_4$](BF$_4$)$_3$}$^+$ calc. 1697.3.

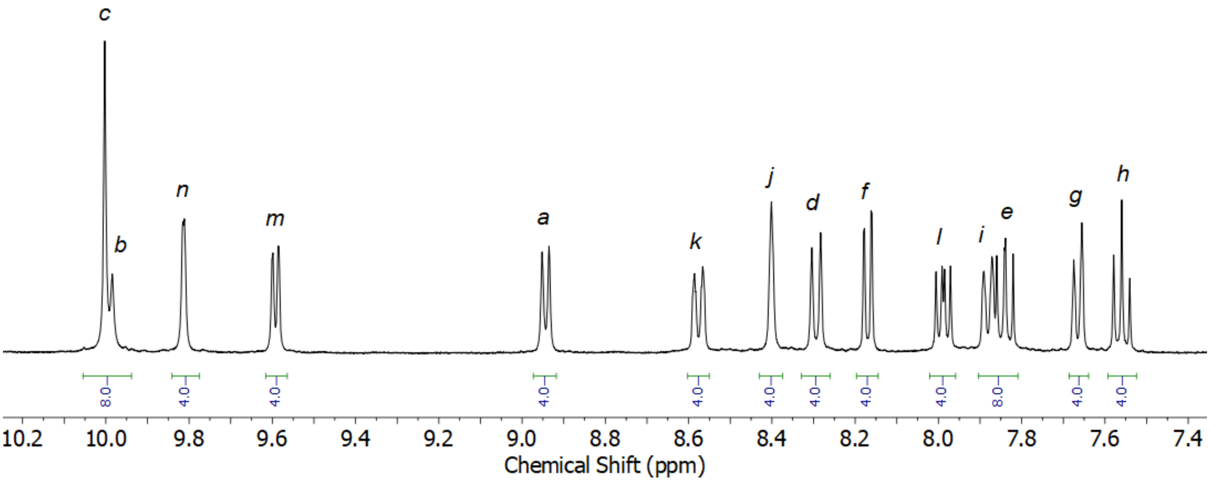

Figure S98 ^{1}H NMR (d_6-DMSO, 400 MHz) of [Pd$_2$(**3**)$_4$](BF$_4$)$_4$.

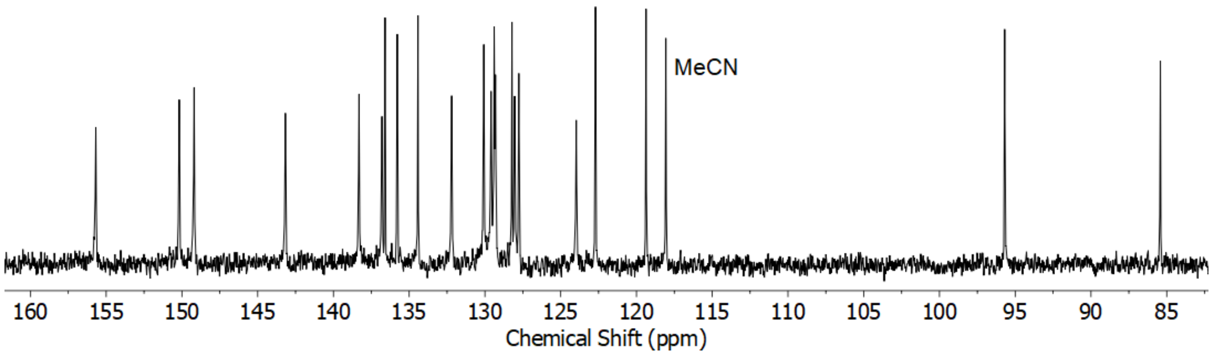

Figure S99 ^{13}C NMR (d_6-DMSO, 101 MHz) of $[Pd_2(\mathbf{3})_4](BF_4)_4$.

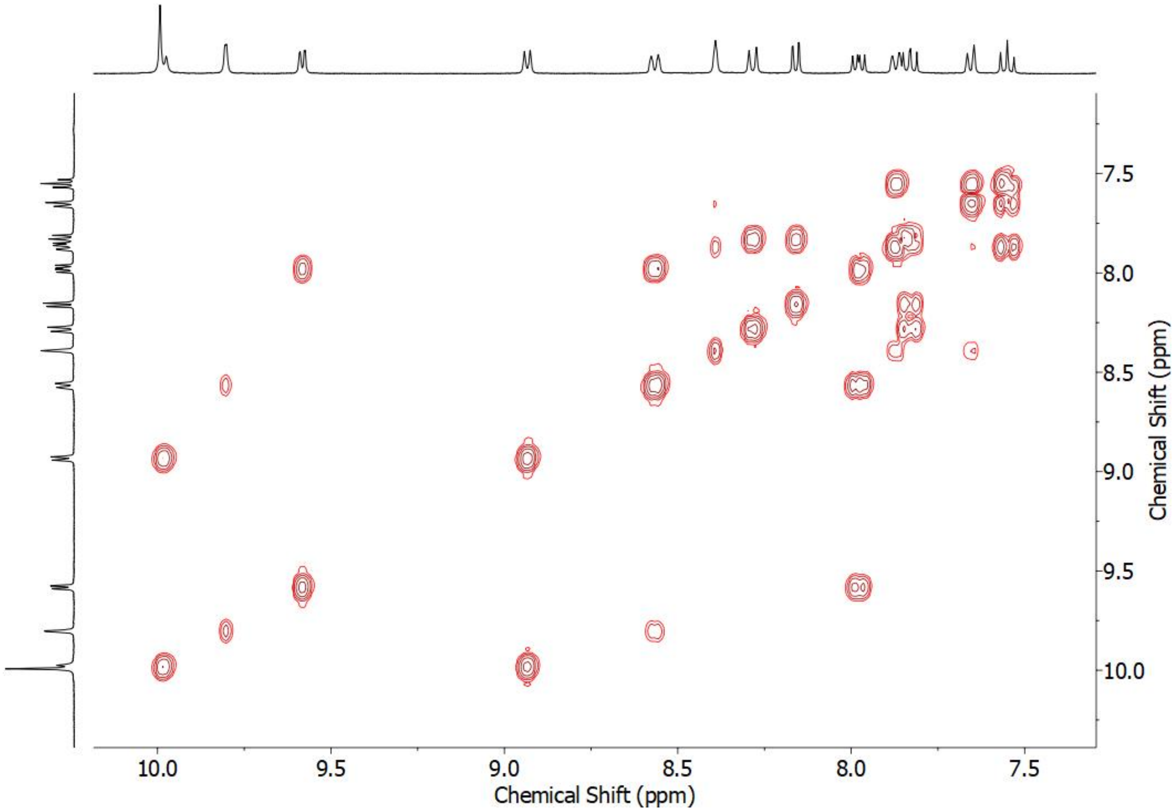

Figure S100 COSY NMR (d_6-DMSO) of $[Pd_2(\mathbf{3})_4](BF_4)_4$.

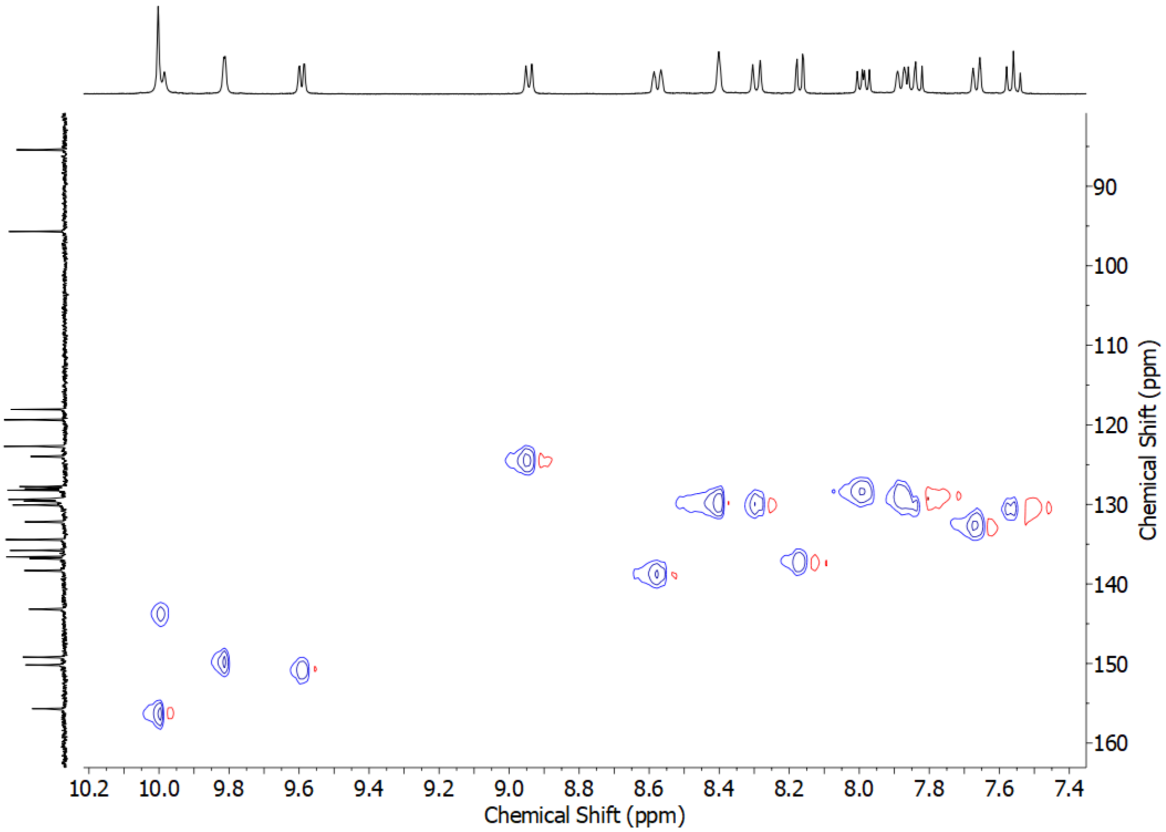

Figure S101 HSQC NMR (d_6-DMSO) of $[Pd_2(\mathbf{3})_4](BF_4)_4$.

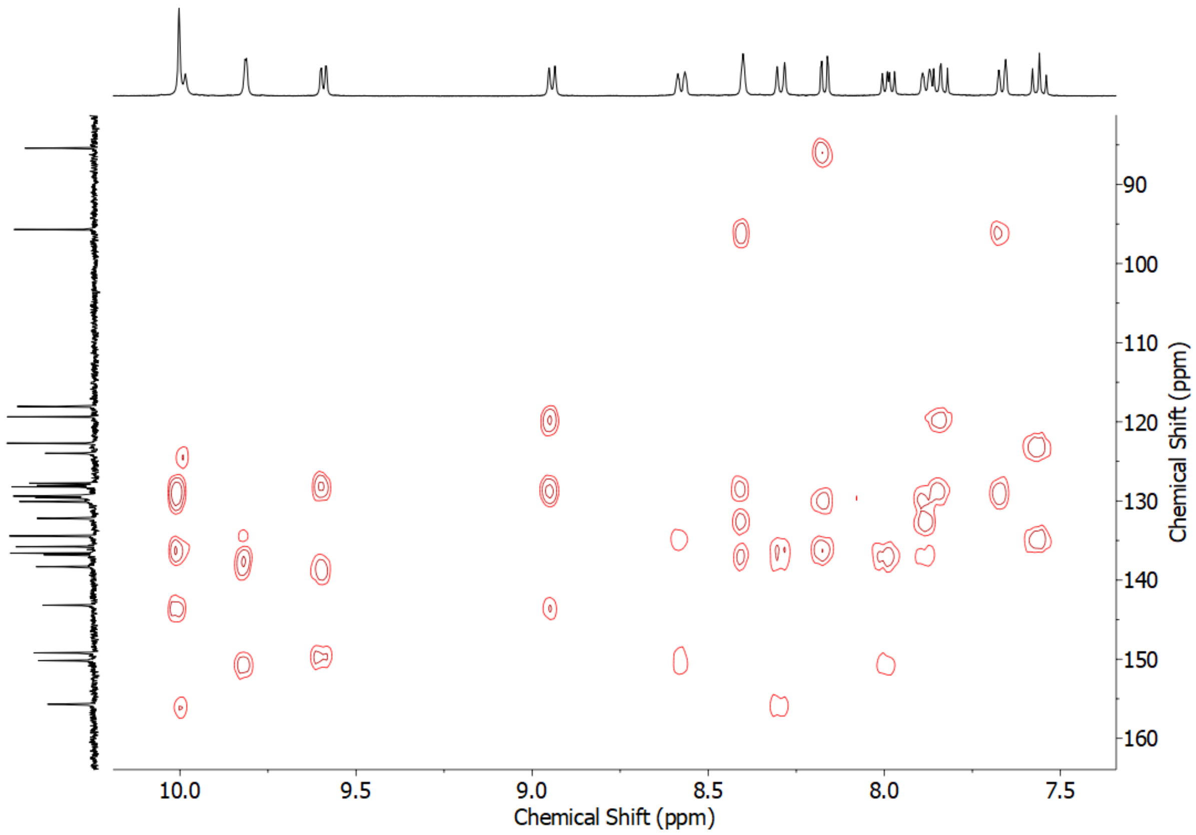

Figure S102 HMBC NMR (d_6-DMSO) of $[Pd_2(\mathbf{3})_4](BF_4)_4$.

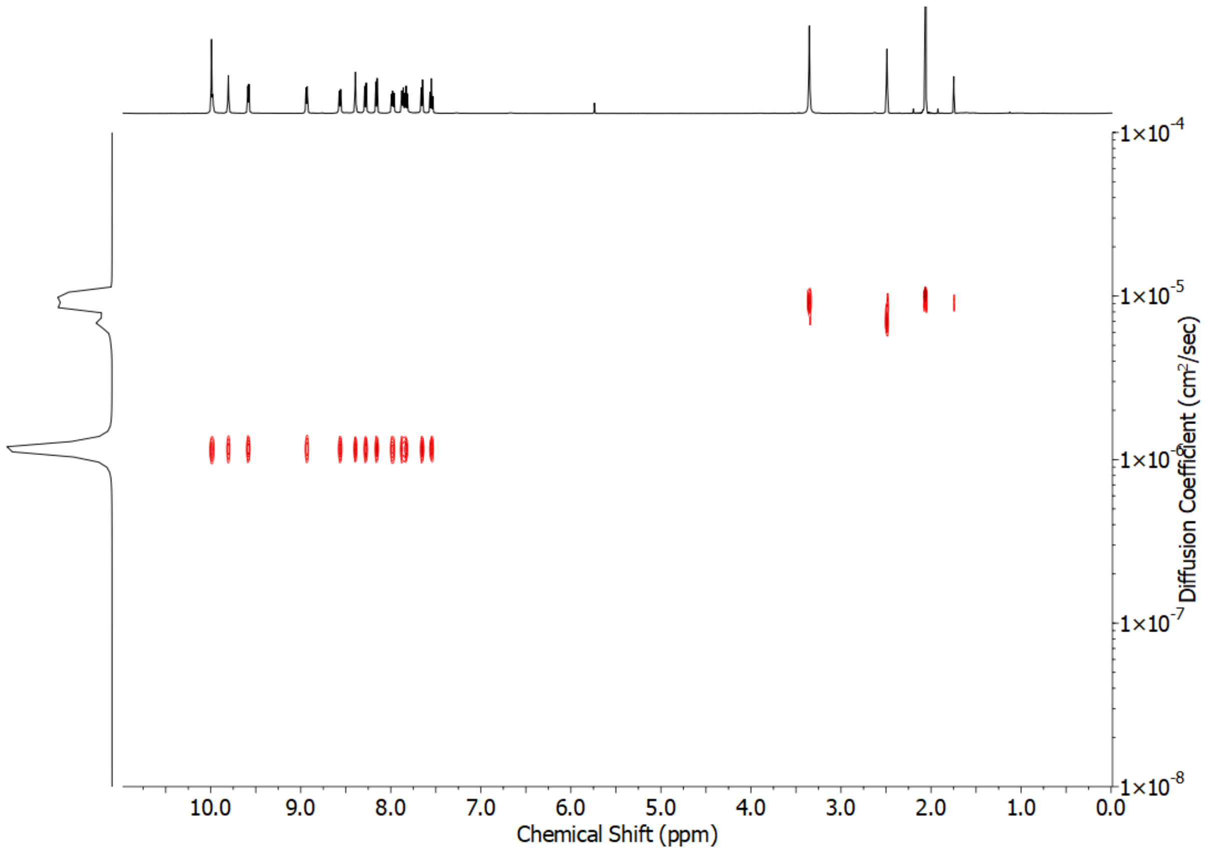

Figure S103 DOSY NMR (d_6-DMSO, 500 MHz) of $[Pd_2(\mathbf{3})_4](BF_4)_4$.

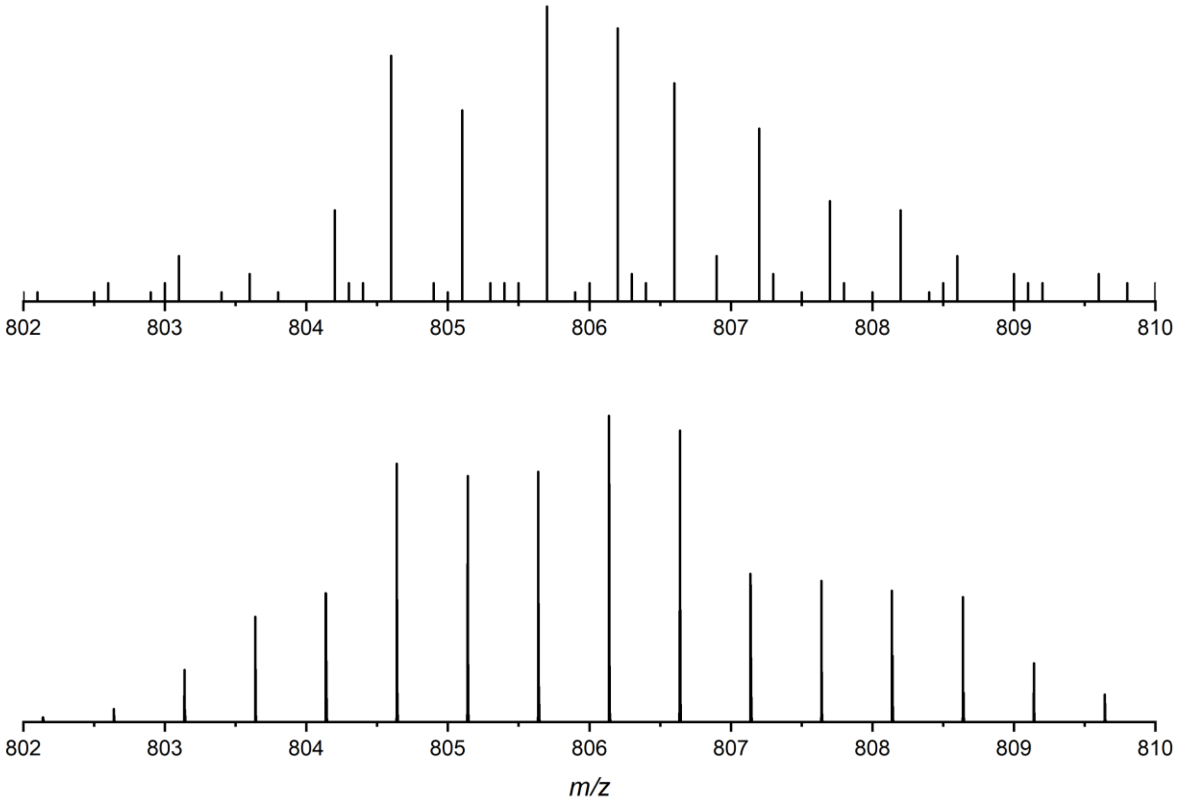

Figure S104 Calculated (top) and experimental (bottom) isotopic patterns for $\{[Pd_2(\mathbf{3})_4](BF_4)_2\}^{2+}$.

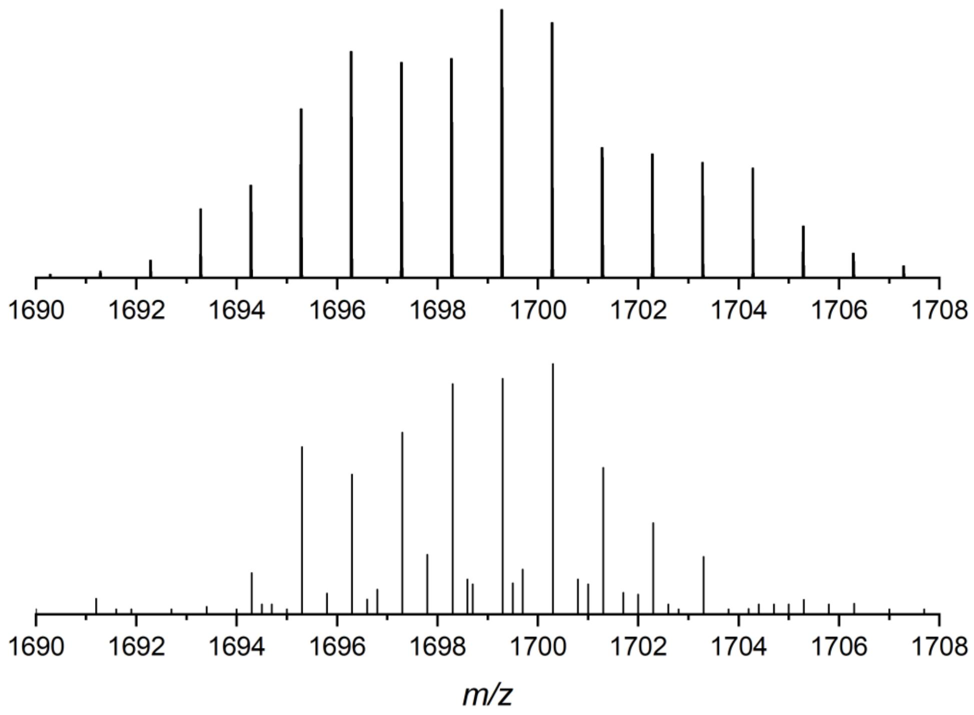

Figure S105 Calculated (top) and experimental (bottom) isotopic patterns for $\{[Pd_2(\mathbf{3})_4](BF_4)_3\}^+$.

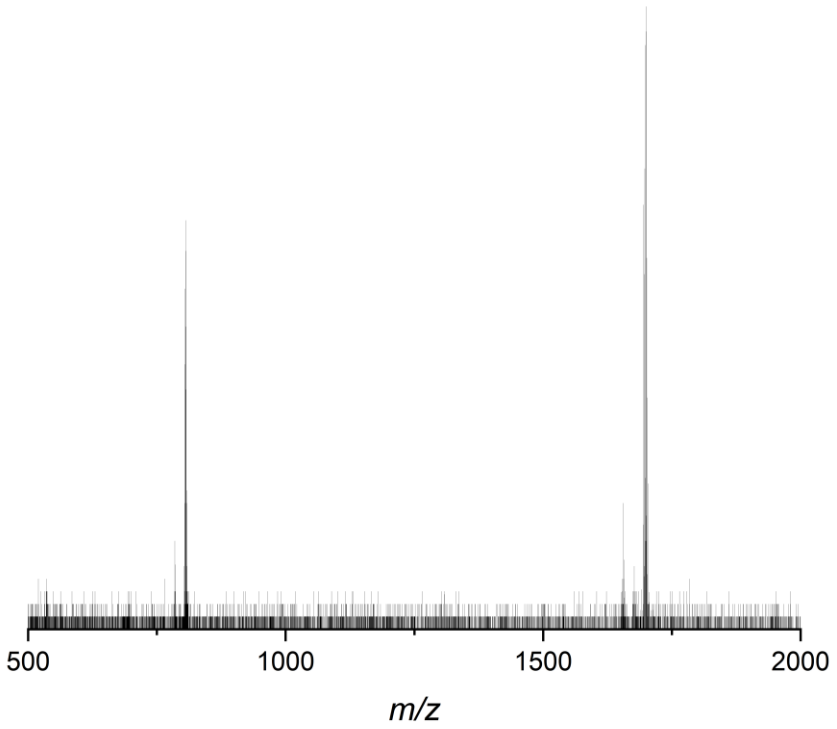

Figure S106 ESI-MS of $[Pd_2(\mathbf{3})_4](BF_4)_4$.

$[Pd_2(4)_4](BF_4)_4$

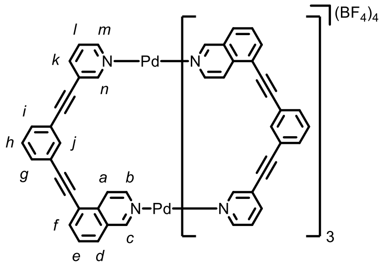

4 (9.9 mg, 0.030 mmol, 2 eq.) and $[Pd(CH_3CN)_4](BF_4)_2$ (6.7 mg, 0.015 mmol, 1 eq.) were sonicated in *d*$_6$-DMSO (0.75 mL) until all solids were dissolved. After standing at rt for 18 h, quantitative conversion to $[Pd_2(\mathbf{4})_4](BF_4)_4$ was observed by ^{1}H NMR. ^{1}H NMR (400 MHz, *d*$_6$-DMSO) δ: 10.10 (s, 4H, H_c), 9.62 (d, *J* = 1.9 Hz, 4H, H_n), 9.54 (dd, *J* = 5.8, 1.1 Hz, 4H, H_m), 9.49 (d, *J* = 6.7 Hz, 4H, H_b), 8.58 (d, *J* = 6.7 Hz, 4H, H_a), 8.34 (d, *J* = 8.5 Hz, 4H, H_d), 8.30 (app. dt, *J* = 8.0, 1.5 Hz, 4H, H_k), 8.25 (dd, *J* = 7.3, 1.0 Hz, 4H, H_f), 7.98 (app. td, *J* = 1.7, 1.0 Hz, 4H, H_j), 7.94-7.89 (m, 8H, H_e, H_l), 7.74 (app. dt, *J* = 7.7, 1.3 Hz, 4H, H_g/H_i), 7.69 (app. dt, *J* = 7.8, 1.3 Hz, 4H, H_g/H_i), 7.59-7.55 (m, 4H, H_h). Diffusion coefficient (500 MHz, *d*$_6$-DMSO) *D*: 9.78×10^{-11} m^{2}s^{-1}. ^{13}C NMR (101 MHz, *d*$_6$-DMSO) δ: 156.8, 153.0, 150.7, 142.7, 141.9, 137.6, 135.4, 134.3, 132.7 (×2), 130.2, 130.1, 129.7, 128.4, 127.6, 122.8, 122.2, 121.7, 121.5, 118.9, 95.0, 93.3, 85.6, 84.8. ESI-MS *m/z* = 853.13 $\{[Pd_2(C_{24}H_{14}N_2)_4](BF_4)_2\}^{2+}$ calc. 853.14; 539.75 $\{[Pd_2(C_{24}H_{14}N_2)_4](BF_4)\}^{3+}$ calc. 539.76.

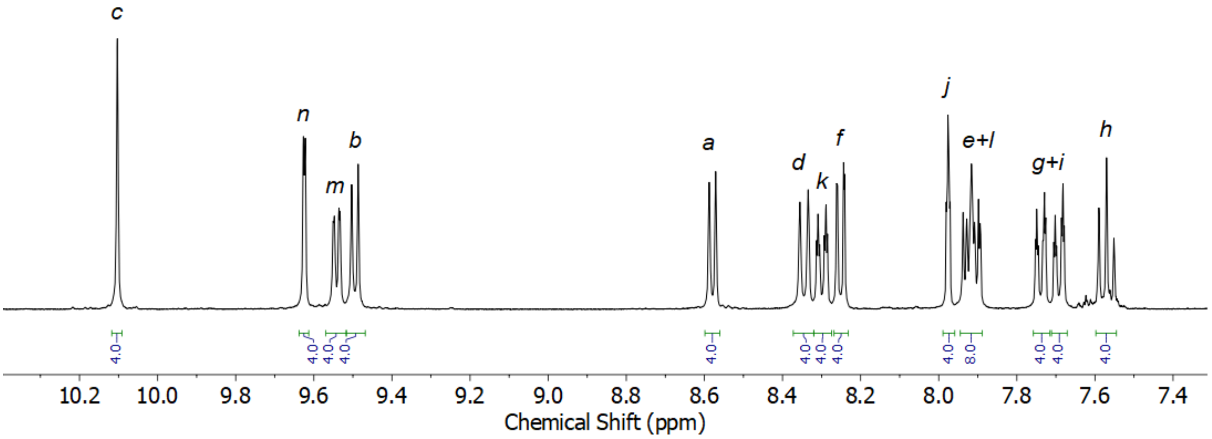

Figure S107 ^{1}H NMR (*d*$_6$-DMSO, 400 MHz) of $[Pd_2(\mathbf{4})_4](BF_4)_4$.

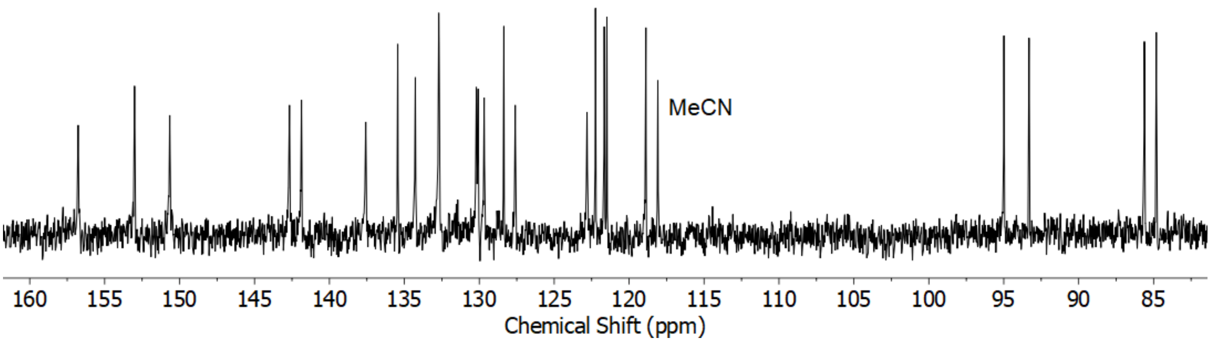

Figure S108 ^{13}C NMR (*d*$_6$-DMSO, 101 MHz) of $[Pd_2(\mathbf{4})_4](BF_4)_4$.

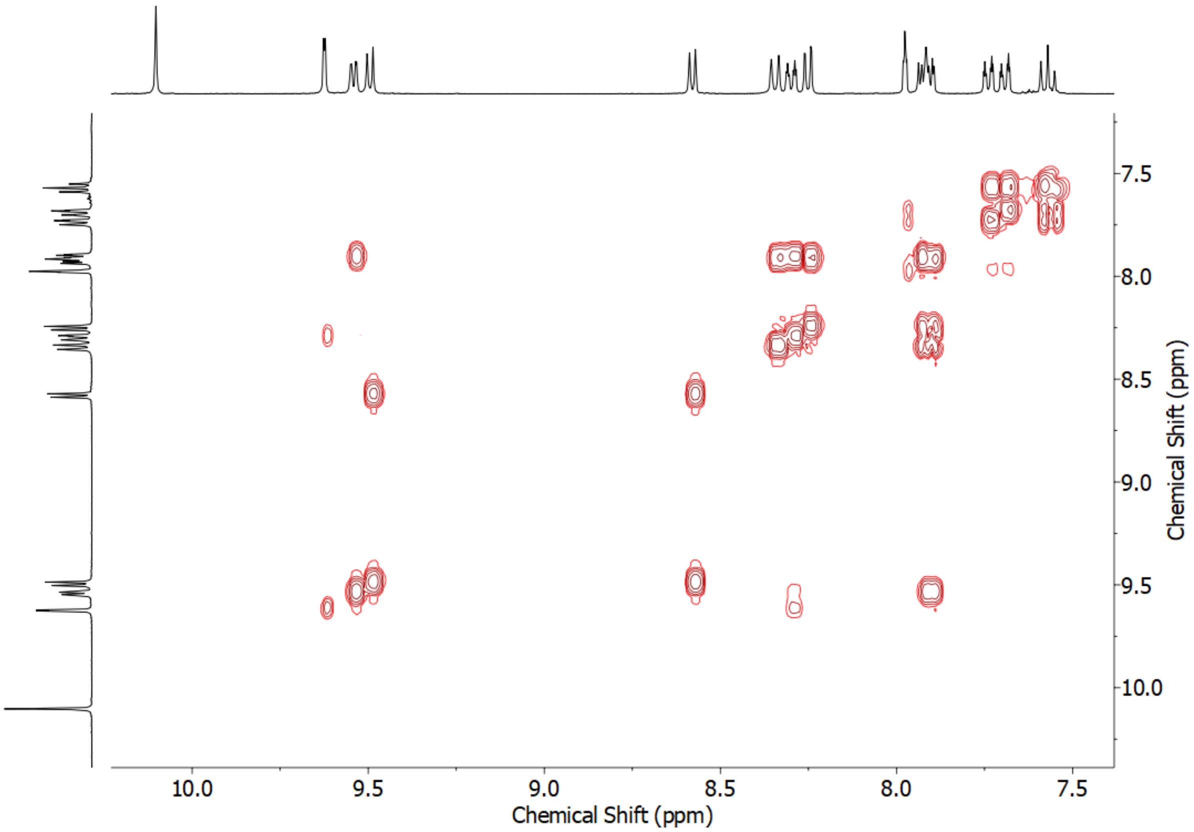

Figure S109 COSY NMR (d_6-DMSO) of $[Pd_2(\mathbf{4})_4](BF_4)_4$.

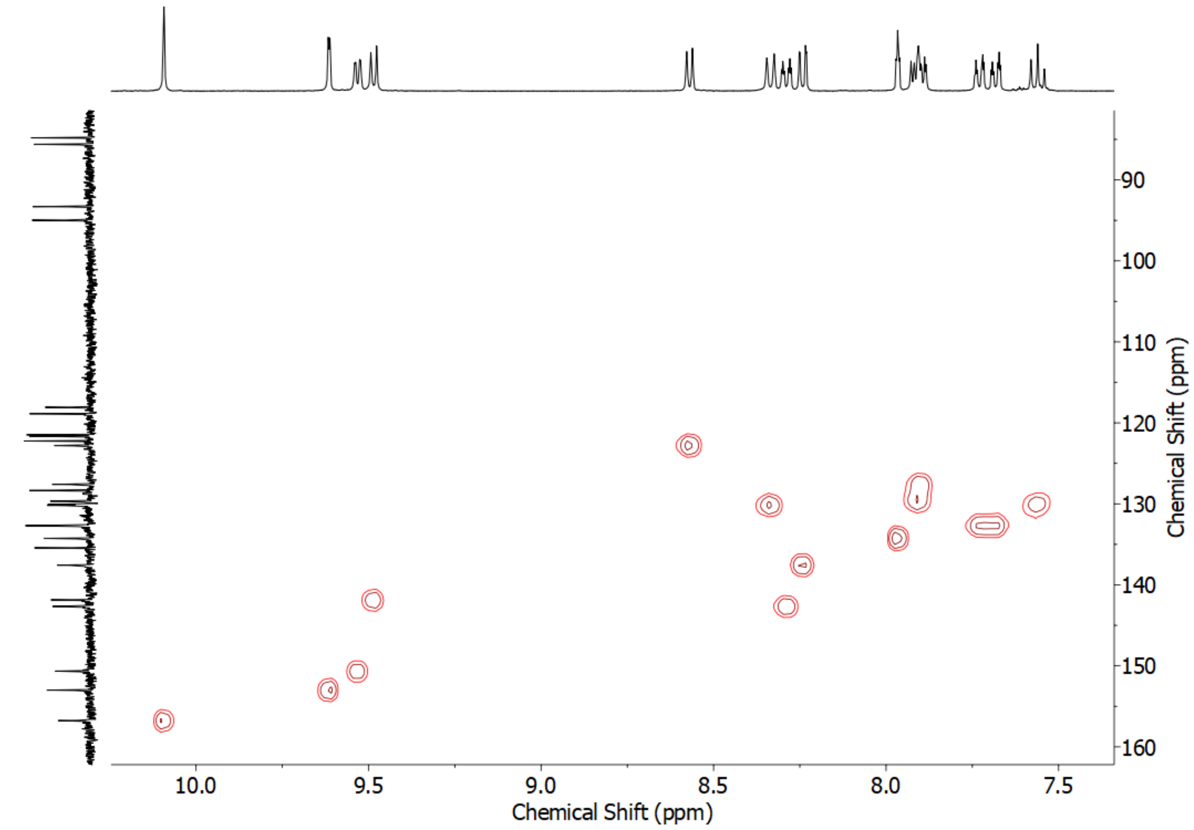

Figure S110 HSQC NMR (d_6-DMSO) of $[Pd_2(\mathbf{4})_4](BF_4)_4$.

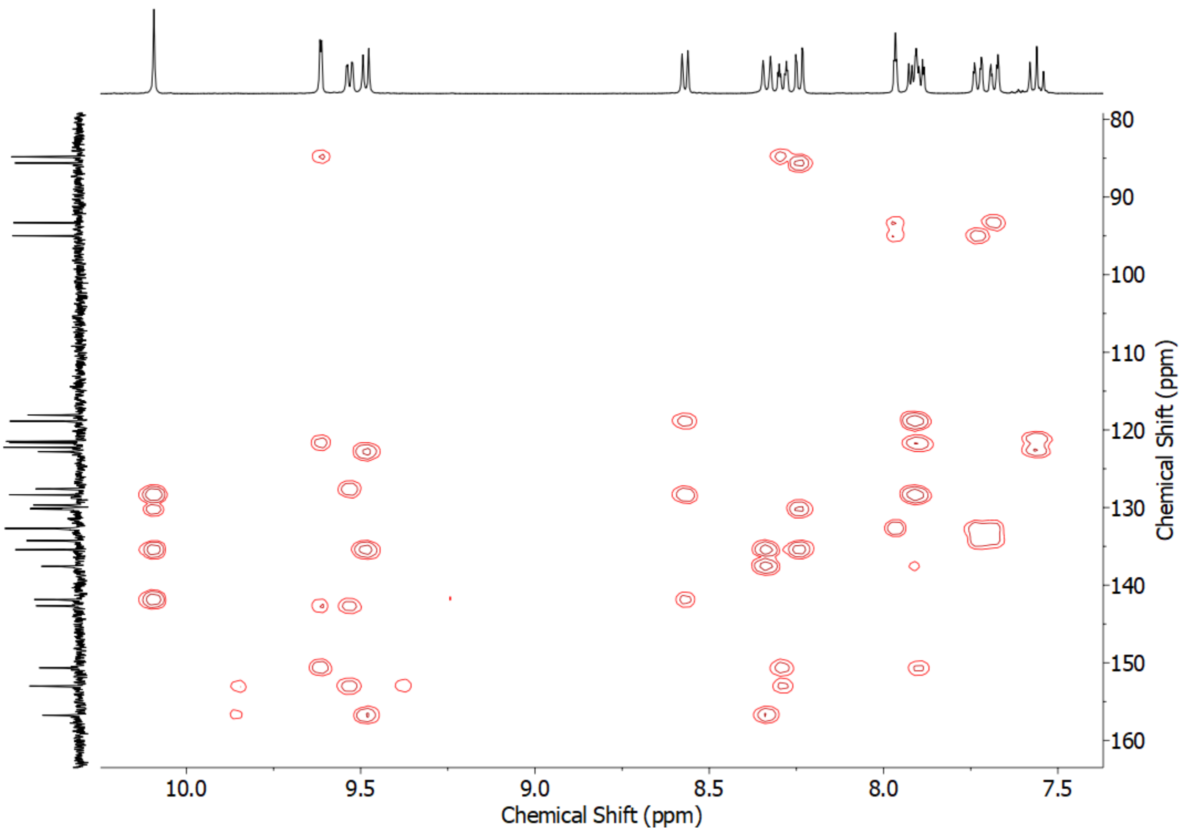

Figure S111 HMBC NMR (d_6-DMSO) of $[Pd_2(\mathbf{4})_4](BF_4)_4$.

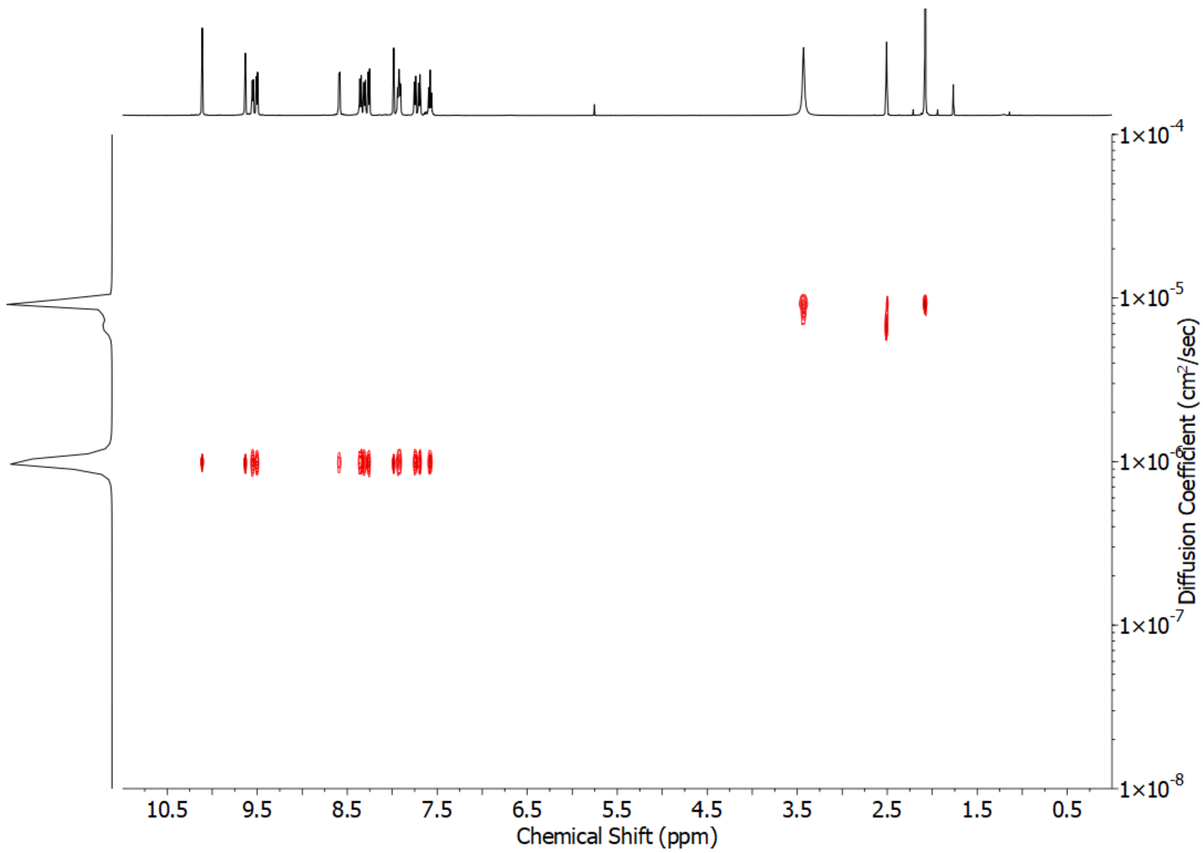

Figure S112 DOSY NMR (d_6-DMSO, 500 MHz) of $[Pd_2(\mathbf{4})_4](BF_4)_4$.

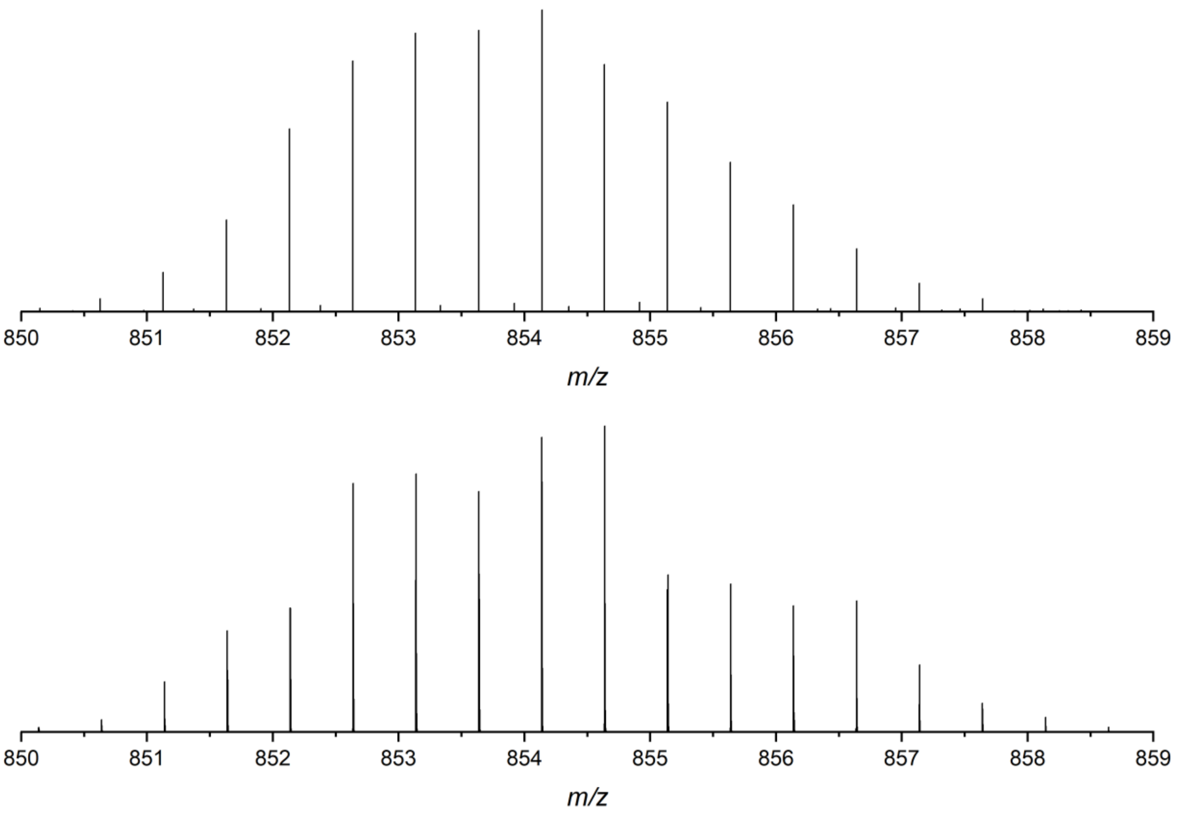

Figure S113 Calculated (top) and experimental (bottom) isotopic patterns for $\{[Pd_2(\mathbf{4})_4](BF_4)_2\}^{2+}$.

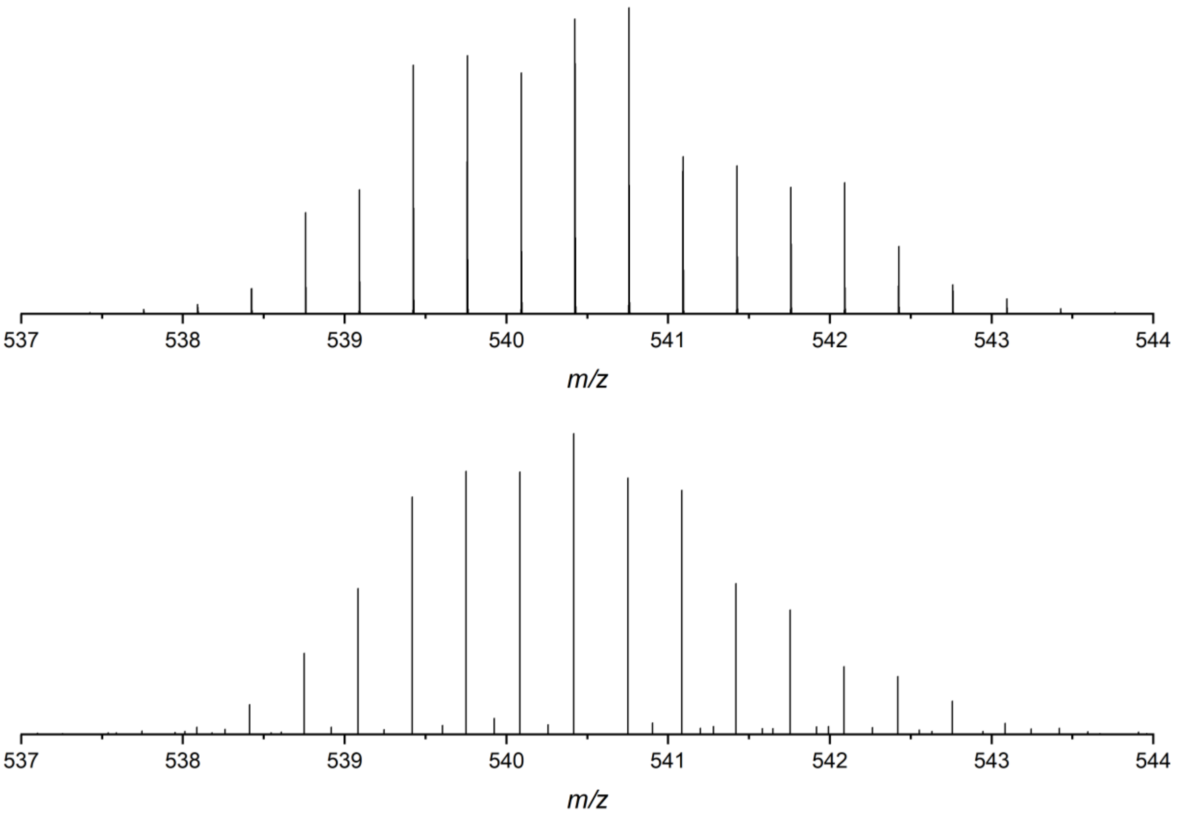

Figure S114 Calculated (top) and experimental (bottom) isotopic patterns for $\{[Pd_2(\mathbf{4})_4](BF_4)\}^{3+}$.

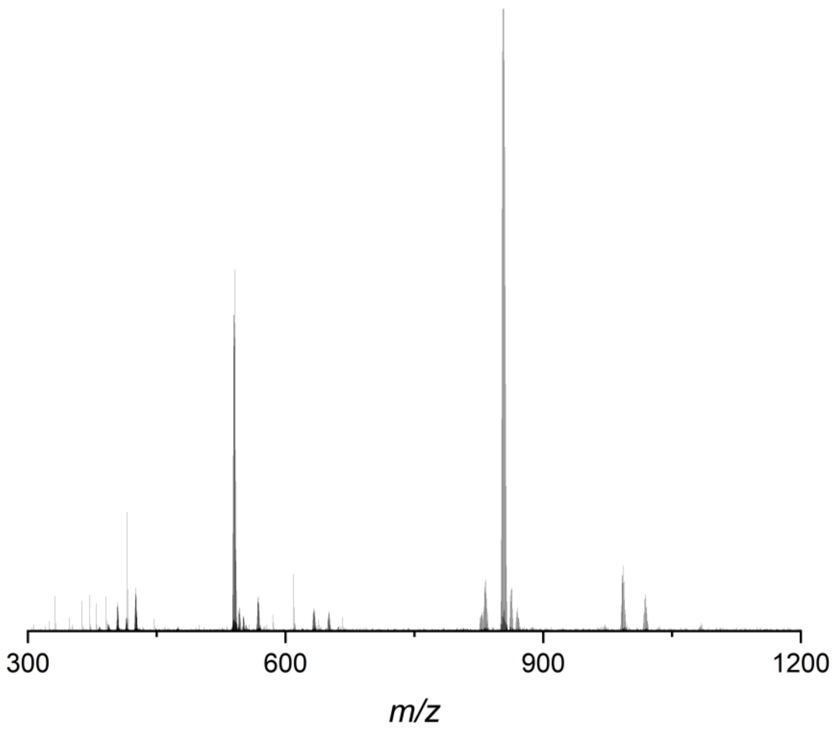

Figure S115 ESI-MS of $[Pd_2(\mathbf{4})_4](BF_4)_4$.

$[Pd_2(5)_4](BF_4)_4$

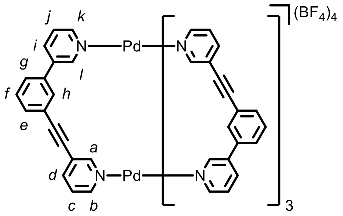

5 (7.7 mg, 0.030 mmol, 2 eq.) and $[Pd(CH_3CN)_4](BF_4)_2$ (6.7 mg, 0.015 mmol, 1 eq.) were sonicated in d_6-DMSO (0.75 mL) until all solids were dissolved. After standing at rt for 4 d, a number of species were observed by ^{1}H NMR. Heating at 60 °C for several days had no effect on the speciation observed by ^{1}H NMR. Diffusion coefficient (500 MHz, d_6-DMSO) D: 1.16 × 10^{-10} m^2s^{-1}. ESI-MS m/z = 1497.23 $\{[Pd_2(C_{18}H_{12}N_2)_4](BF_4)_3\}^+$ calc. 1497.23; 705.11 $\{[Pd_2(C_{18}H_{12}N_2)_4](BF_4)_2\}^{2+}$ calc. 705.11.

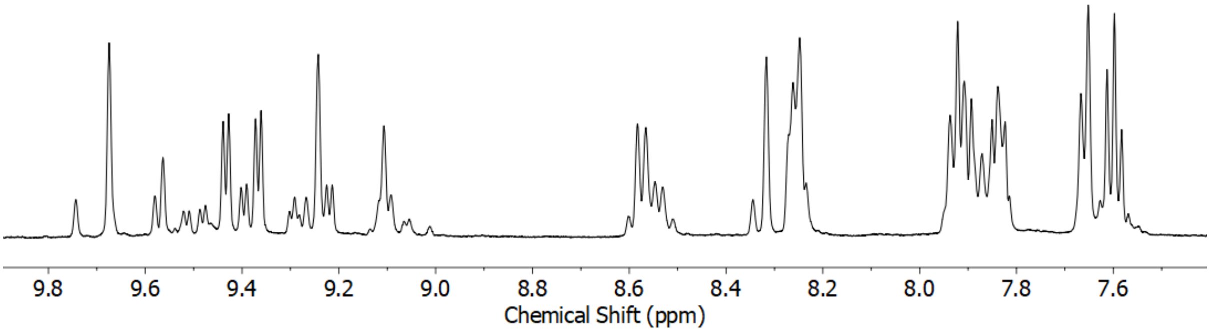

Figure S116 ^{1}H NMR (d_6-DMSO, 500 MHz) of $[Pd_2(\mathbf{5})_4](BF_4)_4$.

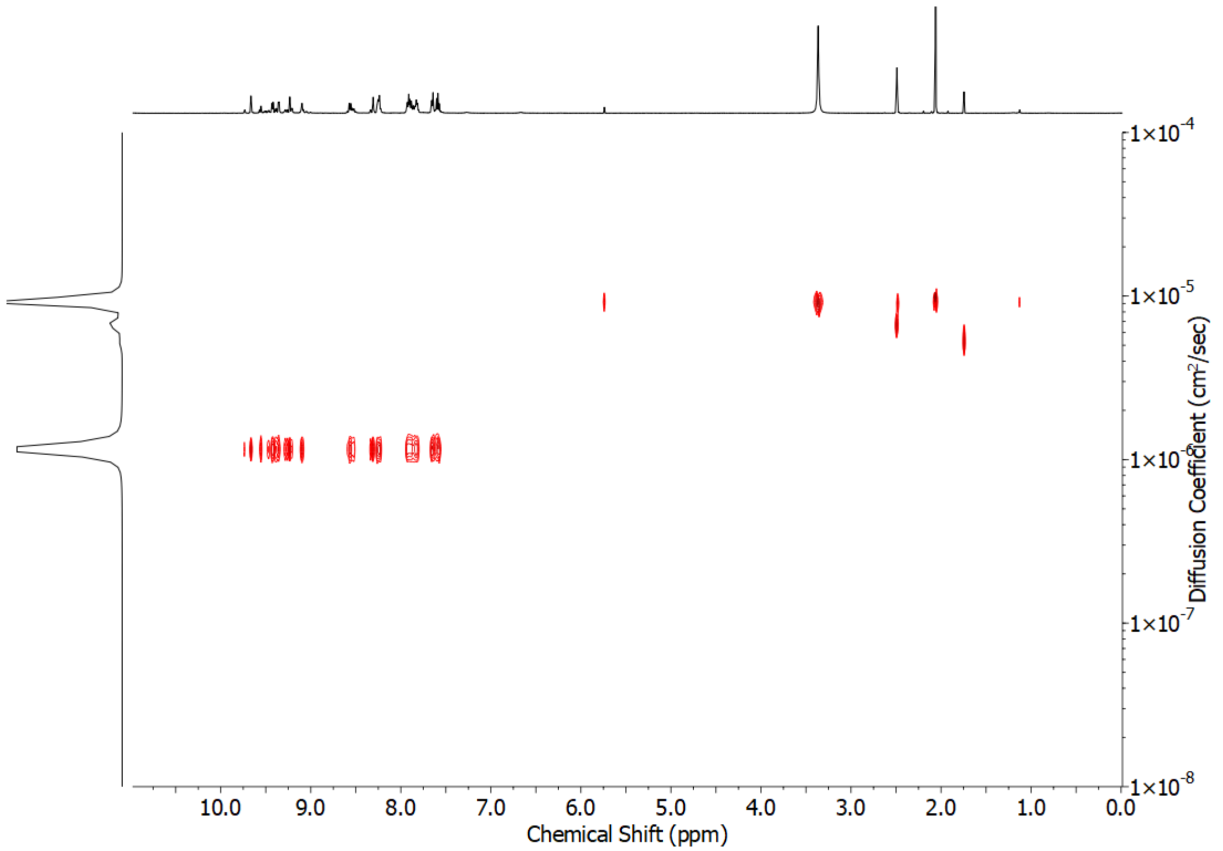

Figure S117 DOSY NMR (d_6-DMSO, 500 MHz) of $[Pd_2(\mathbf{5})_4](BF_4)_4$.

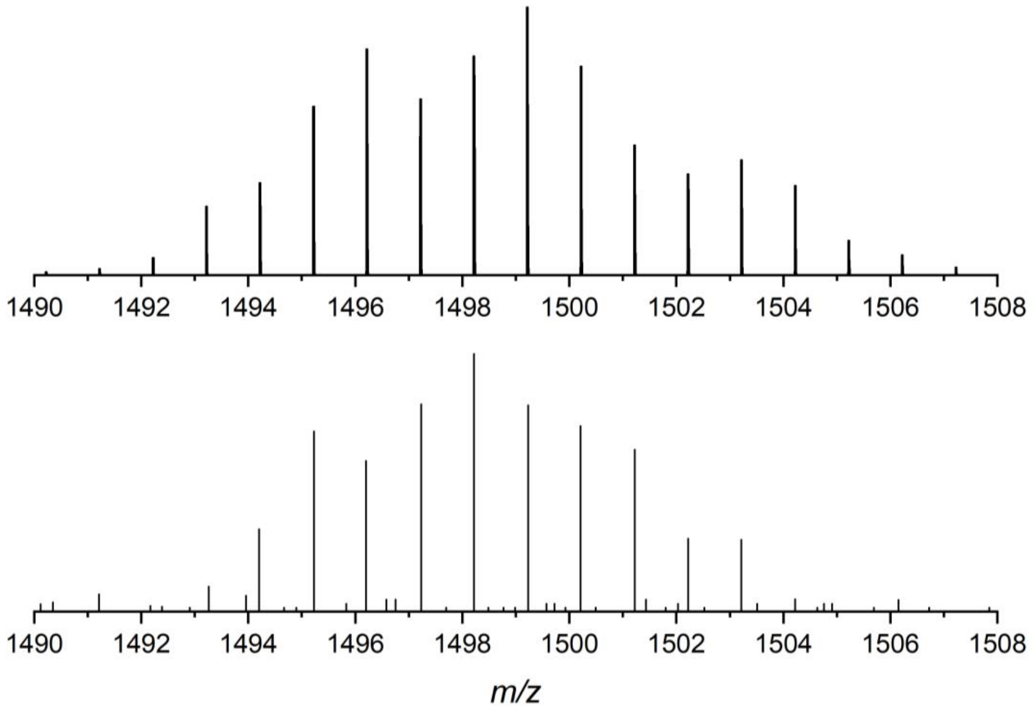

Figure S118 Calculated (top) and experimental (bottom) isotopic patterns for $\{[Pd_2(\mathbf{5})_4](BF_4)_3\}^+$.

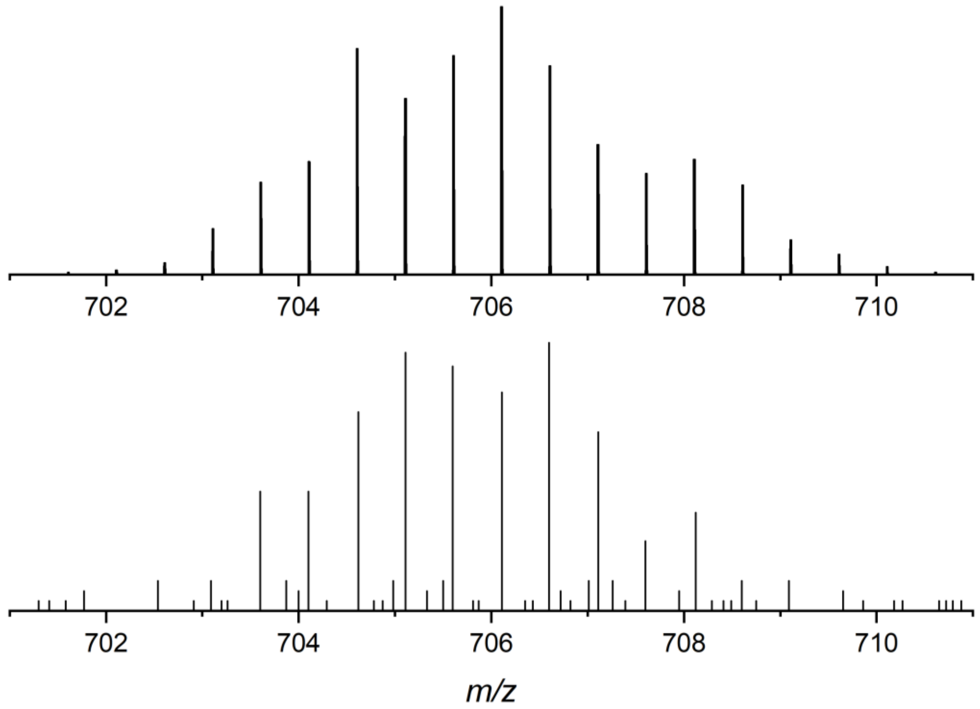

Figure S119 Calculated (top) and experimental (bottom) isotopic patterns for $\{[Pd_2(\mathbf{5})_4](BF_4)_2\}^{2+}$.

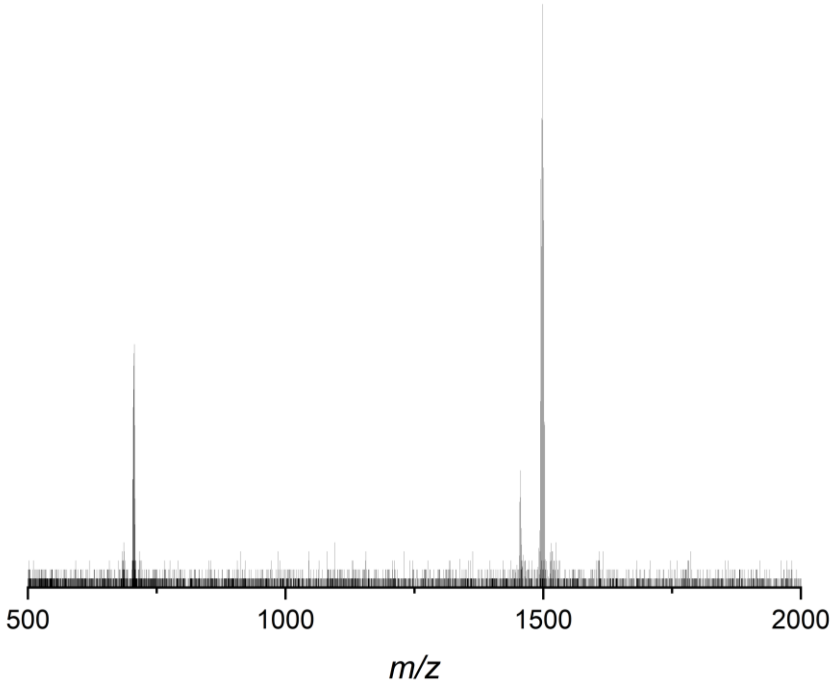

Figure S120 ESI-MS of $[Pd_2(\mathbf{5})_4](BF_4)_4$.

5 (7.7 mg, 0.030 mmol, 2 eq.) and $[Pd(CH_3CN)_4](BF_4)_2$ (6.7 mg, 0.015 mmol, 1 eq.) were sonicated in CD_3CN (0.75 mL) until all solids were dissolved. After standing at 60 °C for 1 d, a number of species were observed by ^1H NMR. Continued heating at 60 °C for several days had no effect on the speciation observed by ^1H NMR. Diffusion coefficient (500 MHz, CD_3CN) *D*: 8.50×10^{-10} m^2s^{-1}.

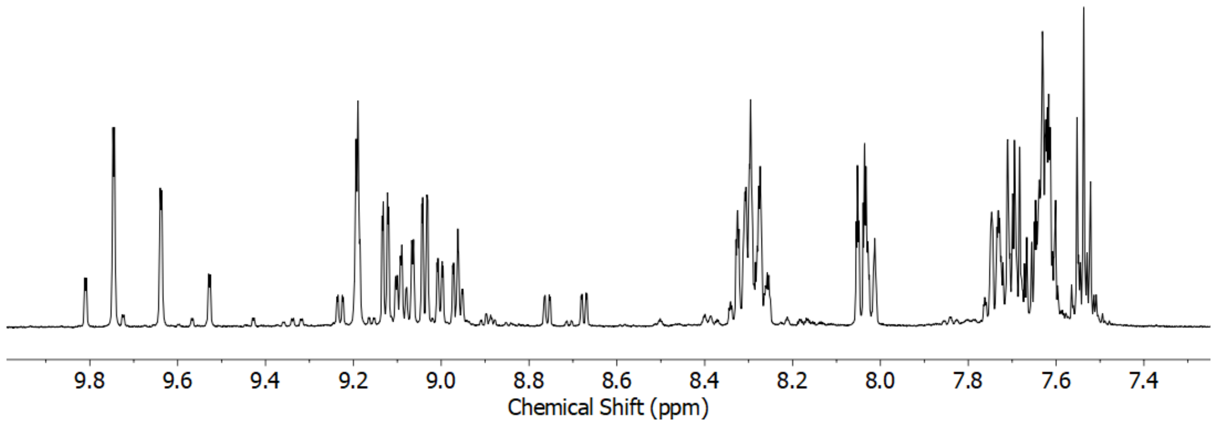

Figure S121 ^1H NMR (CD_3CN, 500 MHz) of $[Pd_2(\mathbf{5})_4](BF_4)_4$.

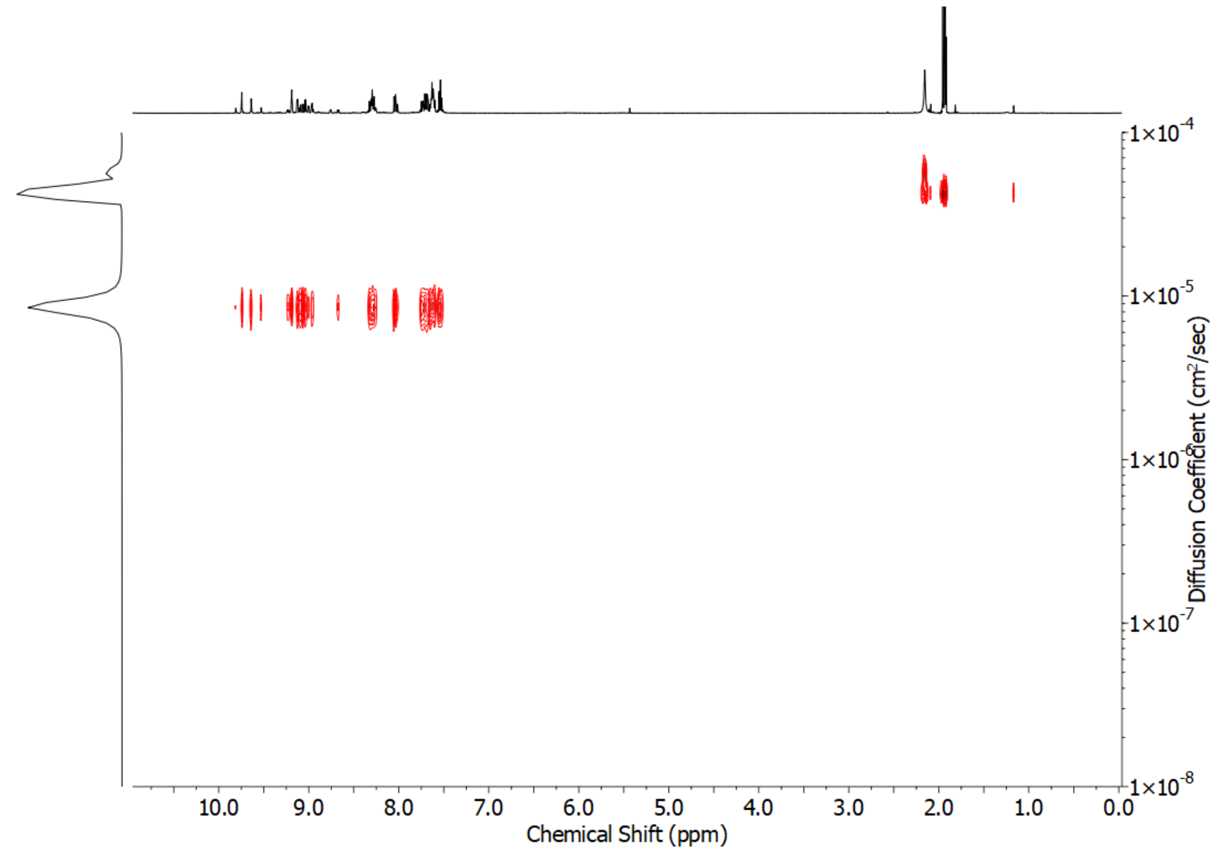

Figure S122 DOSY NMR (CD_3CN, 500 MHz) of $[Pd_2(\mathbf{5})_4](BF_4)_4$.

[Pd$_2$(6)$_4$](BF$_4$)$_4$

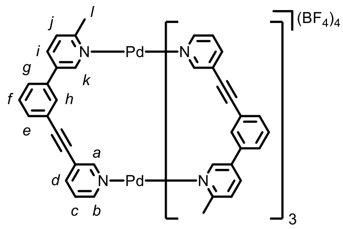

6 (8.1 mg, 0.030 mmol, 2 eq.) and [Pd(CH$_3$CN)$_4$](BF$_4$)$_2$ (6.7 mg, 0.015 mmol, 1 eq.) were sonicated in d_6-DMSO (0.75 mL) until all solids were dissolved. After standing at rt for 24 h, conversion to [Pd$_2$(**6**)$_4$](BF$_4$)$_4$ was observed by ^{1}H NMR. ^{1}H NMR (400 MHz, d_6-DMSO) δ: 9.29 (br. s, 4H, H$_a$), 9.15 (m, 4H, H$_b$), 9.09 (d, J = 1.8 Hz, 4H, H$_k$), 8.50 (dd, J = 8.4, 2.0 Hz, 4H, H$_i$), 8.28 (dt, J = 8.0, 1.4 Hz, 4H, H$_d$), 8.24 (br. s, 4H, H$_h$), 7.86 (dt, J = 7.8, 1.7 Hz, 4H, H$_g$), 7.78-7.73 (m, 8H, H$_c$, H$_j$), 7.64-7.56 (m, 8H, H$_e$, H$_f$), 3.30 (s, 12H, H$_l$). Diffusion coefficient (500 MHz, d_6-DMSO) D: 1.15 × 10^{-10} m^{2}s^{-1}. ^{13}C NMR (101 MHz, d_6-DMSO) δ: 160.5, 152.4, 151.8, 146.9, 143.2, 139.1, 134.7, 134.3, 132.4, 130.3, 128.8 (×2), 128.5, 127.1, 122.9, 121.9, 94.5, 84.2, 25.9. ESI-MS m/z = 459.76 {[Pd$_2$(C$_{19}$H$_{14}$N$_2$)$_4$](BF$_4$)}$^{3+}$ calc. 459.76; 733.14 {[Pd$_2$(C$_{19}$H$_{14}$N$_2$)$_4$](BF$_4$)$_2$}$^{2+}$ calc. 733.14; 1553.30 {[Pd$_2$(C$_{19}$H$_{14}$N$_2$)$_4$](BF$_4$)$_3$}$^{+}$ calc. 1553.28.

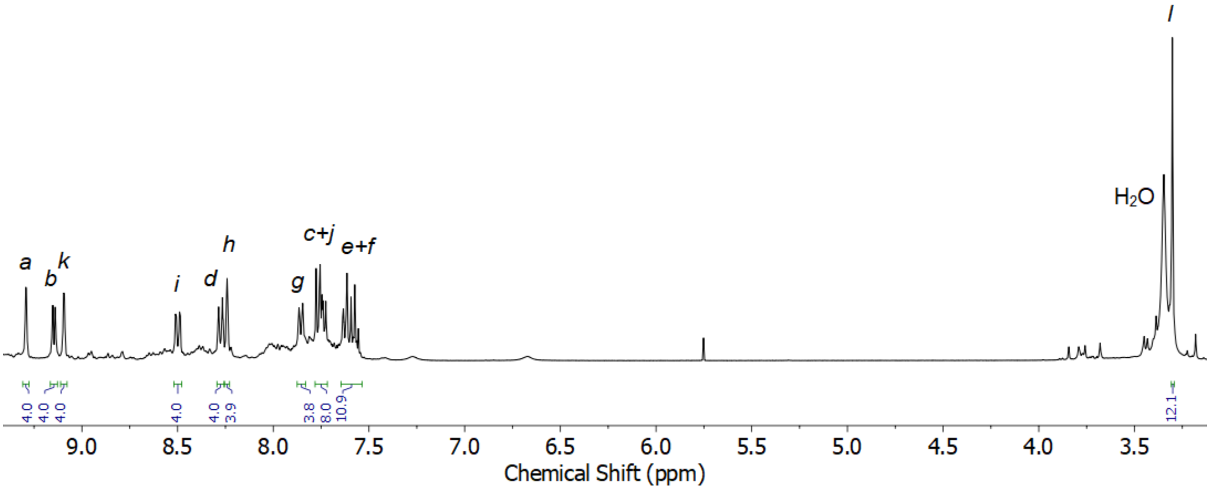

Figure S123 ^{1}H NMR (d_6-DMSO, 400 MHz) of [Pd$_2$(**6**)$_4$](BF$_4$)$_4$.

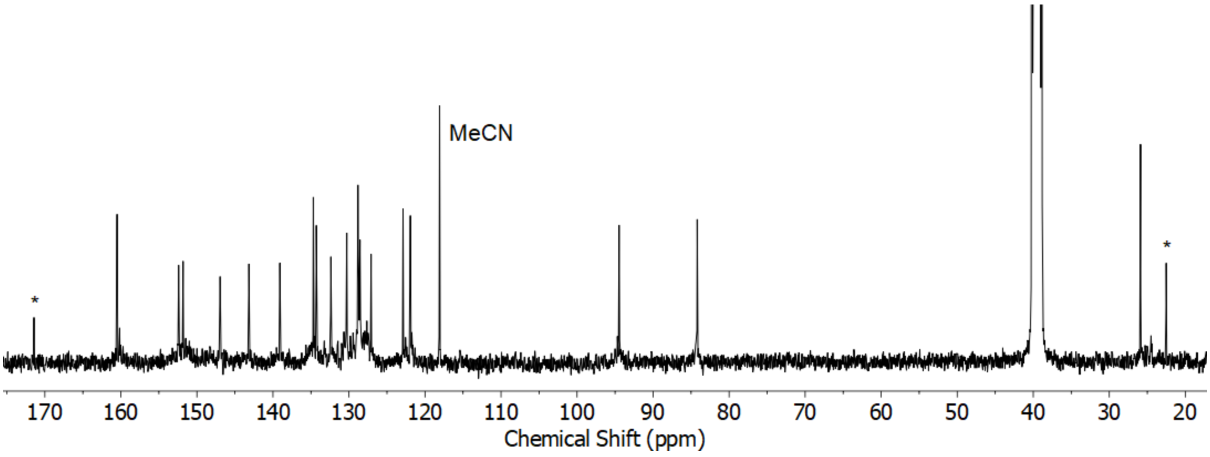

Figure S124 ^{13}C NMR (d_6-DMSO, 101 MHz) of [Pd$_2$(**6**)$_4$](BF$_4$)$_4$.

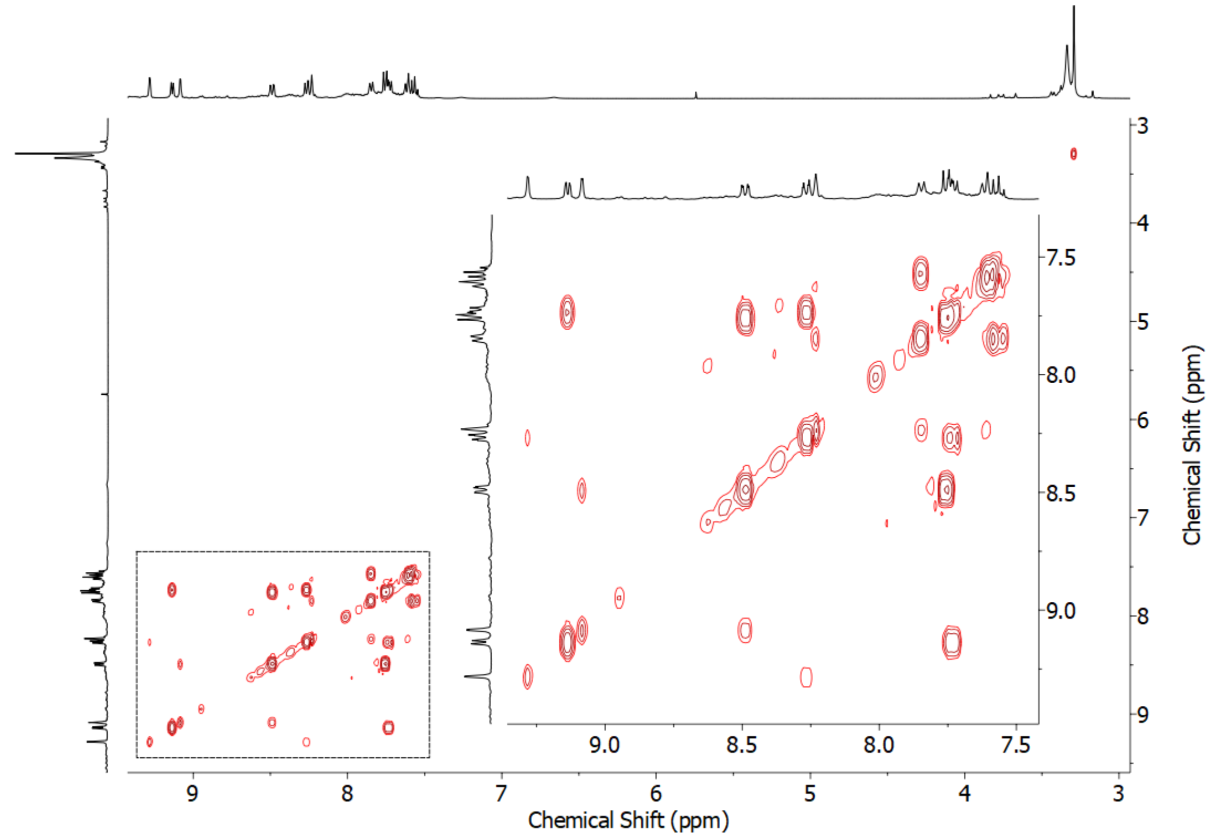

Figure S125 COSY NMR (d_6-DMSO) of $[Pd_2(\mathbf{6})_4](BF_4)_4$.

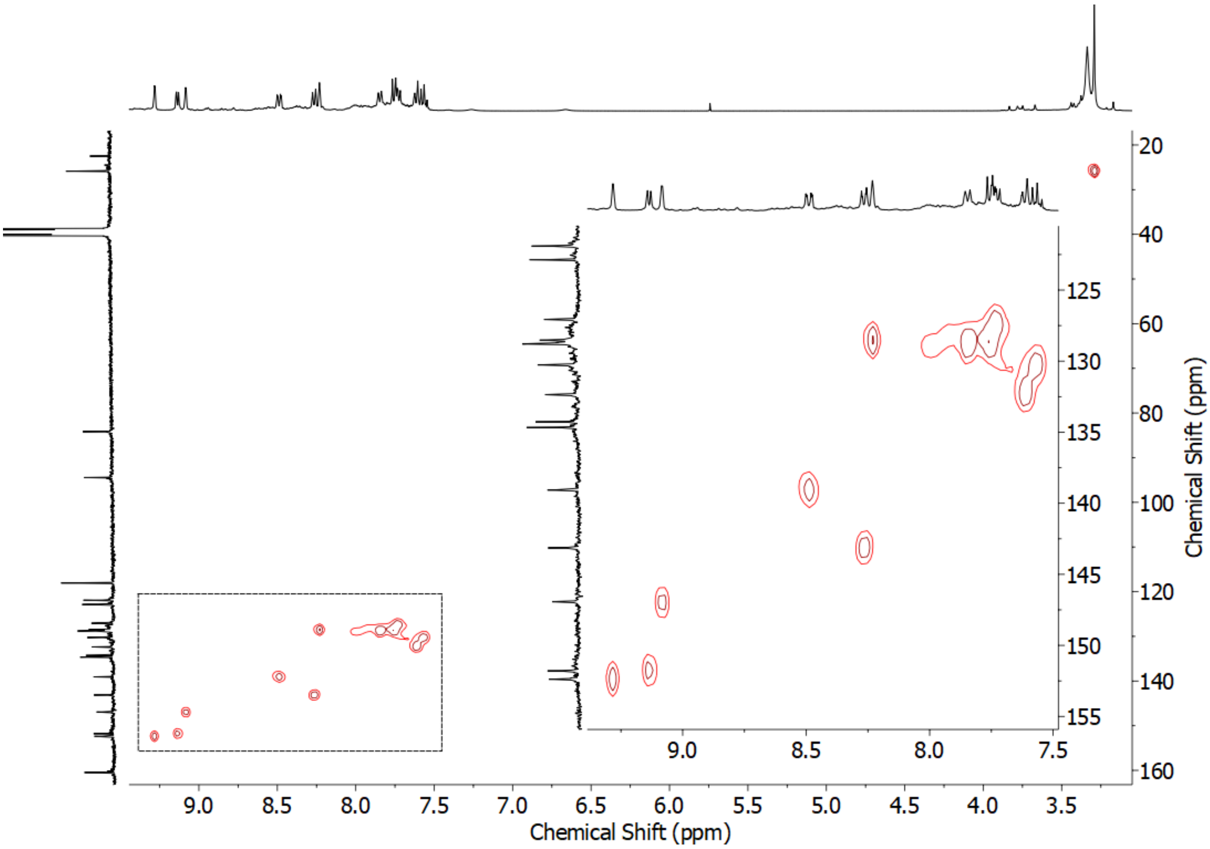

Figure S126 HSQC NMR (d_6-DMSO) of $[Pd_2(\mathbf{6})_4](BF_4)_4$.

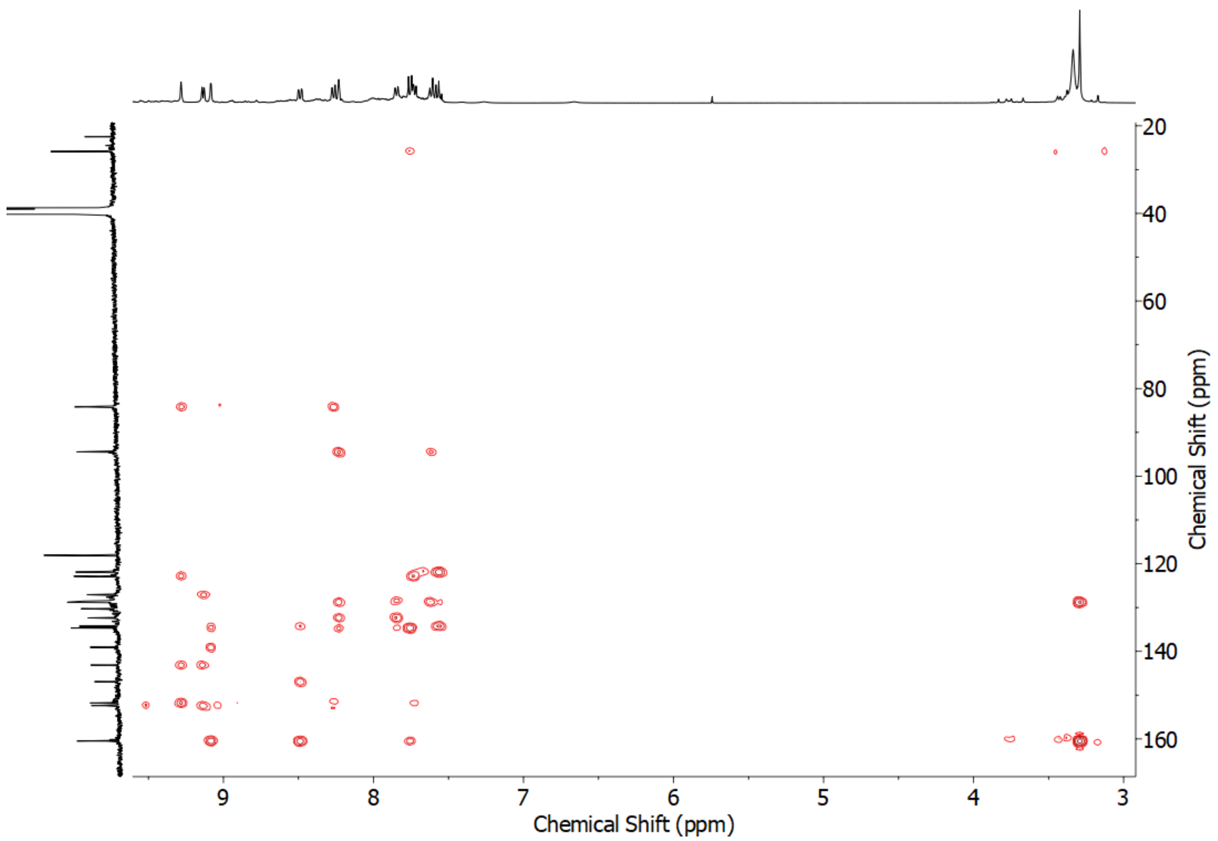

Figure S127 HMBC NMR (d_6-DMSO) of $[Pd_2(\mathbf{6})_4](BF_4)_4$.

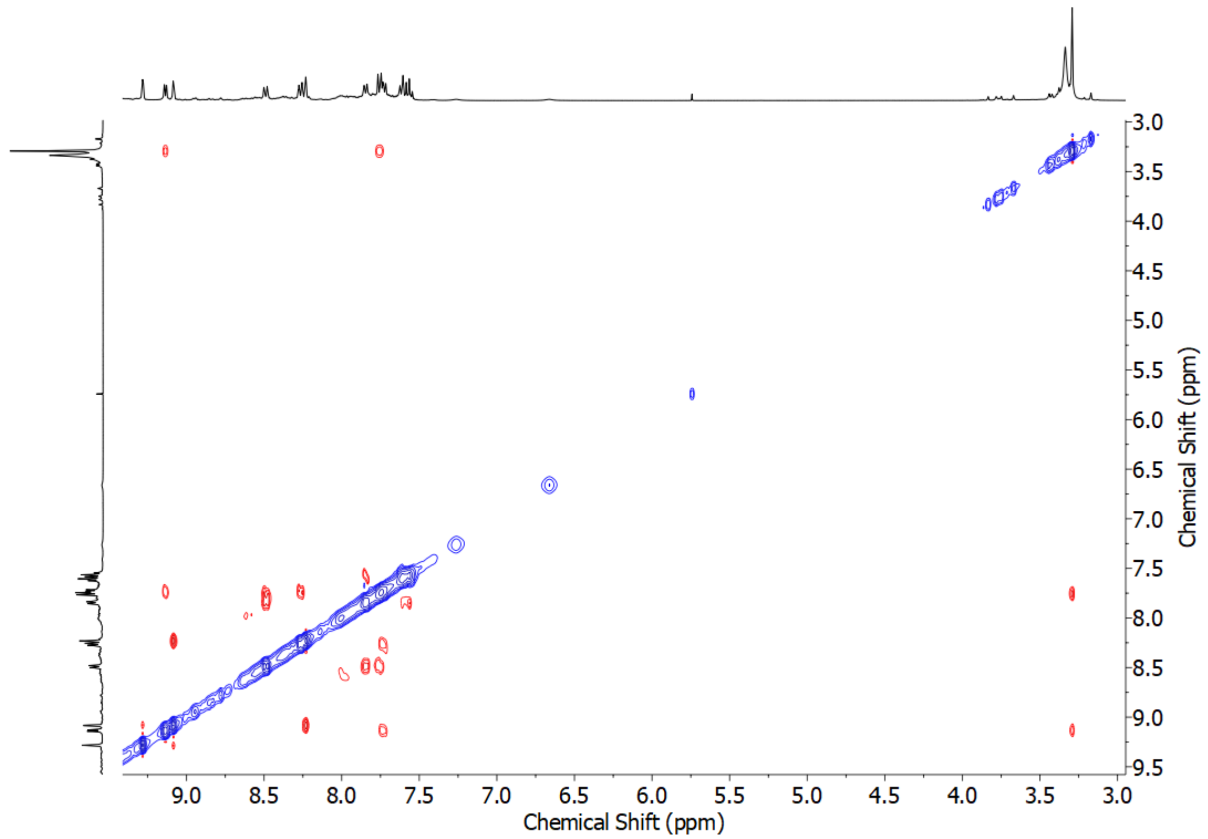

Figure S128 ROESY NMR (d_6-DMSO) of $[Pd_2(\mathbf{6})_4](BF_4)_4$.

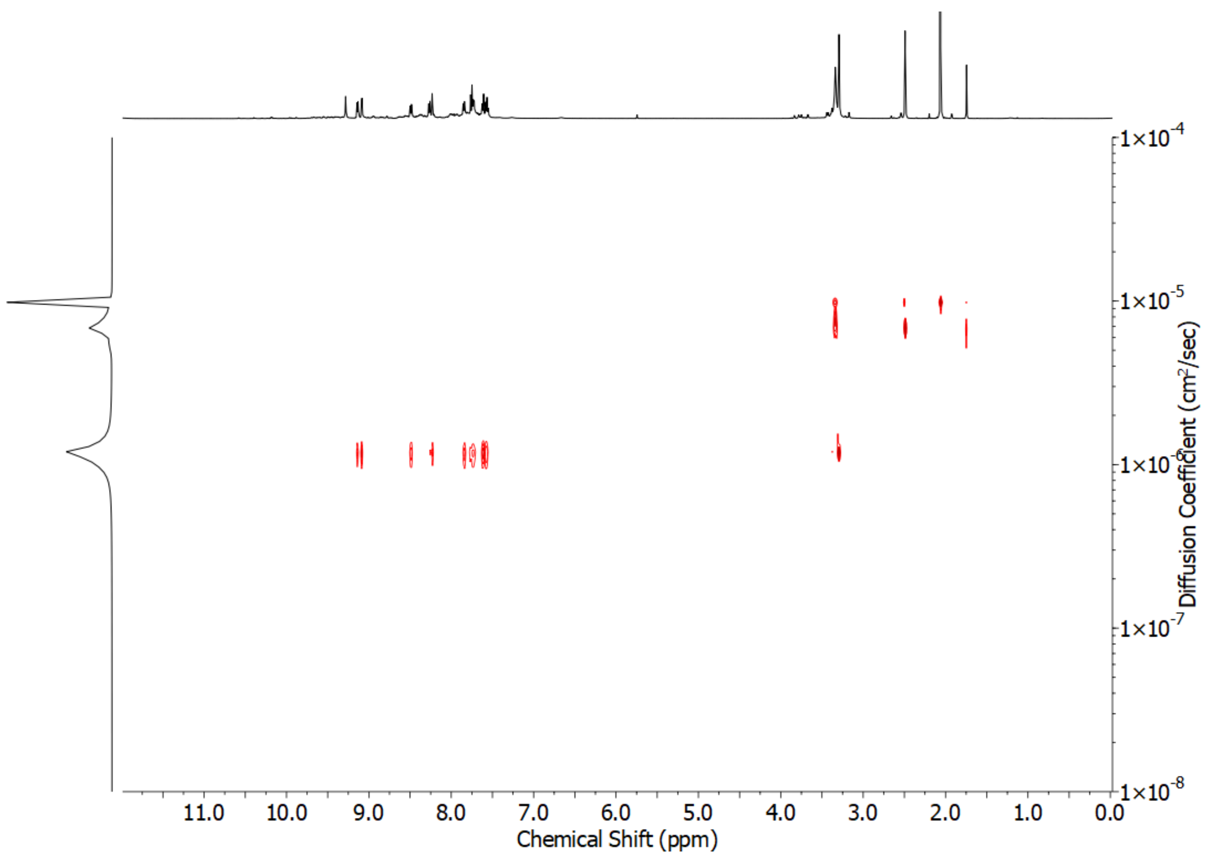

Figure S129 DOSY NMR (d_6-DMSO, 500 MHz) of $[Pd_2(\mathbf{6})_4](BF_4)_4$.

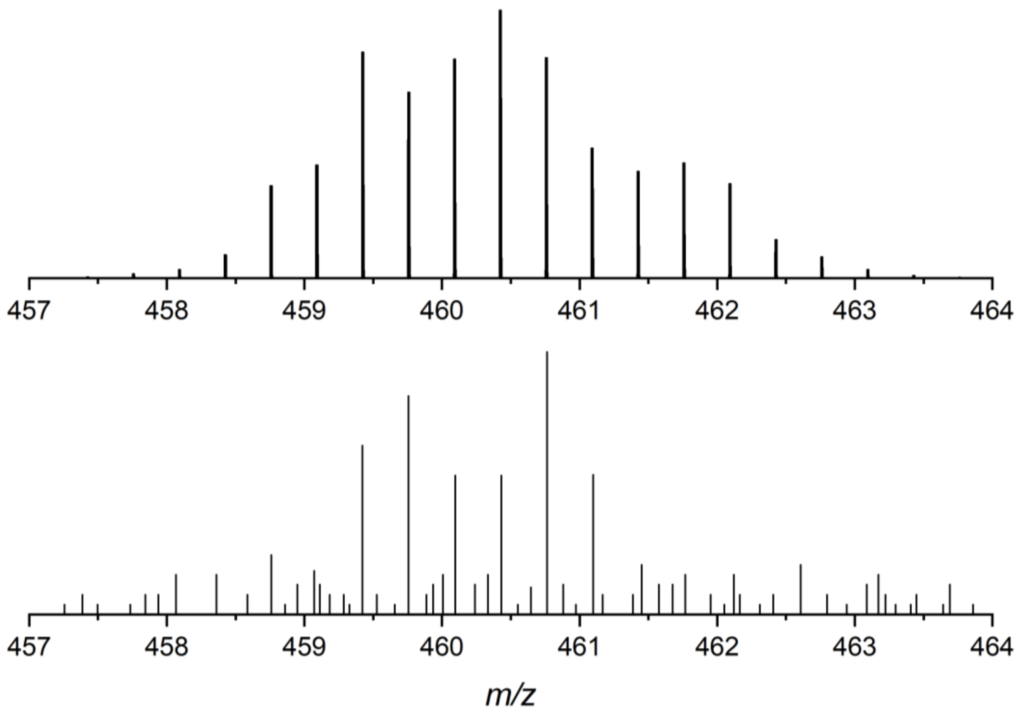

Figure S130 Calculated (top) and experimental (bottom) isotopic patterns for $\{[Pd_2(\mathbf{6})_4](BF_4)\}^{3+}$.

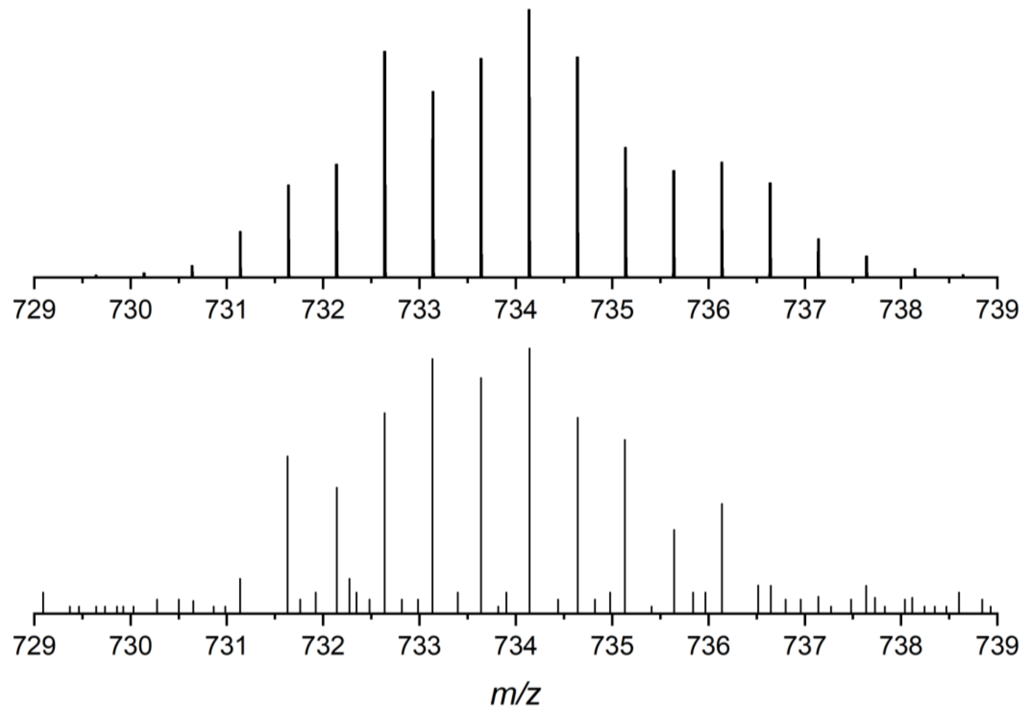

Figure S131 Calculated (top) and experimental (bottom) isotopic patterns for $\{[Pd_2(\mathbf{6})_4](BF_4)_2\}^{2+}$.

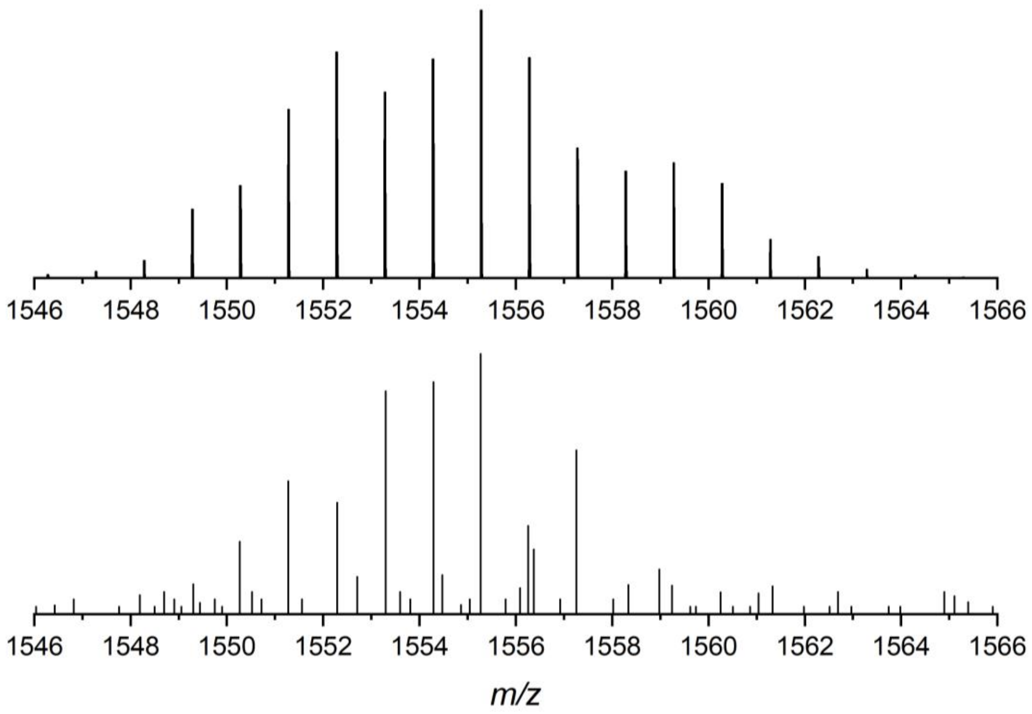

Figure S132 Calculated (top) and experimental (bottom) isotopic patterns for $\{[Pd_2(\mathbf{6})_4](BF_4)_3\}^{+}$.

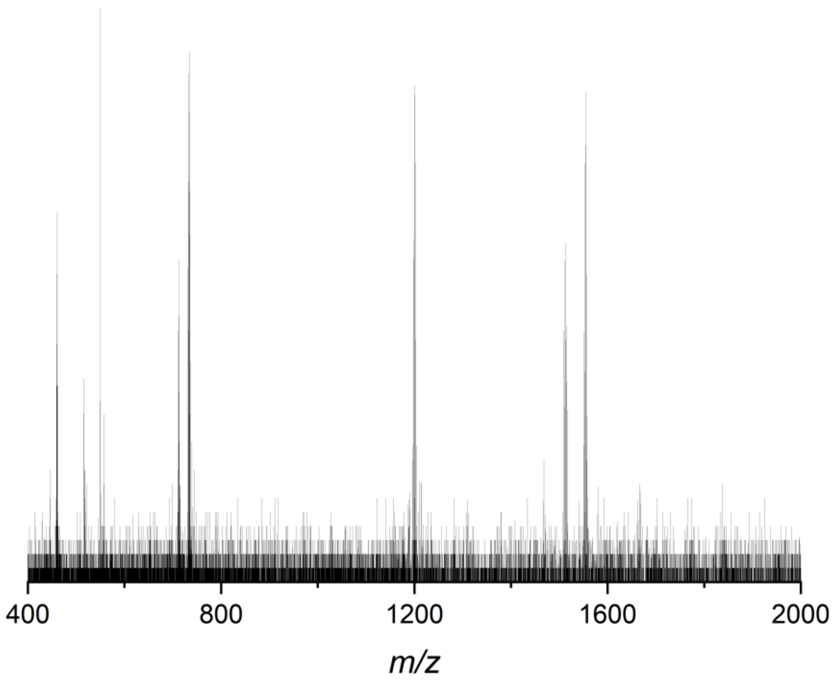

Figure S133 ESI-MS of $[Pd_2(\mathbf{6})_4](BF_4)_4$.

X-ray Crystallographic Data

X-ray crystal structure of *cis*-$[Pd_2(2)_4](BF_4)_4$

2 (12.3 mg, 0.040 mmol) and $[Pd(CH_3CN)_4](BF_4)_2$ (8.9 mg, 0.020 mmol) were stirred in DMF (3 mL) at rt for 2 h. X-ray quality crystals were grown by vapour diffusion of Et_2O into the solution over a period of several days.

Crystal data for cis-$[Pd_2(\mathbf{2})_4](BF_4)_4$: $[C_{88}H_{56}N_8Pd_2](BF_4)_4{\cdot}4(C_4H_{10}O)$, M = 2081.92, monoclinic, $I2/a$ (no. 15), a = 28.3600(4), b = 12.35285(17), c = 27.6660(4) Å, β = 94.6541(14)°, V = 9660.2(2) Å^3, Z = 4 [C_i symmetry], D_c = 1.431 g cm^{-3}, μ(Cu-Kα) = 3.749 mm^{-1}, T = 173 K, colourless platy needles, Agilent Xcalibur PX Ultra A diffractometer; 9259 independent measured reflections (R_{int} = 0.0268), F^2 refinement,[2,3] R_1(obs) = 0.0419, wR_2(all) = 0.1190, 7879 independent observed absorption-corrected reflections [$|F_o| > 4\sigma(|F_o|)$, completeness to θ_{full}(67.7°) = 98.7%], 555 parameters. CCDC 1946272.

The N22- and N52-based pyridyl rings, and the B60-based tetrafluoroborate anion, in the structure of *cis*-$[Pd_2(\mathbf{2})_4](BF_4)_4$ were all found to be disordered. In each case two orientations were identified, of *ca*. 67:33, 68:32, and 69:31% occupancy respectively, their geometries were optimised, the thermal parameters of adjacent atoms were restrained to be similar, and only the non-hydrogen atoms of the major occupancy orientations were refined anisotropically (those of the minor occupancy orientations were refined isotropically).

The included solvent was found to be highly disordered, and the best approach to handling this diffuse electron density was found to be the SQUEEZE routine of PLATON.[4] This suggested a total of 662 electrons per unit cell, equivalent to 165.5 electrons per complex. Before the use of SQUEEZE the solvent clearly resembled diethyl ether ($C_4H_{10}O$, 42 electrons), and 4 dichloromethane molecules corresponds to 168 electrons, so this was used as the solvent present. As a result, the atom list for the asymmetric unit is low by $2(C_4H_{10}O) = C_8H_{20}O_2$ (and that for the unit cell low by $C_{64}H_{160}O_{16}$) compared to what is actually presumed to be present.

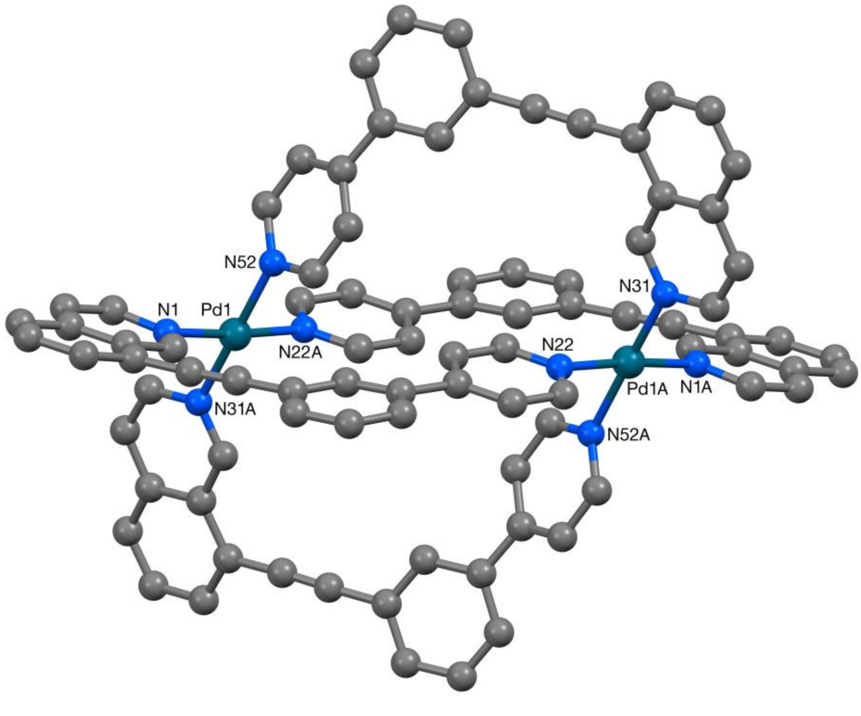

Figure S134 The structure of the C_i-symmetric cation present in the crystal of *cis*-$[Pd_2(\mathbf{2})_4](BF_4)_4$.

X-ray crystal structure of *cis*-[Pd_2(4)$_4$](BF_4)$_4$

4 (9.9 mg, 0.030 mmol, 2 eq.) and [Pd(CH_3CN)$_4$](BF_4)$_2$ (6.7 mg, 0.015 mmol, 1 eq.) were sonicated in DMF (3 mL) until all solids were dissolved. After stirring at rt overnight, X-ray quality crystals were grown by vapour diffusion of Et_2O into the solution over a period of several days.

Crystal data for cis-[Pd_2(**4**)$_4$](BF_4)$_4$: [$C_{96}H_{56}N_8Pd_2$](BF_4)$_4$·9(C_3H_7NO), *M* = 2539.38, monoclinic, *C*2/*c* (no. 15), *a* = 23.2193(9), *b* = 15.8896(5), *c* = 38.7298(15) Å, β = 112.281(5)°, *V* = 13222.3(9) Å^3, *Z* = 4 [C_i symmetry], D_c = 1.276 g cm^{-3}, μ(Cu-Kα) = 2.884 mm^{-1}, *T* = 173 K, colourless tablets, Agilent Xcalibur PX Ultra A diffractometer; 14084 independent measured reflections (R_{int} = 0.0507), F^2 refinement,[2,3] R_1(obs) = 0.0994, wR_2(all) = 0.2743, 12479 independent observed absorption-corrected reflections [$|F_o| > 4\sigma(|F_o|)$, completeness to θ_{full}(67.7°) = 98.7%], 479 parameters. CCDC 1946273.

The crystal of *cis*-[Pd_2(**4**)$_4$](BF_4)$_4$ that was studied was found to be a two component twin in a *ca.* 62:38 ratio, with the two lattices related by the approximate twin law [1.00 0.00 0.00 0.00 –1.00 0.00 –0.74 –0.01 –1.00].

Both the presumed tetrafluoroborate anions and the included solvent were found to be highly disordered, and the best approach to handling this diffuse electron density was found to be the SQUEEZE routine of PLATON.[4] This suggested a total of 2090 electrons per unit cell, equivalent to 522.5 electrons per complex. Before the use of SQUEEZE, one tetrafluoroborate anion (BF_4, 41 electrons) and one dimethylformamide solvent molecule (C_3H_7NO, 40 electrons) were identified amongst the diffuse electron density. Removing the electron density of the presumed four tetrafluoroborate anions per complex leaves 522.5 – (4 x 41) = 358.5 electrons for the solvent; 9 dimethylformamide molecules corresponds to 360 electrons, so this was used as the solvent present. As a result, the atom list for the asymmetric unit is low by 2(BF4) + 4.5(C_3H_7NO) = $C_{13.5}H_{31.5}B_2F_8N_{4.5}O_{4.5}$ (and that for the unit cell low by $C_{108}H_{252}B_{16}F_{64}N_{36}O_{36}$) compared to what is actually presumed to be present.

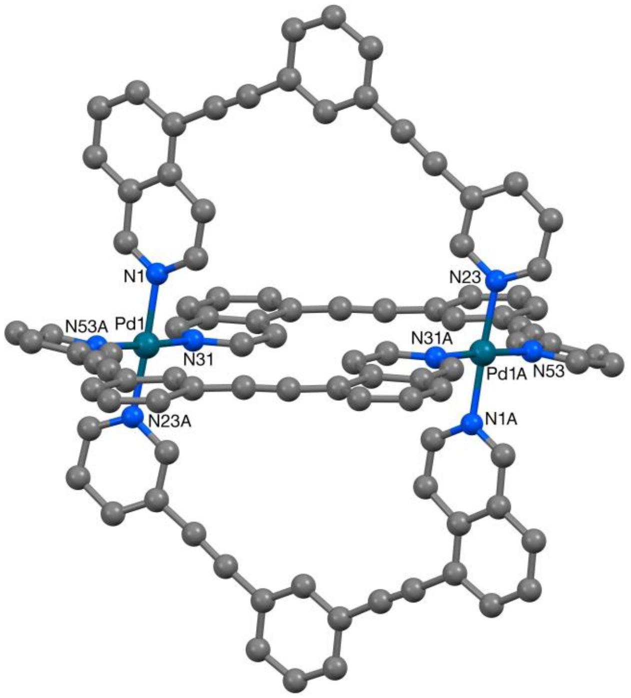

Figure S135 The structure of the C_i-symmetric cation present in the crystal of *cis*-[Pd_2(**4**)$_4$](BF_4)$_4$.

Density Functional Theory Calculations

All models were constructed using Avogadro[5] starting from the crystal structures reported herein. The semi-empirical method GFN2-xTB[6] was used to geometry optimise all structures in implicit solvent before optimisation using density functional theory (DFT). The xTB family of functionals have been shown to reproduce the geometries of metal-containing supramolecular architectures.[7] DFT optimisations were carried out using Gaussian 16.[8] All optimisations were carried out in dimethyl sulfoxide (DMSO) using the polarizable continuum model (PCM), unless stated otherwise. All geometries were optimised at the B3LYP/6-31G(d) level of theory with the D3 empirical dispersion correction.[9] The Stuttgart-Dresden (SDD) effective core potentials were used for the Pd^{2+} centres. Each cage has a charge of 4 and a multiplicity of 1. A similar approach has been used by Clever and co-workers on similar cage systems.[10] The total electronic energies are reported in all cases (Tables S1 and S2). Numerical frequency calculations were performed on all optimised structures to confirm that the optimised structure was a minimum. However, there was at most one small negative frequency in some cases. We note that the accuracy of the Hessian calculations was not sufficient in all cases to reliably use the results of the thermochemistry calculations. The geometry of *cis*-[Pd_2(**4**)$_4$] optimised with DFT matches well with the solid-state structure (Fig. S127).

Input files and output files for DFT calculations can be accessed at:
https://github.com/andrewtarzia/citable_data/tree/master/lewis_2019

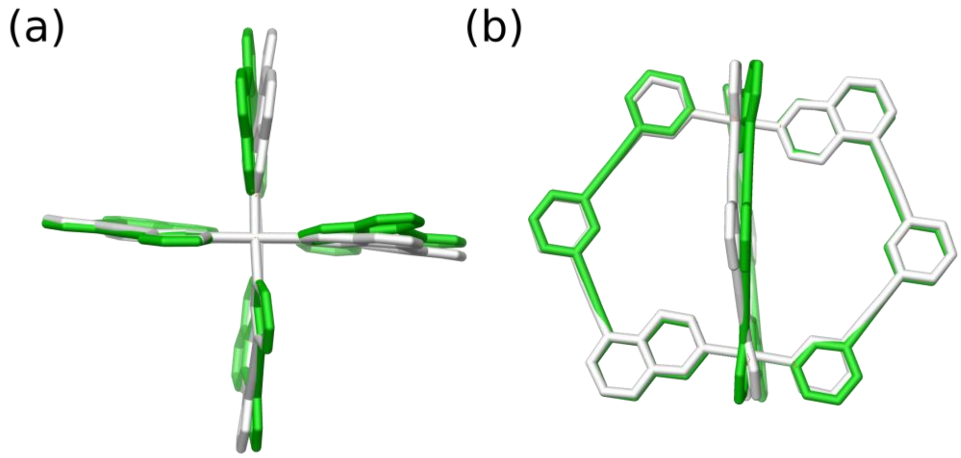

Figure S136 Overlay of the solid-state (green) and DFT-optimised structure (white) of *cis*-[Pd_2(**4**)$_4$] (a) along and (b) orthogonal to the Pd-Pd axis.

Table S1 Relative energy (the minimum of each *cis-trans* pair is set to 0 kJ mol^{-1}) and the magnitude of the dipole moment of *cis* (**C**) and *trans* (**D**) isomers of the cages formed from ligand **1** using two implicit solvent models.

Ligand	Isomer	Solvent	Relative energy (kJ mol^{-1})	Dipole moment (Debye)
**1**	**C**	Acetonitrile	6.1	0.40
**1**	**D**	Acetonitrile	0.0	0.00
**1**	**C**	DMSO	6.1	0.39
**1**	**D**	DMSO	0.0	0.00

Table S2 Relative energy (the minimum of each set of isomers is set to 0 kJ mol^{-1}) and the magnitude of the dipole moment of optimised cage structures formed from ligands **4**, **5** and **6**.

Ligand	Isomer	Relative energy (kJ mol^{-1})	Dipole moment (Debye)
**4**	**A**	26.8	12.60
**4**	**B**	4.4	6.20
**4**	**C**	0.0	0.96
**4**	**D**	8.9	0.06
**5**	**A**	18.0	2.94
**5**	**B**	0.1	1.12
**5**	**C**	0.0	1.09
**5**	**D**	12.0	2.85
**6**	**C**	0.0	1.34
**6**	**D**	12.2	2.20

Ligand Distortion Measurements

Ligand distortion measures (Fig. S128) used in this work were adapted from related work reported by Lusby, Duarte and co-workers.[11]

The optimised pore diameters were calculated using pyWindow[12] (Python scripts are made available). All guests, ions and solvent molecules were removed from the cages extracted from SCXRD structures for pyWindow analysis.

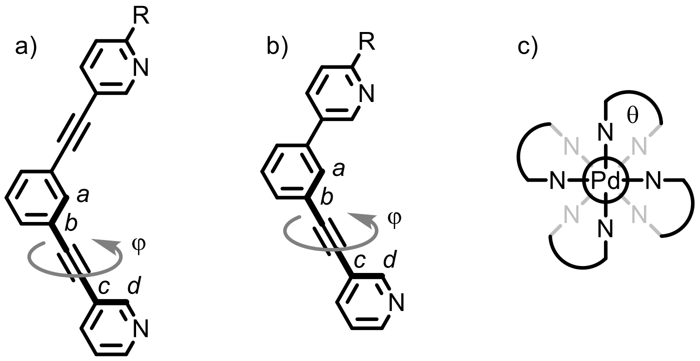

L R = H
1 R = Me

5 R = H
6 R = Me

Figure S137 Alkyne twist angles (ϕ) were measured as the torsion angle defined by the carbon atoms *a*, *b*, *c* and *d* for both a) ligands **L** and **1**, and b) **5** and **6**. c) Ligand twist angles (θ) were measured as the torsion angle defined by the two Pd-N bonds of an individual ligand.

Table S3 Ligand distortion and intrinsic porosity parameters obtained from SCXRD and calculated structures. (a) SCXRD data reported by Hooley and co-workers;[13] (b) SCXRD data reported by Lusby and co-workers.[14]

Ligand	Isomer	Ligand twist angles (θ)	Alkyne twist angles (ϕ)	Pd···Pd distance	Pore diameter
**L**[(a)]	–	1.00°<br>0.78°	3.01°<br>3.12°<br>6.99°<br>7.21°	11.852 Å	6.65 Å
**L**[(b)]	–	0.56°<br>0.86°	6.42°<br>8.47°<br>12.53°<br>22.40°	12.215 Å	6.35 Å
**1**	**D**	41.83°	17.24°<br>29.65°	11.866 Å	6.26 Å
**5**	**C**	25.82°<br>27.25°<br>27.53°<br>27.54°	0.55°<br>2.41°<br>4.21°<br>47.70°	9.697Å	4.36 Å
**6**	**C**	39.48°<br>40.01°<br>42.05°<br>42.81°	11.16°<br>18.05°<br>18.32°<br>57.06°	9.904 Å	4.48 Å

References

[1] S. A. Gamage, J. A. Spicer, G. W. Rewcastle, J. Milton, S. Sohal, W. Dangerfield, P. Mistry, N. Vicker, P. A. Charlton and W. A. Denny, *J. Med. Chem.*, 2002, **45**, 740–743.
[2] SHELXTL v5.1, Bruker AXS, Madison, WI, 1998.
[3] SHELX-2013, G.M. Sheldrick, *Acta Cryst.*, 2015, **C71**, 3-8.
[4] A.L. Spek (2003, 2009) PLATON, A Multipurpose Crystallographic Tool, Utrecht University, Utrecht, The Netherlands. See also A.L. Spek, *Acta. Cryst.*, 2015, **C71**, 9-18.
[5] M. D. Hanwell, D. E. Curtis, D. C. Lonie, T. Vandermeersch, E. Zurek and G. R. Hutchison, *J. Cheminform.*, 2012, **4**, 17.
[6] C. Bannwarth, S. Ehlert and S. Grimme, *J. Chem. Theory Comput.* 2019, **153**, 1652-1671
[7] M. Bursch, H. Neugebauer and S. Grimme, *Angew. Chem. Int. Ed.*, 2019, **58**, 11078-11087.
[8] Gaussian 16, Revision C.01, M. J. Frisch, G. W. Trucks, H. B. Schlegel, G. E. Scuseria, M. A. Robb, J. R. Cheeseman, G. Scalmani, V. Barone, G. A. Petersson, H. Nakatsuji, X. Li, M. Caricato, A. V. Marenich, J. Bloino, B. G. Janesko, R. Gomperts, B. Mennucci, H. P. Hratchian, J. V. Ortiz, A. F. Izmaylov, J. L. Sonnenberg, D. Williams-Young, F. Ding, F. Lipparini, F. Egidi, J. Goings, B. Peng, A. Petrone, T. Henderson, D. Ranasinghe, V. G. Zakrzewski, J. Gao, N. Rega, G. Zheng, W. Liang, M. Hada, M. Ehara, K. Toyota, R. Fukuda, J. Hasegawa, M. Ishida, T. Nakajima, Y. Honda, O. Kitao, H. Nakai, T. Vreven, K. Throssell, J. A. Montgomery, Jr., J. E. Peralta, F. Ogliaro, M. J. Bearpark, J. J. Heyd, E. N. Brothers, K. N. Kudin, V. N. Staroverov, T. A. Keith, R. Kobayashi, J. Normand, K. Raghavachari, A. P. Rendell, J. C. Burant, S. S. Iyengar, J. Tomasi, M. Cossi, J. M. Millam, M. Klene, C. Adamo, R. Cammi, J. W. Ochterski, R. L. Martin, K. Morokuma, O. Farkas, J. B. Foresman, and D. J. Fox, Gaussian, Inc., Wallingford CT, 2016.
[9] S. Grimme, S. Ehrlich and L. Goerigk, *J. Comput. Chem.*, 2011, **32**, 1456–1465.
[10] W. M. Bloch, Y. Abe, J. J. Holstein, C. M. Wandtke, B. Dittrich and G. H. Clever, *J. Am. Chem. Soc.*, 2016, **138**, 13750–13755; R. Zhu, W. M. Bloch, J. J. Holstein, S. Mandal, L. V. Schäfer and G. H. Clever, *Chem. Eur. J.*, 2018, **24**, 12976–12982.
[11] T. A. Young, V. Martí-Centelles, J. Wang, P. J. Lusby and F. Duarte, *ChemRxiv Preprint*, 2019, DOI: 10.26434/chemrxiv.9576644.
[12] M. Miklitz and K. E. Jelfs, *J. Chem. Inf. Model.*, 2018, **58**, 2387–2391.
[13] P. Liao, B. W. Langloss, A. M. Johnson, E. R. Knudsen, F. S. Tham, R. R. Julian and R. J. Hooley, *Chem. Commun.*, 2010, **46**, 4932–4934.
[14] D. P. August, G. S. Nichol and P. J. Lusby, *Angew. Chem. Int. Ed.*, 2016, **55**, 15022–15026.
